# Use of a p64 MW Flow Diverter with Hydrophilic Polymer Coating (HPC) and Prasugrel Single Antiplatelet Therapy for the Treatment of Unruptured Anterior Circulation Aneurysms: Safety Data and Short-term Occlusion Rates

**DOI:** 10.1007/s00270-022-03153-8

**Published:** 2022-05-13

**Authors:** V. Hellstern, M. Aguilar Pérez, E. Henkes, E. Donauer, C. Wendl, H. Bäzner, O. Ganslandt, H. Henkes

**Affiliations:** 1 Neuroradiologische Klinik, Kopf- und Neurozentrum, Klinikum Stuttgart, Stuttgart, Germany; 2 Klinik Für Neurochirurgie, Mediclin Krankenhaus Plau Am See, Plau Am See, Germany; 3 Institut Für Röntgendiagnostik, Zentrum Für Neuroradiologie, Universitätsklinikum Regensburg, Regensburg, Germany; 4 Neurologische Klinik, Neurozentrum, Klinikum Stuttgart, Stuttgart, Germany; 5 Neurozentrum, Neurochirurgische Klinik, Klinikum Stuttgart, Stuttgart, Germany; 6 Medizinische Fakultät der Universität Duisburg-Essen, Essen, Germany

**Keywords:** Aneurysm, Flow diverter, Anti-thrombogenic coating, Prasugrel, Single antiplatelet therapy

## Abstract

**Purpose:**

To assess the safety and short-term occlusion rates in procedures using the p64 MW hydrophilic polymer-coated (HPC) flow diverter (FD) with prasugrel single antiplatelet therapy (SAPT) for the treatment of anterior circulation saccular aneurysms.

**Methods:**

We retrospectively identified patients who underwent treatment of one or more intracranial anterior circulation saccular aneurysms between March 2020 and December 2021 with a p64 MW HPC FD and prasugrel SAPT with verified P2Y12 platelet receptor inhibition. Patients diagnosed with fusiform, dissecting, or recently ruptured aneurysms were excluded. Periprocedural and postprocedural complications, clinical outcomes, and angiographic follow-up results were evaluated.

**Results:**

One hundred and two patients with 132 intracranial aneurysms met the inclusion criteria. Previous or concomitant treatments (e.g., coil occlusion) had been performed on 18 of these aneurysms. The technical success rate (i.e., implantation of the intended FD) was 100% with an average of 1.1 devices implanted per patient. Periprocedural and postprocedural complications occurred in 13.6% and 6.8% of these patients, respectively. No mortality or permanent clinical deterioration (i.e., modified Rankin scale score ≥ 3) were reported. Early follow-up digital subtraction angiography revealed aneurysmal occlusion rates of 72.6% and 83.8% at four and nine months, respectively.

**Conclusions:**

The implantation of a p64 MW HPC FD with prasugrel SAPT is safe and results in rapid, reliable and effective aneurysmal occlusion.

**Supplementary Information:**

The online version contains supplementary material available at 10.1007/s00270-022-03153-8.

## Introduction

While flow diverter (FD) implantation has become a standard method for managing intracranial aneurysms (IAs) [1, 2], patients undergoing this procedure typically require concomitant dual antiplatelet therapy (DAPT). While DAPT can result in bleeding complications, including intracranial hemorrhage [3, 4], its discontinuation after the deployment of an uncoated device may result in thrombotic occlusion of the implant and ultimately ischemic stroke [3]. Five FDs with anti-thrombogenic surface modifications are currently available (Suppl. Table 1). Among these, only the p64 and p48 MW hydrophilic polymer-coating (HPC) devices include instructions for use with single antiplatelet therapy (SAPT). While anecdotal reports from our group suggested that implantation of these devices with aspirin SAPT might be hazardous [5], improved results were achieved in studies with our collaborators in which prasugrel was used as SAPT [6, 7]. This study aimed to evaluate the safety and efficacy of p64 MW HPC FD implants used to treat unruptured aneurysms of the anterior circulation with prasugrel SAPT. We present a series of postprocedural magnetic resonance imaging (MRI) findings as a surrogate marker for safety and occlusion rates as a key criterion for efficacy.

## Methods

### Adherence to Ethical Standards and Patient Population

We retrospectively reviewed our prospectively-maintained database to identify patients with saccular unruptured aneurysms of the anterior circulation who were implanted with at least one p64 MW HPC FD with prasugrel SAPT between March 2020 and December 2021. Patient selection was based on criteria similar to those used in the ongoing COATING trial (ClinicalTrials.gov Identifier: NCT04870047). The patients enrolled in our study had not been considered in previous publications. The ethical standards directing this study as well as the inclusion and exclusion criteria are included in Suppl. Table 2.

We recorded demographic data, the results of platelet function tests, anatomical features, location of the aneurysm, procedural and postprocedural complications, and clinical and angiographic outcomes. The results of follow-up (FU) studies performed through March 18, 2022, were also included.

### Endovascular Procedure

All patients signed the informed consent at least 24 h before the procedure. All treatments were performed under general anesthesia. A 7F short sheath (Terumo, Tokyo, Japan) with a 7F guiding catheter (Guider Softip, Stryker, Kalamazoo, MI, USA) was introduced via right-sided femoral access in combination with an intermediate catheter (e. g., Navien A + 058, Medtronic, Dublin, Ireland). Heparin (3000 IU unfractionated heparin) was administered intravenously after groin puncture. All pressurized flushing solutions were heparinized (5000 IU/L). The p64 MW HPC was deployed via a Prowler Select Plus (Cerenovus, Irvine, CA, USA), a Trevo Pro 18 (Stryker Neurovascular, Fremont, CA, USA), or a Headway 21 (MicroVention Terumo, Aliso Viejo, CA, USA) microcatheter. The diameter and length of the p64 MW HPC were chosen based on two- and three-dimensional measurements of the diameter of the parent artery, the distance between the proximal and distal landing zones, the discrepancies between the diameters of the two landing zones, and the width of the aneurysm neck.

### Medication

All patients received a loading dose of 30 mg prasugrel as SAPT at least three days prior to the procedure followed by doses of 10 mg *per os* (PO) per day. Effective anti-platelet responses were determined with a Multiplate Analyzer (Roche Diagnostics, Mannheim, Germany) or VerifyNow (Accriva, San Diego, CA, USA). The device was not implanted until adequate platelet receptor inhibition was confirmed. None of the patients in this study required a shift to another medication. Values ranged from 0 to 119 P2Y12 reaction units (PRU; mean,19.9). Adenosine diphosphate (ADP) receptor inhibition values ranged from 1 to 45 units (U; mean, 10.8).

Postprocedural medication included 10 mg prasugrel PO daily for six months. The response tests were repeated approximately two weeks after the procedure. If tests revealed excessive inhibition (e. g., PRU < 10) the prasugrel dosage was reduced, and the patient was re-evaluated two weeks later with a goal of PRU < 100. After six months, the patient was switched to 100 mg aspirin PO daily with an overlap of three days. According to our institutional protocol, patients at high risk of ischemic complications were treated with subcutaneous injections of low-molecular-weight (LMW) heparin (3,000 IU Mono-Embolex, Aspen Germany, Germany) in two daily doses for four to six weeks after the procedure.

### Data Collection and Follow-up (FU)

Patients underwent magnetic resonance imaging (MRI) that included T2 and diffusion-weighted imaging (DWI) within 48 h after the procedure. The results were rated as “no new lesions,” “1–5 small focal DWI lesions,” “ > 5 small focal DWI lesions,” or “territorial T2 lesions, infarct.”

Patients were scheduled for clinical and angiographic examinations at time points that included very early (1–69 days, F0), early (70–180 days, F1), mid-term (181–500 days, F2), and long-term FU (500–1000 days, F3) intervals. Assessment of aneurysm occlusion was evaluated according to the O'Kelly-Marotta (OKM) scale [8]; adequate occlusion was defined as OKM C or OKM D. Neurological examinations were performed during the periprocedural period (≤ 24 h), the postprocedural period (> 24 h and ≤ 30 days), and during FU (> 30 days). Results were recorded using the modified Rankin Scale (mRS) [9].

## Results

### Patient Demographics and Aneurysm Characteristics

One hundred and two patients (median age 58.1 years, 75.5% female) with 132 aneurysms met the inclusion criteria (see Suppl. Table 2). The mean aneurysm dome size, mean neck width, and mean width/neck ratio were 4.8 mm, 3.4 mm, and 1.3, respectively. 48.5% of the aneurysms were classified as very small (< 4 mm), 33.3% were identified as small (4–7 mm) and 10.6% were identified as medium-sized (7–10 mm); only 7.6% (*n* = 10) were classified as large (> 10 mm). Of these, 18 had been treated previously. 65.9% of the aneurysms were across the internal carotid artery (ICA). Others were identified across the anterior cerebral artery (ACA), the anterior commuting artery (AcomA), or the middle cerebral artery (MCA) (Table 1).

**Table 1:** Characteristics of patients and aneurysms

Characteristics	Number	Percentage
Patients/aneurysms	102/132	
Sex (m/f)	25/77	24.5/75.5
Age (years)	Range 21–84; mean 58.1	
Previously treated	18/132 (17 × Coiling, 1 × pCONUS + Coiling)	13.6
*Location*		
ICA–cavernous/petrous segment	12	9.1
ICA–ophthalmic segment	27	20.5
ICA–hypophyseal artery aneurysms	25	18.9
ICA–posterior communicating segment	19	14.4
ICA–choroidal segment	4	3.0
ACA	5	3.8
AcomA	9	6.8
MCA	11	8.3
MCA bifurcation	20	15.2
*Aneurysm size*		
Very small (< 4 mm)	64	48.5
Small (4–7 mm)	44	33.3
Medium (7–10 mm)	14	10.6
Large > 11 mm	10	7.6
Neck	0.6**–**13 mm; mean 3.4 mm	
Width/neck ratio	0.7**–**2.8; mean 1.3	

ICA, internal carotid artery; ACA, anterior cerebral artery; AcomA, anterior communicating artery; MCA, middle cerebral artery

### Treatment and Complications

A total of 147 p64 MW HPC FD devices were implanted. 92% of the patients were treated with a single FD; 8 aneurysms required two FDs and each one aneurysm required 3 and 6 FDs, respectively. Adjunctive devices were used in eight cases, including five aneurysms with coils. Coils were placed in larger aneurysms in which the use of an FD device might be expected to result in delayed occlusion. An aneurysm of the AcomA was treated by exclusion of the AcomA by implanting a FD in each ACA- due to the small caliber of the parent vessel a p48 MW HPC FD was placed in one ACA while a p64 MW HPC was placed in the other ACA. Two contour devices (Cerus Endovascular Inc, Fremont, USA) were implanted in two large, wide-necked ICA aneurysms as “bridging devices” that provided access to the MCA and facilitated FD implantation.

Intraprocedural complications were identified in 18 of the 132 aneurysms treated (Table 2). In seven of these aneurysms, the implanted FD foreshortened significantly after deployment which led to incomplete coverage and required implantation of a second device. In 10 cases, the deployed FD did not open properly; complete expansion and appropriate positioning of the FD was achieved by temporary deployment of a stent-retriever or by inflation of a compliant balloon. One patient developed an ICA dissection caused by the guiding catheter. We encountered no intraprocedural or periprocedural thromboembolic or hemorrhagic complications.

**Table 2:** Complication rates and DWI lesions based on location for the 132 aneurysms treated

	ICA –cavernous/petrous segment	ICA –superior hypophyseal artery	ICA –ophthalmic segment	ICA – PcomA	ICA –anterior choroidal artery	ACA	AcomA-Complex	MCA –M1	MCA –bifurcation
Number of aneurysms treated	12 (9.1%)	25 (18.9%)	27 (20.5%)	19 (14.4%)	4 (3.0%)	5 (3.8%)	9 (6.8%)	11 (8.3%)	20 (15.2%)
Periprocedural complications	2 (1.5%)	5 (3.8%)	6 (4.5%)	4 (3.0%)	0	0	0	0	1 (0.8%)
Postprocedural complications	0	0	0	0	0	0	0	1 (1.0%)	6 (5.9%)
Delayed complications	1(1.0%)	0	0	0	1 (1.0%)	0	0	0	0
No DWI lesions	8	21	13	11	1	1	0	6	12
1–5 DWI lesions	3	3	7	5	1	2	4	0	4
> 5 DWI	1	1	7	3	2	2	5	3	4
No MRI available	0	0	0	0	0	0	0	2	0
Permanent deterioration of the mRS score	1 (1.0%)	0	0	0	1 (1.0%)	0	0	0	4 (3.9%)

ICA, internal carotid artery; PcomA, posterior communicating artery; ACA, anterior cerebral artery; AcomA, anterior communicating artery; MCA, middle cerebral artery, DWI, diffusion-weighted imaging; mRS: modified Rankin scale, MRI, magnetic resonance imaging

Postprocedural complications (24 h–30 days) were observed in seven patients. Four patients suffered ischemia secondary to jailed MCA branches, including one patient who did not adhere to the prescribed SAPT. Two patients developed an in-stent-thrombosis due to SAPT non-adherence. Both patients were treated with mechanical thrombectomy and intra-arterial infusion of eptifibatide; complete recanalization of the occluded FD was achieved. One patient presented with severe headaches 15 days after the FD implantation. MRI revealed multiple small contrast-enhancing foci with surrounding edema in the cerebral hemisphere ipsilateral to the treated aneurysm in a pattern which was interpreted as a foreign body reaction [10]. Headaches and cerebral lesions resolved in response to high-dose steroids.

Two patients presented with delayed complications (> 30 days). One patient suffered a right frontal intraparenchymal hemorrhage two months postprocedure that was treated conservatively, leading to a good recovery. One patient developed an oculomotor nerve palsy seven weeks after treatment of a large ipsilateral ICA aneurysm. Although the aneurysm was angiographically obliterated (OKM D) 90 days after treatment, cranial nerve palsy persisted.

Overall, nine of the 102 patients developed postprocedural or delayed complications; among these, three patients experienced transient neurological deficits only. Six patients developed permanent albeit minor neurological deficits. Only four of the patients exhibited deteriorations in mRS from 0 to ≥ 2.

One hundred and ten pre-discharge MRI examinations were available for our review. 55.5% revealed no DWI lesions. Fewer than five DWI lesions were detected in 28 MRIs; ≥ 5 DWI lesions were detected in another 21 scans. No territorial infarcts or subarachnoid or intracerebral hemorrhages were detected.

### Angiographic Results

**Fig. 1:**
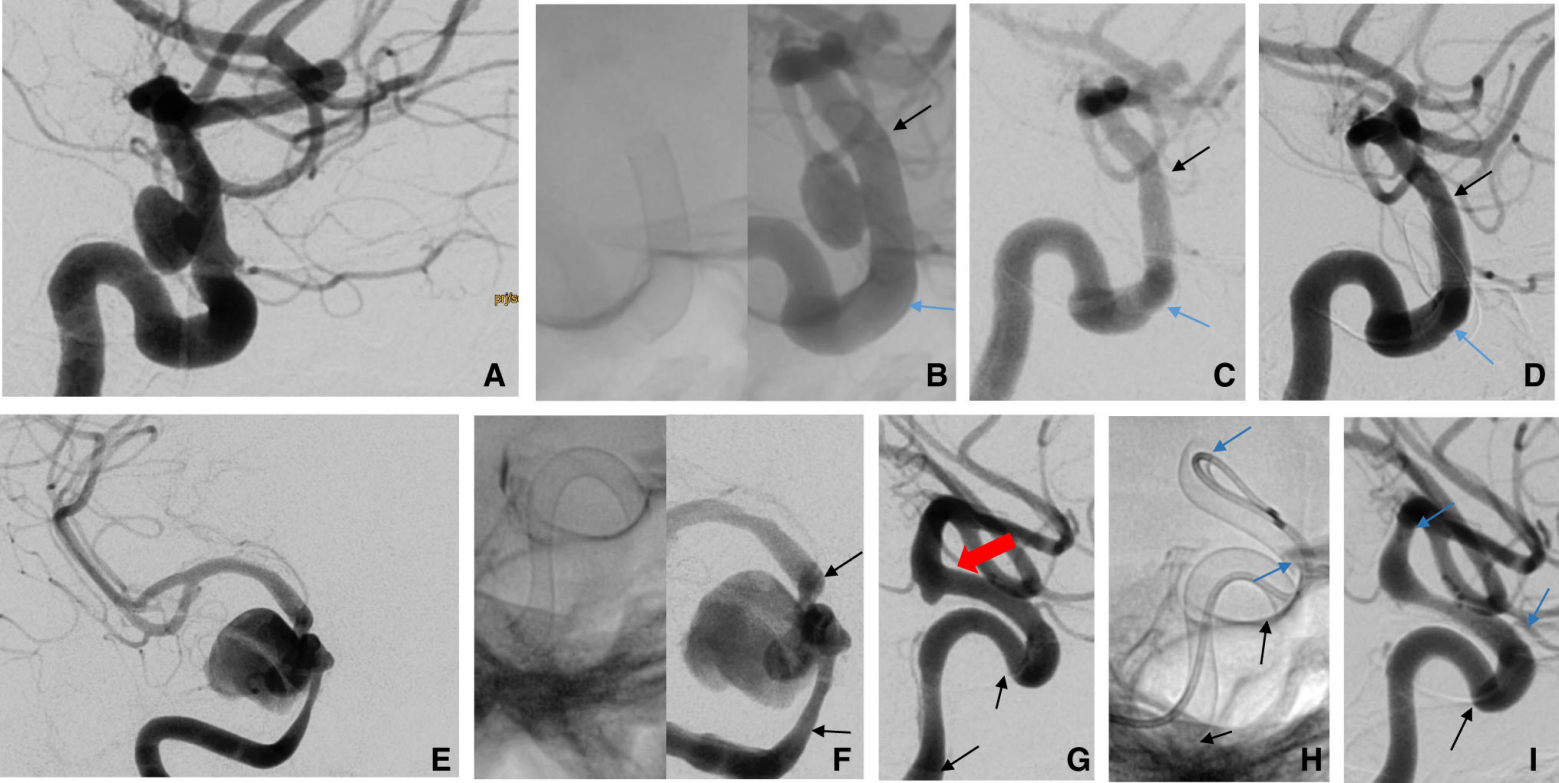
Digital subtraction angiography (DSA). a–d DSA imaging of an aneurysm of the supraclinoid segment of the left internal carotid artery (ICA). **a** Lateral projection prior to placement of a flow diverter (FD). **b** Lateral projection after deployment of a 3/15 mm p64 MW HPC; left, a non-contrast-enhanced image demonstrating complete opening of the FD and right, contrast-enhanced image after deployment of the FD showing complete coverage of the aneurysm neck. Follow-up (FU) angiography at **c** 39 days and **d** 251 days postprocedure. The lateral view documents the complete occlusion of the aneurysm (OKM D). In panels b–d, the distal and proximal ends of the FD are indicated by the black and blue arrows, respectively. **e–i** DSA imaging of a large symptomatic aneurysm of the cavernous segment of the right ICA and a small aneurysm at the origin of the choroidal artery. **e** Posterior-anterior projection before placement of the first FD. (f) Lateral (non-contrast-enhanced) image and anterior–posterior projection after deployment of a 3.5/21 mm p64MW HPC FD (black arrows indicating the ends of the device). **g** FU angiography at 56 days postprocedure revealed the complete occlusion of the cavernous aneurysm (OKM D) (black arrows indicating the ends of the FD device; the small aneurysm at the choroidal segment of the ICA is as indicated by the red arrow). **h** This lesion was treated in the same session with a 4/12 mm p64 MW HPC FD (blue arrows indicating the ends of the second FD; non-contrast-enhanced lateral view after the deployment of a second FD). **i** FU angiography at 91 days postprocedure with a lateral view that revealed complete occlusion of both aneurysms (OKM D) with mild intimal hyperplasia in the distal FD (blue arrows at the ends of the distal FD and a black arrow indicating the distal end of the proximal FD; the proximal end of this latter FD is not shown in this picture)

Angiographic results at FU0 revealed that most aneurysms (14/26) were completely occluded (OKM D; Fig. 1). Nine aneurysms underwent no change (OKM A) while four exhibited subtotal filling (OKM B); no neck remnants (OKM C) were detected. Two aneurysms exhibited in-stent stenosis (ISS), one mild and one moderate (loss of < 50% or 50–75% of the lumen, respectively).

Ninety-five aneurysms were evaluated at FU1 and 67.4% revealed complete occlusion (OKM D; *n* = 64), 5.3% neck remnants (OKM C; *n* = 5), 11.6% subtotal filling (OKM B; *n* = 11),  and 15.8% no change (OKM A; *n* = 15). Two of the unchanged ICA aneurysms exhibited incomplete coverage due to FD foreshortening that required re-treatment. Eighteen of these aneurysms developed mild (*n* = 13), moderate (*n* = 1), or severe (*n* = 4) ISS, the latter with loss of > 75% of the lumen. These patients were switched to DAPT; one asymptomatic patient underwent balloon angioplasty because of the absence of sufficient collaterals and recovered with no complications. All other patients remained asymptomatic with adequate responses to SAPT.

Complete occlusion (OKM D) was noted for 58 of 74 of these aneurysms at FU2; an additional 4 achieved OKM C. Seven aneurysms exhibited subtotal filling (OKM B) while five remained unchanged (OKM A). Additional FU angiographic data are not yet available.

**Fig. 2:**
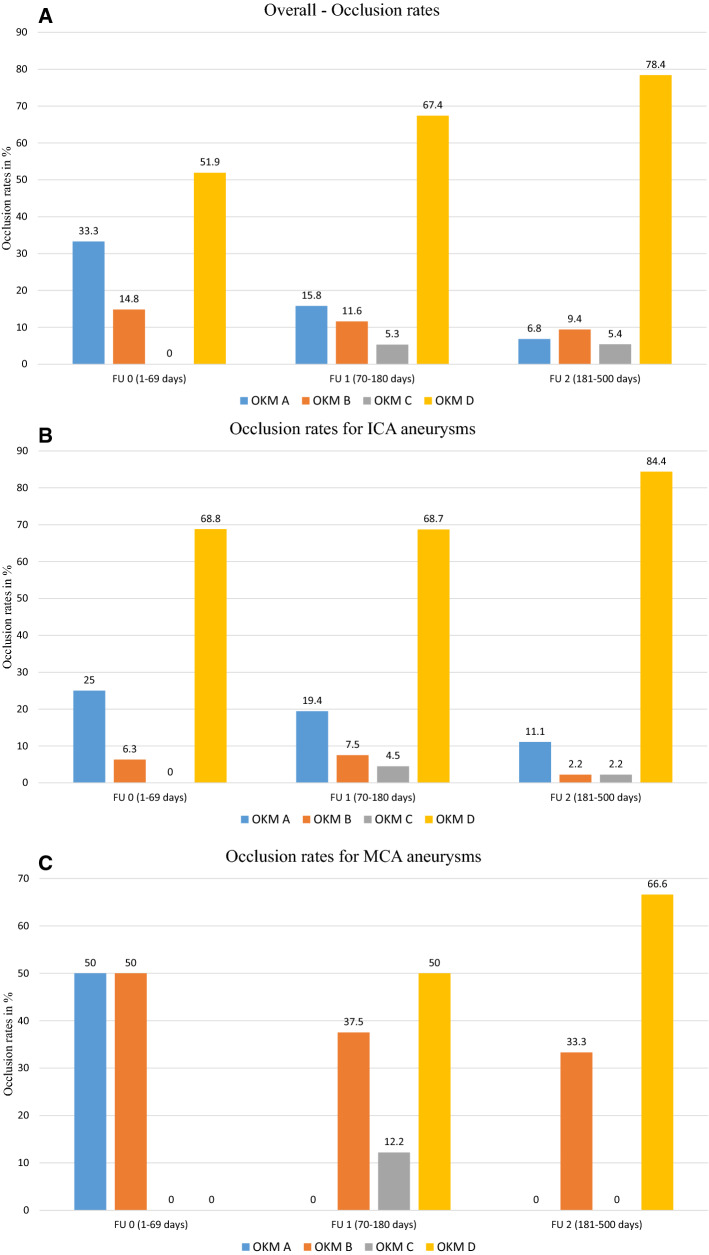

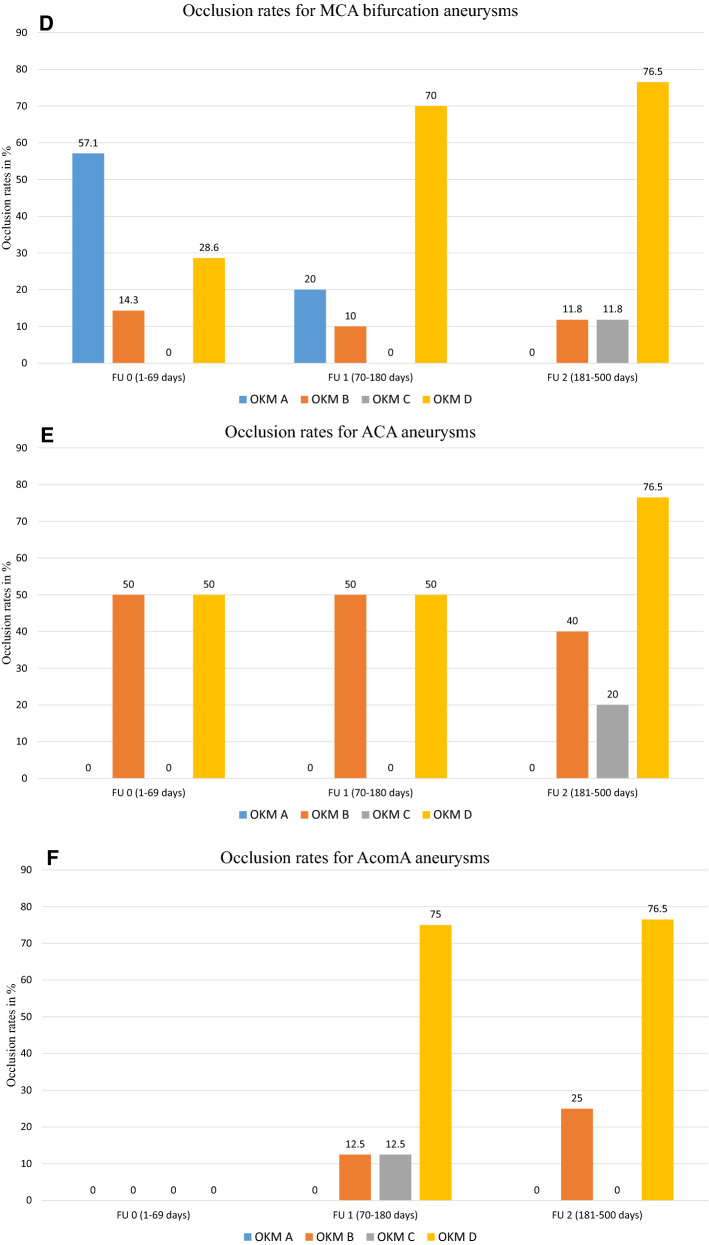
Progressive increase in aneurysmal occlusion over time. Occlusion was evaluated (**a**) overall, and for (**b**) ICA aneurysms; (**c**) MCA aneurysms; (**d**) MCA bifurcation aneurysms; (**e**) ACA aneurysms; and (**f**) AcomA aneurysms. FU, follow-up; OKM, O'Kelly-Marotta; ICA, internal carotid artery; MCA, middle cerebral artery; ACA, anterior cerebral artery; AcomA, anterior communicating artery

The overall occlusion rates and occlusion rates associated with each location are shown in Fig. 2 and Table 3, respectively.

**Table 3:** Occlusion rates detected from early and intermediate angiographic FU data based on the location of the aneurysm

Site of aneurysm	ICA	ACA	AcomA	MCA–M1	MCA–bifurcation
Number of aneurysms	87 (65.9%)	5 (3.8%)	9 (6.8%)	11 (8.3%)	20 (15.2%)
Available follow-up (FU) information at three months (FU0)*	16	1	0	2	7
Mean time to FU (days)	41	50	0	56	44
Complete occlusion rate at FU0 (OKM D)	11 (68.8%)	0	0	0	2 (28.6%)
Neck remnants at FU0 (OKM C)	0	0	0	0	0
Aneurysm remnants at FU0 (OKM B)	1 (6.3%)	1 (100%)	0	1 (50%)	1 (14.3%)
Aneurysm unchanged at FU0 (OKM A)^†^	4 (25%)	0	0	1 (50%)	4 (57.1%)
Available FU information at three months (FU1)*	67	2	8	8	10
Mean time to FU (days)	107	106	122	117	106
Complete occlusion rate at FU1 (OKM D)	46 (68.7%)	1 (50%)	6 (75%)	4 (50%)	7 (70%)
Neck remnants at FU1 (OKM C)	3 (4.5%)	0	1 (12.5%)	1 (12.5%)	0
Aneurysm remnants at FU1 (OKM B)	5 (7.5%)	1 (50%)	1 (12.5%)	3 (37.5%)	1 (10%)
Aneurysm unchanged at FU1 (OKM A)	13 (19.4%)*	0	0	0	2 (20%)
Available FU information at nine months (FU2)*	45	5	4	3	17
Mean time to FU (days)	231	267	279	216	278
Complete occlusion rate at FU2 (OKM D)	38 (84.4%)	2 (40%)	3 (75%)	2 (66.7%)	13 (76.5%)
Neck remnants at FU2 (OKM C)	1 (2.2%)	1 (20%)	0	0	2 (11.8%)
Aneurysm remnants at FU2 (OKM B)	1 (2.2%)	1 (33.3%)	1 (25%)	1 (33.3%)	2 (11.8%)
Aneurysm unchanged at FU2 (OKM A)	5 (11.2%)	0	0	0	0

*In this patient population, very early FU (FU0) was 7–69 days, with a mean of 50 days; early FU (FU1) was 73–178 days with a mean of 116 days and intermediate FU (FU2) was 192–490 days with a mean of 287 days.

^†^Two of the four aneurysms identified as unchanged at FU0 were no longer covered by the FD and required re-treatment. ICA, internal carotid artery; PcomA, posterior communicating artery; ACA, anterior cerebral artery; AcomA, anterior communicating artery; MCA, middle cerebral artery; FU, follow-up

## Discussion

This study aimed to determine the safety and efficacy of implantation of p64 MW HPC FDs under prasugrel SAPT from data collected from a large series of patients diagnosed with IAs. The treatment of small IAs is justified by our institutional experience which has revealed that the median diameter of a ruptured aneurysm is 5 mm [11]. We successfully performed coil embolization on 188 aneurysms during the enrollment phase of this study. However, according to our institutional treatment strategy even saccular aneurysms are treated with FD in a high frequency as our findings suggest that the results of implantation of FD devices might be superior as compared to coil embolization. Similar experiences have also already been published in literature some time ago [12].

Here, we report an overall aneurysm occlusion rate (OKM D) of 67.4% after approximately three months; an additional 5.3% of these lesions present with neck remnants only (OKM C). After nine months, the occlusion rate had increased to 78.4%, with an additional 5.4% of the aneurysms reported as nearly occluded. Thus, our results revealed that adequate occlusion was achieved in 72.7% and 83.8% of the cases at three and nine months, respectively. Occlusion rates were even higher for isolated ICA aneurysms. While 68.8% of these lesions were already occluded at six weeks, 84.4% were completely occluded and 2.2% were nearly occluded at the nine-month time point. Similarly, Bhogal et al. [7] reported complete occlusion of 62.5% of the aneurysms after 183 days in a series of 23 Mongolian patients who were treated with p64 MW HPC FDs and SAPT. The occlusion rates in our population were also similar to those reported for the uncoated p64 device [13, 14].

The results reported in this series suggest that this strategy results in improved occlusion rates. Our findings can be compared to results from the SAFE study that revealed a complete aneurysmal occlusion rate of 73.3% after 12 months [15]. Hanel et al. [16] recently published results of the PREMIER study in which the Pipeline device (see Suppl. Table 1) was used to treat 141 (95% ICA) aneurysms, resulting in complete aneurysmal occlusion in 81.9% of cases at 12 months. Similarly, Taschner et al. [17] published a study using the Derivo embolization device (DED, Acandis, Pforzheim, Germany) that reported a complete occlusion rate of 82% at 18 months. Thus, our findings compare favorably to those previously reported, as they demonstrate higher occlusion rates at earlier FU dates.

MCA-bifurcation aneurysms exhibited occlusion rates (FU1, 70%; and FU2, 76.5%) that were comparable to overall values. Most literature reports note that this type of aneurysms frequently responds with reduced occlusion rates and longer times to aneurysm occlusion. For example, (Aguilar) Pérez et al. [13] reported occlusion rates for MCA-bifurcation aneurysms treated with an uncoated p64 FD device at 65% and 83% at three and nine months, respectively.

All seven symptomatic postprocedural ischemic complications in this series occurred in patients presenting with MCA or MCA-bifurcation aneurysms. However, we note that one of these patients was diagnosed with a foreign body reaction and the three others were not compliant with the SAPT regimen; thus, only 9.7% of the patients treated for MCA or MCA-bifurcation aneurysms displayed postprocedural complications while maintained on adequate SAPT. These findings represent a significant improvement over previously published data on the use of FD devices for MCA aneurysms. For example, (Aguilar) Pérez et al. [13] and Diestro et al. [18] reported postprocedural complication rates of 15% and 16.7%, respectively, in patients with MCA-bifurcation aneurysms. However, we recognize that patients with an anticipated higher risk for ischemic complications were treated daily with subcutaneous LMW heparin for 4–6 weeks after FD treatment to reduce the risk of ischemic complications. Taking into consideration both the occlusion and complication rates, treatment of MCA aneurysms with the p64 MW HPC and sufficient SAPT appears to be safer and at least equally effective to the use of other FD devices. Treatment of complex MCA aneurysms remains challenging and optimal strategies remain to be determined.

Although FD implantation under SAPT raises concerns regarding thromboembolic complications, these were not exhibited by any of the patients in our series who maintained adequate SAPT. Bhogal et al. [7] reported similar findings. In addition, recent reports describe the use of both p48 and p64 MW HPC FDs in acutely ruptured aneurysms. Collectively, the results of these studies emphasize that the use of HPC-coated FDs is safe in patients that maintain sufficient SAPT [5, 19, 20].

There are no data available at this time that provide a direct comparison of the occlusion rates in patients treated with a p64 MW HPC FD under DAPT *versus* SAPT. The closest comparison is the occlusion rate of the uncoated p64 under DAPT and the p64MW HPC under SAPT. The p64-Diversion study reported complete aneurysm occlusion and neck remnants only in 71.7% and 4.5% of patients, respectively, after approximately 5 months, and 83.7% and 2.3%, respectively, after approximately 12 months [14]. A second large series reported by (Aguilar) Pérez et al. [13] presented similar findings. The percentage of complete occlusion identified in each of these studies was notably lower than those presented in our study and was associated with a fraction with neck remnants remaining at the same time points. Again, this shows a trend toward more rapid aneurysm occlusion but needs to be studied further as we report here a significantly smaller population.

Postprocedural MRIs revealed no ischemic lesions in more than half of the patients. DWI lesions were more prevalent in patients who underwent p64 MW HPC implantation for MCA/MCA-bifurcation aneurysms. By contrast, the results of a systematic review and meta-analysis published by Bond et al. [18] revealed that DWI lesions developed in up to 67% of patients presenting with IAs after FD implantation. Silent ischemic events were reported in as many as 50.9–90% of cases involving the implantation of the uncoated Pipeline FD [21–24]. In studies involving the newest version of the Pipeline FD (a phosphorylcholine polymer-coated Pipeline Shield), silent ischemic lesions were reported in 18.2% of the patients, all of whom were treated with DAPT [25].

Recently several studies focused on outcomes associated with other coated FD devices (e.g., p48 MW HPC or the Pipeline Shield) have been published [5–7, 19, 20, 26–37]. The devices used and the study outcomes are outlined in Supplementary Table 3.

## Limitations

The main limitations of this study are those inherent in retrospective data collection. This study reports our experience with the implantation of a single type of FD device at one center only. The applicability of these results to other FDs and FD-associated procedures remains unknown. Furthermore, it is not clear whether the results of this study could be extrapolated to fusiform, ruptured, or posterior circulation aneurysms as well as SAPT provided by other drugs. The safety profile and occlusion rate of the p64 MW HPC under prasugrel SAPT needs to be compared to results obtained using the uncoated variant of this device implanted under DAPT. To this end, COATING is a recently-initiated randomized controlled trial designed to address this issue with a non-inferiority hypothesis of the SAPT arm.

## Conclusion

Use of p64MW HPC FDs with prasugrel SAPT for saccular aneurysms of the anterior circulation is both safe and effective.

## Supplementary Information

Below is the link to the electronic supplementary material.

Supplementary file1 (DOCX 12 kb)

Supplementary file2 (DOCX 13 kb)

Supplementary file3 (DOCX 13 kb)

Supplementary file4 (PPTX 71164 kb)
