# Supplementary material for: Use of a p64 MW Flow Diverter with Hydrophilic Polymer Coating (HPC) and Prasugrel Single Antiplatelet Therapy for the Treatment of Unruptured Anterior Circulation Aneurysms: Safety Data and Short-term Occlusion Rates
Source: Cardiovasc Intervent Radiol. 2022 May 13;45(9):1364–74. doi: 10.1007/s00270-022-03153-8 (PMC9458553; doi:10.1007/s00270-022-03153-8)
Supplement: Supplementary file 1 — Supplementary file1 (DOCX 12 kb) [file 270_2022_3153_MOESM1_ESM.docx]

**Suppl. Table 1** Flow-diverter (FD) devices with anti-thrombogenic surface modifications

| Device | Company | Location |
| --- | --- | --- |
| Pipeline Vantage with Shield Technology | Medtronic | Dublin, Ireland |
| Derivo Heal | Acandis | Pforzheim, Germany |
| FredX | MicroVention | Aliso Viejo, California, USA |
| p48 MW HPC | phenox | Bochum, Germany |
| p64 MW HPC | phenox | Bochum, Germany |
