# Supplementary material for: Use of a p64 MW Flow Diverter with Hydrophilic Polymer Coating (HPC) and Prasugrel Single Antiplatelet Therapy for the Treatment of Unruptured Anterior Circulation Aneurysms: Safety Data and Short-term Occlusion Rates
Source: Cardiovasc Intervent Radiol. 2022 May 13;45(9):1364–74. doi: 10.1007/s00270-022-03153-8 (PMC9458553; doi:10.1007/s00270-022-03153-8)
Supplement: Supplementary file 2 — Supplementary file2 (DOCX 13 kb) [file 270_2022_3153_MOESM2_ESM.docx]

**Suppl. Table 2** Ethical standards and criteria for patient inclusion or exclusion

| **Parameter** | **Description** | **Additional details** |
| --- | --- | --- |
| **Ethical standards** | Used FD devices with “Conformité Européenne” (CE) certificate. |  |
|  | Patients were provided with information on available treatment modalities’ | Conservative, microsurgical, and endovascular options were presented.  Relevant details (e.g., stent-assisted coiling, use of other FD devices with DAPT, among others) were provided. |
|  | Written informed consent was obtained. | Forms included permission for retrospective data use and publication in a de-identified format.  The signed forms inform the patients about their rights according to the European General Data Protection Regulation. |
|  | Approvals obtained. | No patient withdrew his or her permission to use the data.  The responsible ethics committee was consulted for the retrospective data analysis and publication, which was granted in written form.  (Reference No.: F-2018-110) |
| **Inclusion criteria** | ≥18 years of age. |  |
|  | Diagnosis of IAs | A saccular, unruptured, or recanalized lesion, or >30 days after aneurysm rupture.  One or two or more adjacent aneurysms that were treated with at least one FD  Selection of FD based on an interdisciplinary decision by team members from neurology, neurosurgery, and neuroradiology.  Presented with mRS ≤ 2. |
| **Exclusion criteria** | On long-term anti-coagulation or requires DAPT |  |
|  | Past medical history | Underwent an unrelated surgical procedure during the previous 30 days.  Diagnosed with intracranial hemorrhage (ICH) or subarachnoid hemorrhage (SAH) during the 30 days prior to the procedure.  Stenting of the ipsilateral carotid artery during the previous three months.  Target aneurysm previously treated with a stent or FD device.  Anticipating the need to treat another aneurysm during the 30 days after the initial procedure. |
|  | Associated lesions and patient characteristics | Confirmed stenosis of the parent artery of the lesion targeted by the FD device.  Blister-like, fusiform, or dissecting aneurysm or a lesion associated with an arteriovenous malformation.  Any known contraindication precluding the use of a p64 MW HPC device.  Significant sensitivity to contrast agents or known allergy to heparin and/or prasugrel.  Renal failure (i.e., serum creatinine > 2.5 mg/dL or glomerular filtration rate < 30 mL/min.  Women who were pregnant or breastfeeding. |
|  | Patient unable to document informed consent | Unable to approve the use of de-identified data for publication. |
