# Supplementary material for: Use of a p64 MW Flow Diverter with Hydrophilic Polymer Coating (HPC) and Prasugrel Single Antiplatelet Therapy for the Treatment of Unruptured Anterior Circulation Aneurysms: Safety Data and Short-term Occlusion Rates
Source: Cardiovasc Intervent Radiol. 2022 May 13;45(9):1364–74. doi: 10.1007/s00270-022-03153-8 (PMC9458553; doi:10.1007/s00270-022-03153-8)
Supplement: Supplementary file 3 — Supplementary file3 (DOCX 13 kb) [file 270_2022_3153_MOESM3_ESM.docx]

**Suppl. Table 3** A summary of clinical studies featuring coated FD devices.

| **Authors** | **Year** | **Device** | **Anti-platelet therapy** | **Outcomes** |
| --- | --- | --- | --- | --- |
| Pierot et al. [26] | 2021 | p48 MW HPC | DAPT | Complete occlusion in 90% of 28 aneurysm patients. |
| Bhogal et al. [6] | 2020 | p48 MW HPC | SAPT | Few complications and occlusion rates that were similar to those associated with other FD devices. |
| Lobsien et al. [19] | 2021 | p48 and p64 MW HPC | DAPT and SAPT | The use of coated FD devices with SAPT is safe and effective in a carefully selected cohort of patients with SAH. |
| Guzzardi et al. [20] | 2020 | FD coated with a hydrophilic polymer | SAPT (prasugrel) | No intraprocedural stent thrombosis, rebleeding, or retreatment was required in a series of seven patients treated for SAH. |
| Aguilar-Perez et al. [5] | 2020 | p48 MW HPC | SAPT | A small patient series reporting the effective use of these modalities in patients presenting with ruptured aneurysms. |
| Bhogal et al. [7] | 2021 | p48 and p64 MW HPC | SAPT (prasugrel) | Occlusion rate of 64.7% at three months postprocedure in 24 patients with unruptured aneurysms; safety and efficacy similar to that seen using uncoated devices in patients under DAPT. |
| de Castro-Afonso [27] | 2021 | p48 MW HPC | SAPT (prasugrel) | The use of this coated device under SAPT monotherapy was safe and effective in a series of 21 patients, including four with asymptomatic ischemic brain lesions on MRI. |
| de Castro-Afonso [28] | 2021 | p48 MW HPC | SAPT (aspirin) | As above in citation [27] although associated with an increased risk of ischemic complications. |
| Manning et al. [29] | 2019 | Pipeline Shield | SAPT (aspirin) | A small study that presented results that were similar to those from FD implantation studies under DAPT in patients with SAH. |
| Agnoletto et al. [30] | 2018 | Pipeline Shield | DAPT | Good results when used to treat fusiform MCA aneurysms. |
| Trivelato et al. [31] | 2020 | Pipeline Shield | DAPT | A multi-center study documenting complete occlusion and periprocedural complication rates of 79.7% and 7.3%, respectively, at six months. |
| Rice et al. [32] | 2020 | Pipeline Shield | DAPT | The Shield study. A prospective, post-market, multi-center study documenting complete occlusion rates of 70.8% and 77.2% at six months and one year, respectively, in a series of 204 patients. |
| Martinez-Galdamez et al. [33] | 2019 | Pipeline Shield | DAPT | Documented the safety and efficacy of this regimen. |
| Martinez-Galdamez et al. [34] | 2017 | Pipeline Shield | DAPT | High rates of technical success without any major intraprocedural complications. |
| Capuzzo et al. [35] | 2022 | Pipeline Shield | DAPT | High rates of technical success without any major intraprocedural complications in a series of 10 patients. Suggestions were made regarding the optimization of the anti-platelet regimen. |
| Hanel et al. [36] | 2017 | Pipeline Shield | SAPT (aspirin) | Use of this strategy to treat ruptured vertebral aneurysms. Insufficient SAPT resulted in occlusion of the FD albeit without clinical sequelae. |
| Guerrero et al. [37] | 2018 | Pipeline Shield | DAPT | Reported endothelialization of the FD device at eight weeks after implantation with no thrombus formation. |
