# Supplementary material for: Use of a p64 MW Flow Diverter with Hydrophilic Polymer Coating (HPC) and Prasugrel Single Antiplatelet Therapy for the Treatment of Unruptured Anterior Circulation Aneurysms: Safety Data and Short-term Occlusion Rates
Source: Cardiovasc Intervent Radiol. 2022 May 13;45(9):1364–74. doi: 10.1007/s00270-022-03153-8 (PMC9458553; doi:10.1007/s00270-022-03153-8)
Supplement: Supplementary file 4 — Supplementary file4 (PPTX 71164 kb) [file 270_2022_3153_MOESM4_ESM.pptx]

## Slide 1
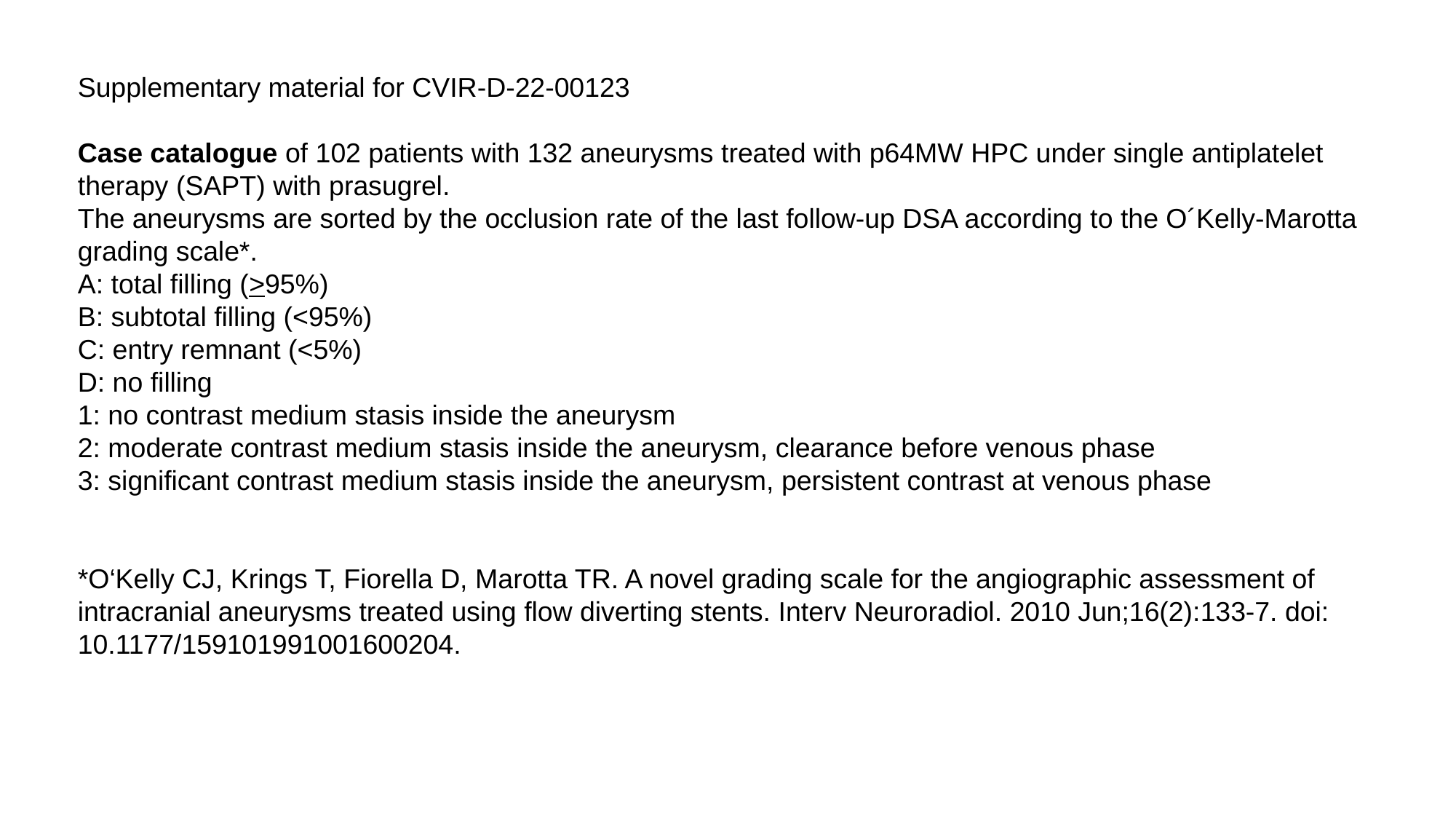

Supplementary material for CVIR-D-22-00123
Case catalogue of 102 patients with 132 aneurysms treated with p64MW HPC under single antiplatelet therapy (SAPT) with prasugrel.
The aneurysms are sorted by the occlusion rate of the last follow-up DSA according to the O´Kelly-Marotta grading scale*.
A: total filling (>95%)
B: subtotal filling (<95%)
C: entry remnant (<5%)
D: no filling
1: no contrast medium stasis inside the aneurysm
2: moderate contrast medium stasis inside the aneurysm, clearance before venous phase
3: significant contrast medium stasis inside the aneurysm, persistent contrast at venous phase
*O‘Kelly CJ, Krings T, Fiorella D, Marotta TR. A novel grading scale for the angiographic assessment of intracranial aneurysms treated using flow diverting stents. Interv Neuroradiol. 2010 Jun;16(2):133-7. doi: 10.1177/159101991001600204.

## Slide 2
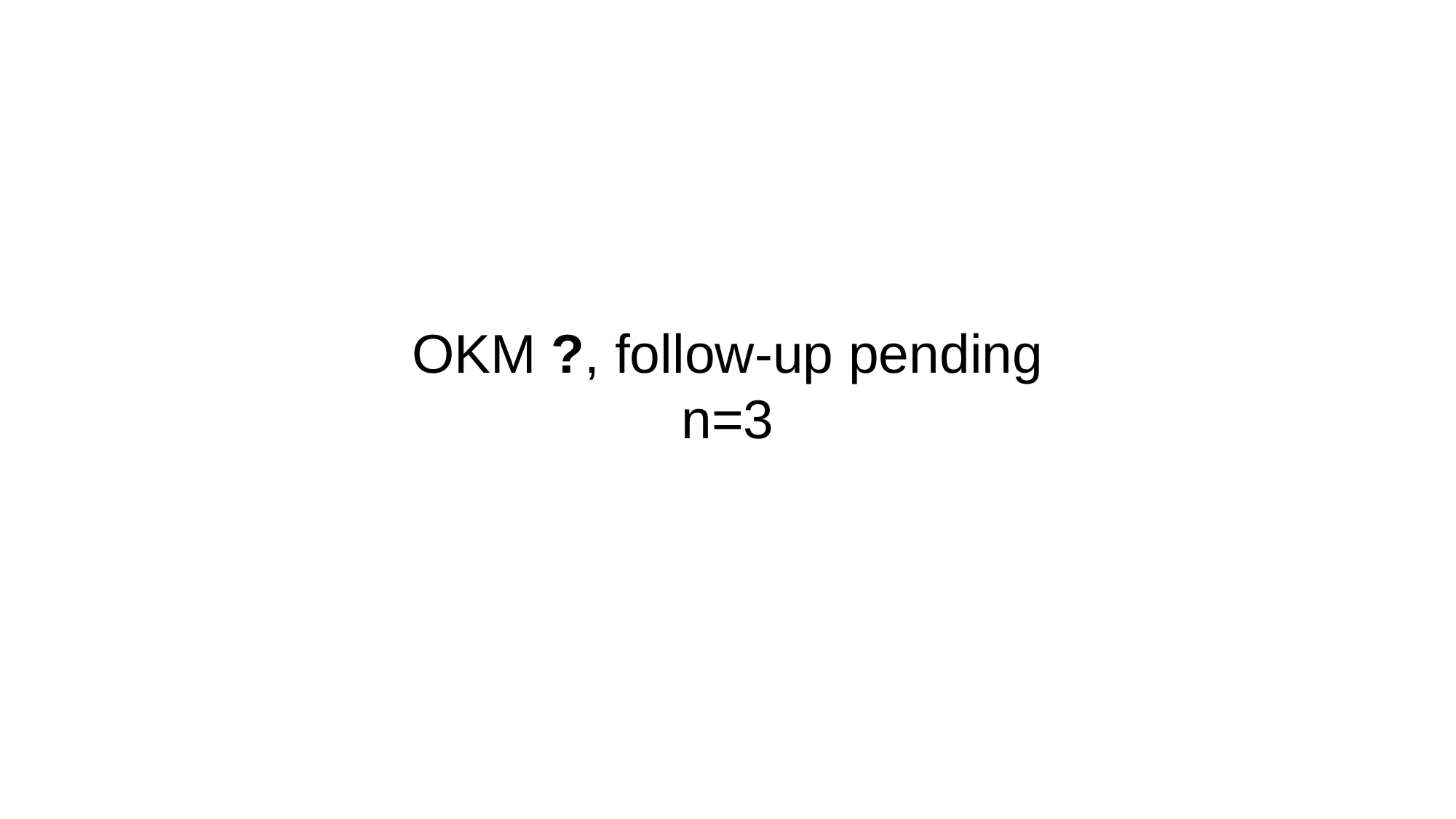

OKM ?, follow-up pending
n=3

## Slide 3
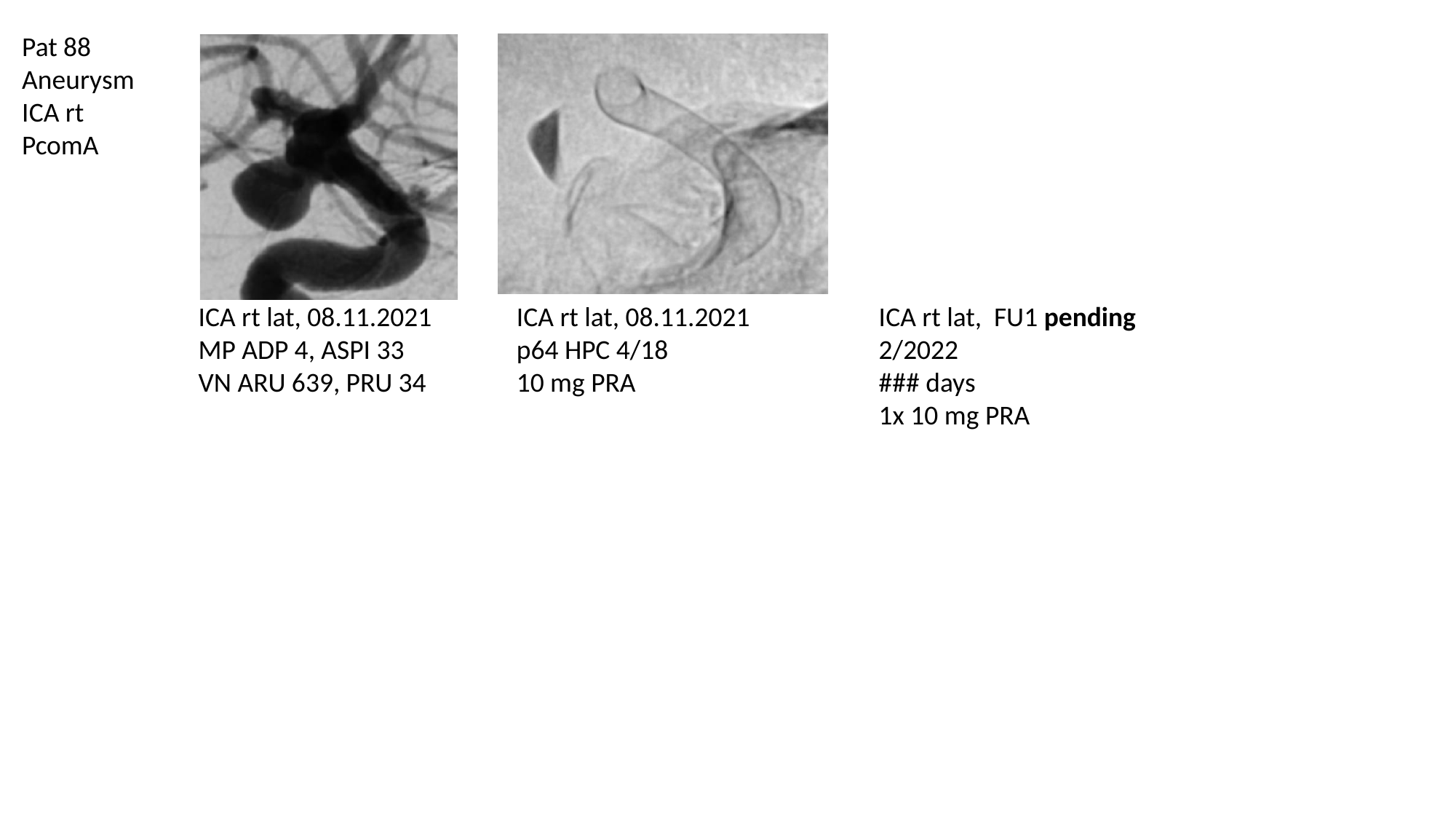

Pat 88
Aneurysm
ICA rt
PcomA
M2
M1
ICA rt lat, 08.11.2021
p64 HPC 4/18
10 mg PRA
ICA rt lat, FU1 pending
2/2022
### days
1x 10 mg PRA
ICA rt lat, 08.11.2021
MP ADP 4, ASPI 33
VN ARU 639, PRU 34

## Slide 4
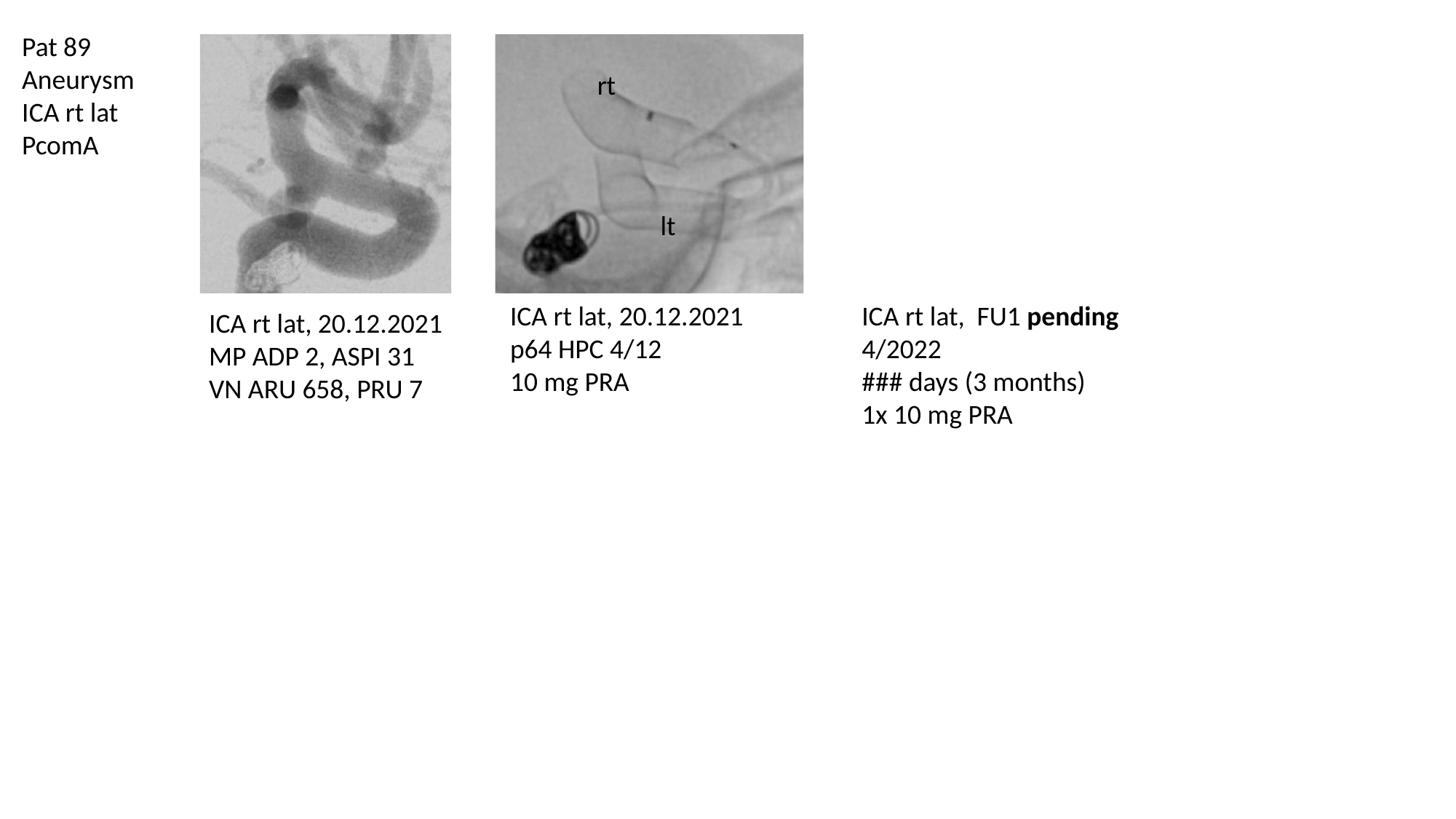

Pat 89
Aneurysm
ICA rt lat
PcomA
rt
lt
ICA rt lat, 20.12.2021
p64 HPC 4/12
10 mg PRA
ICA rt lat, FU1 pending
4/2022
### days (3 months)
1x 10 mg PRA
ICA rt lat, 20.12.2021
MP ADP 2, ASPI 31
VN ARU 658, PRU 7

## Slide 5
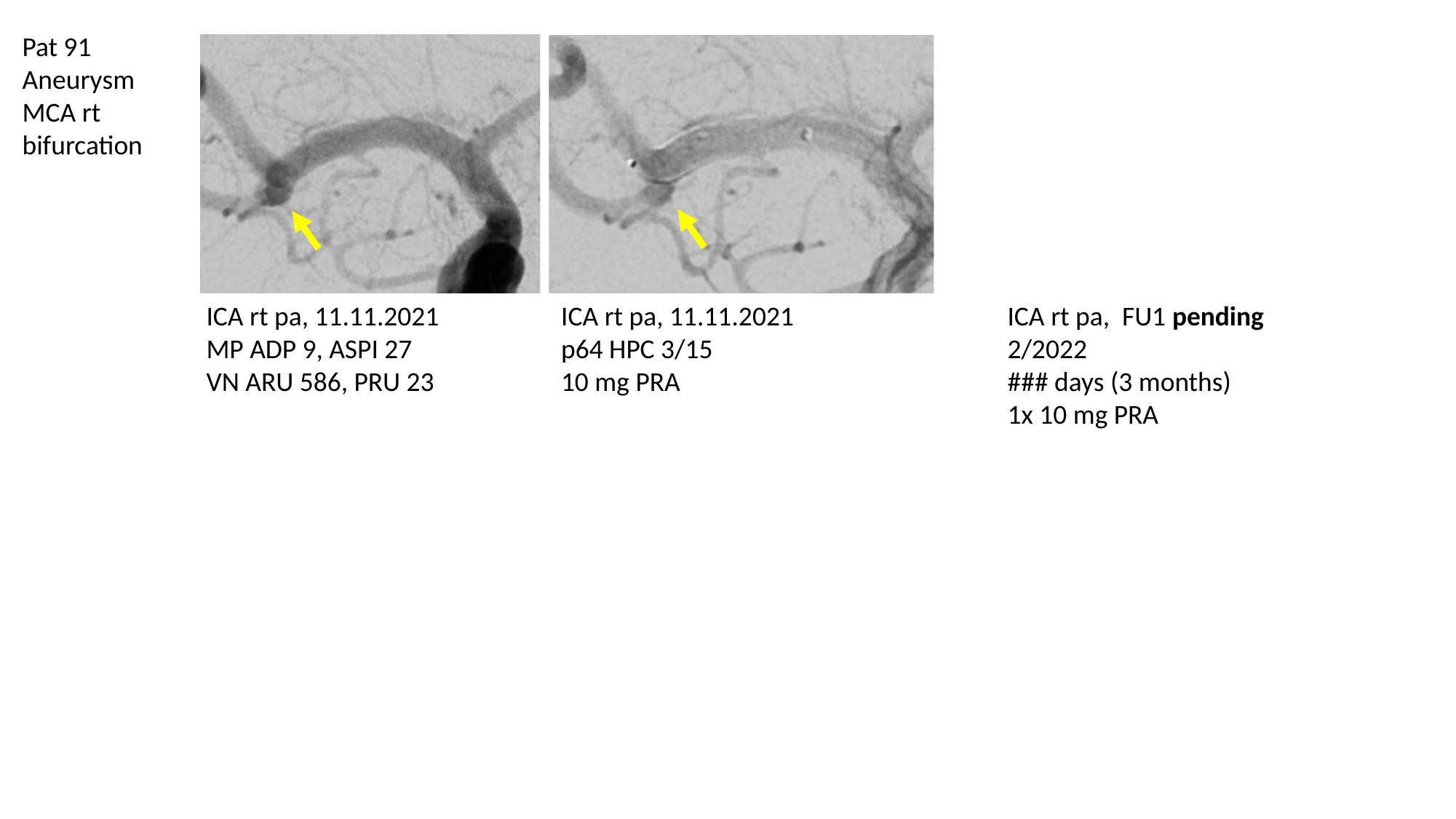

Pat 91
Aneurysm
MCA rt
bifurcation
ICA rt pa, 11.11.2021
p64 HPC 3/15
10 mg PRA
ICA rt pa, FU1 pending
2/2022
### days (3 months)
1x 10 mg PRA
ICA rt pa, 11.11.2021
MP ADP 9, ASPI 27
VN ARU 586, PRU 23

## Slide 6
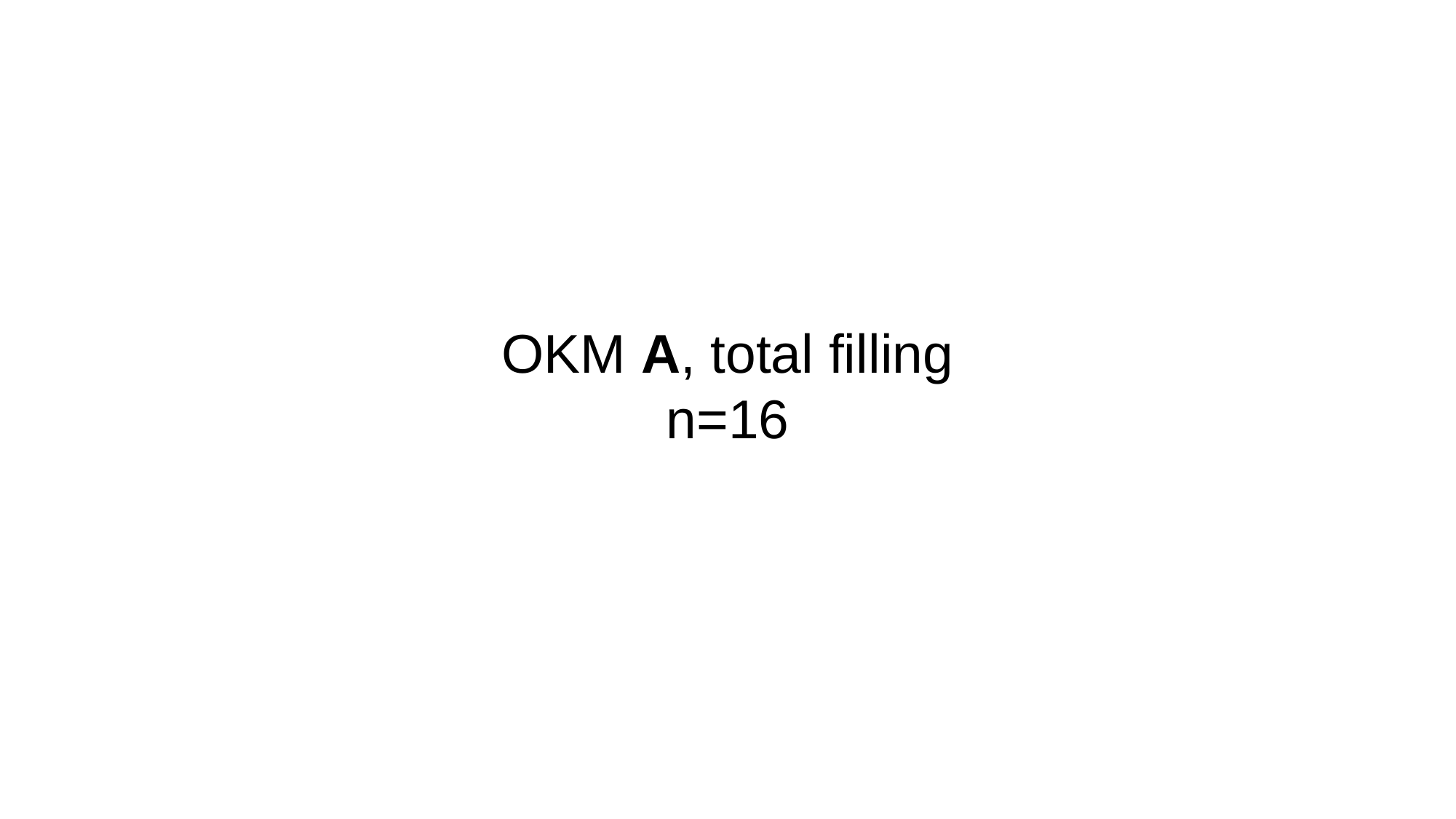

OKM A, total filling
n=16

## Slide 7
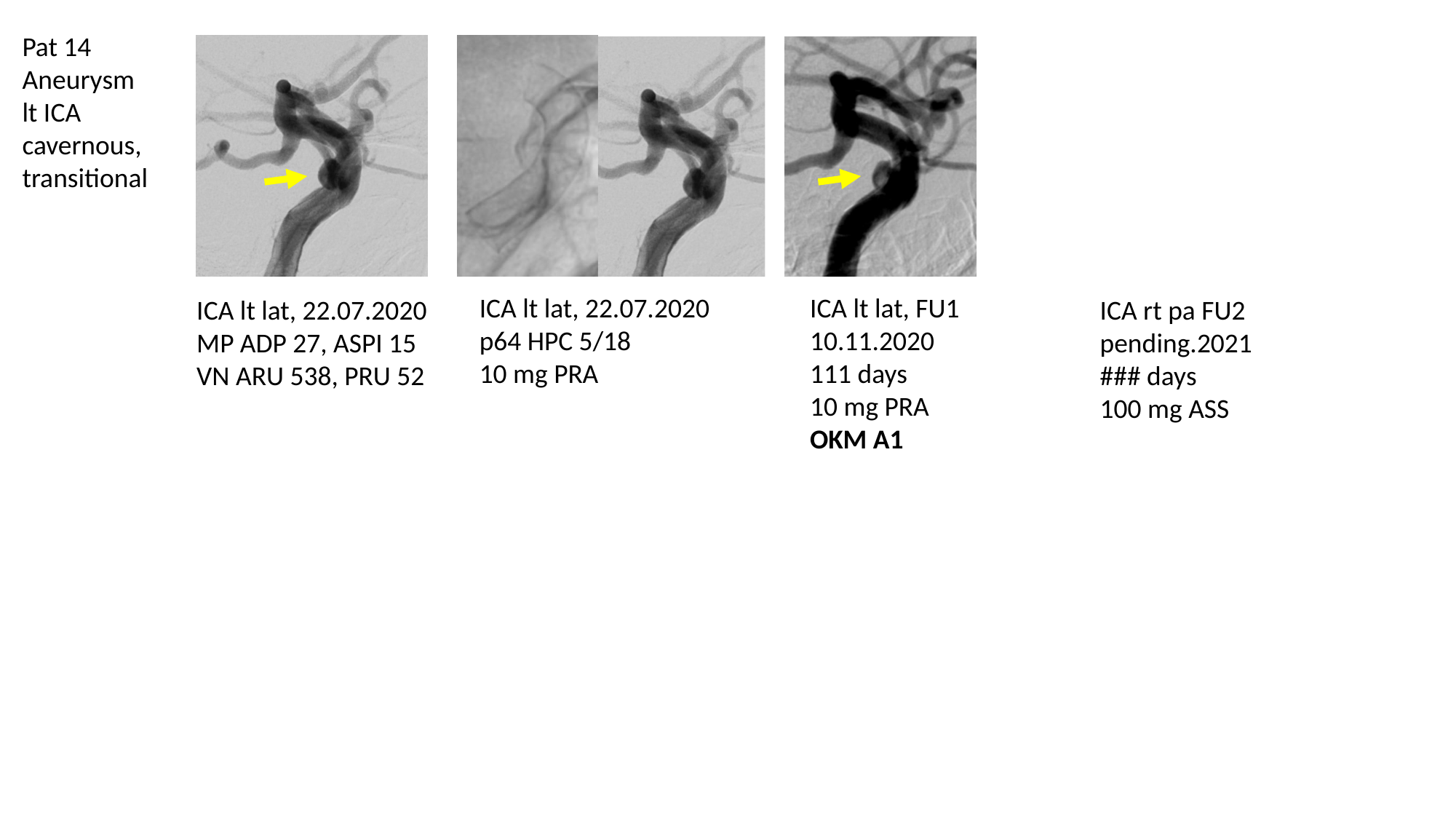

Pat 14
Aneurysm
lt ICA
cavernous,
transitional
ICA lt lat, 22.07.2020
p64 HPC 5/18
10 mg PRA
ICA lt lat, FU1
10.11.2020
111 days
10 mg PRA
OKM A1
ICA lt lat, 22.07.2020
MP ADP 27, ASPI 15
VN ARU 538, PRU 52
ICA rt pa FU2
pending.2021
### days
100 mg ASS

## Slide 8
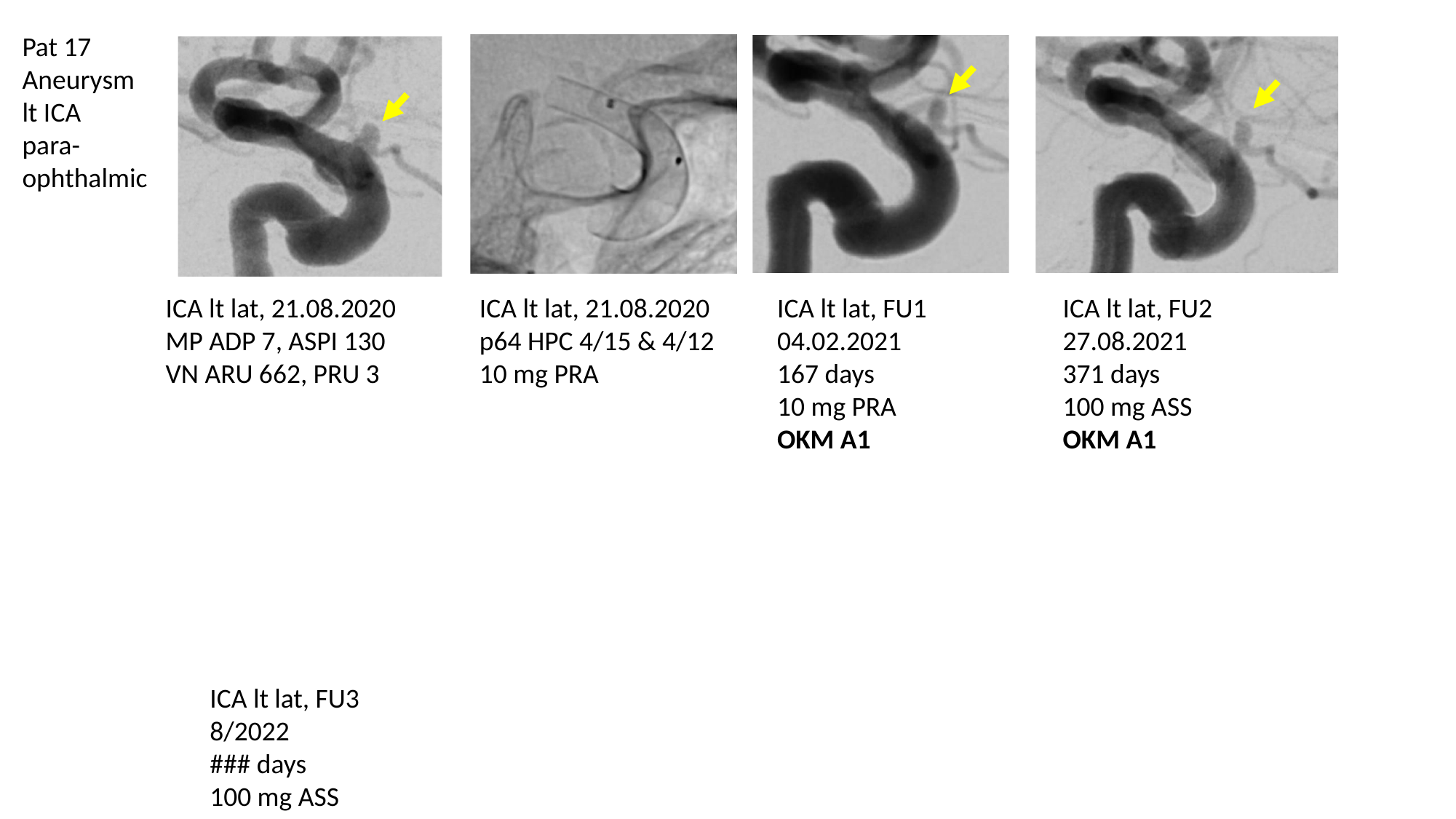

Pat 17
Aneurysm
lt ICA
para-
ophthalmic
ICA lt lat, 21.08.2020
MP ADP 7, ASPI 130
VN ARU 662, PRU 3
ICA lt lat, FU1
04.02.2021
167 days
10 mg PRA
OKM A1
ICA lt lat, FU2
27.08.2021
371 days
100 mg ASS
OKM A1
ICA lt lat, 21.08.2020
p64 HPC 4/15 & 4/12
10 mg PRA
ICA lt lat, FU3
8/2022
### days
100 mg ASS

## Slide 9
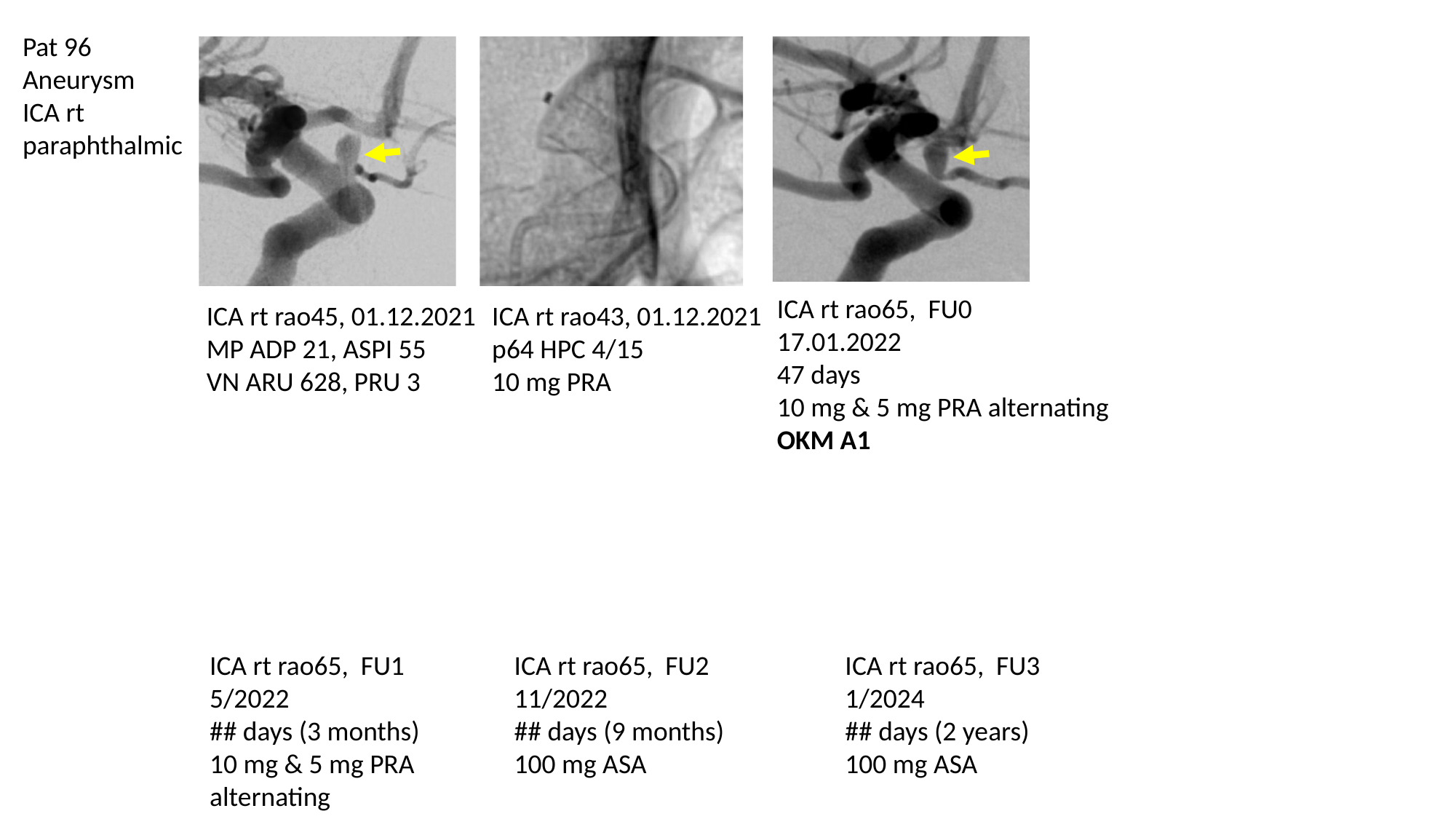

Pat 96
Aneurysm
ICA rt
paraphthalmic
ICA rt rao65, FU0
17.01.2022
47 days
10 mg & 5 mg PRA alternating
OKM A1
ICA rt rao45, 01.12.2021
MP ADP 21, ASPI 55
VN ARU 628, PRU 3
ICA rt rao43, 01.12.2021
p64 HPC 4/15
10 mg PRA
ICA rt rao65, FU3
1/2024
## days (2 years)
100 mg ASA
ICA rt rao65, FU1
5/2022
## days (3 months)
10 mg & 5 mg PRA
alternating
ICA rt rao65, FU2
11/2022
## days (9 months)
100 mg ASA

## Slide 10
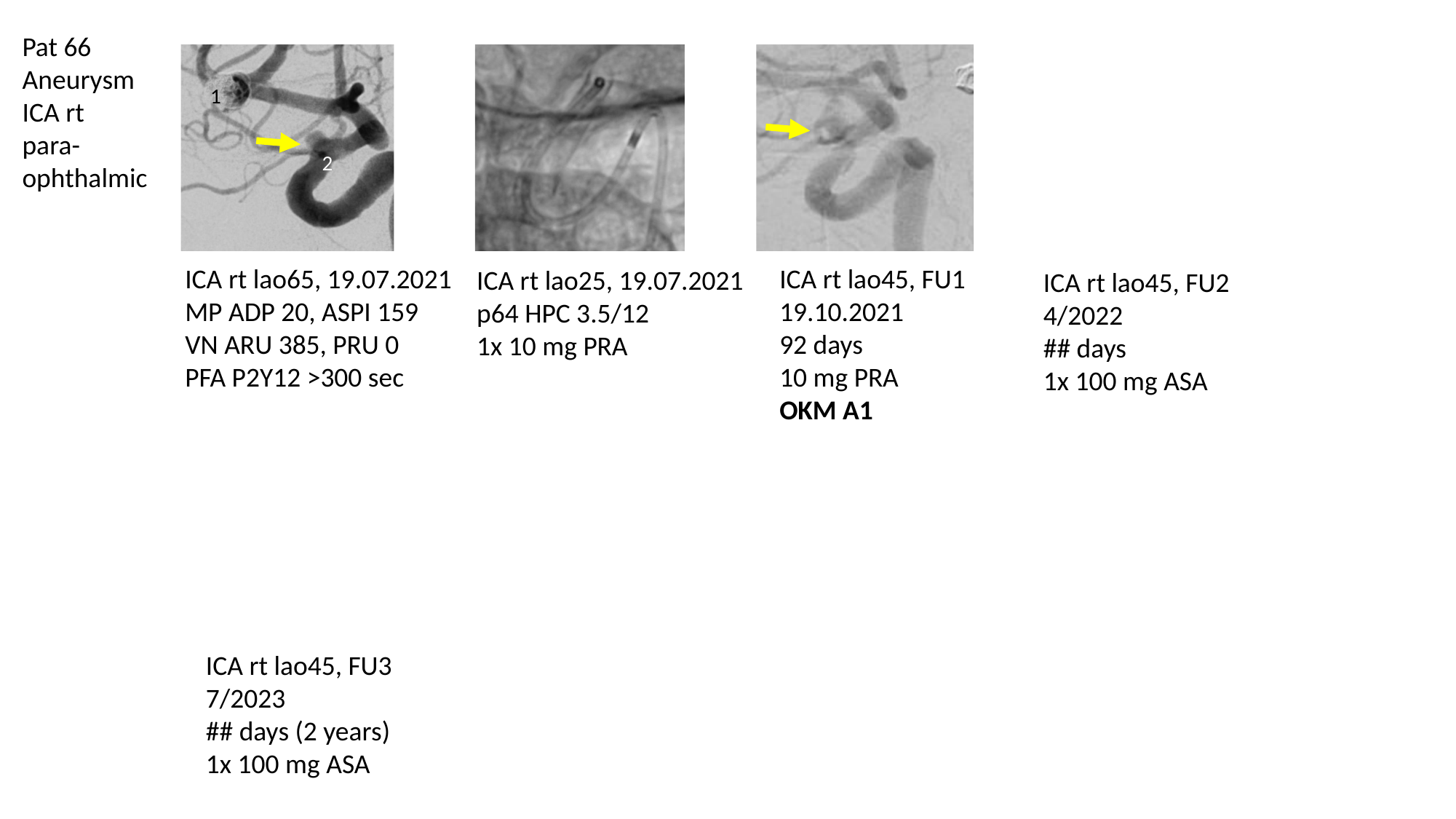

Pat 66
Aneurysm
ICA rt
para-
ophthalmic
1
2
ICA rt lao65, 19.07.2021
MP ADP 20, ASPI 159
VN ARU 385, PRU 0
PFA P2Y12 >300 sec
ICA rt lao45, FU1
19.10.2021
92 days
10 mg PRA
OKM A1
ICA rt lao25, 19.07.2021
p64 HPC 3.5/12
1x 10 mg PRA
ICA rt lao45, FU2
4/2022
## days
1x 100 mg ASA
ICA rt lao45, FU3
7/2023
## days (2 years)
1x 100 mg ASA

## Slide 11
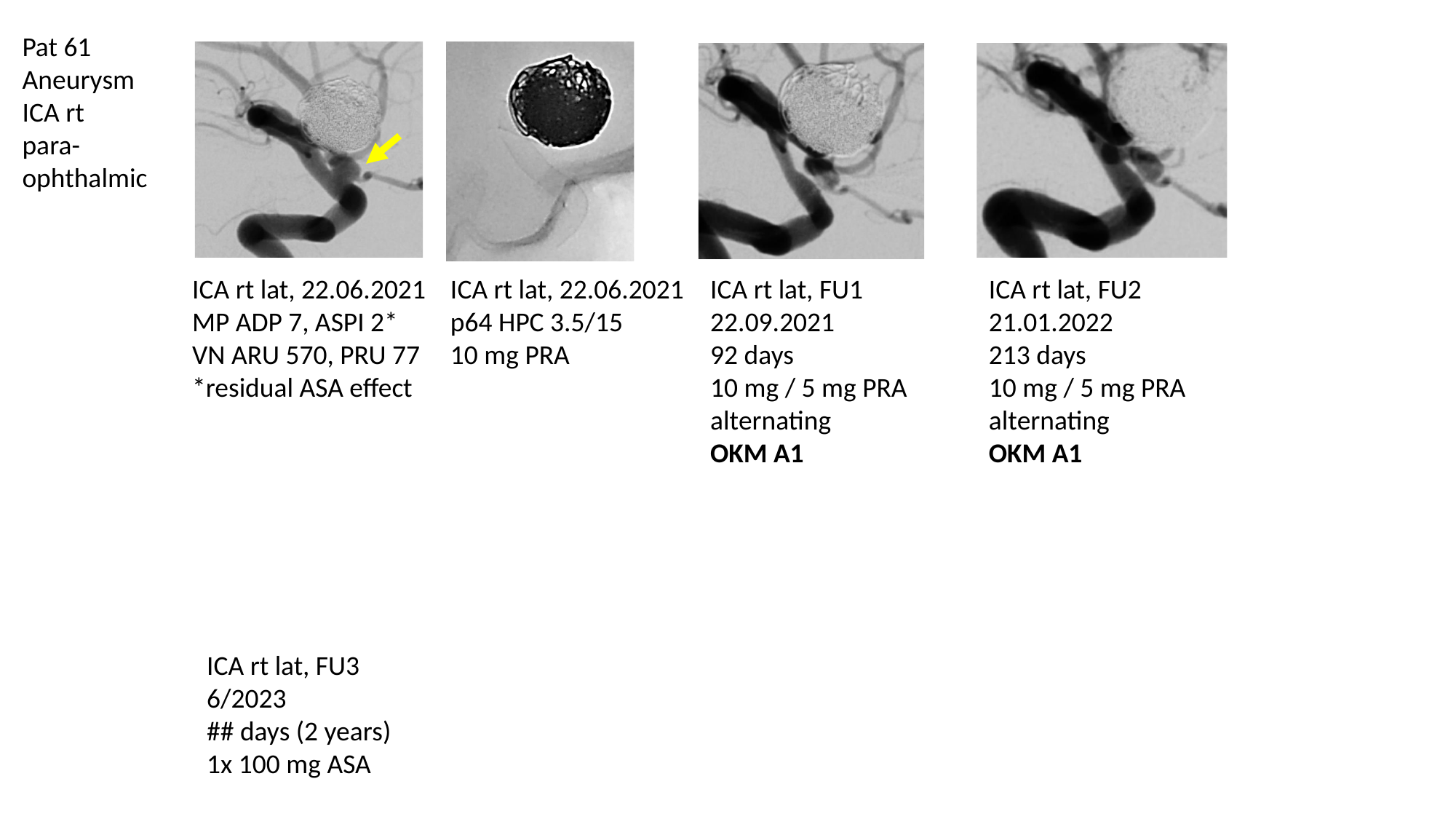

Pat 61
Aneurysm
ICA rt
para-
ophthalmic
ICA rt lat, 22.06.2021
p64 HPC 3.5/15
10 mg PRA
ICA rt lat, FU1
22.09.2021
92 days
10 mg / 5 mg PRA
alternating
OKM A1
ICA rt lat, FU2
21.01.2022
213 days
10 mg / 5 mg PRA
alternating
OKM A1
ICA rt lat, 22.06.2021
MP ADP 7, ASPI 2*
VN ARU 570, PRU 77
*residual ASA effect
ICA rt lat, FU3
6/2023
## days (2 years)
1x 100 mg ASA

## Slide 12
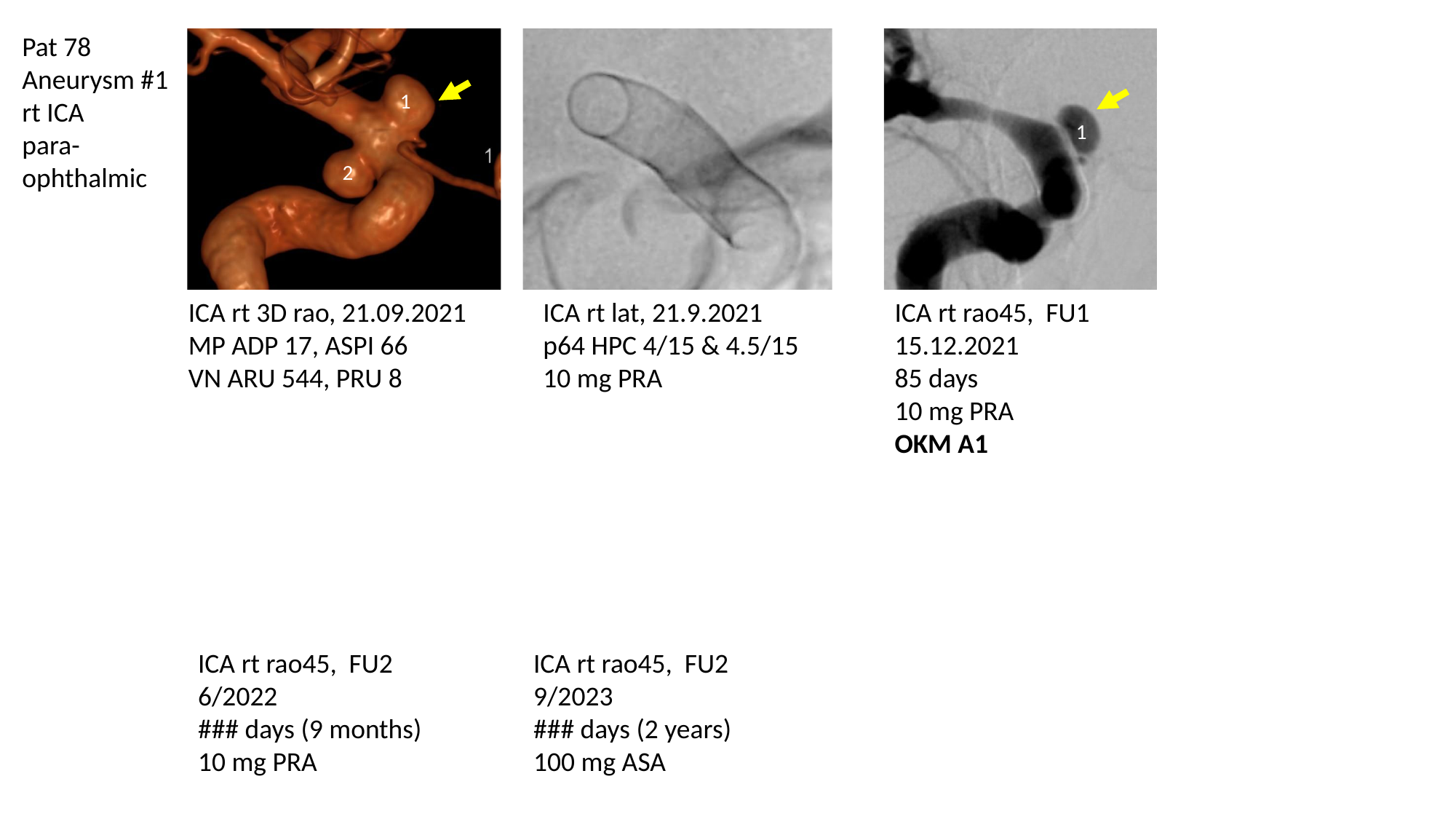

Pat 78
Aneurysm #1
rt ICA
para-
ophthalmic
1
1
2
2
1
ICA rt rao45, FU1
15.12.2021
85 days
10 mg PRA
OKM A1
ICA rt 3D rao, 21.09.2021
MP ADP 17, ASPI 66
VN ARU 544, PRU 8
ICA rt lat, 21.9.2021
p64 HPC 4/15 & 4.5/15
10 mg PRA
ICA rt rao45, FU2
9/2023
### days (2 years)
100 mg ASA
ICA rt rao45, FU2
6/2022
### days (9 months)
10 mg PRA

## Slide 13
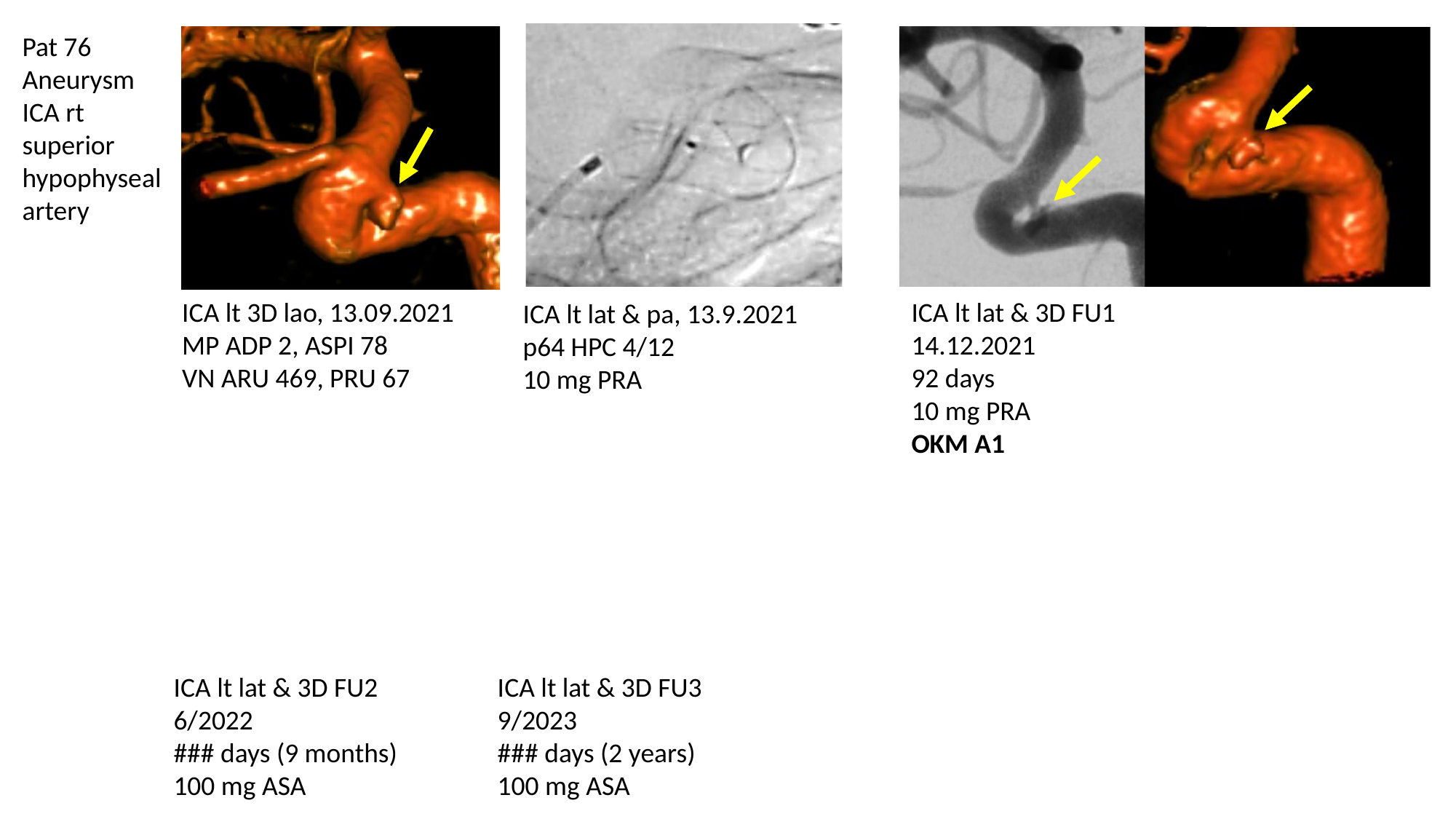

Pat 76
Aneurysm
ICA rt
superior
hypophyseal
artery
ICA lt lat & 3D FU1
14.12.2021
92 days
10 mg PRA
OKM A1
ICA lt 3D lao, 13.09.2021
MP ADP 2, ASPI 78
VN ARU 469, PRU 67
ICA lt lat & pa, 13.9.2021
p64 HPC 4/12
10 mg PRA
ICA lt lat & 3D FU3
9/2023
### days (2 years)
100 mg ASA
ICA lt lat & 3D FU2
6/2022
### days (9 months)
100 mg ASA

## Slide 14
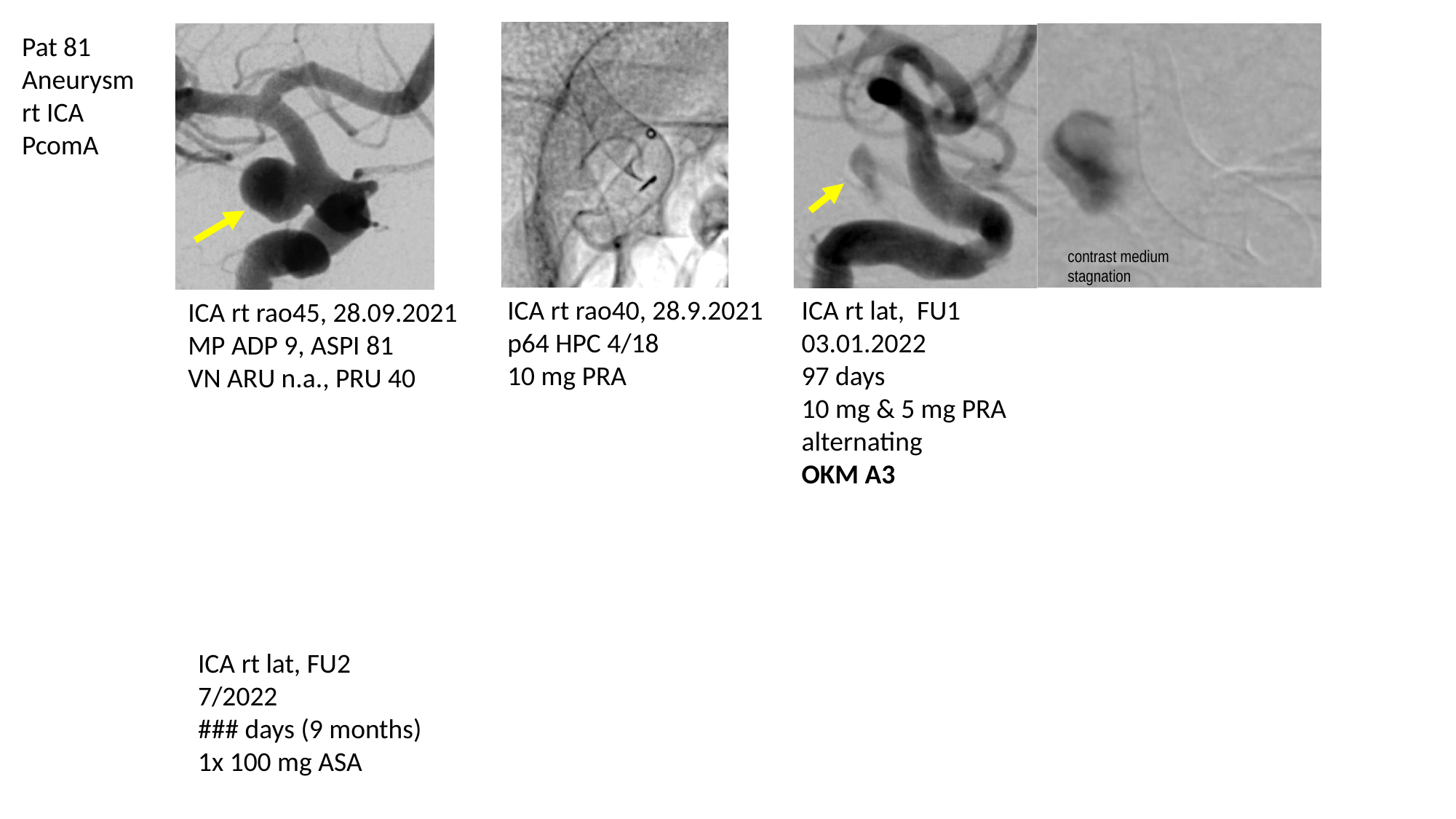

Pat 81
Aneurysm
rt ICA
PcomA
M2
M1
contrast medium
stagnation
ICA rt rao40, 28.9.2021
p64 HPC 4/18
10 mg PRA
ICA rt lat, FU1
03.01.2022
97 days
10 mg & 5 mg PRA
alternating
OKM A3
ICA rt rao45, 28.09.2021
MP ADP 9, ASPI 81
VN ARU n.a., PRU 40
ICA rt lat, FU2
7/2022
### days (9 months)
1x 100 mg ASA

## Slide 15
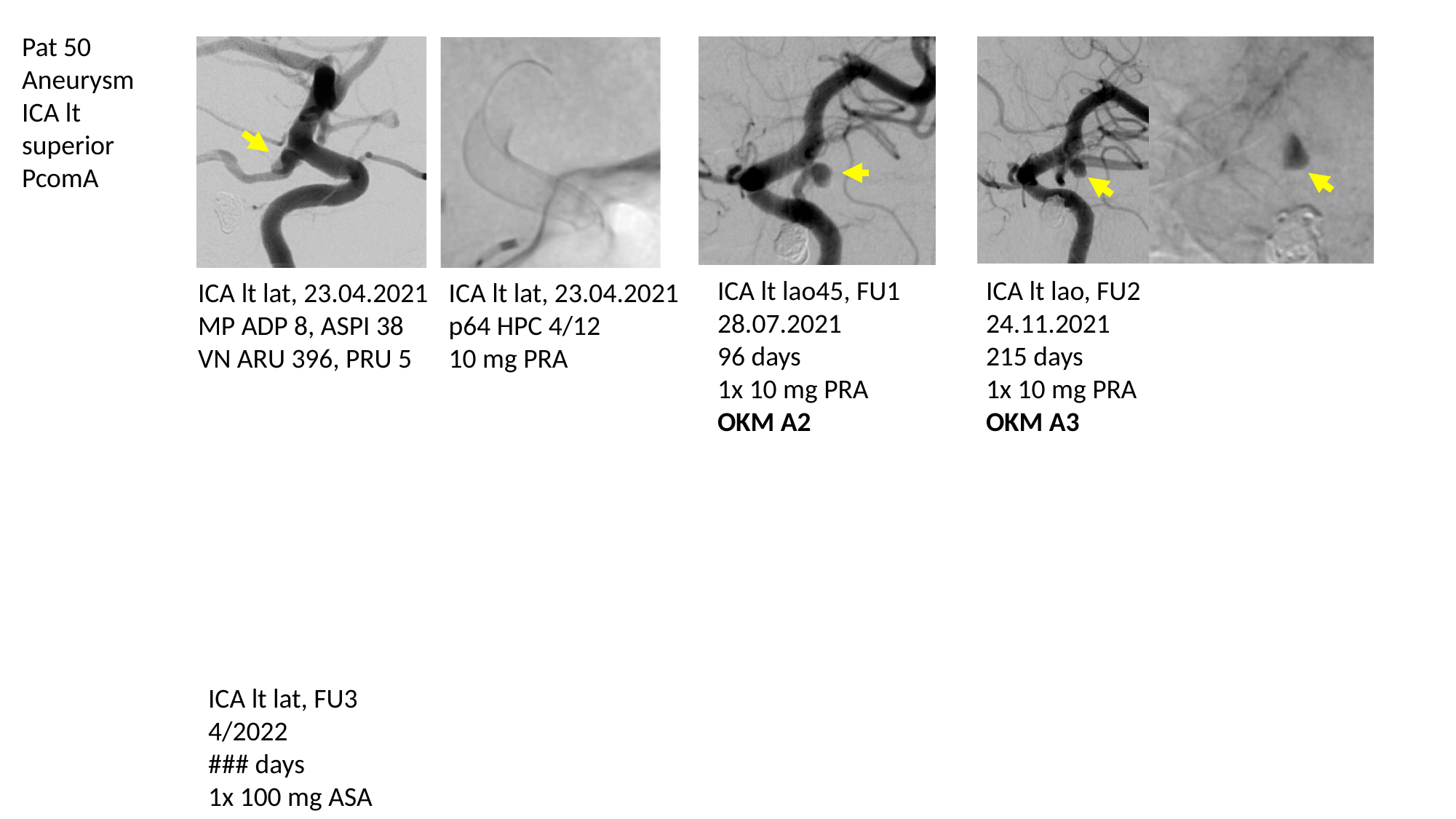

Pat 50
Aneurysm
ICA lt
superior
PcomA
ICA lt lao45, FU1
28.07.2021
96 days
1x 10 mg PRA
OKM A2
ICA lt lao, FU2
24.11.2021
215 days
1x 10 mg PRA
OKM A3
ICA lt lat, 23.04.2021
MP ADP 8, ASPI 38
VN ARU 396, PRU 5
ICA lt lat, 23.04.2021
p64 HPC 4/12
10 mg PRA
ICA lt lat, FU3
4/2022
### days
1x 100 mg ASA

## Slide 16
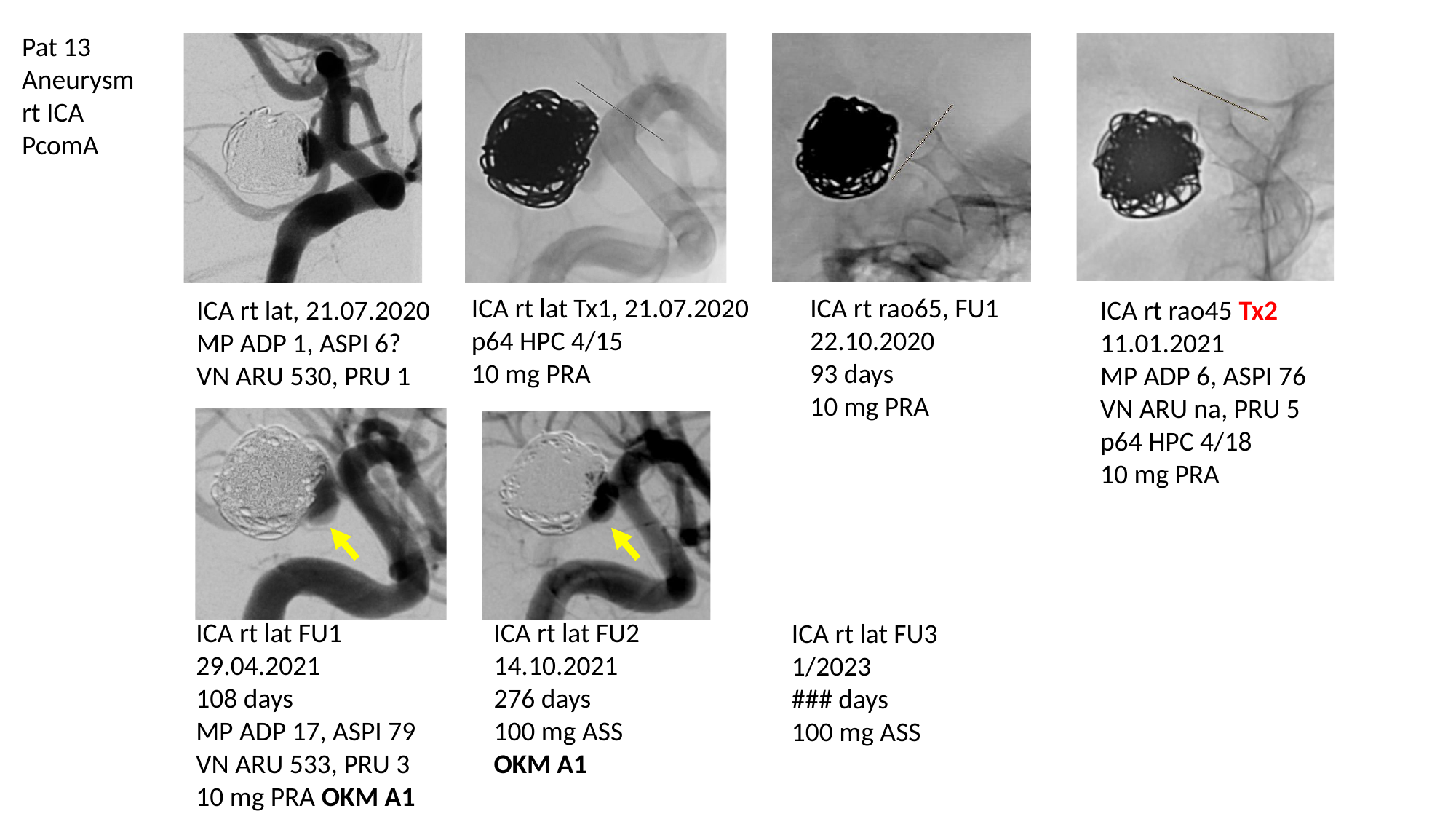

Pat 13
Aneurysm
rt ICA
PcomA
ICA rt lat Tx1, 21.07.2020
p64 HPC 4/15
10 mg PRA
ICA rt rao65, FU1
22.10.2020
93 days
10 mg PRA
ICA rt lat, 21.07.2020
MP ADP 1, ASPI 6?
VN ARU 530, PRU 1
ICA rt rao45 Tx2
11.01.2021
MP ADP 6, ASPI 76
VN ARU na, PRU 5
p64 HPC 4/18
10 mg PRA
ICA rt lat FU1
29.04.2021
108 days
MP ADP 17, ASPI 79
VN ARU 533, PRU 3
10 mg PRA OKM A1
ICA rt lat FU2
14.10.2021
276 days
100 mg ASS
OKM A1
ICA rt lat FU3
1/2023
### days
100 mg ASS

## Slide 17
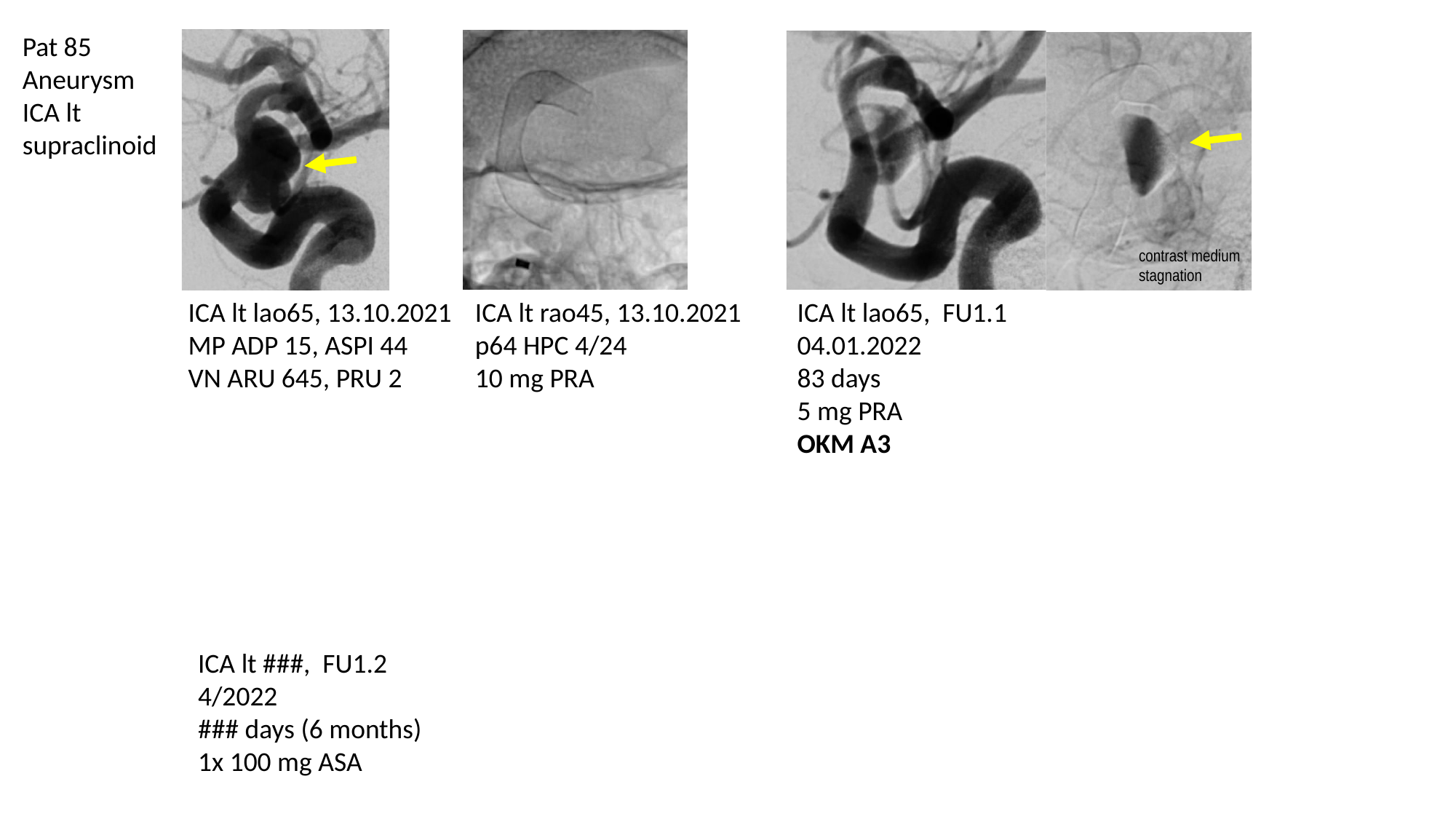

Pat 85
Aneurysm
ICA lt
supraclinoid
M2
M1
contrast medium
stagnation
ICA lt lao65, 13.10.2021
MP ADP 15, ASPI 44
VN ARU 645, PRU 2
ICA lt rao45, 13.10.2021
p64 HPC 4/24
10 mg PRA
ICA lt lao65, FU1.1
04.01.2022
83 days
5 mg PRA
OKM A3
ICA lt ###, FU1.2
4/2022
### days (6 months)
1x 100 mg ASA

## Slide 18
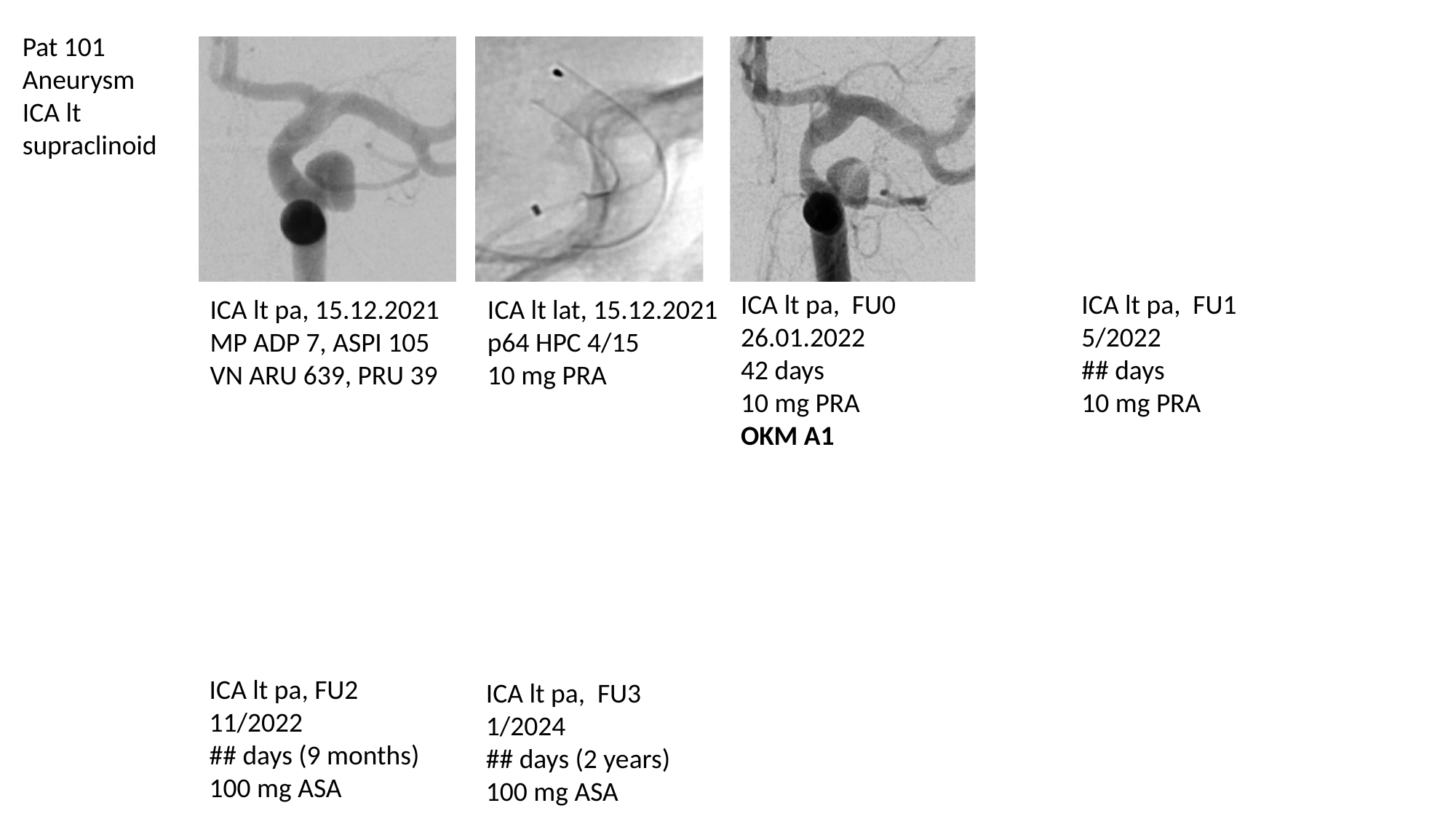

Pat 101
Aneurysm
ICA lt
supraclinoid
ICA lt pa, FU0
26.01.2022
42 days
10 mg PRA
OKM A1
ICA lt pa, FU1
5/2022
## days
10 mg PRA
ICA lt pa, 15.12.2021
MP ADP 7, ASPI 105
VN ARU 639, PRU 39
ICA lt lat, 15.12.2021
p64 HPC 4/15
10 mg PRA
ICA lt pa, FU2
11/2022
## days (9 months)
100 mg ASA
ICA lt pa, FU3
1/2024
## days (2 years)
100 mg ASA

## Slide 19
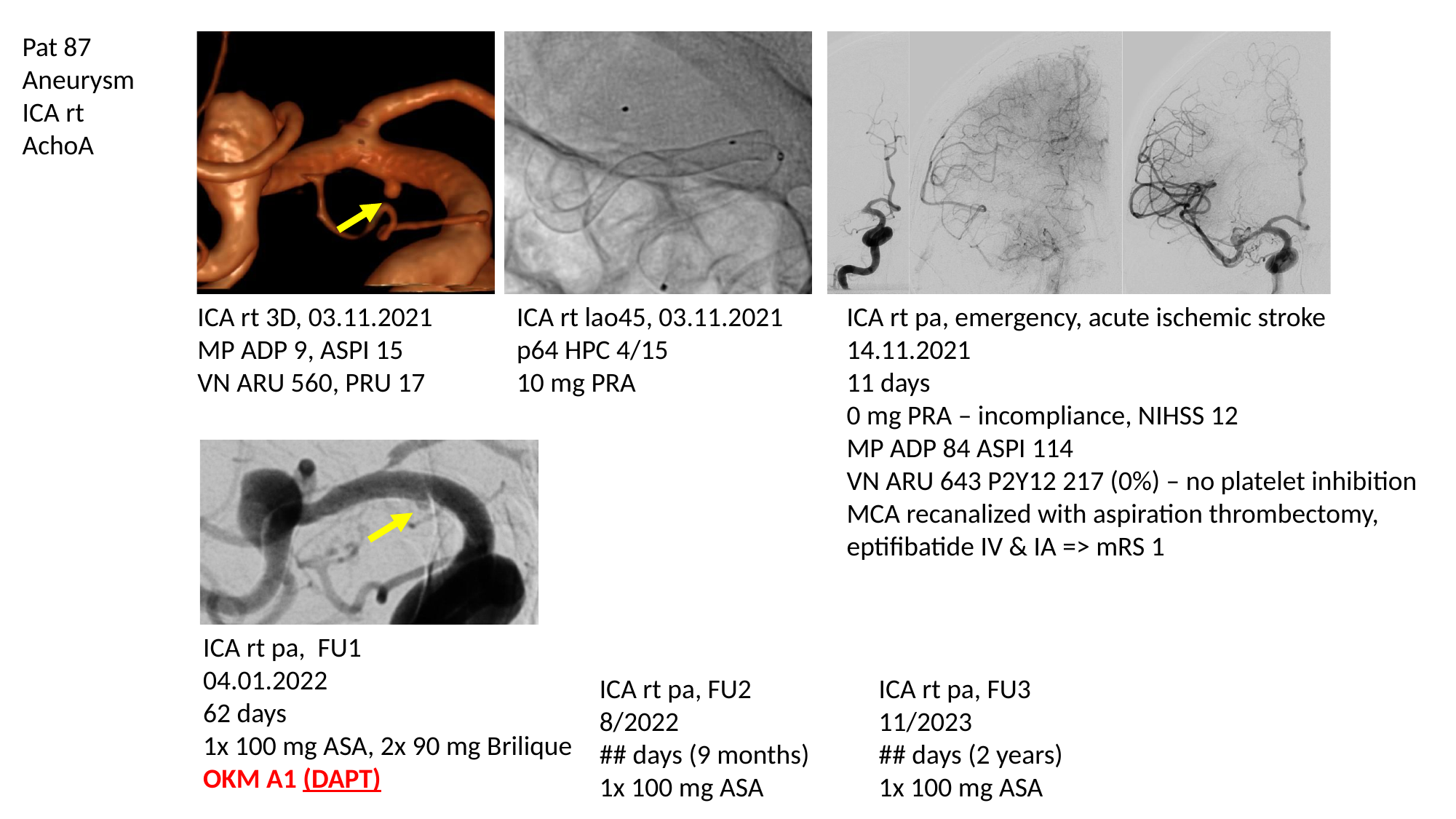

Pat 87
Aneurysm
ICA rt
AchoA
M2
M1
ICA rt lao45, 03.11.2021
p64 HPC 4/15
10 mg PRA
ICA rt pa, emergency, acute ischemic stroke
14.11.2021
11 days
0 mg PRA – incompliance, NIHSS 12
MP ADP 84 ASPI 114
VN ARU 643 P2Y12 217 (0%) – no platelet inhibition
MCA recanalized with aspiration thrombectomy, eptifibatide IV & IA => mRS 1
ICA rt 3D, 03.11.2021
MP ADP 9, ASPI 15
VN ARU 560, PRU 17
ICA rt pa, FU1
04.01.2022
62 days
1x 100 mg ASA, 2x 90 mg Brilique
OKM A1 (DAPT)
ICA rt pa, FU2
8/2022
## days (9 months)
1x 100 mg ASA
ICA rt pa, FU3
11/2023
## days (2 years)
1x 100 mg ASA

## Slide 20
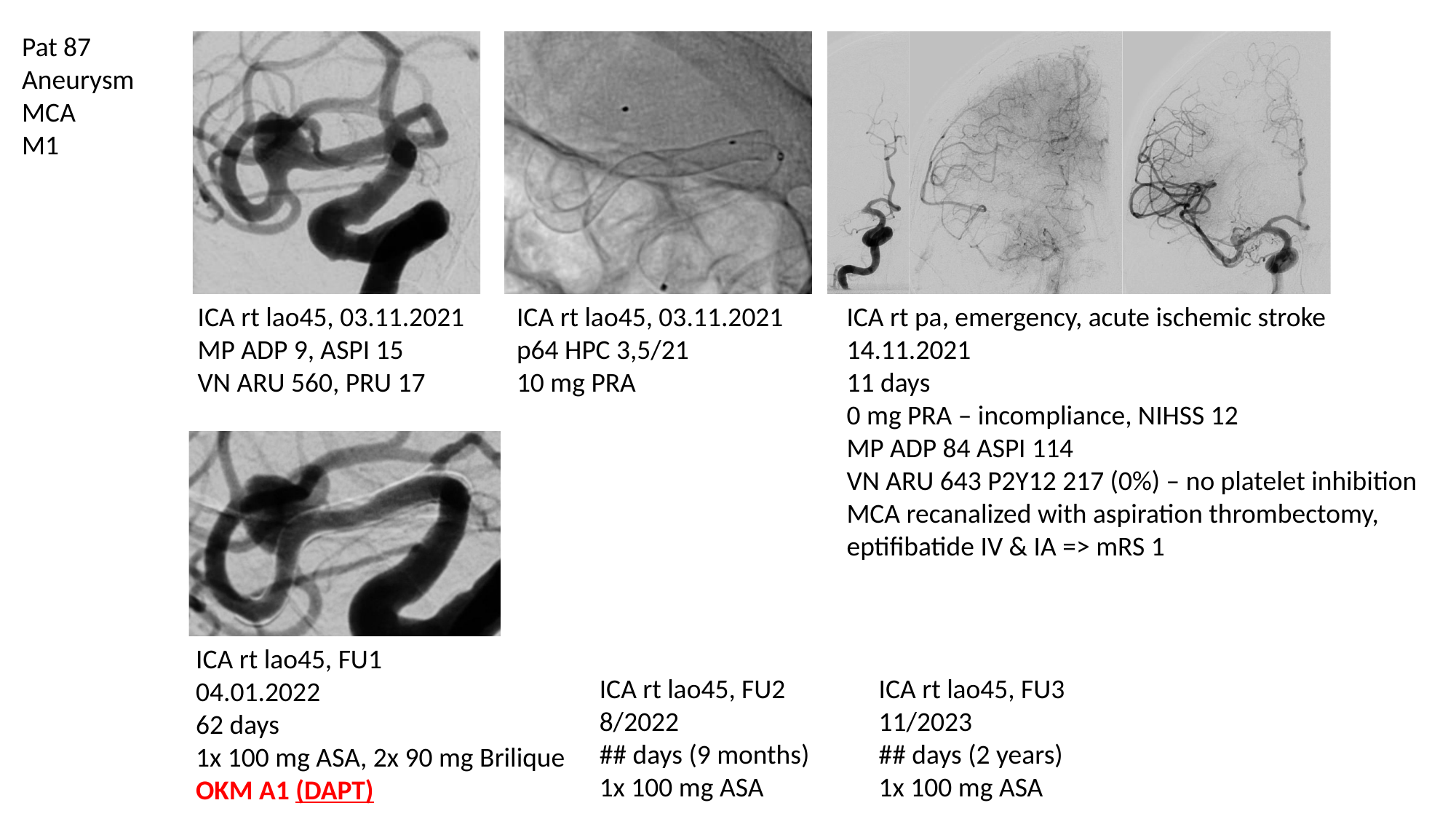

Pat 87
Aneurysm
MCA
M1
M2
M1
ICA rt lao45, 03.11.2021
p64 HPC 3,5/21
10 mg PRA
ICA rt pa, emergency, acute ischemic stroke
14.11.2021
11 days
0 mg PRA – incompliance, NIHSS 12
MP ADP 84 ASPI 114
VN ARU 643 P2Y12 217 (0%) – no platelet inhibition
MCA recanalized with aspiration thrombectomy, eptifibatide IV & IA => mRS 1
ICA rt lao45, 03.11.2021
MP ADP 9, ASPI 15
VN ARU 560, PRU 17
ICA rt lao45, FU1
04.01.2022
62 days
1x 100 mg ASA, 2x 90 mg Brilique
OKM A1 (DAPT)
ICA rt lao45, FU2
8/2022
## days (9 months)
1x 100 mg ASA
ICA rt lao45, FU3
11/2023
## days (2 years)
1x 100 mg ASA

## Slide 21
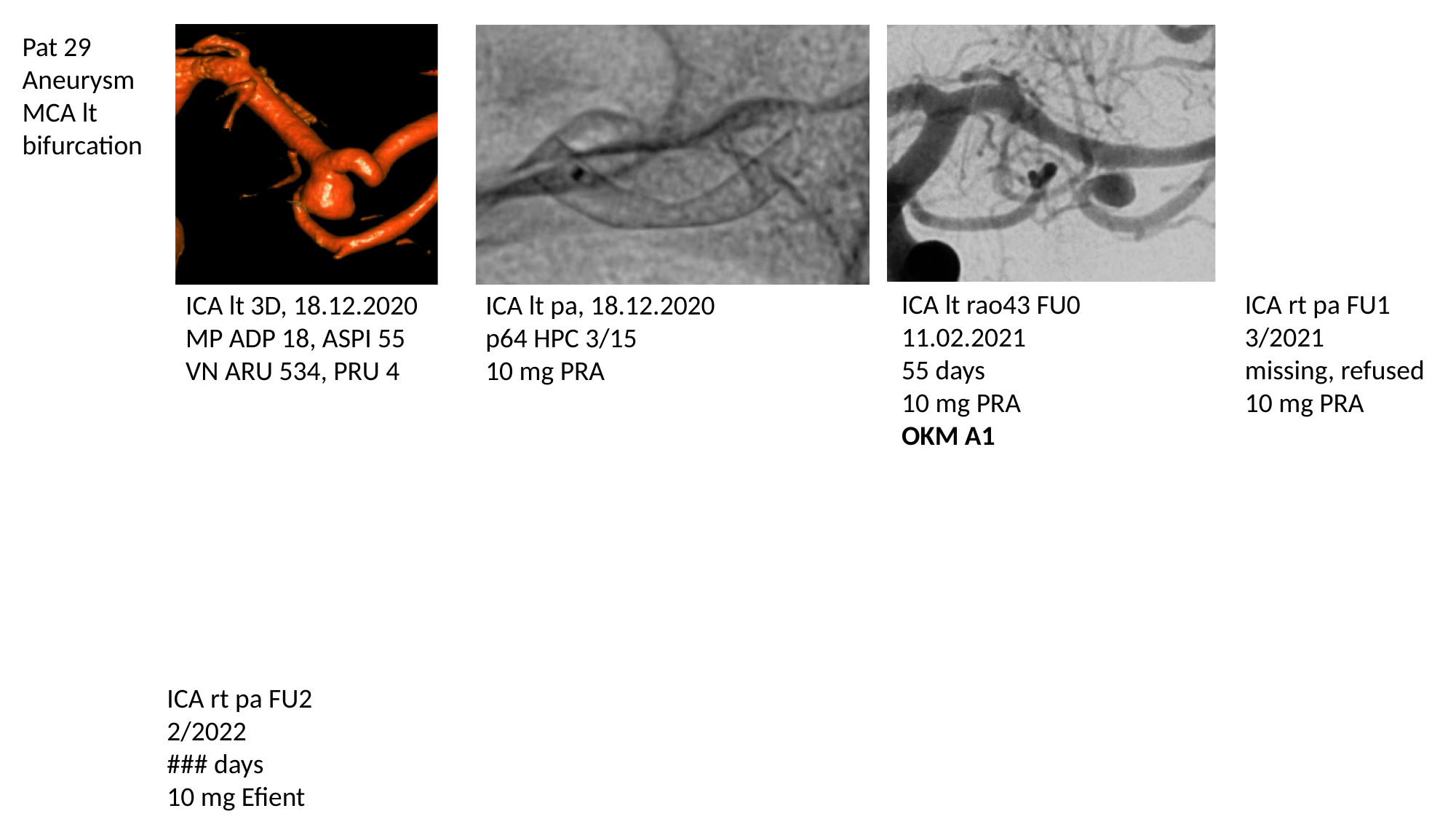

Pat 29
Aneurysm
MCA lt
bifurcation
ICA rt pa FU1
3/2021
missing, refused
10 mg PRA
ICA lt rao43 FU0
11.02.2021
55 days
10 mg PRA
OKM A1
ICA lt 3D, 18.12.2020
MP ADP 18, ASPI 55
VN ARU 534, PRU 4
ICA lt pa, 18.12.2020
p64 HPC 3/15
10 mg PRA
ICA rt pa FU2
2/2022
### days
10 mg Efient

## Slide 22
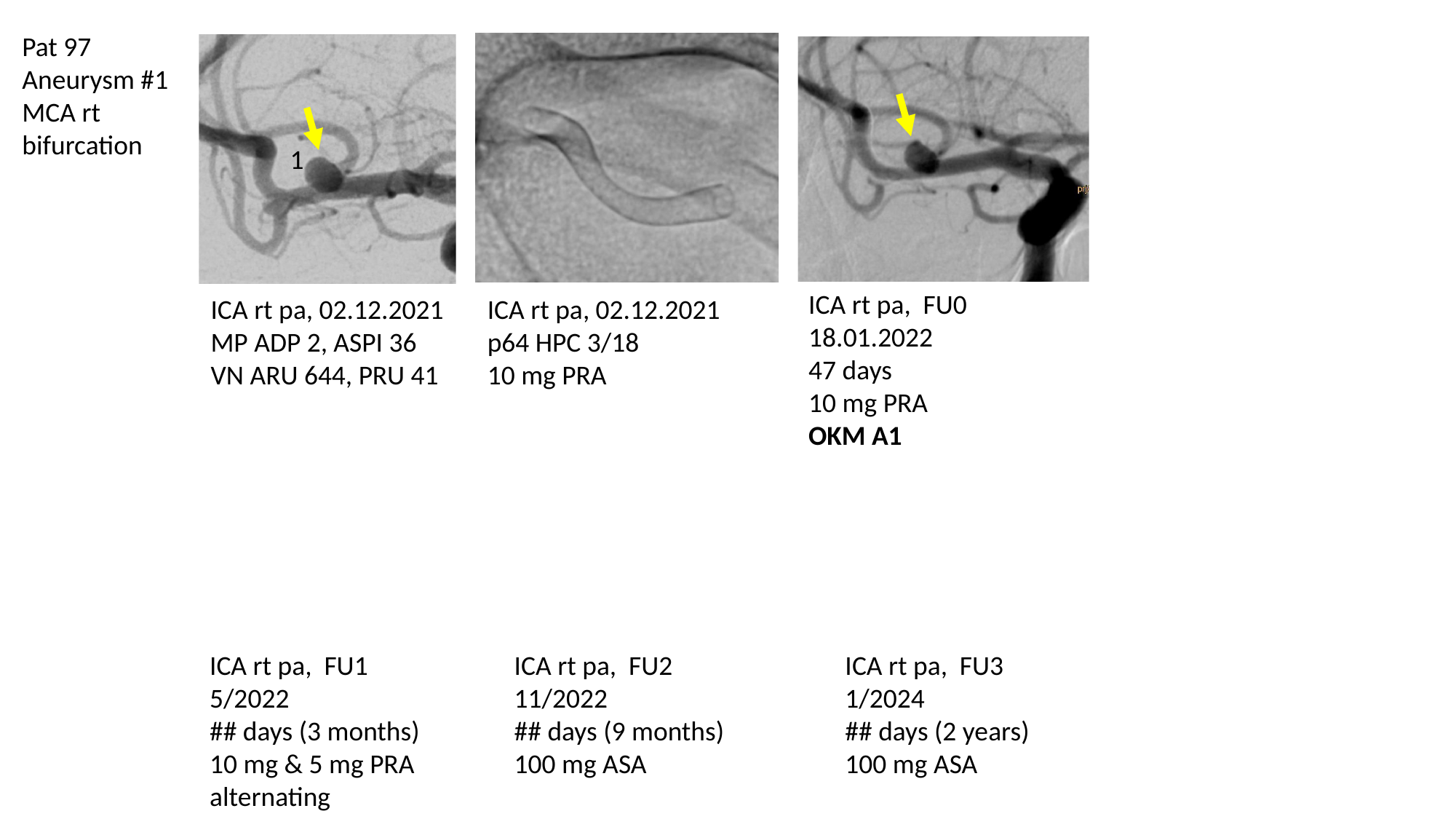

Pat 97
Aneurysm #1
MCA rt
bifurcation
1
ICA rt pa, FU0
18.01.2022
47 days
10 mg PRA
OKM A1
ICA rt pa, 02.12.2021
MP ADP 2, ASPI 36
VN ARU 644, PRU 41
ICA rt pa, 02.12.2021
p64 HPC 3/18
10 mg PRA
ICA rt pa, FU3
1/2024
## days (2 years)
100 mg ASA
ICA rt pa, FU1
5/2022
## days (3 months)
10 mg & 5 mg PRA
alternating
ICA rt pa, FU2
11/2022
## days (9 months)
100 mg ASA

## Slide 23
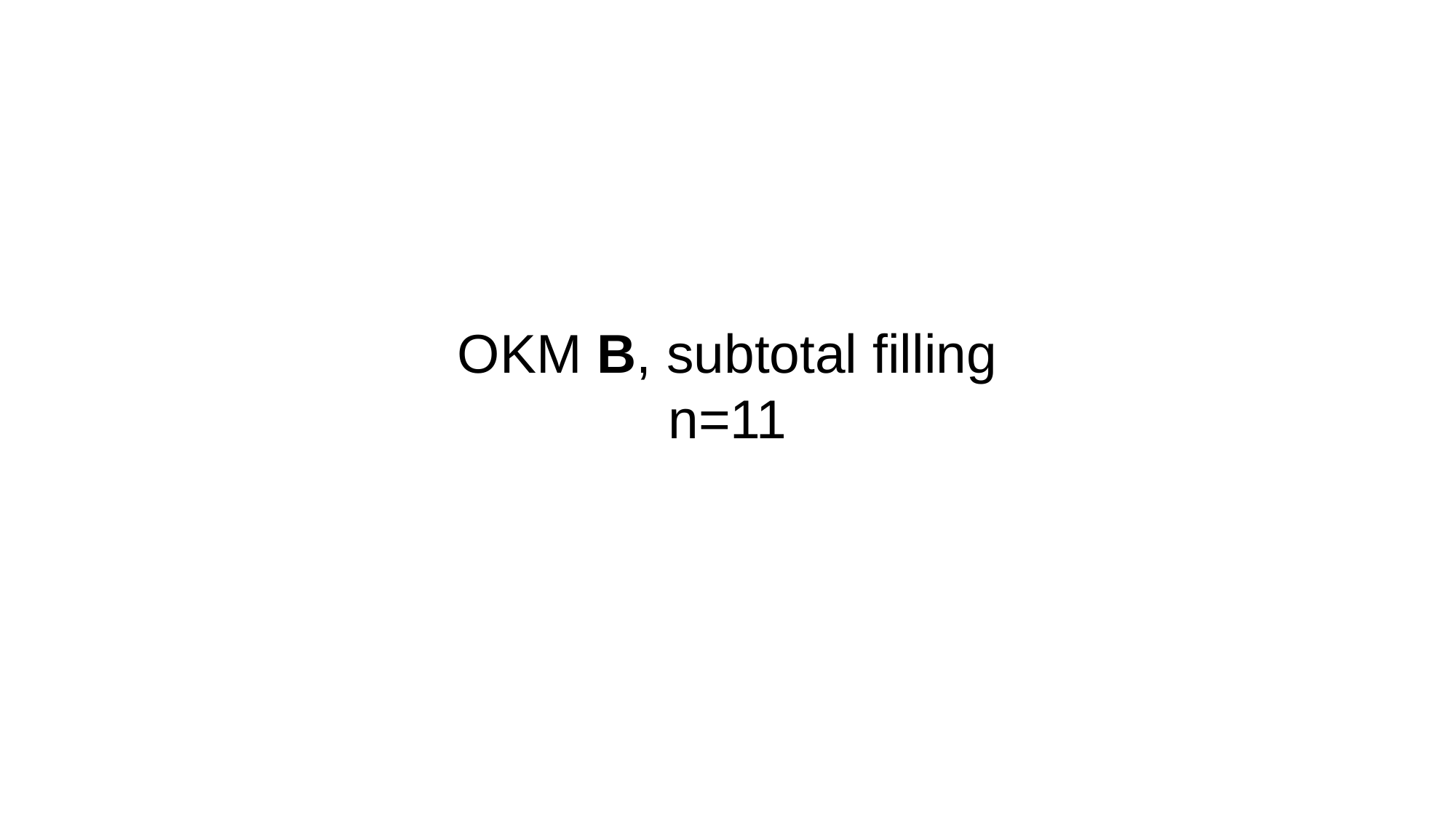

OKM B, subtotal filling
n=11

## Slide 24
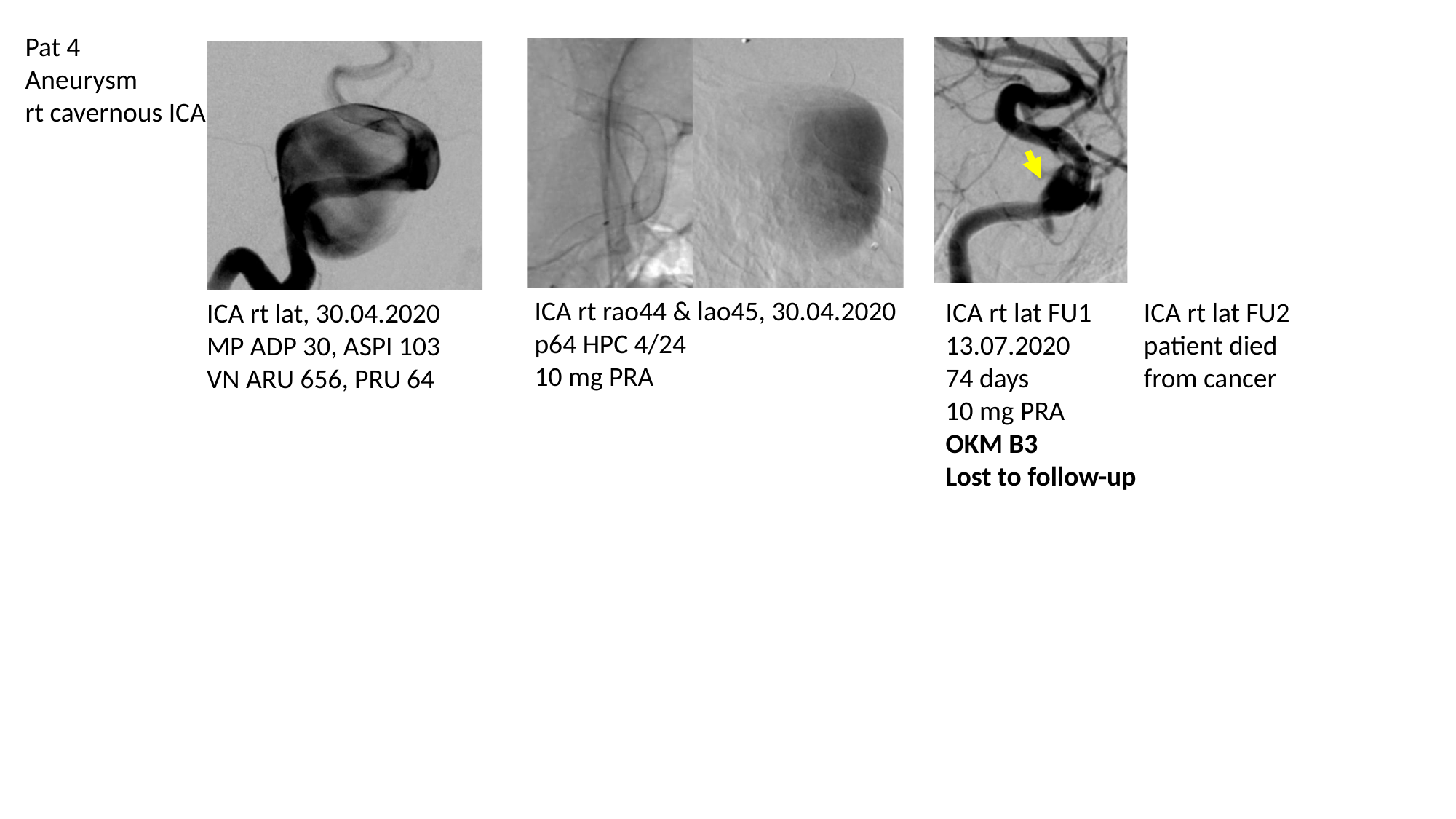

Pat 4
Aneurysm
rt cavernous ICA
ICA rt rao44 & lao45, 30.04.2020
p64 HPC 4/24
10 mg PRA
ICA rt lat FU1
13.07.2020
74 days
10 mg PRA
OKM B3
Lost to follow-up
ICA rt lat FU2
patient died
from cancer
ICA rt lat, 30.04.2020
MP ADP 30, ASPI 103
VN ARU 656, PRU 64

## Slide 25
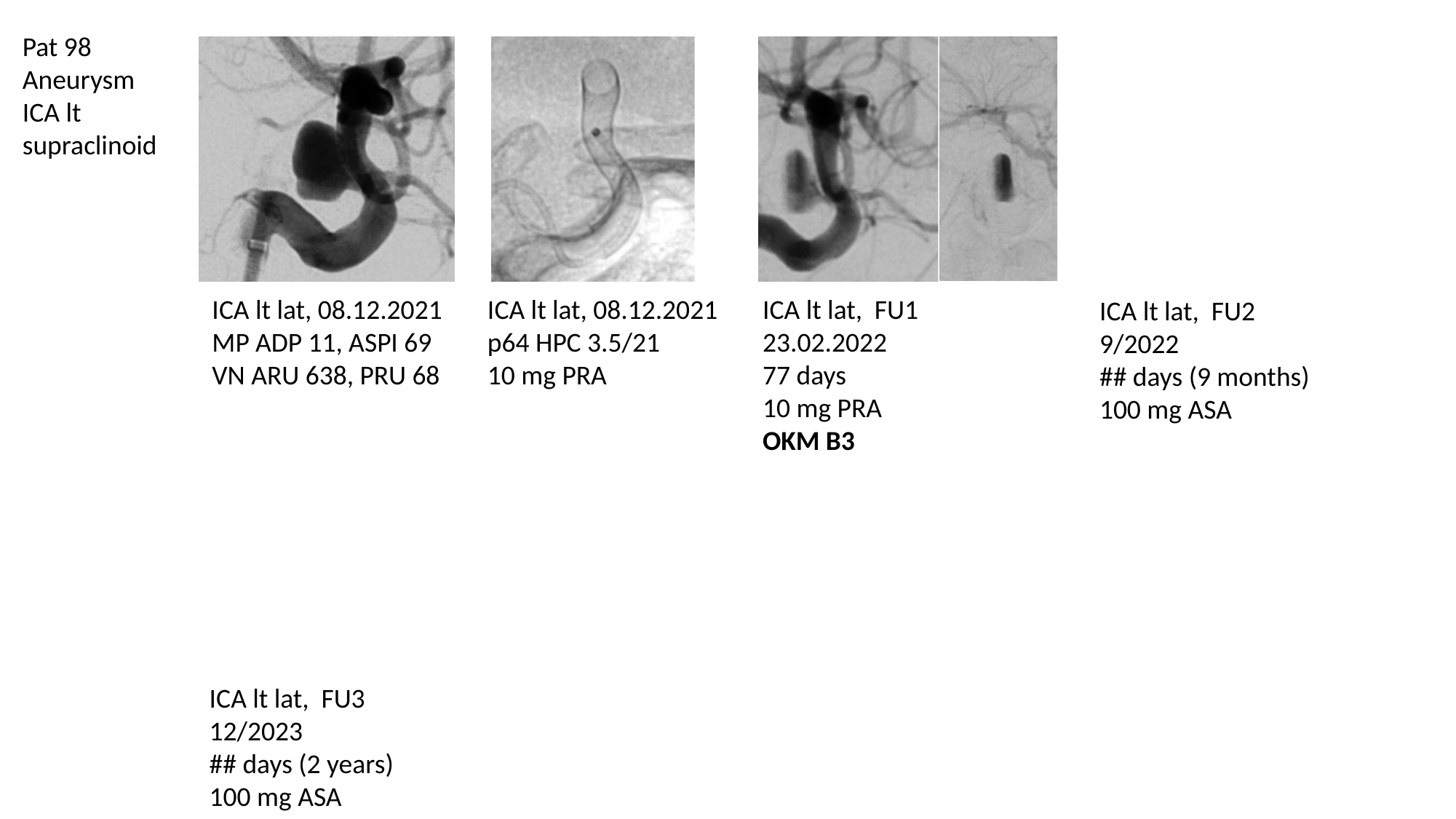

Pat 98
Aneurysm
ICA lt
supraclinoid
ICA lt lat, 08.12.2021
MP ADP 11, ASPI 69
VN ARU 638, PRU 68
ICA lt lat, 08.12.2021
p64 HPC 3.5/21
10 mg PRA
ICA lt lat, FU1
23.02.2022
77 days
10 mg PRA
OKM B3
ICA lt lat, FU2
9/2022
## days (9 months)
100 mg ASA
ICA lt lat, FU3
12/2023
## days (2 years)
100 mg ASA

## Slide 26
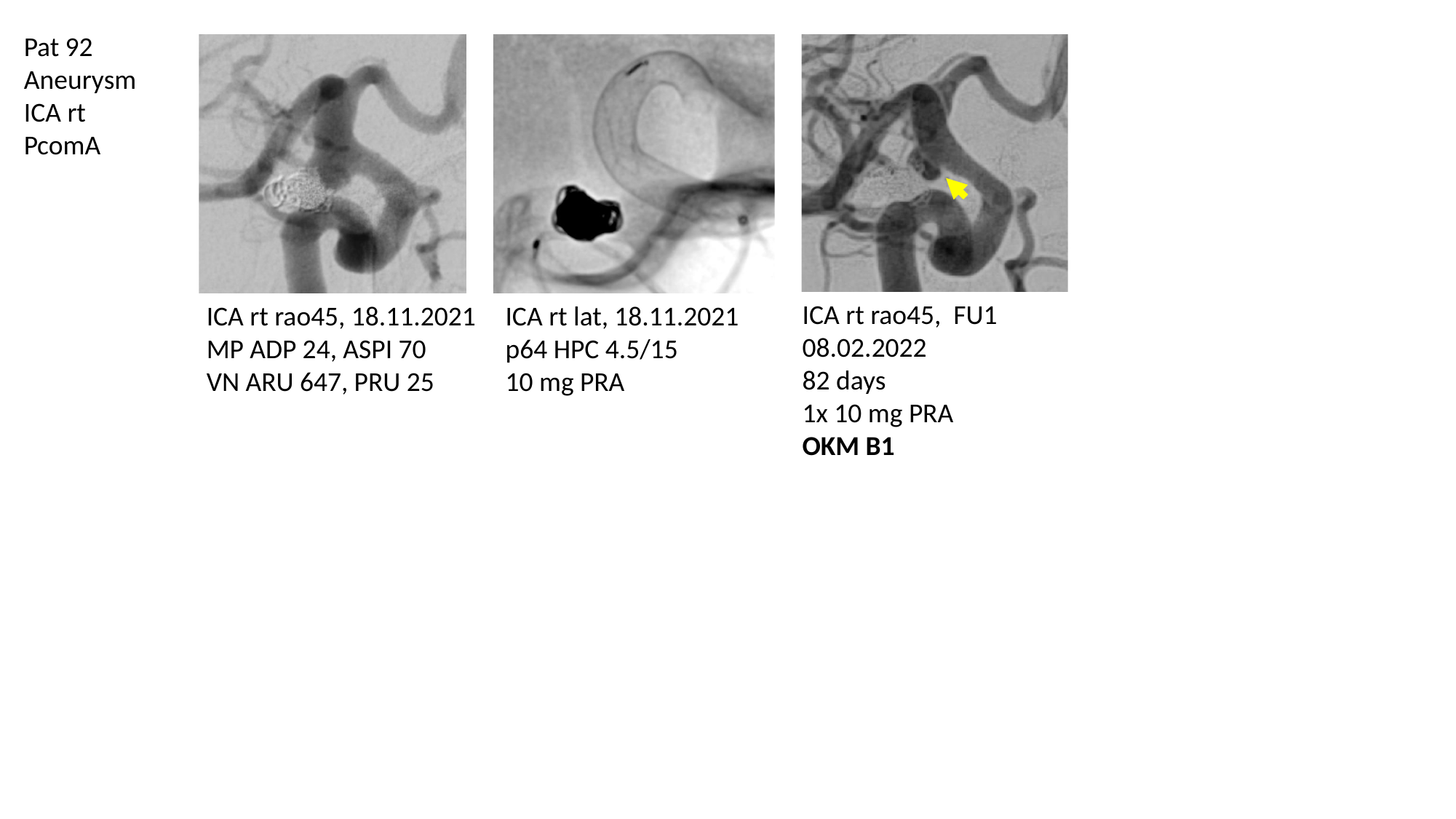

Pat 92
Aneurysm
ICA rt
PcomA
ICA rt rao45, FU1
08.02.2022
82 days
1x 10 mg PRA
OKM B1
ICA rt lat, 18.11.2021
p64 HPC 4.5/15
10 mg PRA
ICA rt rao45, 18.11.2021
MP ADP 24, ASPI 70
VN ARU 647, PRU 25

## Slide 27
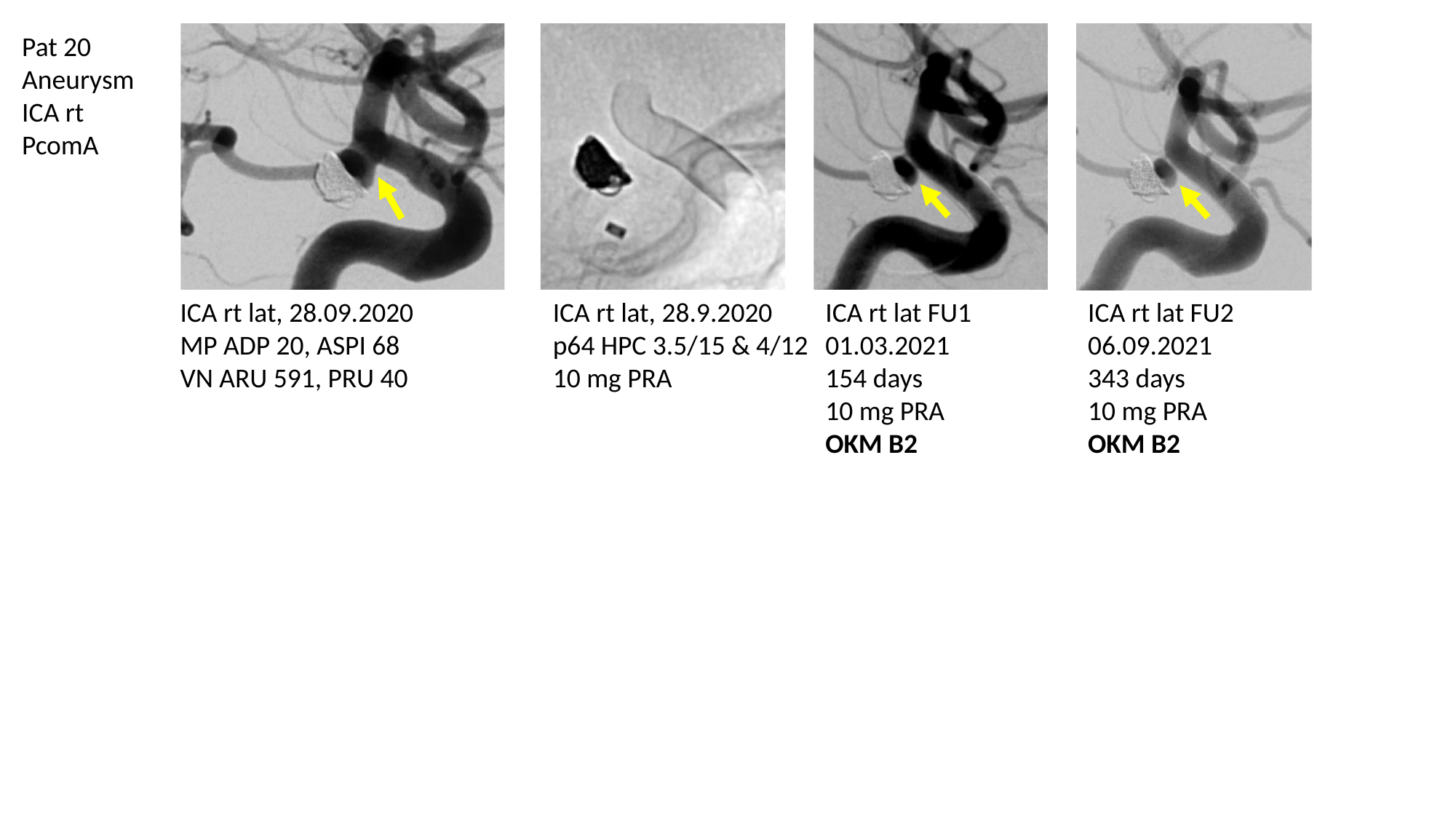

Pat 20
Aneurysm
ICA rt
PcomA
ICA rt lat FU2
06.09.2021
343 days
10 mg PRA
OKM B2
ICA rt lat, 28.09.2020
MP ADP 20, ASPI 68
VN ARU 591, PRU 40
ICA rt lat, 28.9.2020
p64 HPC 3.5/15 & 4/12
10 mg PRA
ICA rt lat FU1
01.03.2021
154 days
10 mg PRA
OKM B2

## Slide 28
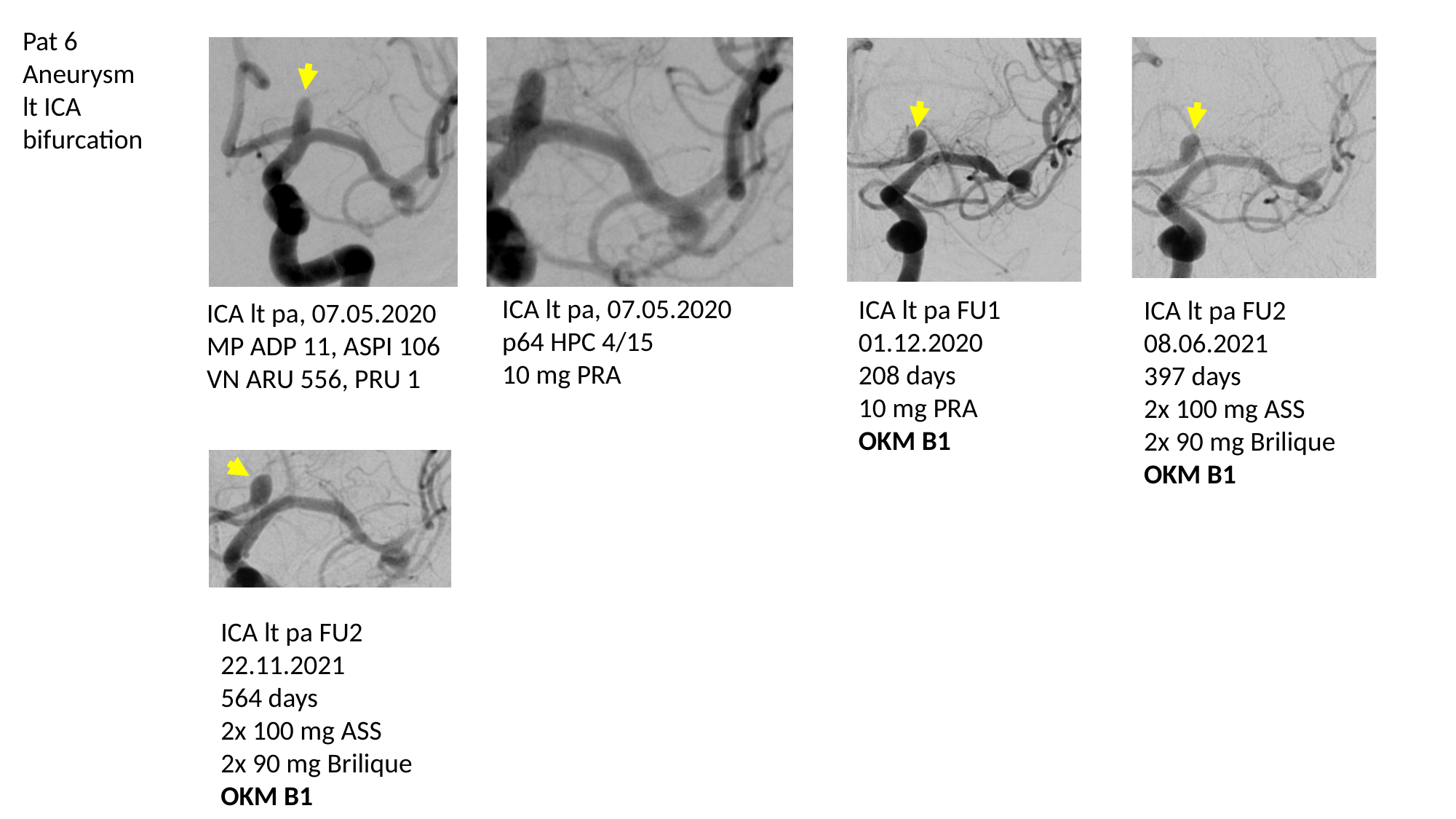

Pat 6
Aneurysm
lt ICA
bifurcation
ICA lt pa, 07.05.2020
p64 HPC 4/15
10 mg PRA
ICA lt pa FU1
01.12.2020
208 days
10 mg PRA
OKM B1
ICA lt pa FU2
08.06.2021
397 days
2x 100 mg ASS
2x 90 mg Brilique
OKM B1
ICA lt pa, 07.05.2020
MP ADP 11, ASPI 106
VN ARU 556, PRU 1
ICA lt pa FU2
22.11.2021
564 days
2x 100 mg ASS
2x 90 mg Brilique
OKM B1

## Slide 29
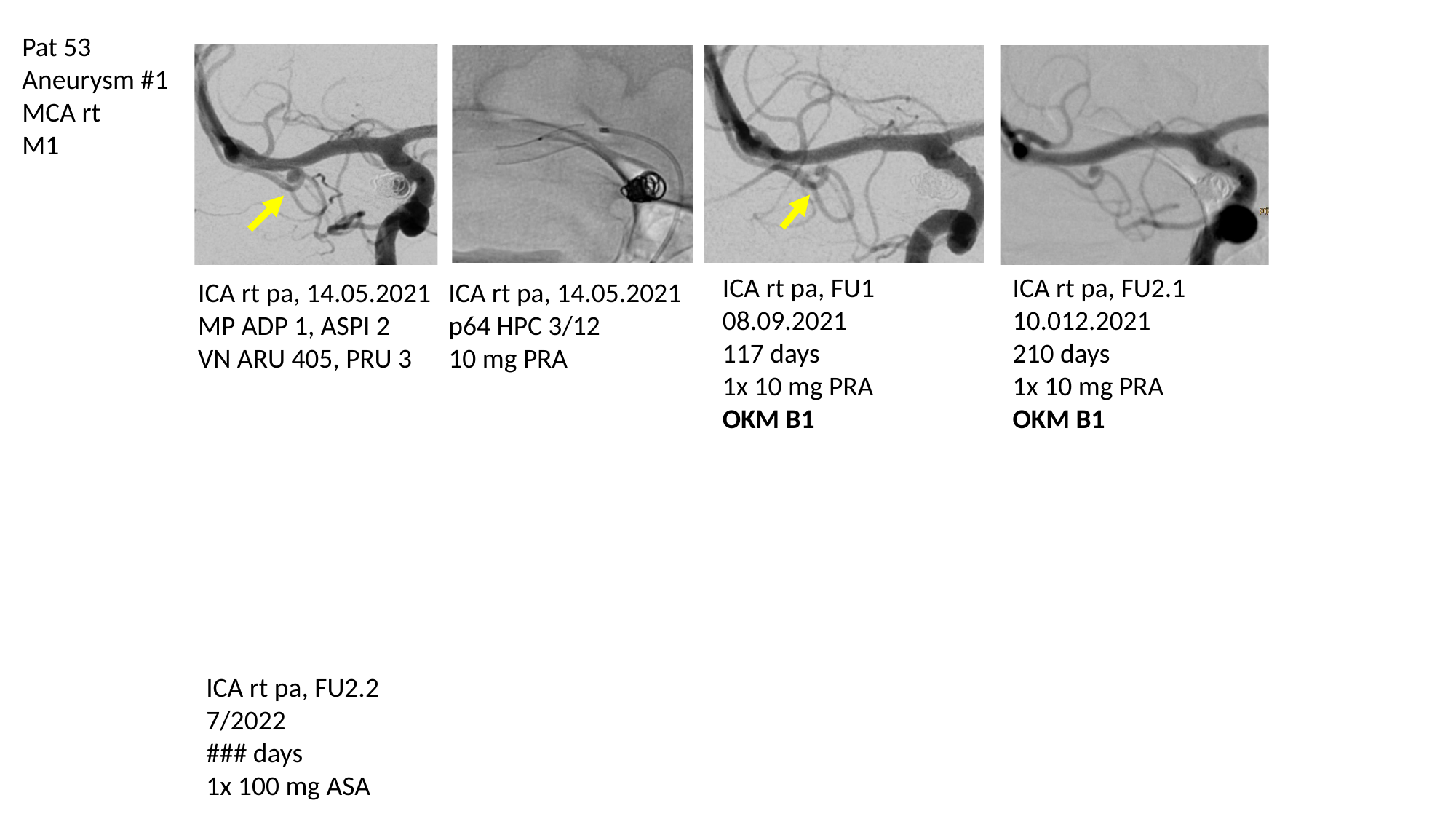

Pat 53
Aneurysm #1
MCA rt
M1
ICA rt pa, FU1
08.09.2021
117 days
1x 10 mg PRA
OKM B1
ICA rt pa, FU2.1
10.012.2021
210 days
1x 10 mg PRA
OKM B1
ICA rt pa, 14.05.2021
MP ADP 1, ASPI 2
VN ARU 405, PRU 3
ICA rt pa, 14.05.2021
p64 HPC 3/12
10 mg PRA
ICA rt pa, FU2.2
7/2022
### days
1x 100 mg ASA

## Slide 30
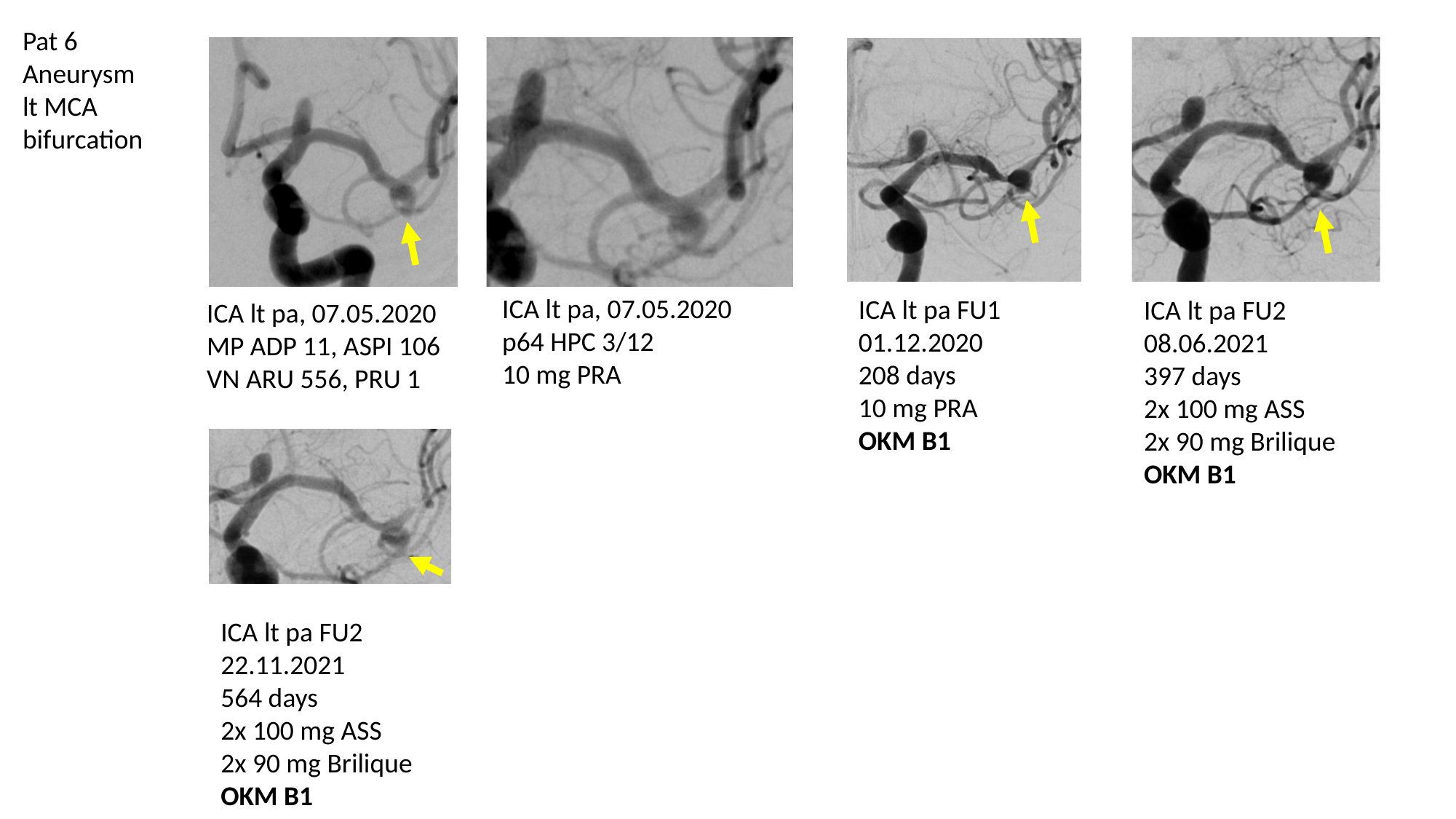

Pat 6
Aneurysm
lt MCA
bifurcation
ICA lt pa, 07.05.2020
p64 HPC 3/12
10 mg PRA
ICA lt pa FU1
01.12.2020
208 days
10 mg PRA
OKM B1
ICA lt pa FU2
08.06.2021
397 days
2x 100 mg ASS
2x 90 mg Brilique
OKM B1
ICA lt pa, 07.05.2020
MP ADP 11, ASPI 106
VN ARU 556, PRU 1
ICA lt pa FU2
22.11.2021
564 days
2x 100 mg ASS
2x 90 mg Brilique
OKM B1

## Slide 31
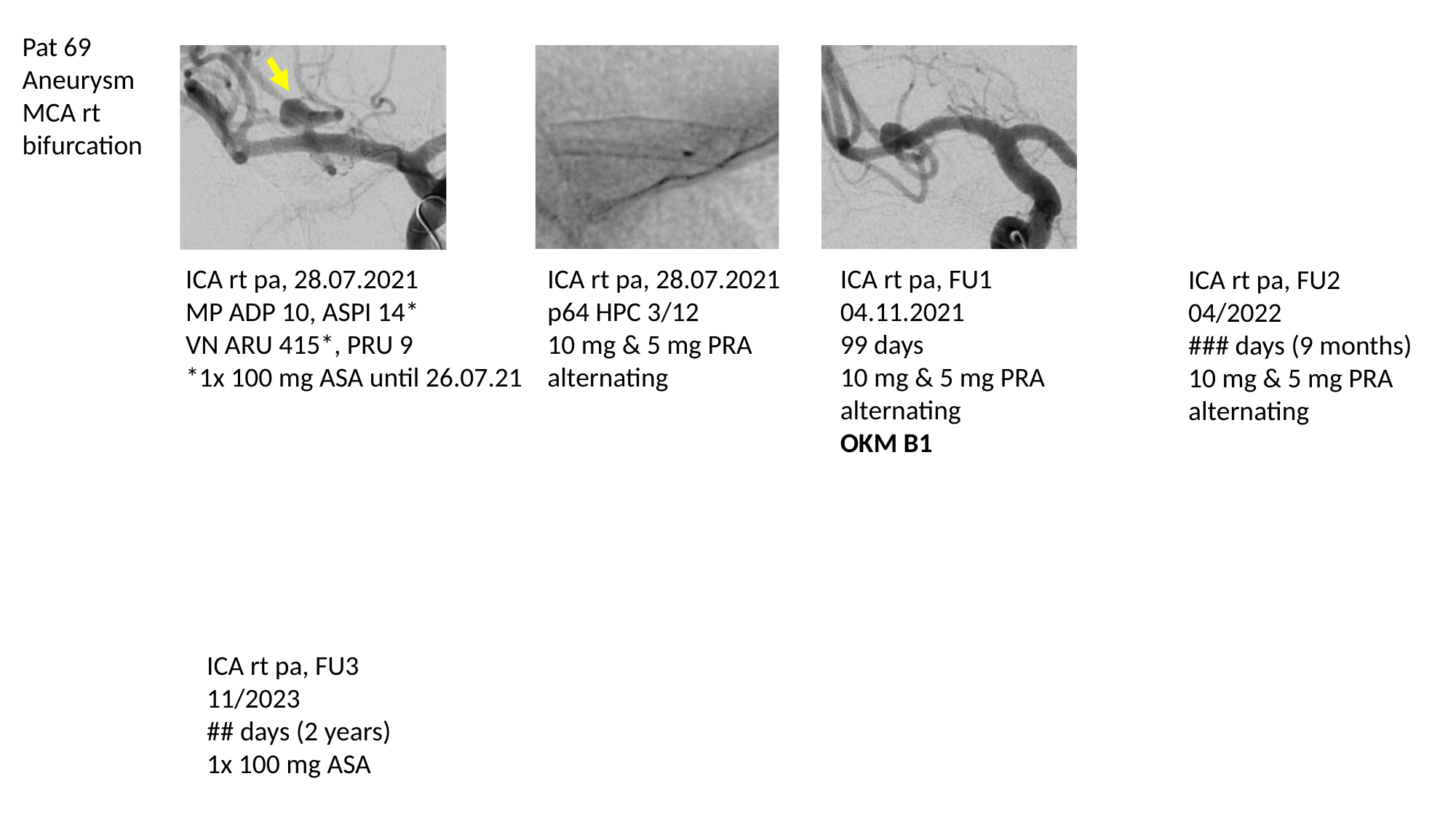

Pat 69
Aneurysm
MCA rt
bifurcation
ICA rt pa, FU1
04.11.2021
99 days
10 mg & 5 mg PRA
alternating
OKM B1
ICA rt pa, 28.07.2021
MP ADP 10, ASPI 14*
VN ARU 415*, PRU 9
*1x 100 mg ASA until 26.07.21
ICA rt pa, 28.07.2021
p64 HPC 3/12
10 mg & 5 mg PRA
alternating
ICA rt pa, FU2
04/2022
### days (9 months)
10 mg & 5 mg PRA
alternating
ICA rt pa, FU3
11/2023
## days (2 years)
1x 100 mg ASA

## Slide 32
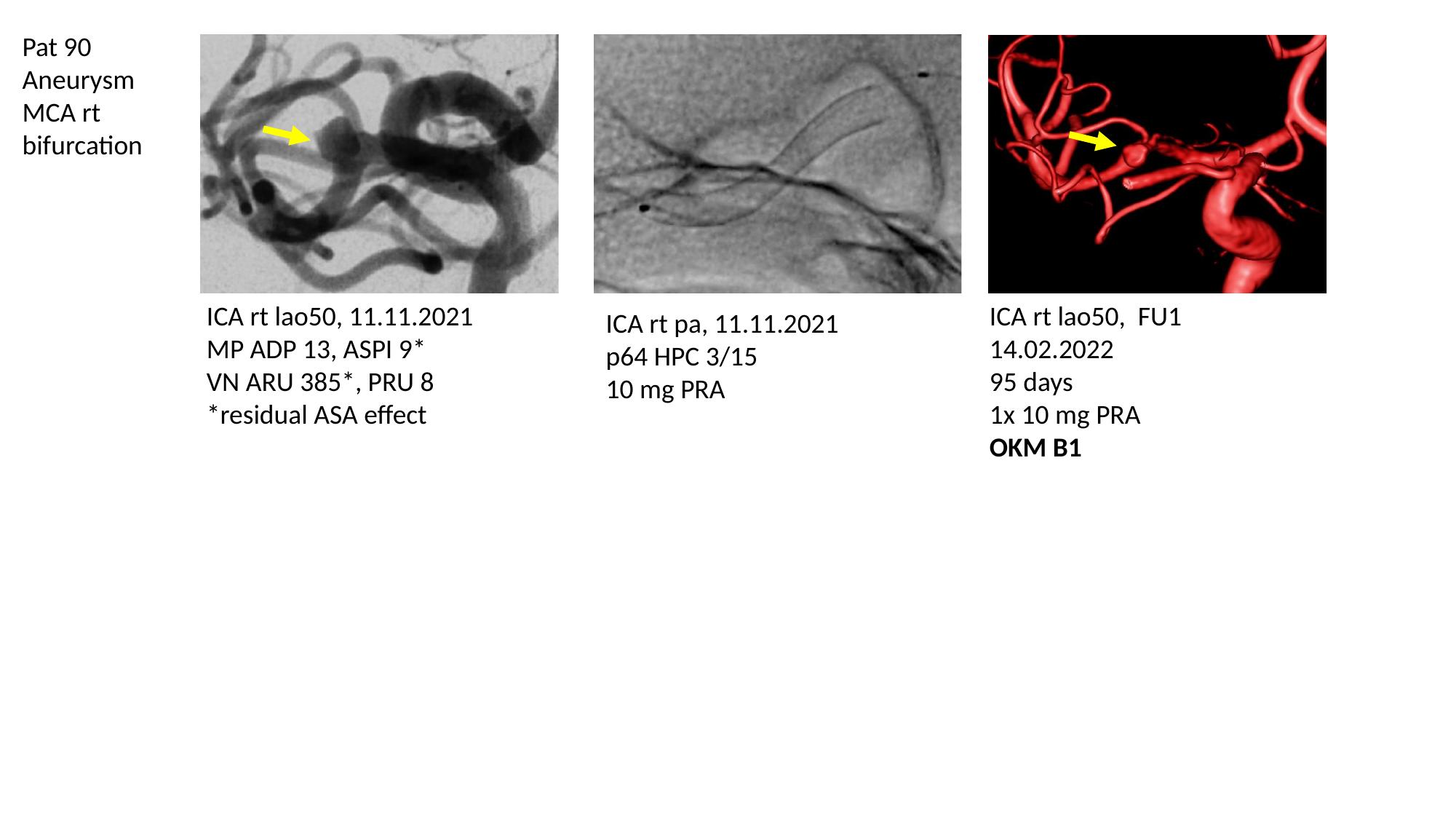

Pat 90
Aneurysm
MCA rt
bifurcation
ICA rt lao50, 11.11.2021
MP ADP 13, ASPI 9*
VN ARU 385*, PRU 8
*residual ASA effect
ICA rt lao50, FU1
14.02.2022
95 days
1x 10 mg PRA
OKM B1
ICA rt pa, 11.11.2021
p64 HPC 3/15
10 mg PRA

## Slide 33
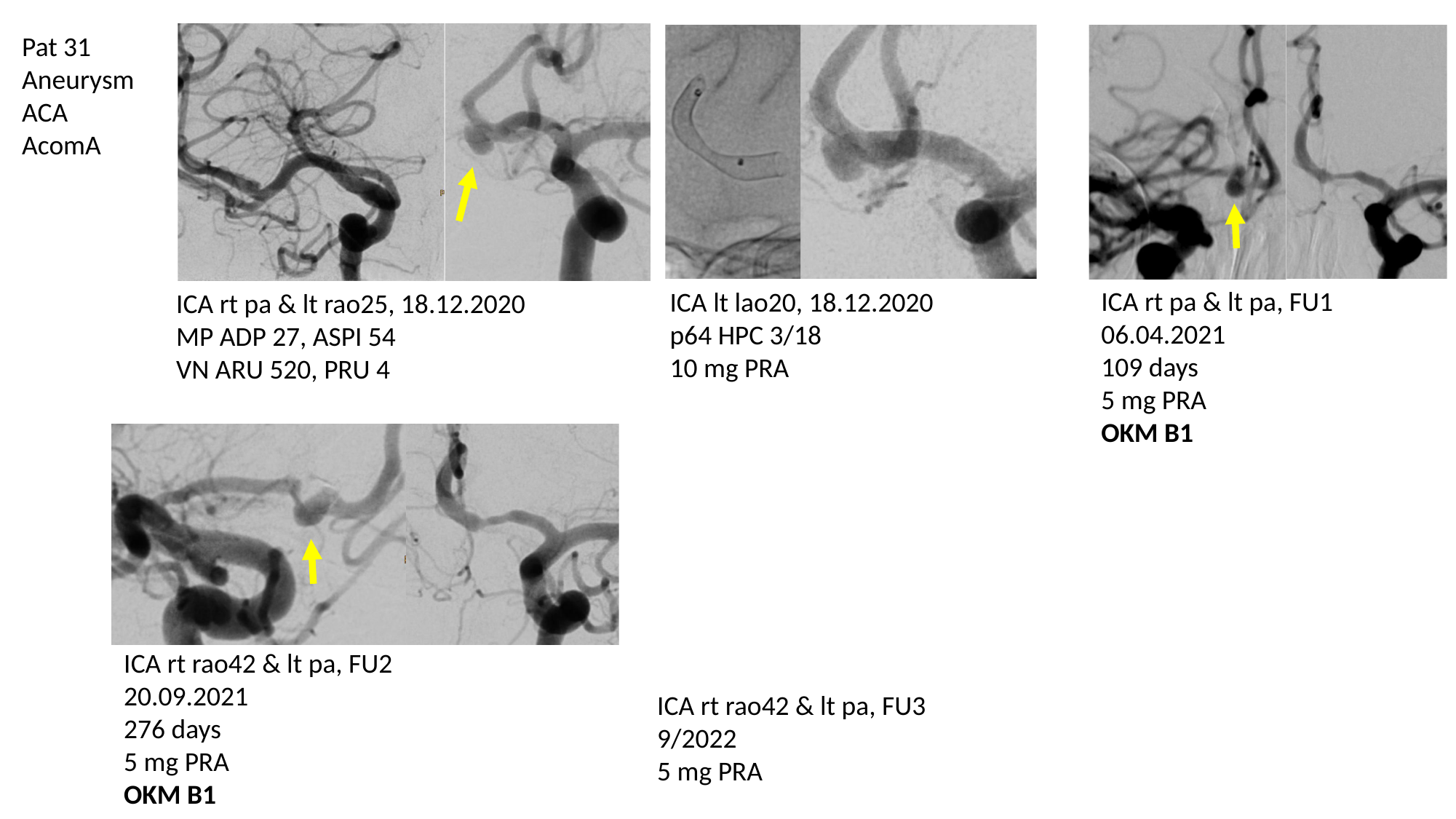

Pat 31
Aneurysm
ACA
AcomA
ICA rt pa & lt pa, FU1
06.04.2021
109 days
5 mg PRA
OKM B1
ICA lt lao20, 18.12.2020
p64 HPC 3/18
10 mg PRA
ICA rt pa & lt rao25, 18.12.2020
MP ADP 27, ASPI 54
VN ARU 520, PRU 4
ICA rt rao42 & lt pa, FU2
20.09.2021
276 days
5 mg PRA
OKM B1
ICA rt rao42 & lt pa, FU3
9/2022
5 mg PRA

## Slide 34
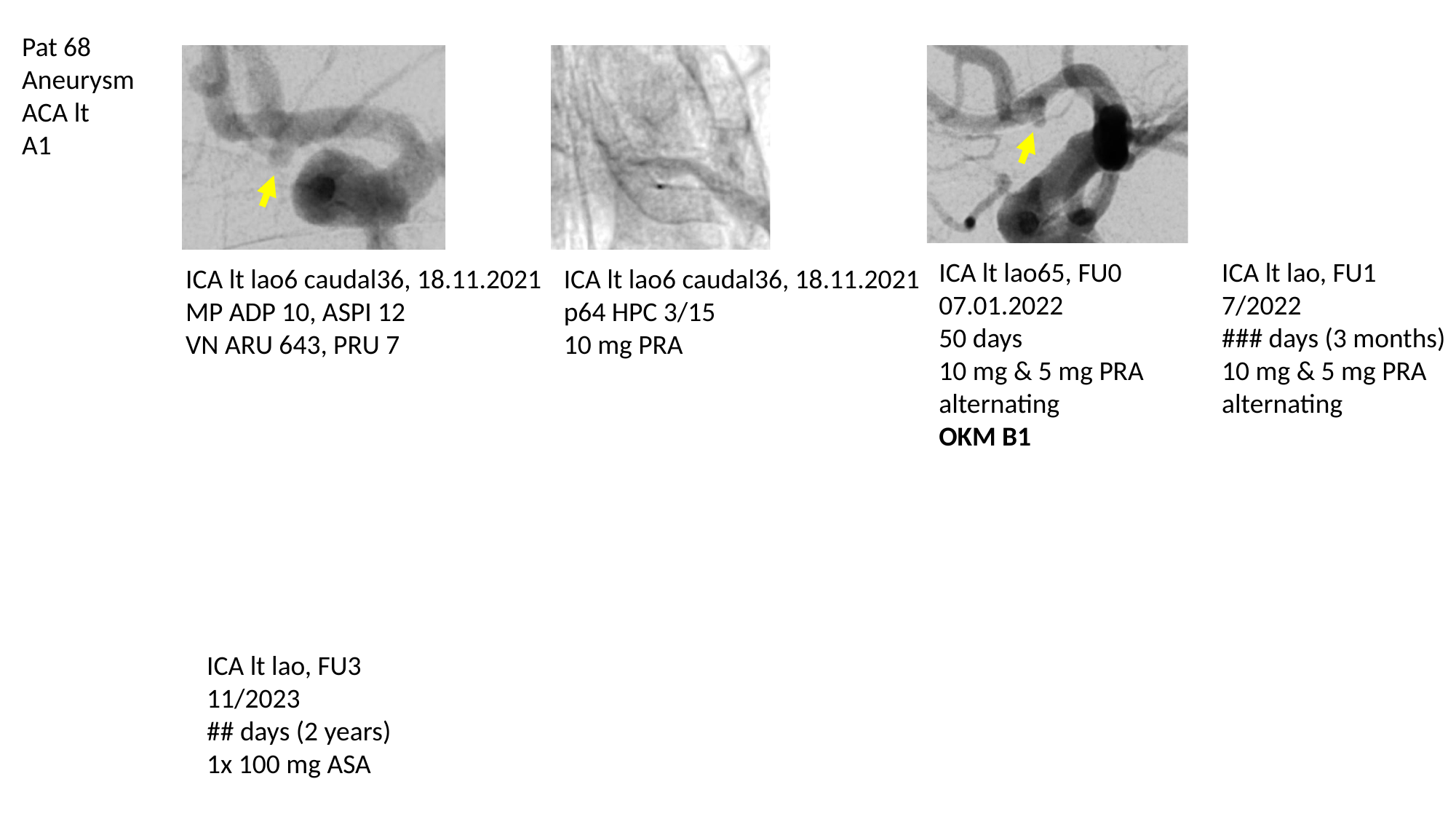

Pat 68
Aneurysm
ACA lt
A1
ICA lt lao65, FU0
07.01.2022
50 days
10 mg & 5 mg PRA
alternating
OKM B1
ICA lt lao, FU1
7/2022
### days (3 months)
10 mg & 5 mg PRA
alternating
ICA lt lao6 caudal36, 18.11.2021
MP ADP 10, ASPI 12
VN ARU 643, PRU 7
ICA lt lao6 caudal36, 18.11.2021
p64 HPC 3/15
10 mg PRA
ICA lt lao, FU3
11/2023
## days (2 years)
1x 100 mg ASA

## Slide 35
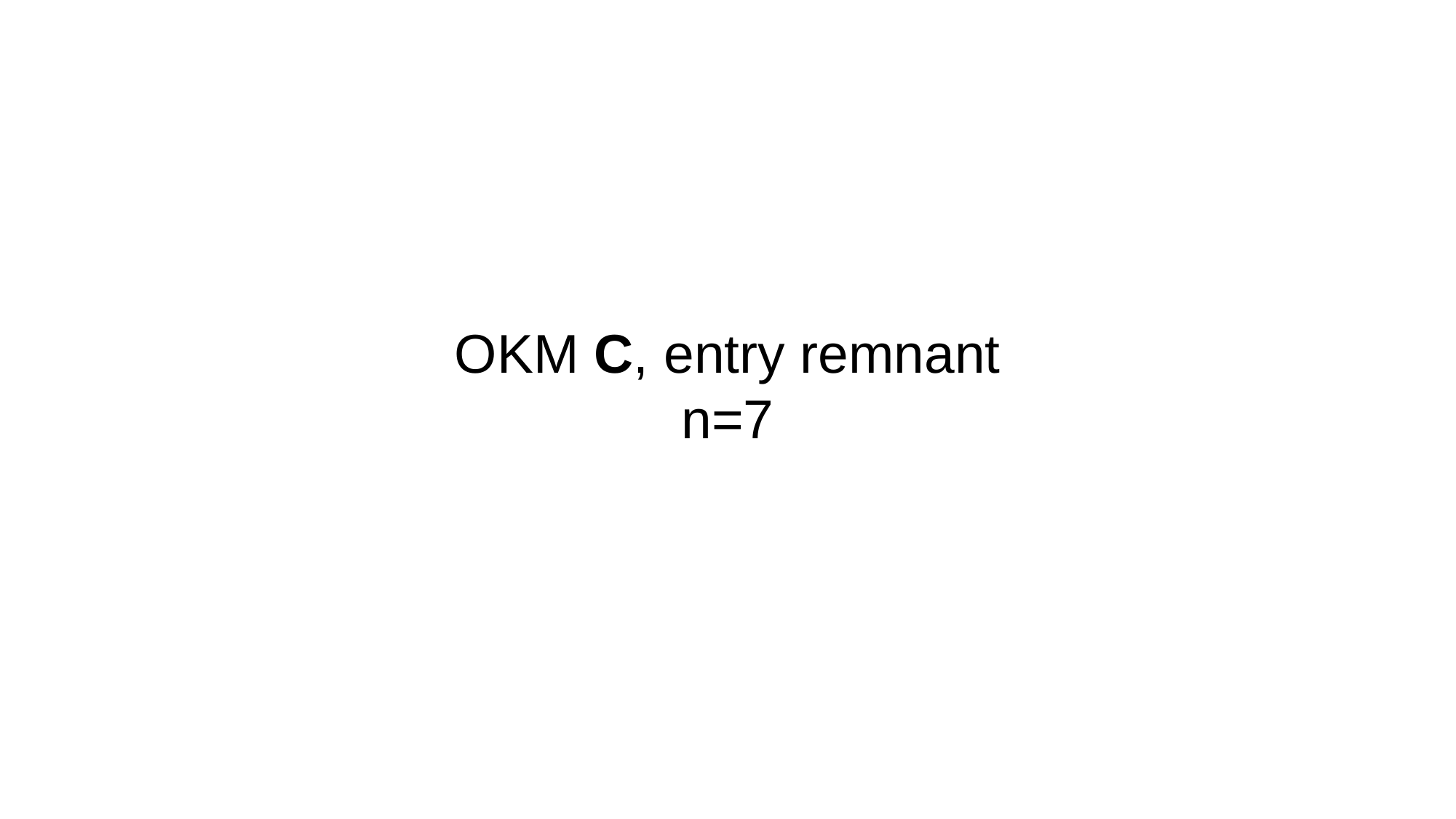

OKM C, entry remnant
n=7

## Slide 36
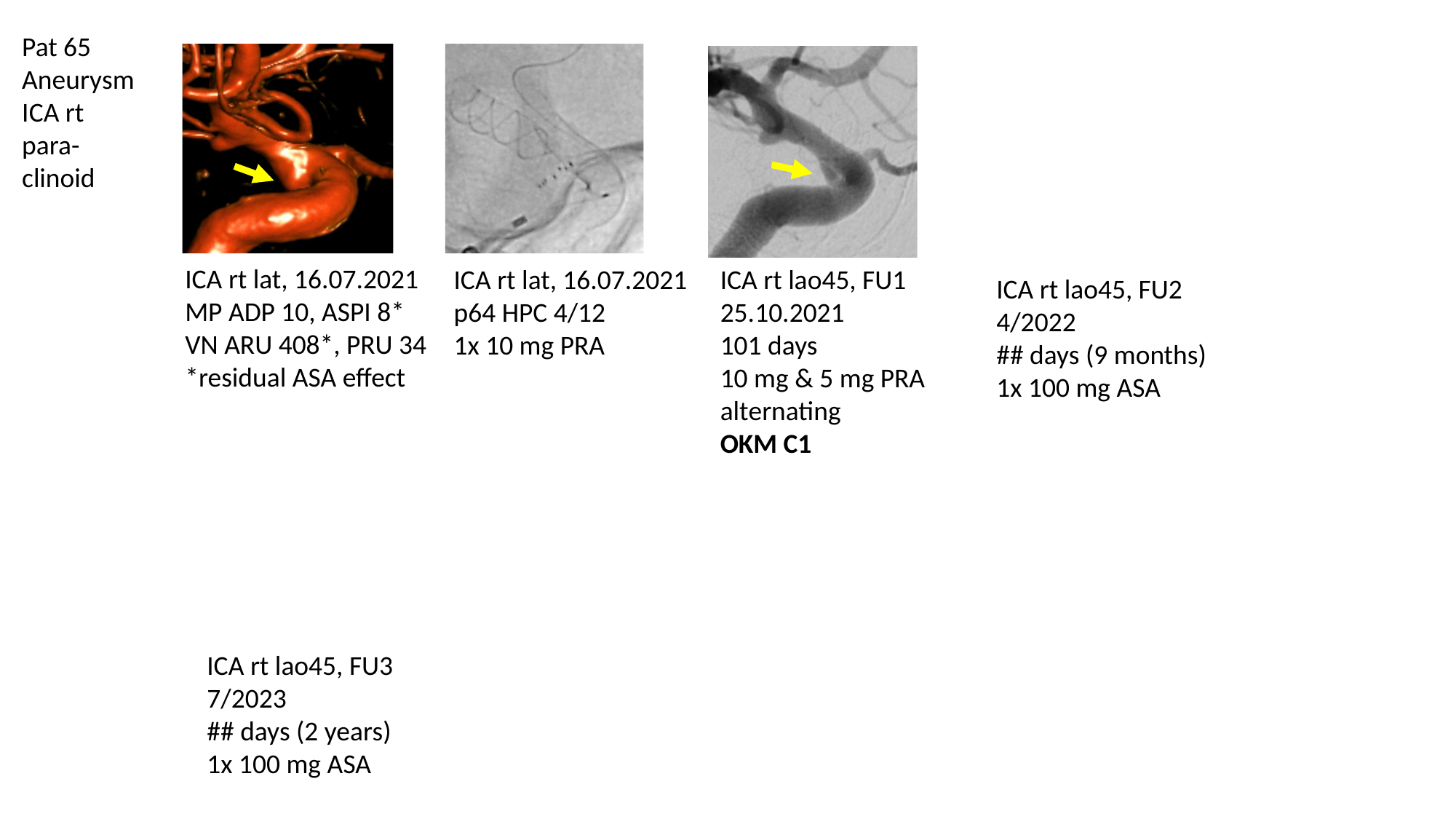

Pat 65
Aneurysm
ICA rt
para-
clinoid
ICA rt lat, 16.07.2021
MP ADP 10, ASPI 8*
VN ARU 408*, PRU 34
*residual ASA effect
ICA rt lat, 16.07.2021
p64 HPC 4/12
1x 10 mg PRA
ICA rt lao45, FU1
25.10.2021
101 days
10 mg & 5 mg PRA
alternating
OKM C1
ICA rt lao45, FU2
4/2022
## days (9 months)
1x 100 mg ASA
ICA rt lao45, FU3
7/2023
## days (2 years)
1x 100 mg ASA

## Slide 37
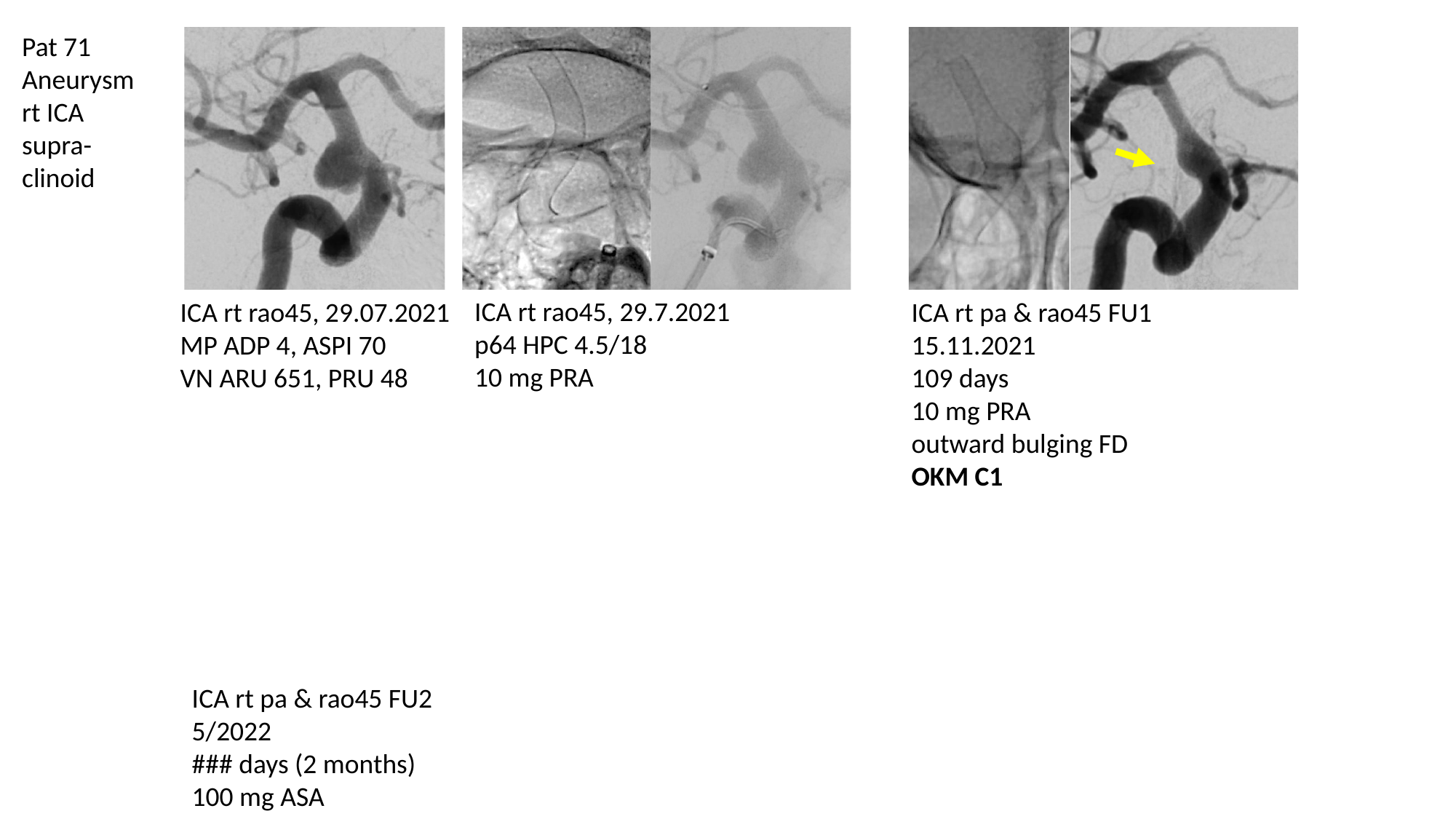

Pat 71
Aneurysm
rt ICA
supra-
clinoid
ICA rt rao45, 29.7.2021
p64 HPC 4.5/18
10 mg PRA
ICA rt pa & rao45 FU1
15.11.2021
109 days
10 mg PRA
outward bulging FD
OKM C1
ICA rt rao45, 29.07.2021
MP ADP 4, ASPI 70
VN ARU 651, PRU 48
ICA rt pa & rao45 FU2
5/2022
### days (2 months)
100 mg ASA

## Slide 38
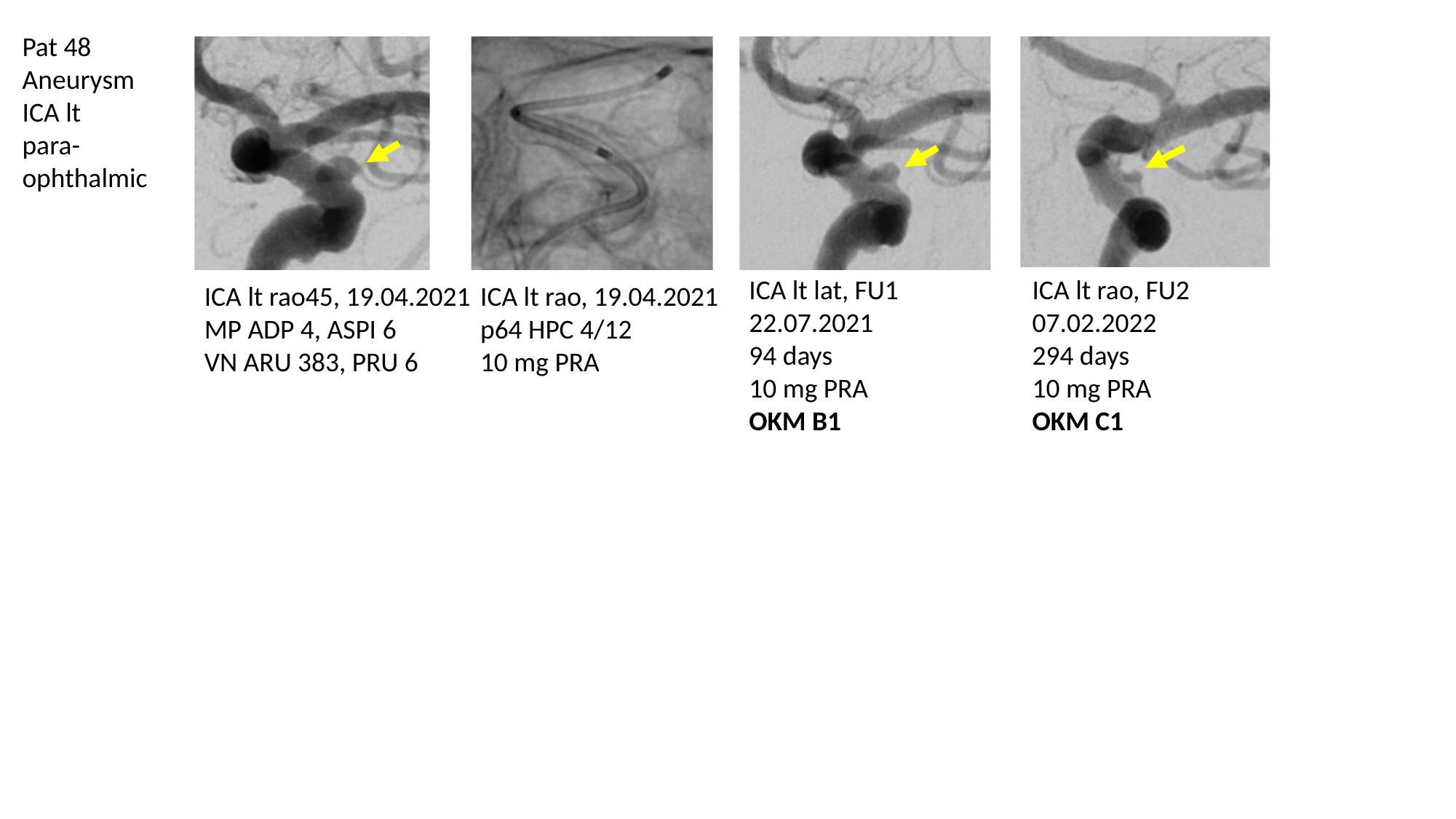

Pat 48
Aneurysm
ICA lt
para-
ophthalmic
ICA lt lat, FU1
22.07.2021
94 days
10 mg PRA
OKM B1
ICA lt rao, FU2
07.02.2022
294 days
10 mg PRA
OKM C1
ICA lt rao, 19.04.2021
p64 HPC 4/12
10 mg PRA
ICA lt rao45, 19.04.2021
MP ADP 4, ASPI 6
VN ARU 383, PRU 6

## Slide 39
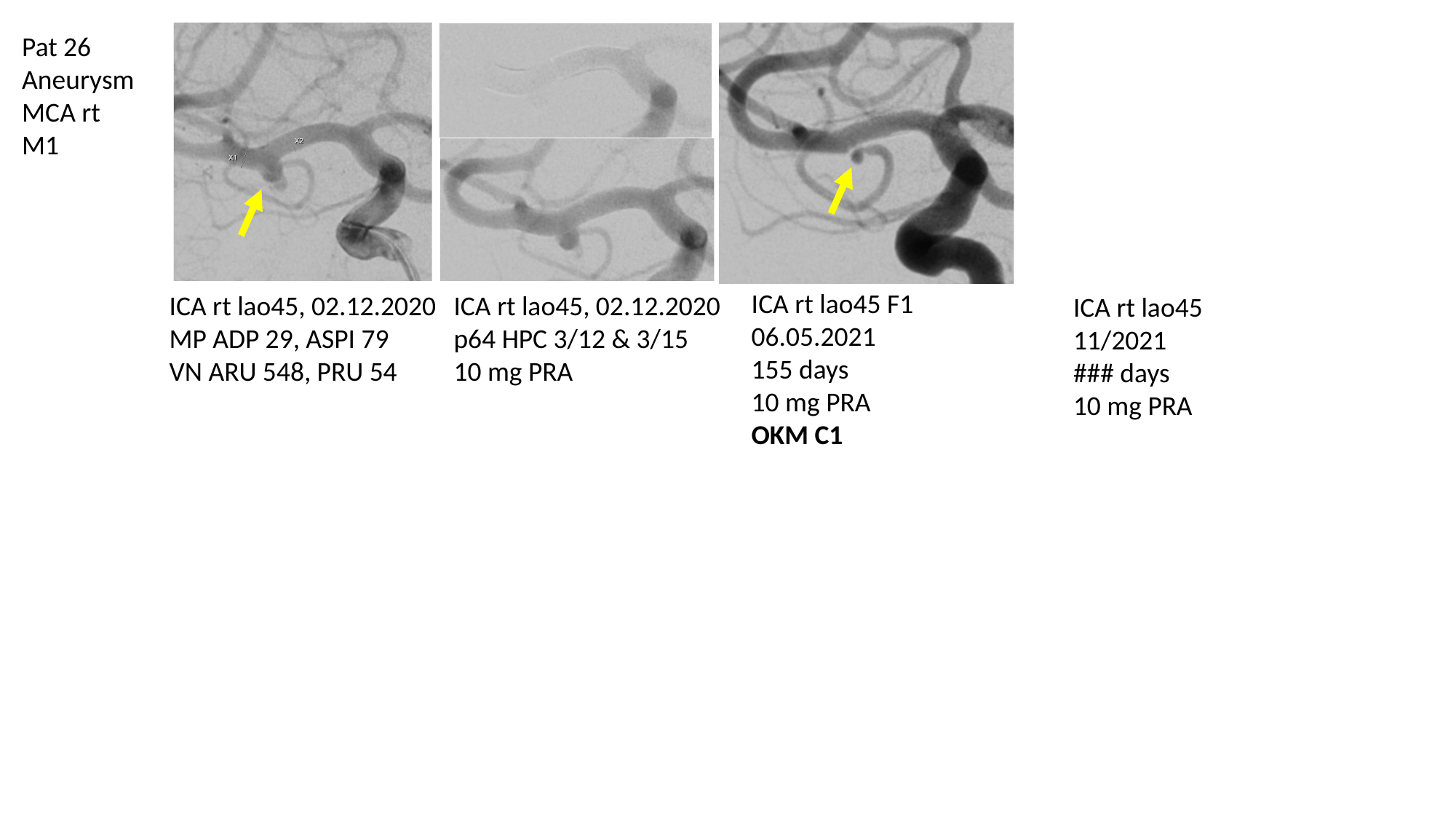

Pat 26
Aneurysm
MCA rt
M1
ICA rt lao45 F1
06.05.2021
155 days
10 mg PRA
OKM C1
ICA rt lao45, 02.12.2020
MP ADP 29, ASPI 79
VN ARU 548, PRU 54
ICA rt lao45, 02.12.2020
p64 HPC 3/12 & 3/15
10 mg PRA
ICA rt lao45
11/2021
### days
10 mg PRA

## Slide 40
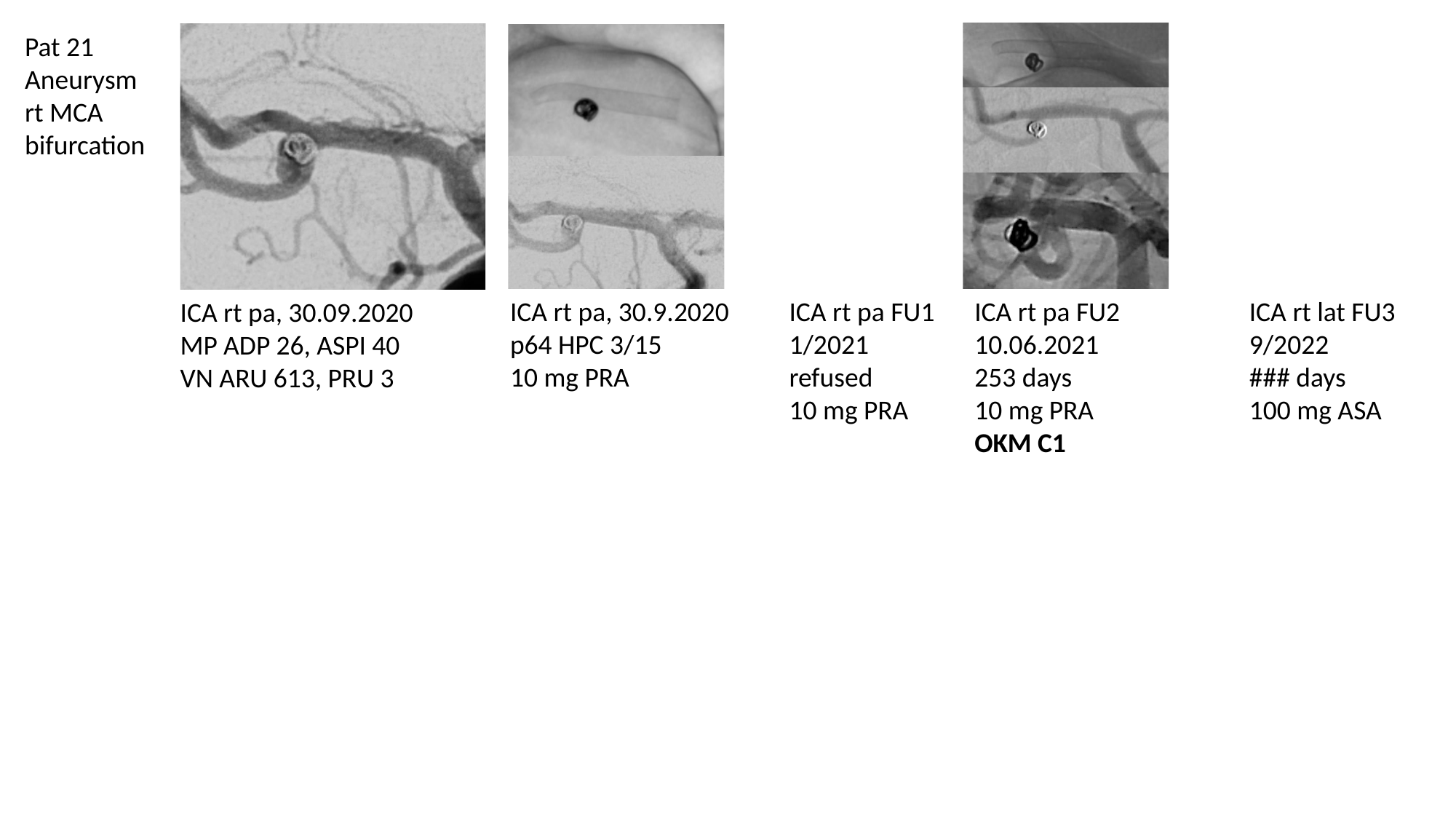

Pat 21
Aneurysm
rt MCA
bifurcation
ICA rt pa FU1
1/2021
refused
10 mg PRA
ICA rt lat FU3
9/2022
### days
100 mg ASA
ICA rt pa, 30.9.2020
p64 HPC 3/15
10 mg PRA
ICA rt pa FU2
10.06.2021
253 days
10 mg PRA
OKM C1
ICA rt pa, 30.09.2020
MP ADP 26, ASPI 40
VN ARU 613, PRU 3

## Slide 41
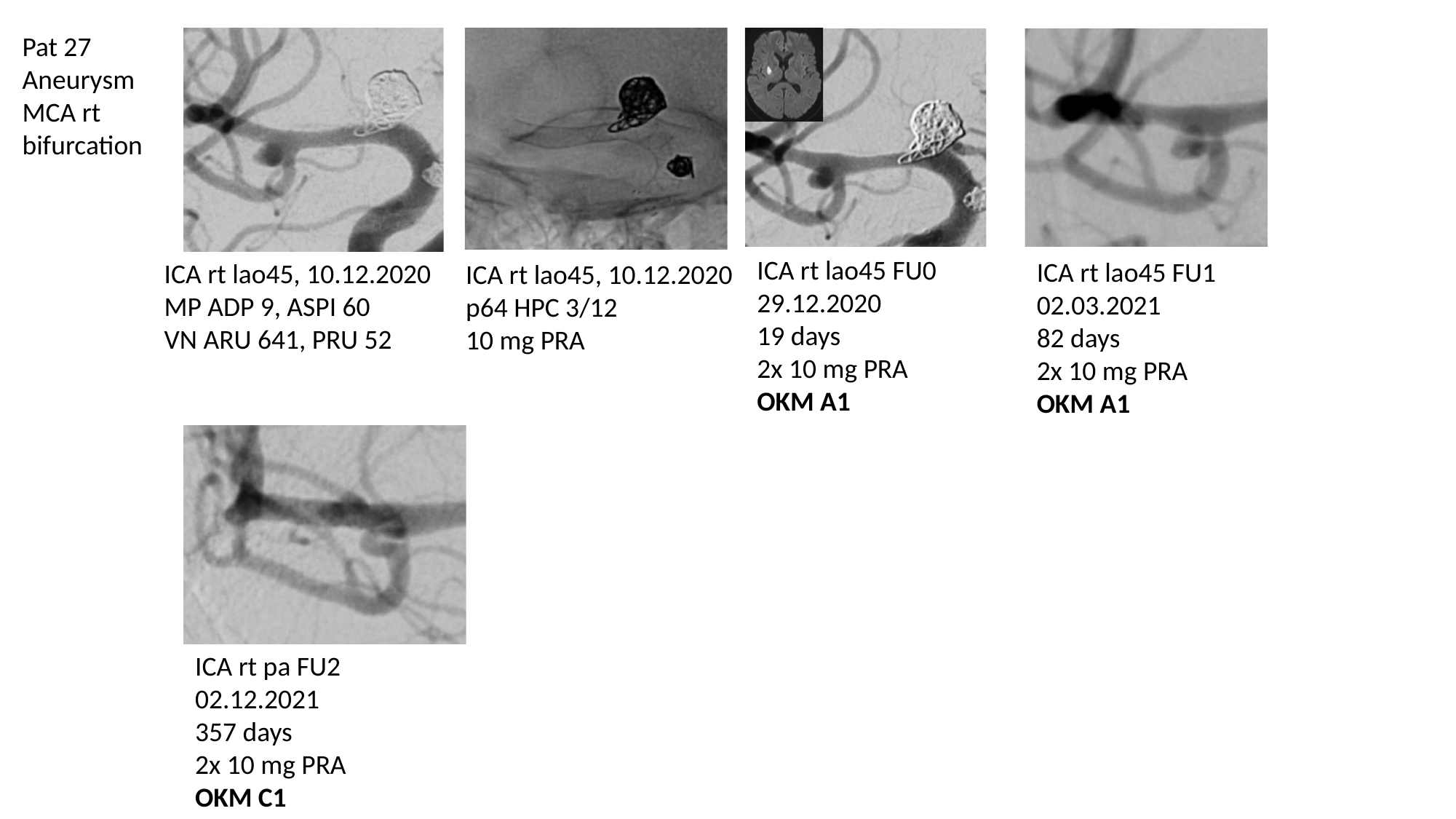

Pat 27
Aneurysm
MCA rt
bifurcation
ICA rt lao45 FU0
29.12.2020
19 days
2x 10 mg PRA
OKM A1
ICA rt lao45 FU1
02.03.2021
82 days
2x 10 mg PRA
OKM A1
ICA rt lao45, 10.12.2020
MP ADP 9, ASPI 60
VN ARU 641, PRU 52
ICA rt lao45, 10.12.2020
p64 HPC 3/12
10 mg PRA
ICA rt pa FU2
02.12.2021
357 days
2x 10 mg PRA
OKM C1

## Slide 42
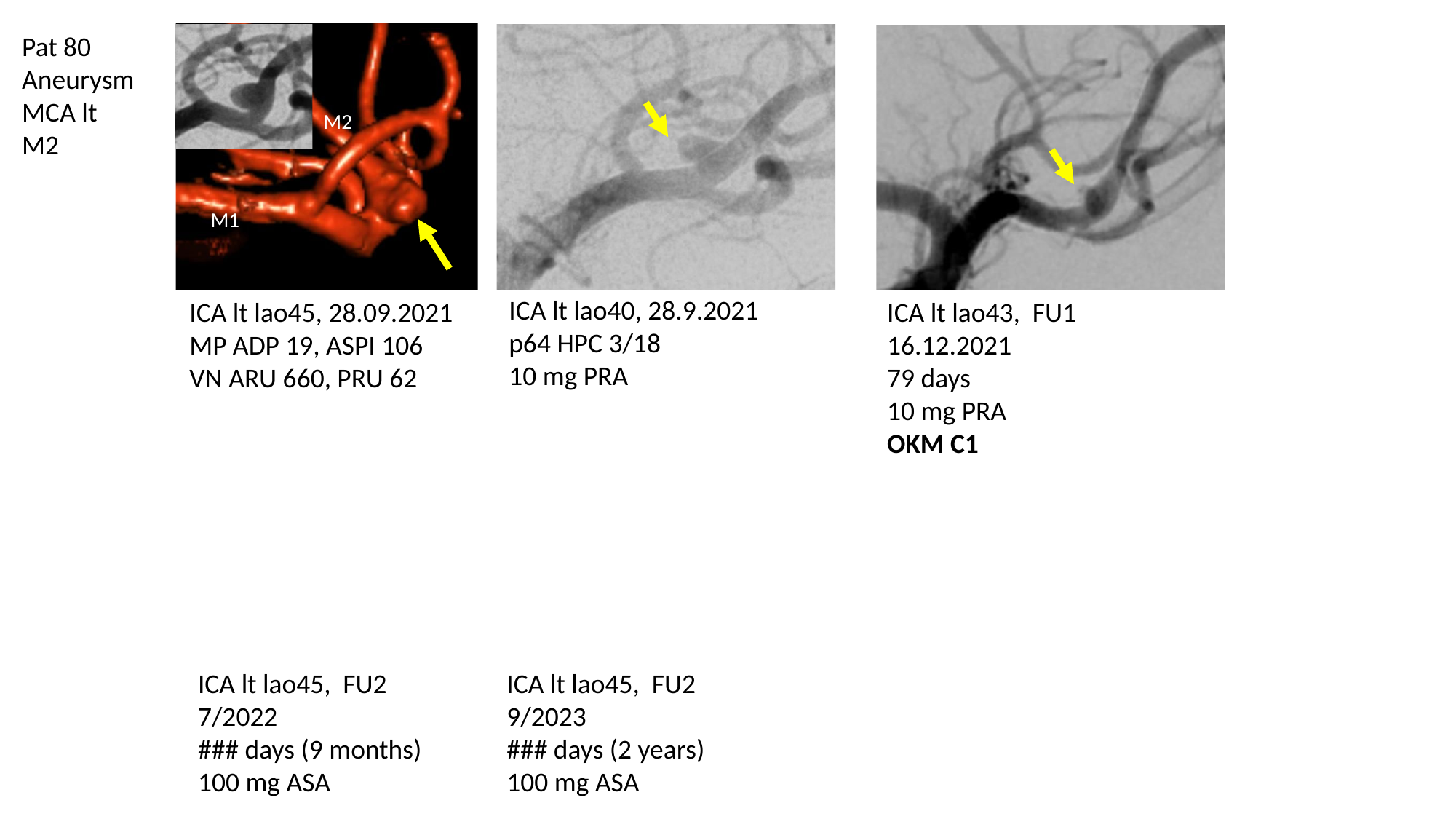

Pat 80
Aneurysm
MCA lt
M2
M2
M1
ICA lt lao40, 28.9.2021
p64 HPC 3/18
10 mg PRA
ICA lt lao43, FU1
16.12.2021
79 days
10 mg PRA
OKM C1
ICA lt lao45, 28.09.2021
MP ADP 19, ASPI 106
VN ARU 660, PRU 62
ICA lt lao45, FU2
7/2022
### days (9 months)
100 mg ASA
ICA lt lao45, FU2
9/2023
### days (2 years)
100 mg ASA

## Slide 43
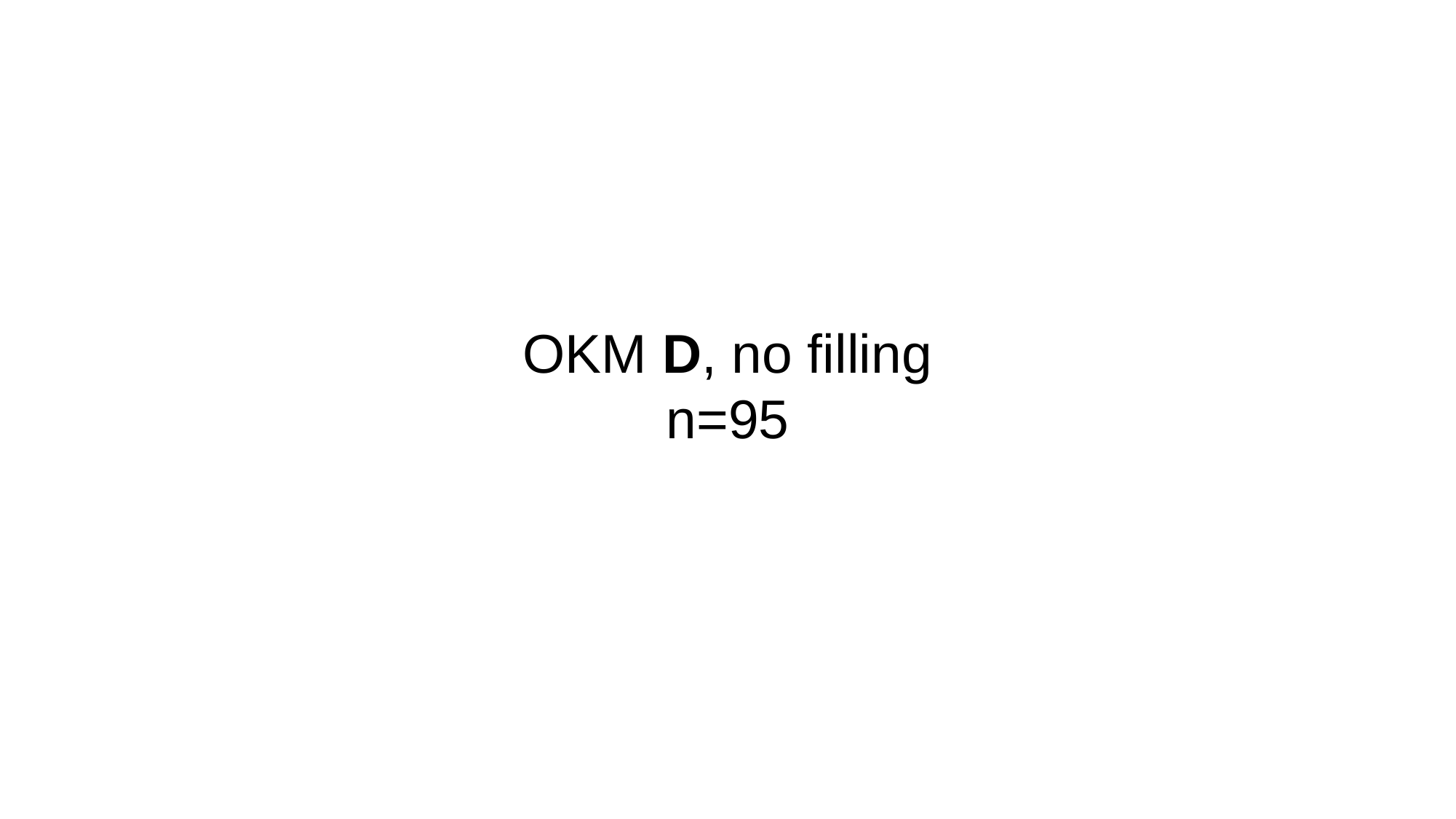

OKM D, no filling
n=95

## Slide 44
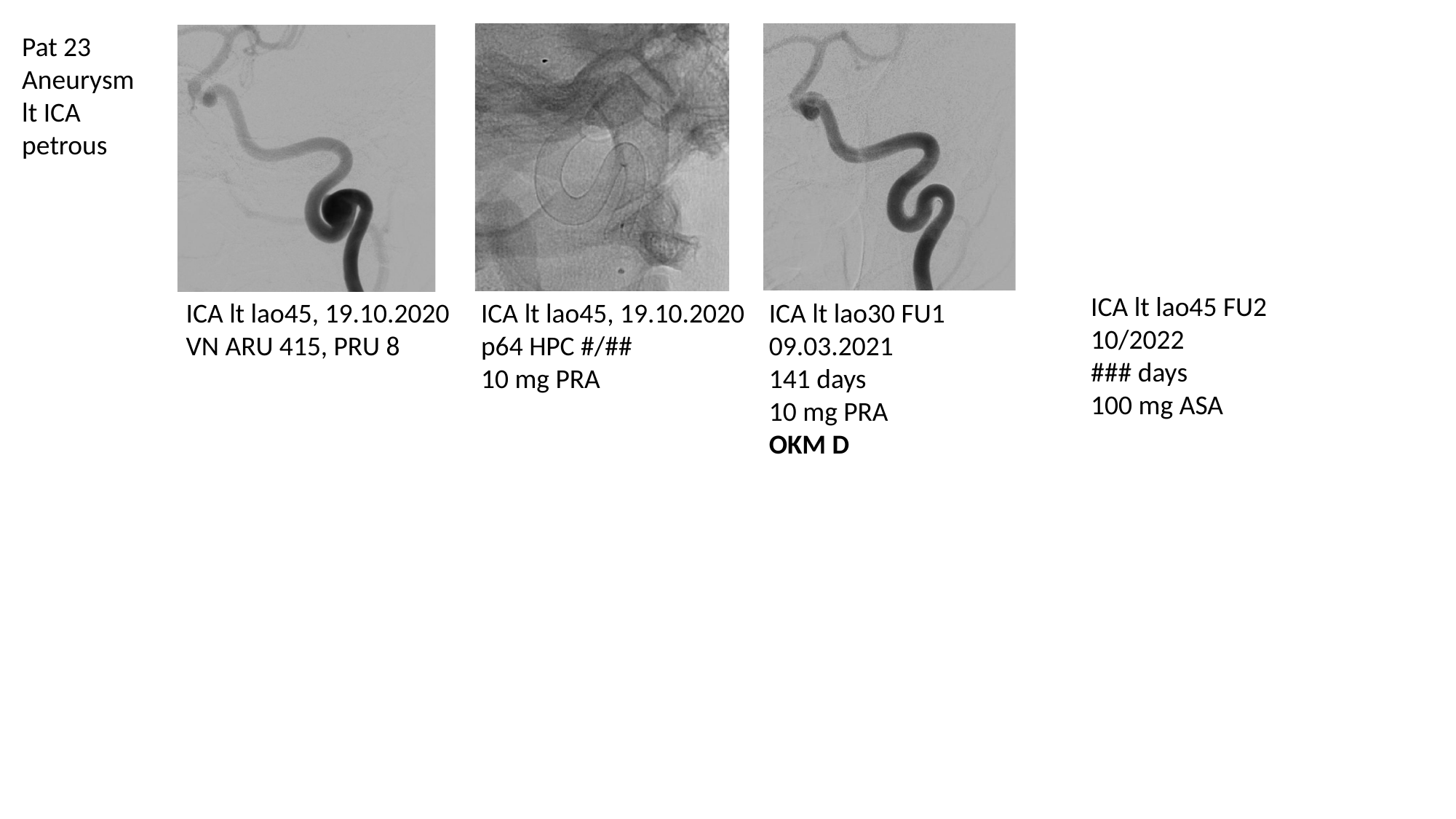

Pat 23
Aneurysm
lt ICA
petrous
ICA lt lao45 FU2
10/2022
### days
100 mg ASA
ICA lt lao45, 19.10.2020
VN ARU 415, PRU 8
ICA lt lao45, 19.10.2020
p64 HPC #/##
10 mg PRA
ICA lt lao30 FU1
09.03.2021
141 days
10 mg PRA
OKM D

## Slide 45
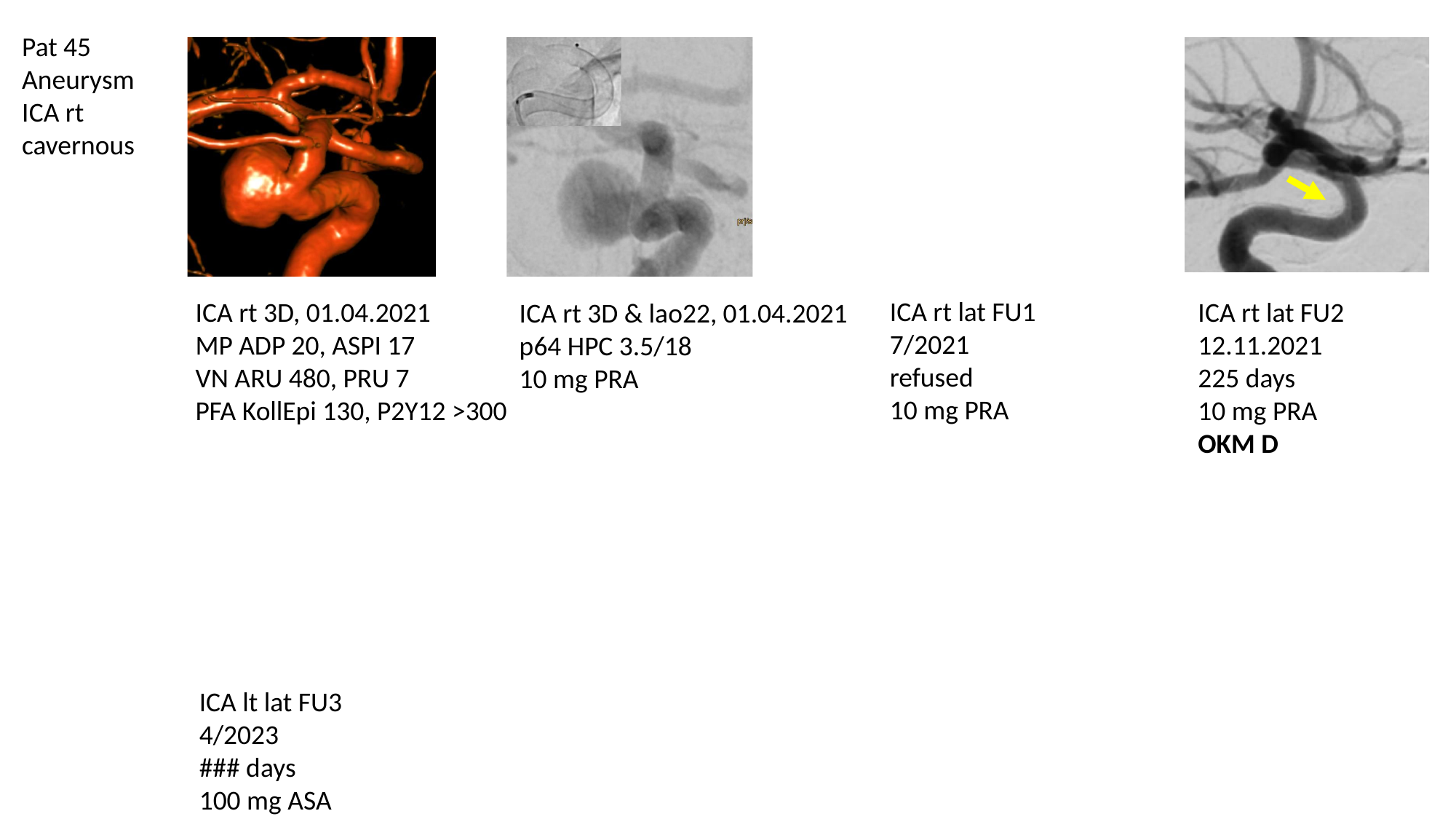

Pat 45
Aneurysm
ICA rt
cavernous
ICA rt lat FU1
7/2021
refused
10 mg PRA
ICA rt 3D, 01.04.2021
MP ADP 20, ASPI 17
VN ARU 480, PRU 7
PFA KollEpi 130, P2Y12 >300
ICA rt lat FU2
12.11.2021
225 days
10 mg PRA
OKM D
ICA rt 3D & lao22, 01.04.2021
p64 HPC 3.5/18
10 mg PRA
ICA lt lat FU3
4/2023
### days
100 mg ASA

## Slide 46
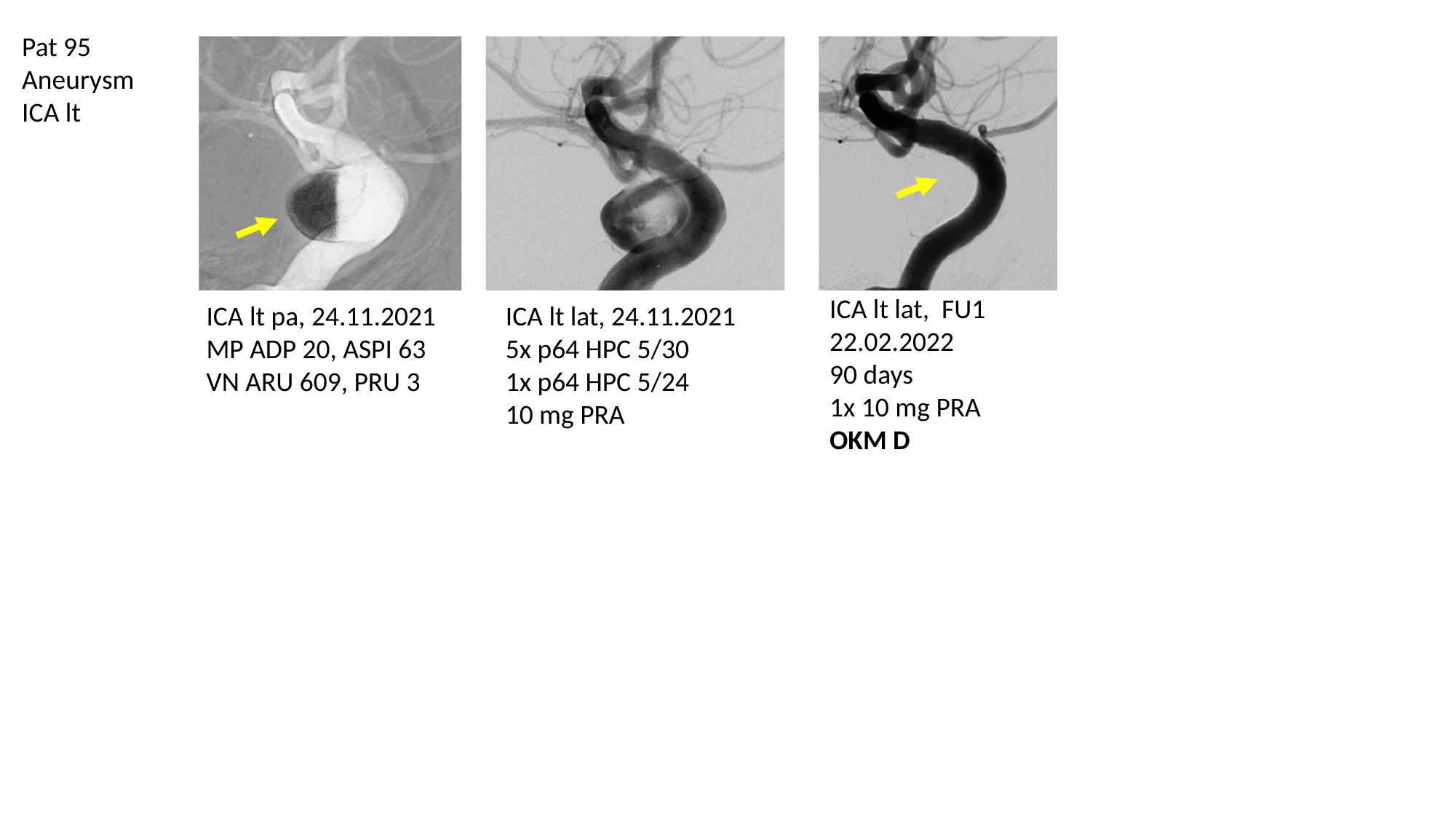

Pat 95
Aneurysm
ICA lt
ICA lt lat, FU1
22.02.2022
90 days
1x 10 mg PRA
OKM D
ICA lt lat, 24.11.2021
5x p64 HPC 5/30
1x p64 HPC 5/24
10 mg PRA
ICA lt pa, 24.11.2021
MP ADP 20, ASPI 63
VN ARU 609, PRU 3

## Slide 47
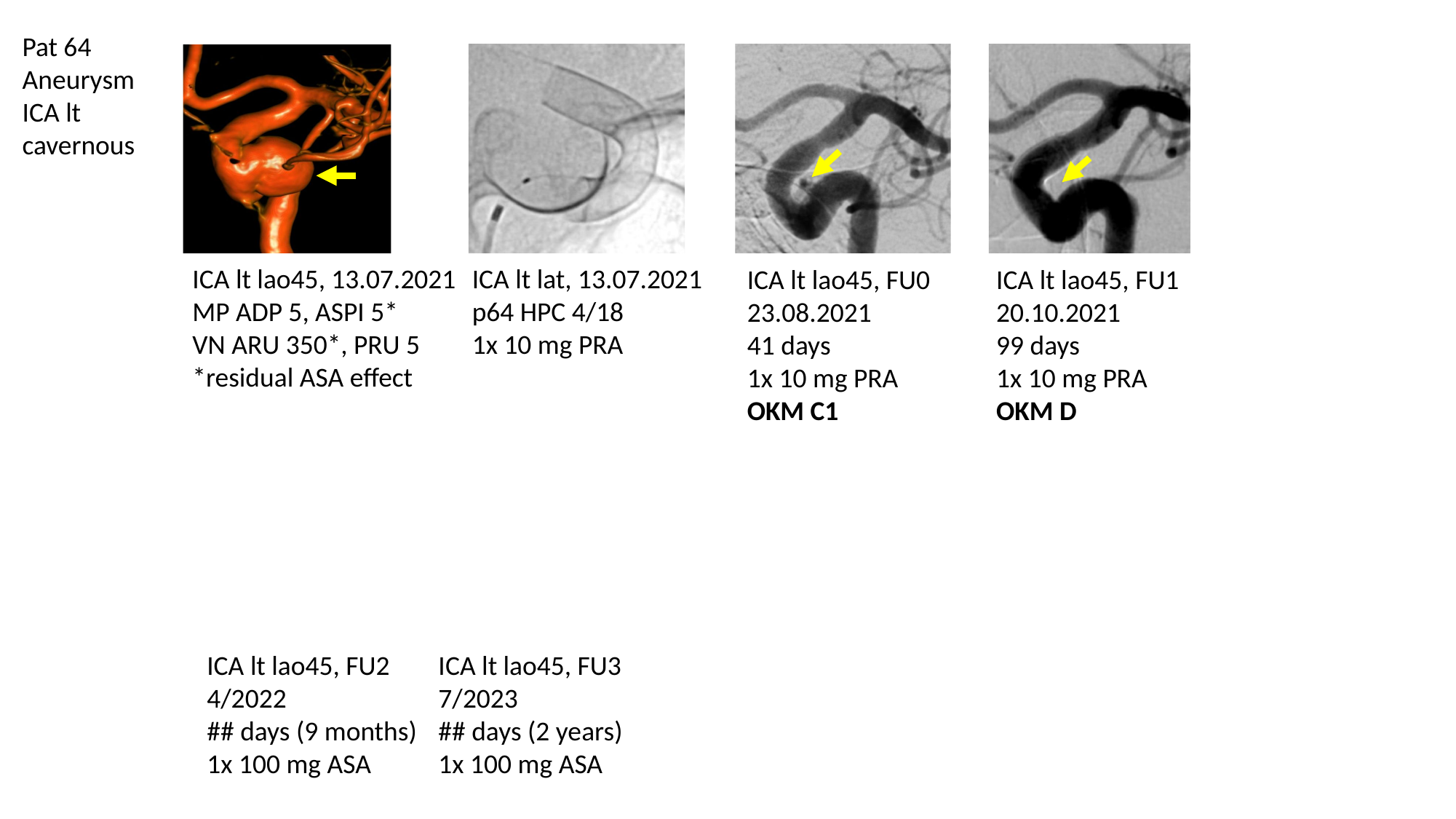

Pat 64
Aneurysm
ICA lt
cavernous
ICA lt lao45, 13.07.2021
MP ADP 5, ASPI 5*
VN ARU 350*, PRU 5
*residual ASA effect
ICA lt lat, 13.07.2021
p64 HPC 4/18
1x 10 mg PRA
ICA lt lao45, FU1
20.10.2021
99 days
1x 10 mg PRA
OKM D
ICA lt lao45, FU0
23.08.2021
41 days
1x 10 mg PRA
OKM C1
ICA lt lao45, FU3
7/2023
## days (2 years)
1x 100 mg ASA
ICA lt lao45, FU2
4/2022
## days (9 months)
1x 100 mg ASA

## Slide 48
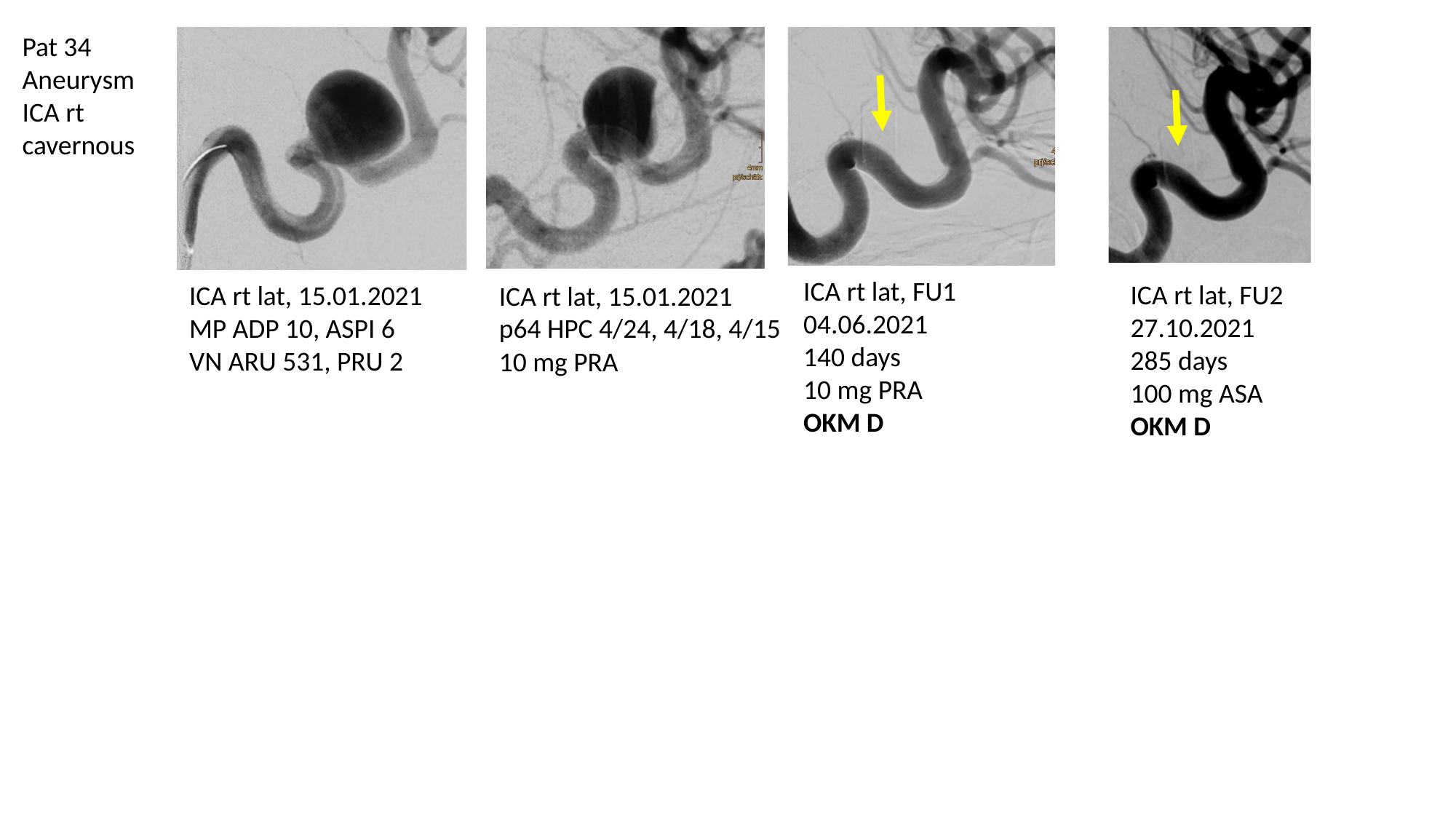

Pat 34
Aneurysm
ICA rt
cavernous
ICA rt lat, FU1
04.06.2021
140 days
10 mg PRA
OKM D
ICA rt lat, FU2
27.10.2021
285 days
100 mg ASA
OKM D
ICA rt lat, 15.01.2021
MP ADP 10, ASPI 6
VN ARU 531, PRU 2
ICA rt lat, 15.01.2021
p64 HPC 4/24, 4/18, 4/15
10 mg PRA

## Slide 49
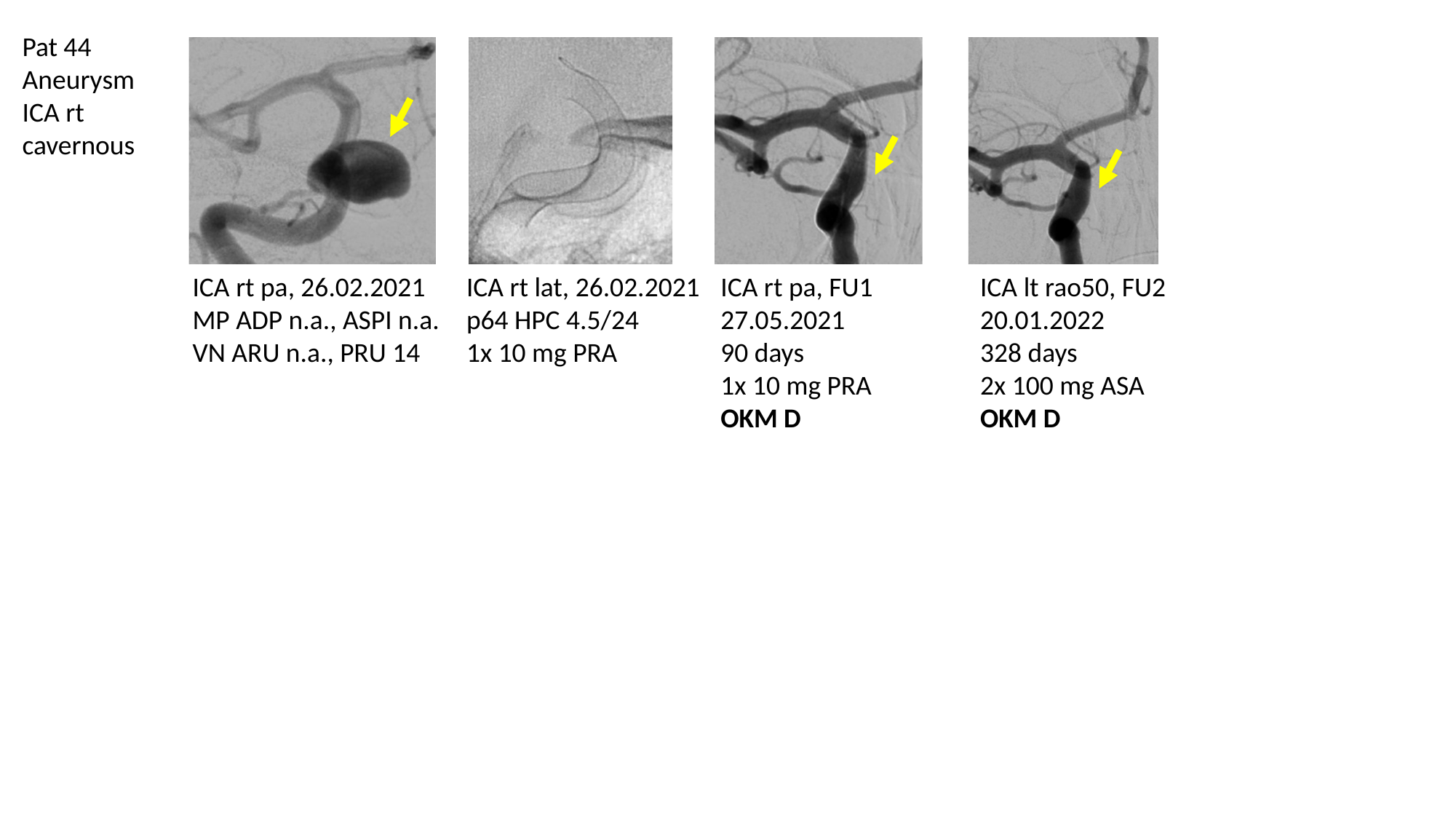

Pat 44
Aneurysm
ICA rt
cavernous
ICA lt rao50, FU2
20.01.2022
328 days
2x 100 mg ASA
OKM D
ICA rt lat, 26.02.2021
p64 HPC 4.5/24
1x 10 mg PRA
ICA rt pa, FU1
27.05.2021
90 days
1x 10 mg PRA
OKM D
ICA rt pa, 26.02.2021
MP ADP n.a., ASPI n.a.
VN ARU n.a., PRU 14

## Slide 50
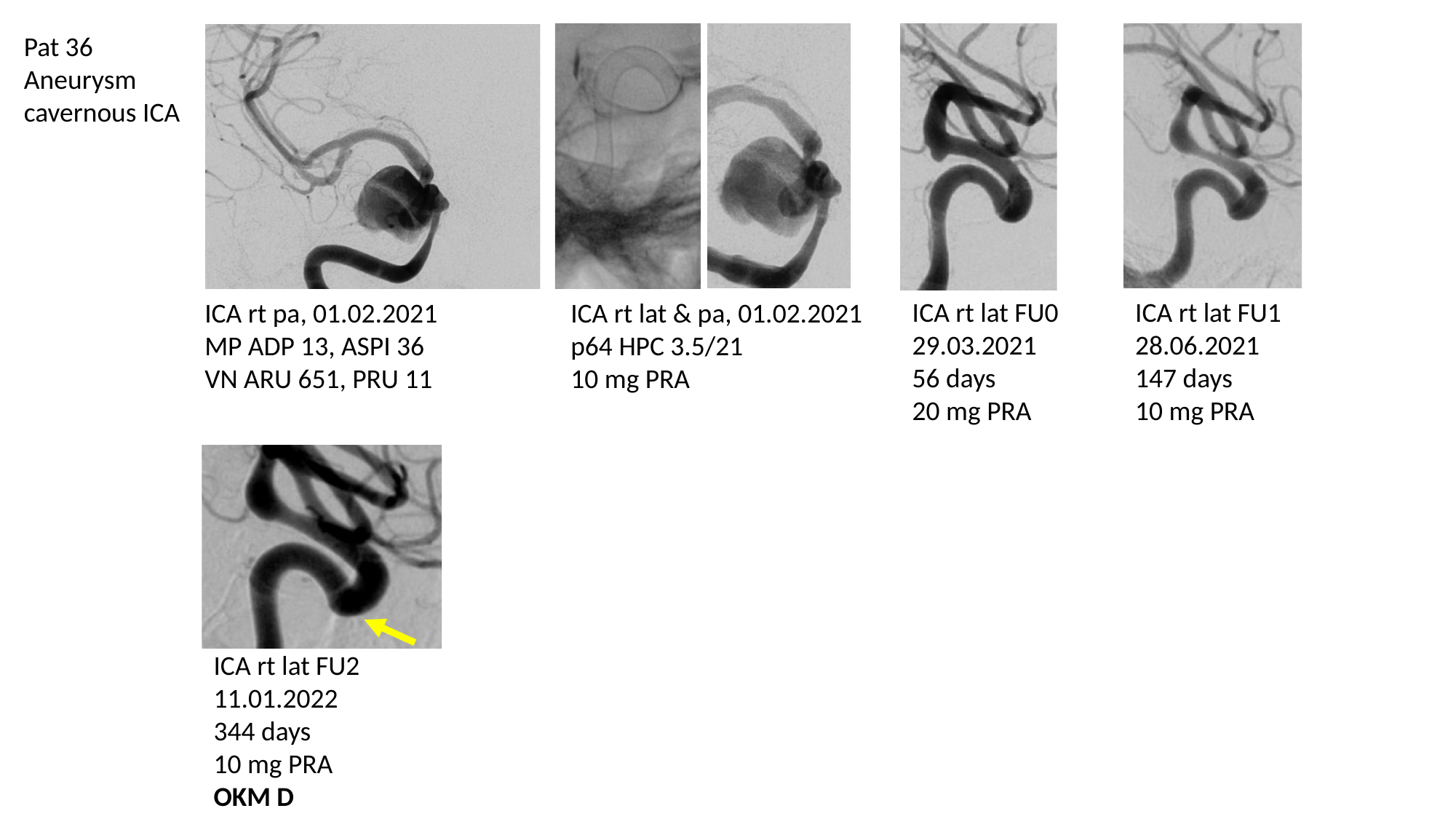

Pat 36
Aneurysm
cavernous ICA
ICA rt lat FU0
29.03.2021
56 days
20 mg PRA
ICA rt lat FU1
28.06.2021
147 days
10 mg PRA
ICA rt pa, 01.02.2021
MP ADP 13, ASPI 36
VN ARU 651, PRU 11
ICA rt lat & pa, 01.02.2021
p64 HPC 3.5/21
10 mg PRA
ICA rt lat FU2
11.01.2022
344 days
10 mg PRA
OKM D

## Slide 51
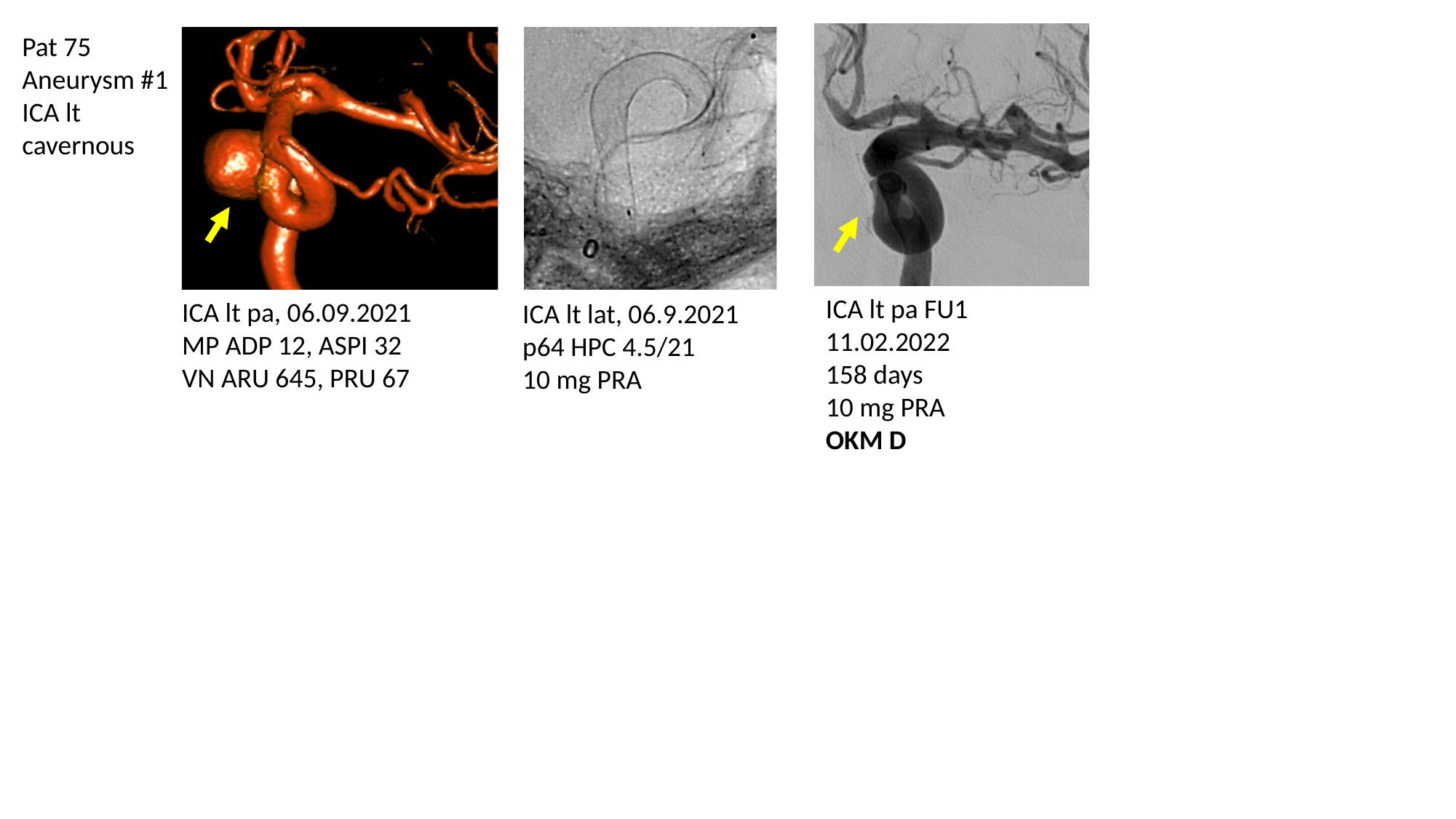

Pat 75
Aneurysm #1
ICA lt
cavernous
ICA lt pa FU1
11.02.2022
158 days
10 mg PRA
OKM D
ICA lt pa, 06.09.2021
MP ADP 12, ASPI 32
VN ARU 645, PRU 67
ICA lt lat, 06.9.2021
p64 HPC 4.5/21
10 mg PRA

## Slide 52
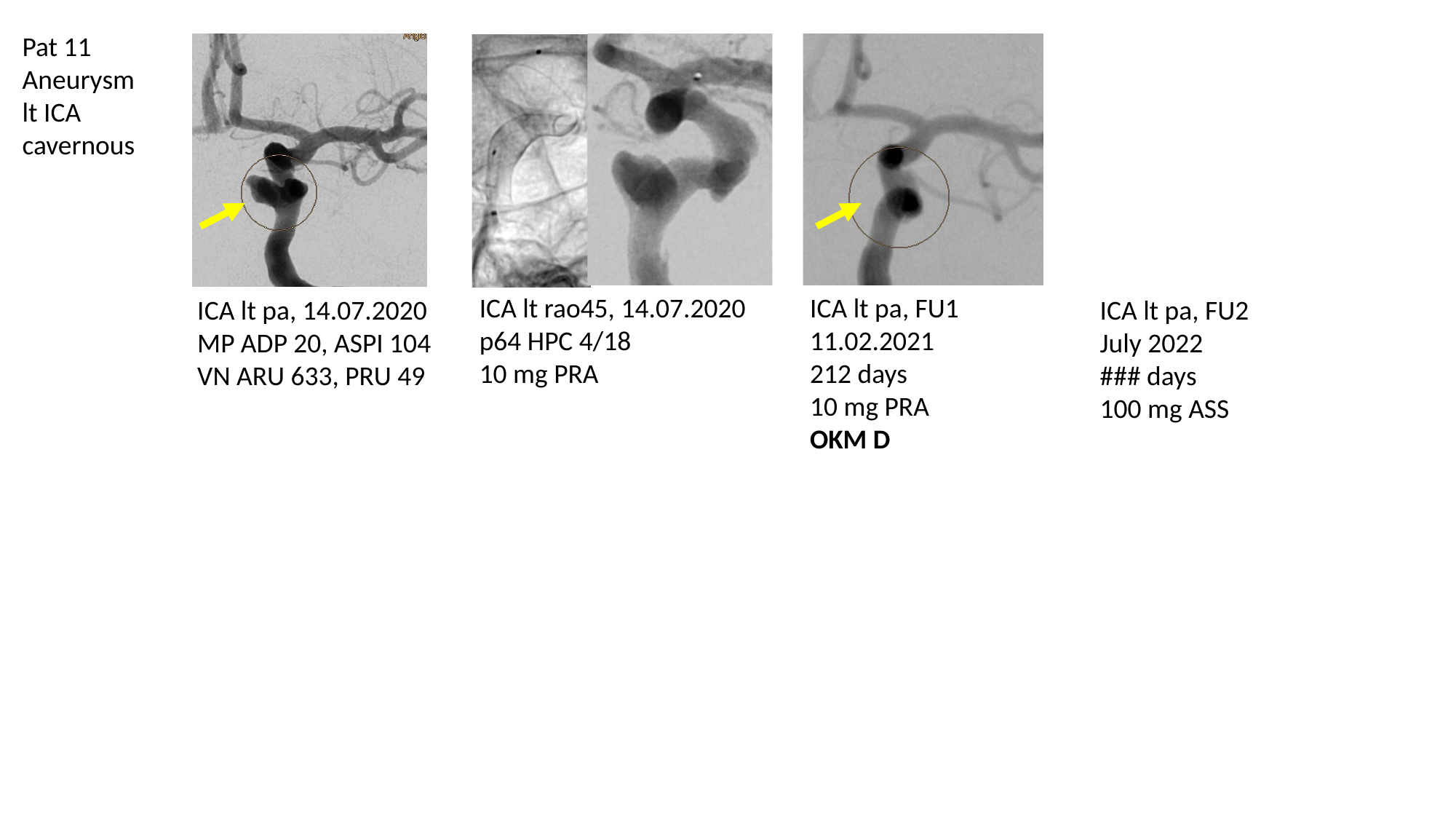

Pat 11
Aneurysm
lt ICA
cavernous
ICA lt rao45, 14.07.2020
p64 HPC 4/18
10 mg PRA
ICA lt pa, FU1
11.02.2021
212 days
10 mg PRA
OKM D
ICA lt pa, 14.07.2020
MP ADP 20, ASPI 104
VN ARU 633, PRU 49
ICA lt pa, FU2
July 2022
### days
100 mg ASS

## Slide 53
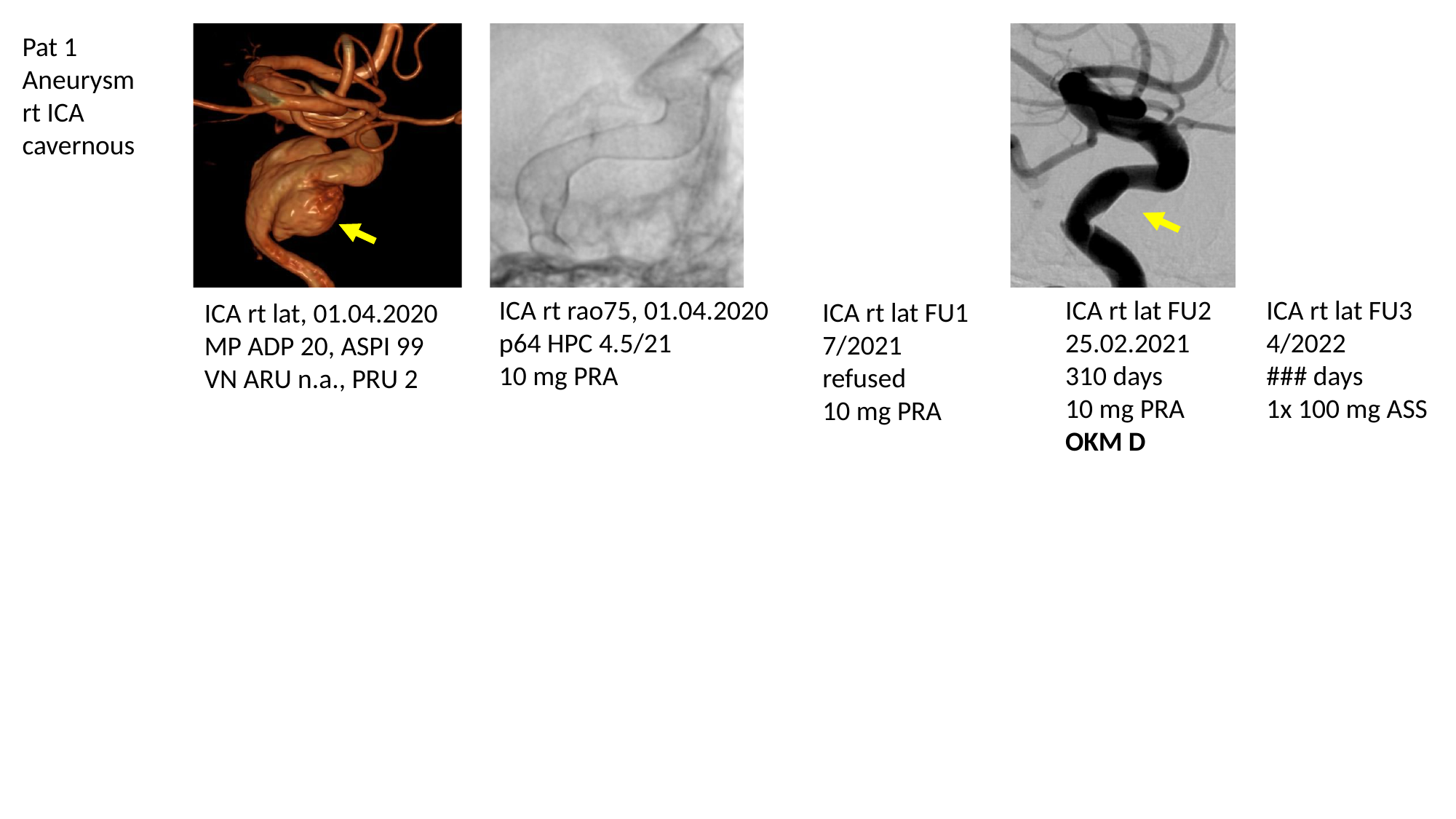

Pat 1
Aneurysm
rt ICA
cavernous
ICA rt rao75, 01.04.2020
p64 HPC 4.5/21
10 mg PRA
ICA rt lat FU2
25.02.2021
310 days
10 mg PRA
OKM D
ICA rt lat FU3
4/2022
### days
1x 100 mg ASS
ICA rt lat FU1
7/2021
refused
10 mg PRA
ICA rt lat, 01.04.2020
MP ADP 20, ASPI 99
VN ARU n.a., PRU 2

## Slide 54
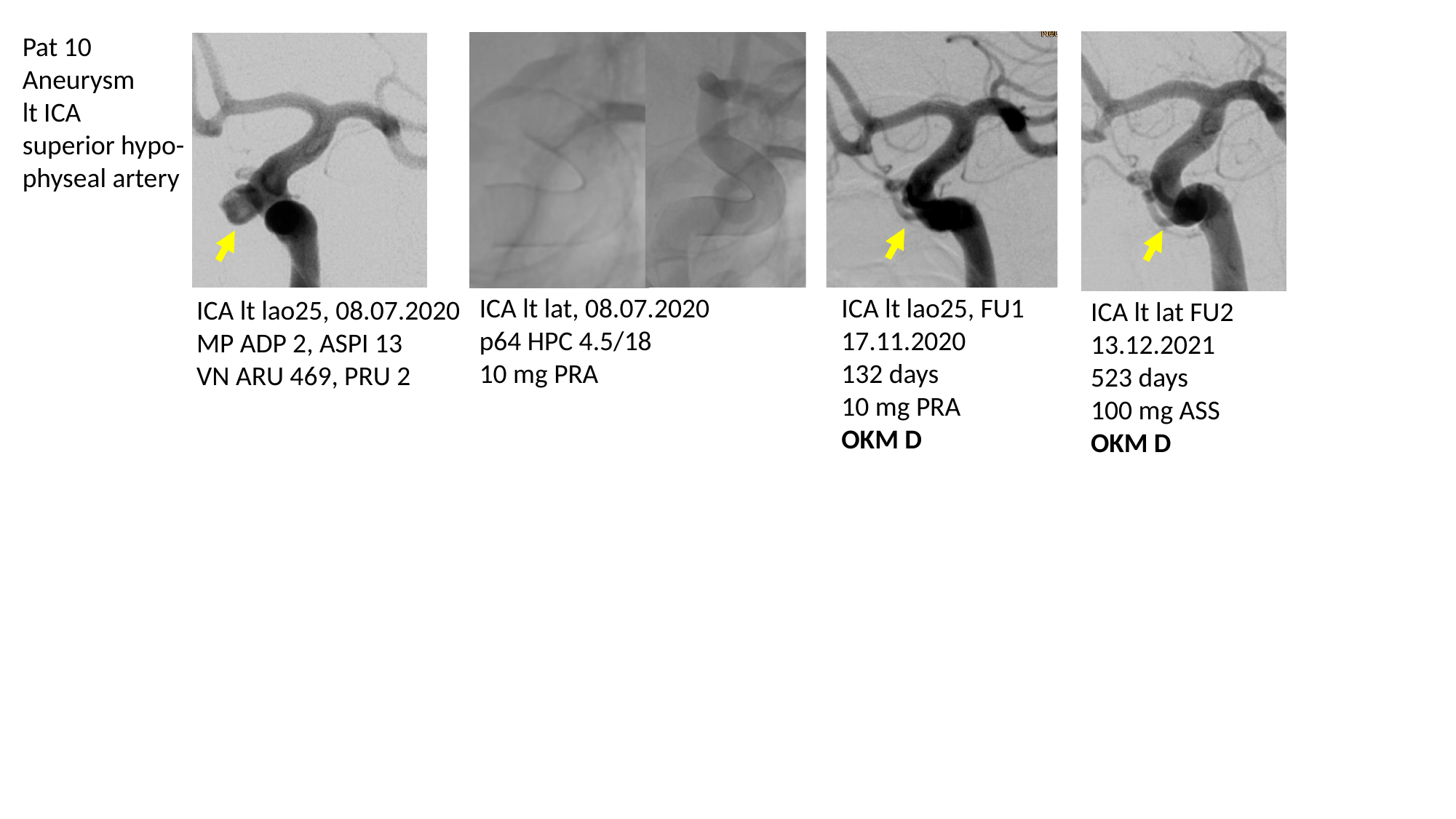

Pat 10
Aneurysm
lt ICA
superior hypo-
physeal artery
ICA lt lat, 08.07.2020
p64 HPC 4.5/18
10 mg PRA
ICA lt lao25, FU1
17.11.2020
132 days
10 mg PRA
OKM D
ICA lt lao25, 08.07.2020
MP ADP 2, ASPI 13
VN ARU 469, PRU 2
ICA lt lat FU2
13.12.2021
523 days
100 mg ASS
OKM D

## Slide 55
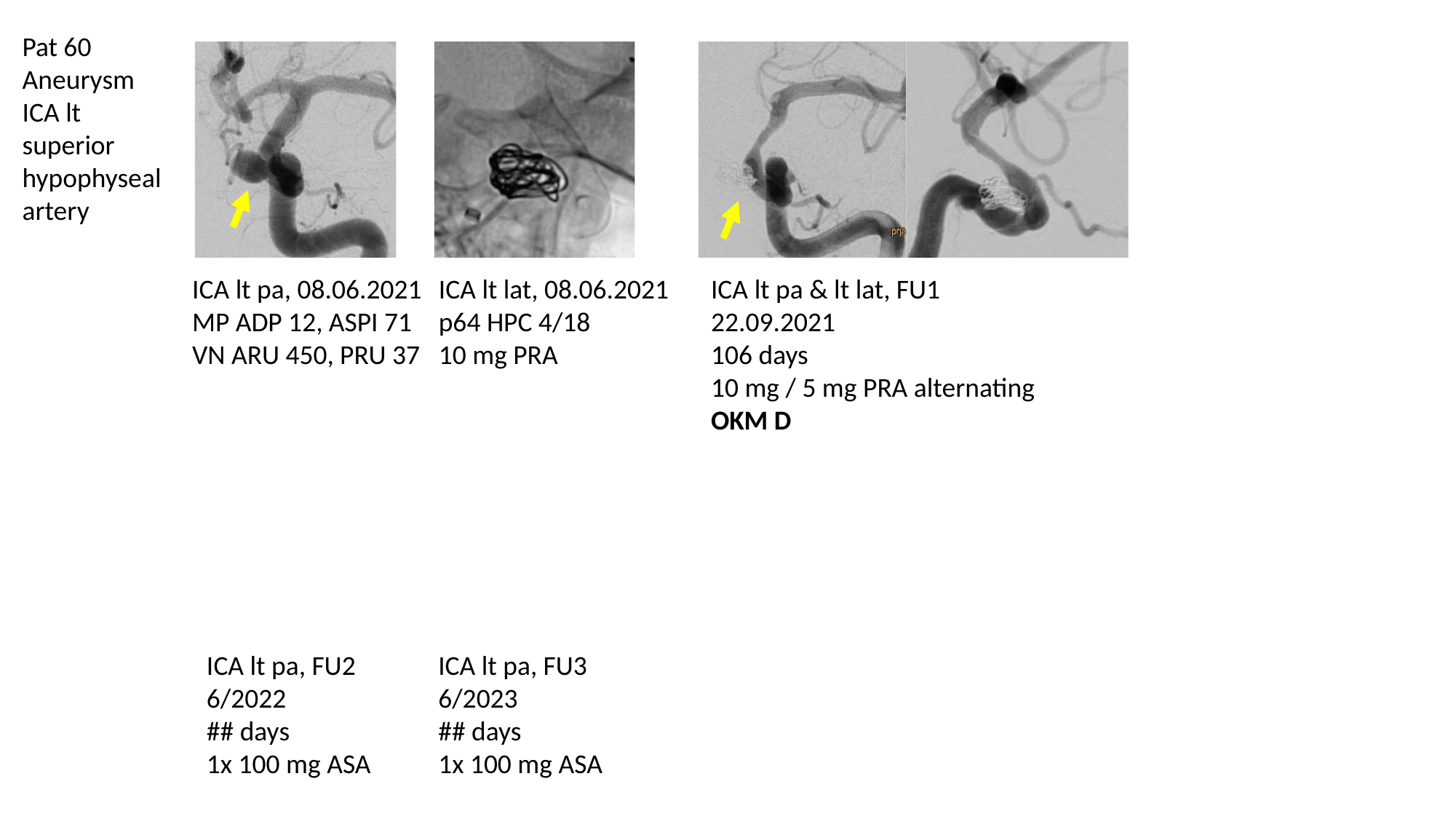

Pat 60
Aneurysm
ICA lt
superior
hypophyseal
artery
ICA lt lat, 08.06.2021
p64 HPC 4/18
10 mg PRA
ICA lt pa & lt lat, FU1
22.09.2021
106 days
10 mg / 5 mg PRA alternating
OKM D
ICA lt pa, 08.06.2021
MP ADP 12, ASPI 71
VN ARU 450, PRU 37
ICA lt pa, FU3
6/2023
## days
1x 100 mg ASA
ICA lt pa, FU2
6/2022
## days
1x 100 mg ASA

## Slide 56
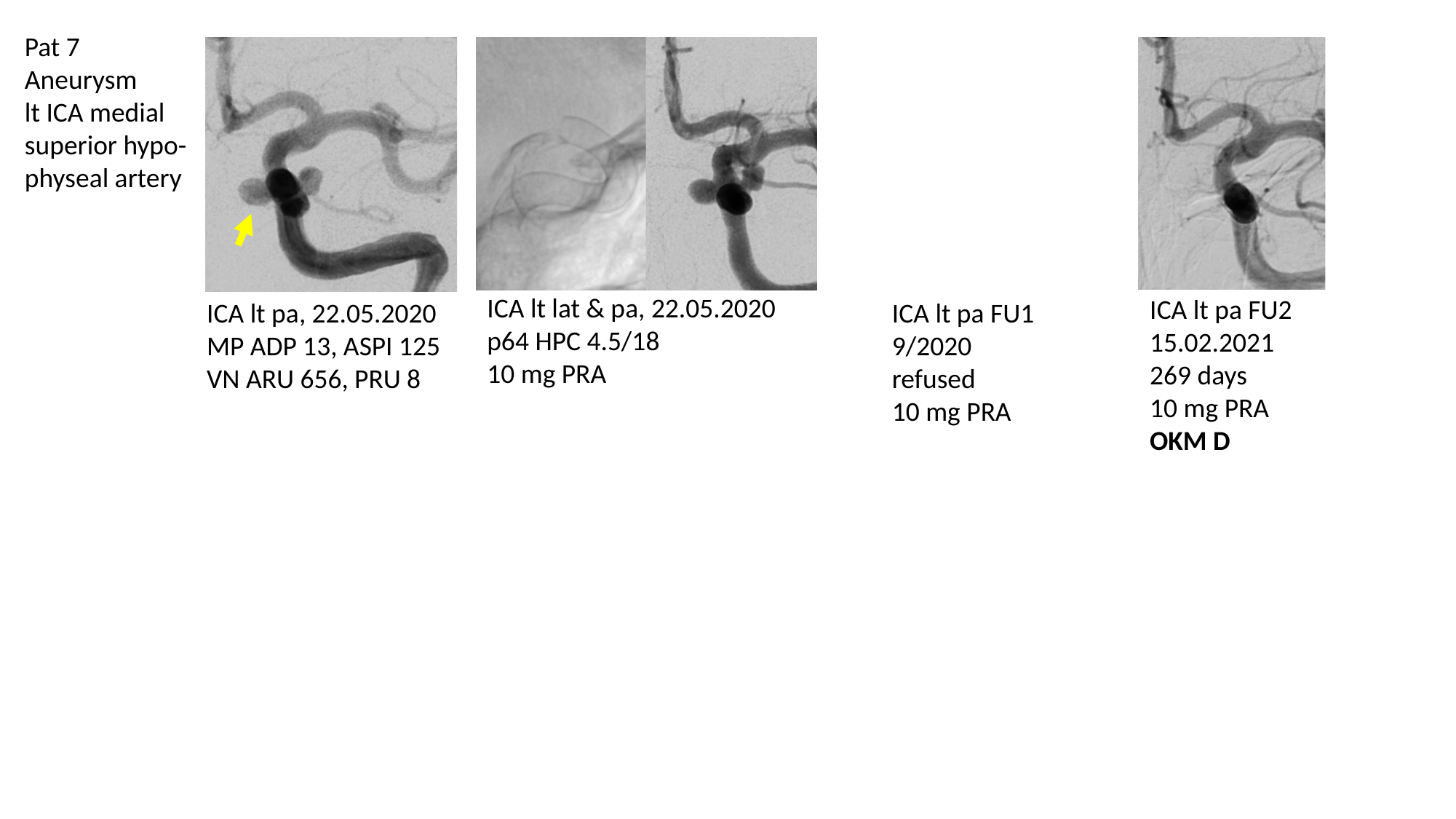

Pat 7
Aneurysm
lt ICA medial
superior hypo-
physeal artery
ICA lt lat & pa, 22.05.2020
p64 HPC 4.5/18
10 mg PRA
ICA lt pa FU2
15.02.2021
269 days
10 mg PRA
OKM D
ICA lt pa, 22.05.2020
MP ADP 13, ASPI 125
VN ARU 656, PRU 8
ICA lt pa FU1
9/2020
refused
10 mg PRA

## Slide 57
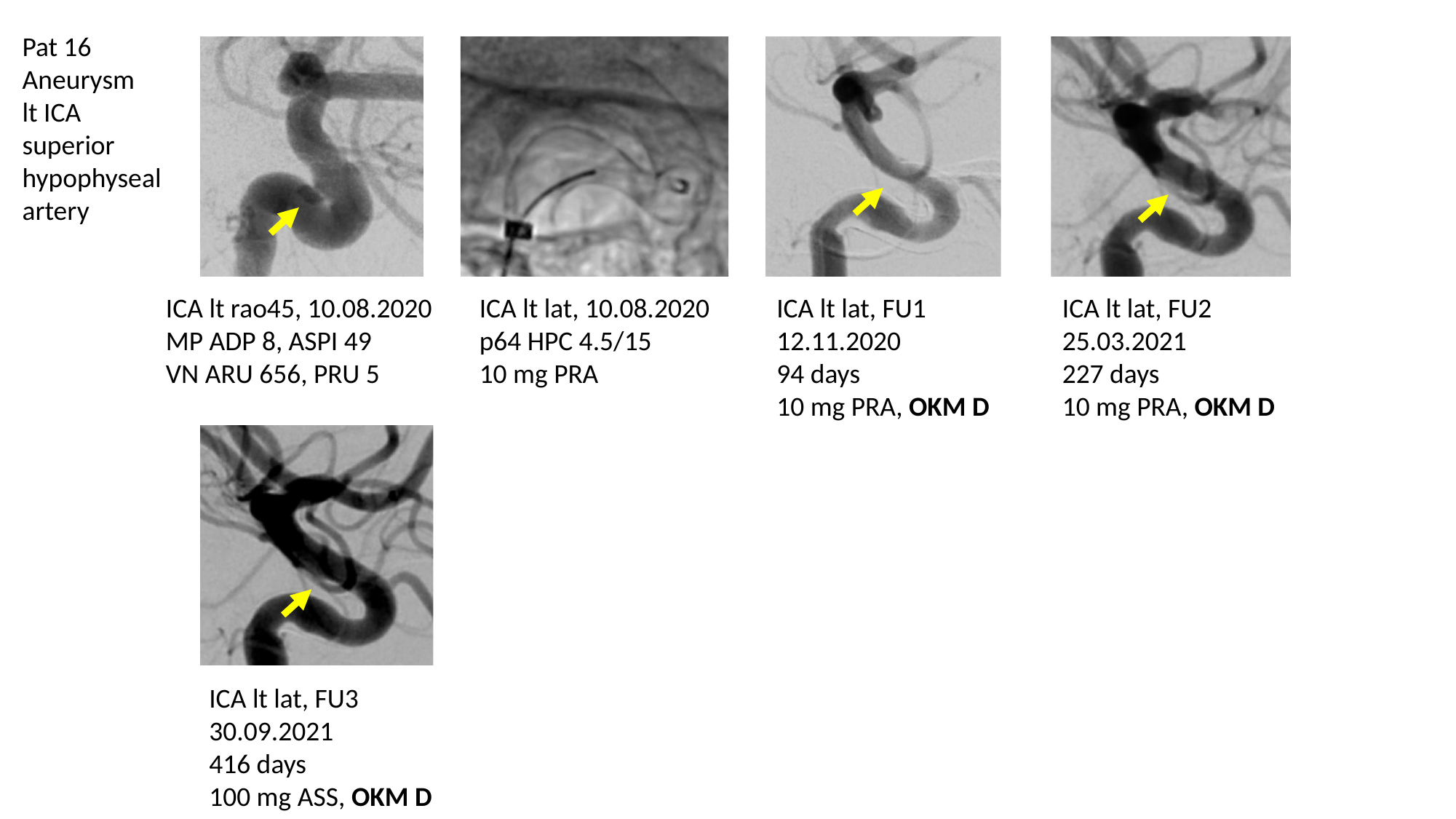

Pat 16
Aneurysm
lt ICA
superior
hypophyseal
artery
ICA lt rao45, 10.08.2020
MP ADP 8, ASPI 49
VN ARU 656, PRU 5
ICA lt lat, FU1
12.11.2020
94 days
10 mg PRA, OKM D
ICA lt lat, FU2
25.03.2021
227 days
10 mg PRA, OKM D
ICA lt lat, 10.08.2020
p64 HPC 4.5/15
10 mg PRA
ICA lt lat, FU3
30.09.2021
416 days
100 mg ASS, OKM D

## Slide 58
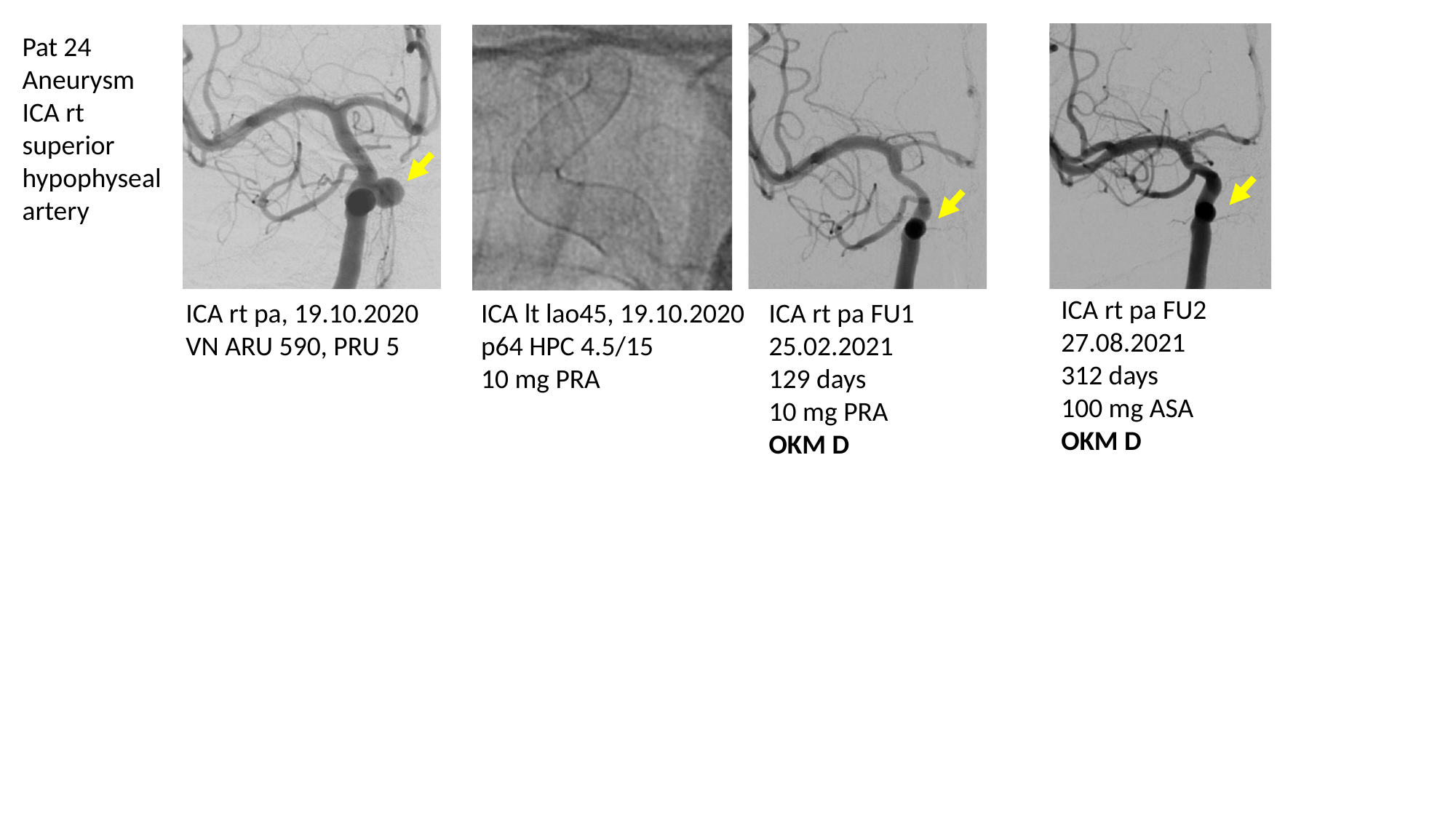

Pat 24
Aneurysm
ICA rt
superior
hypophyseal
artery
ICA rt pa FU2
27.08.2021
312 days
100 mg ASA
OKM D
ICA rt pa, 19.10.2020
VN ARU 590, PRU 5
ICA lt lao45, 19.10.2020
p64 HPC 4.5/15
10 mg PRA
ICA rt pa FU1
25.02.2021
129 days
10 mg PRA
OKM D

## Slide 59
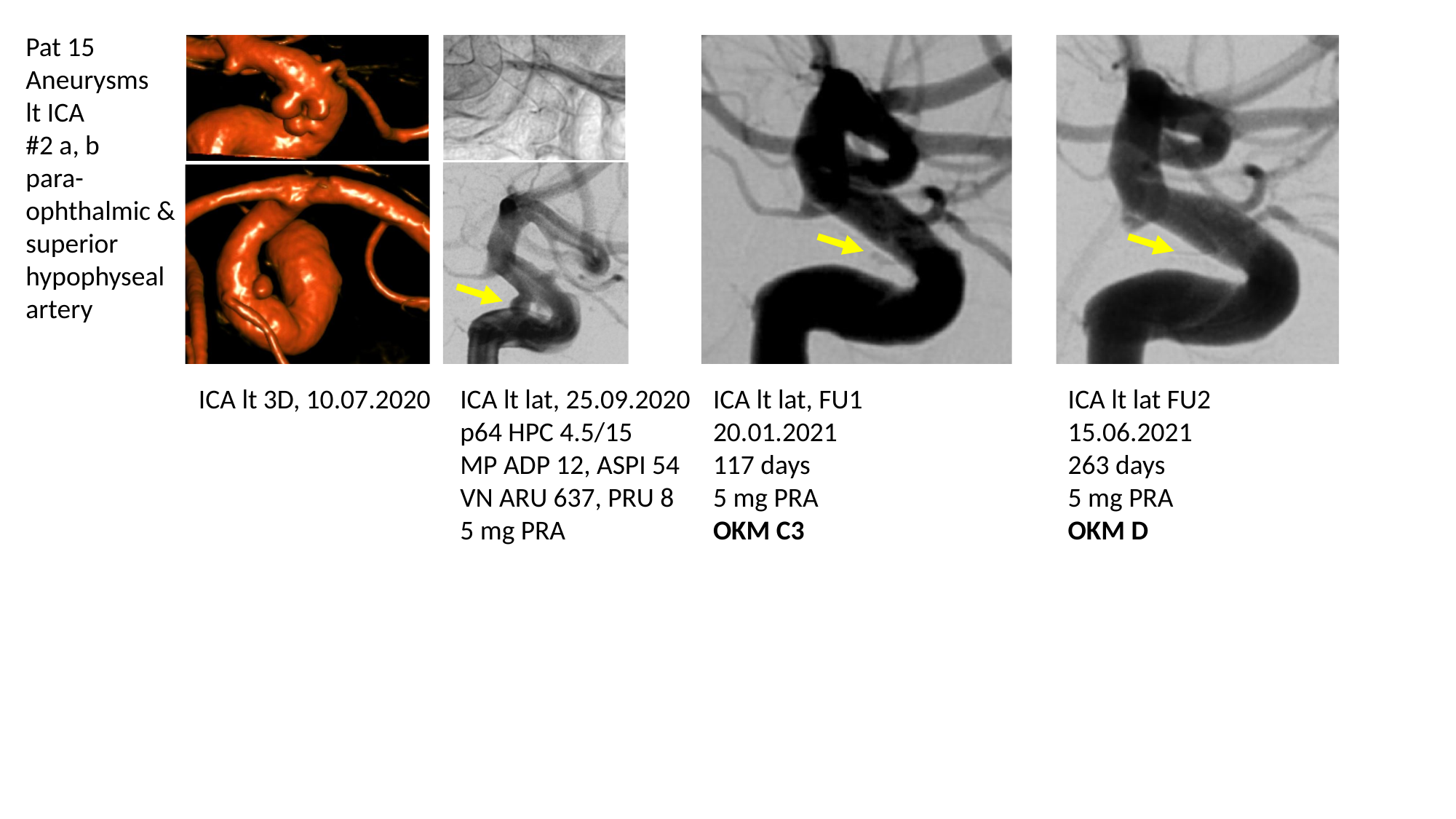

Pat 15
Aneurysms
lt ICA
#2 a, b
para-
ophthalmic &
superior
hypophyseal
artery
ICA lt lat FU2
15.06.2021
263 days
5 mg PRA
OKM D
ICA lt 3D, 10.07.2020
ICA lt lat, 25.09.2020
p64 HPC 4.5/15
MP ADP 12, ASPI 54
VN ARU 637, PRU 8
5 mg PRA
ICA lt lat, FU1
20.01.2021
117 days
5 mg PRA
OKM C3

## Slide 60
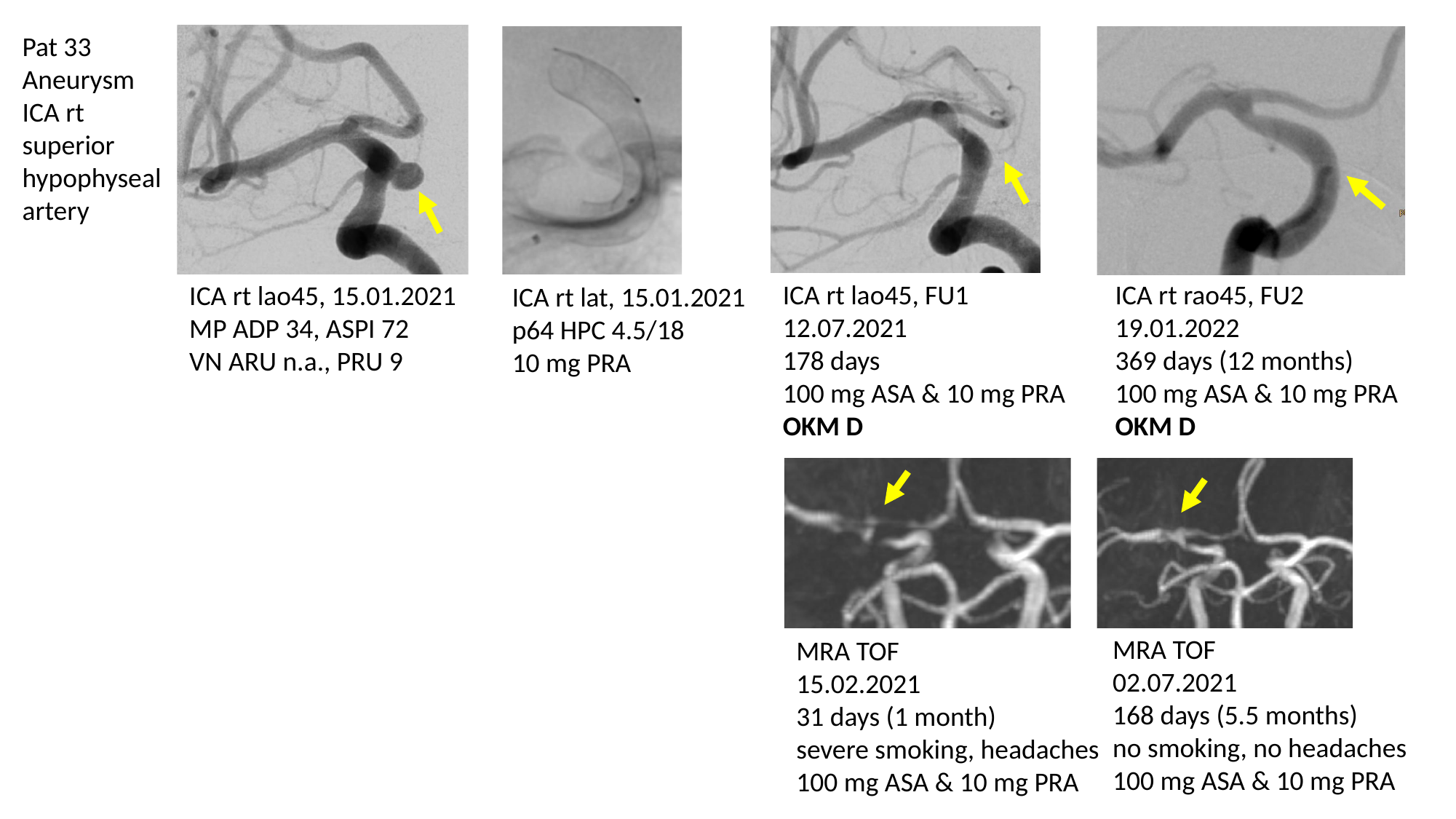

Pat 33
Aneurysm
ICA rt
superior
hypophyseal
artery
ICA rt lao45, FU1
12.07.2021
178 days
100 mg ASA & 10 mg PRA
OKM D
ICA rt rao45, FU2
19.01.2022
369 days (12 months)
100 mg ASA & 10 mg PRA
OKM D
ICA rt lao45, 15.01.2021
MP ADP 34, ASPI 72
VN ARU n.a., PRU 9
ICA rt lat, 15.01.2021
p64 HPC 4.5/18
10 mg PRA
MRA TOF
02.07.2021
168 days (5.5 months)
no smoking, no headaches
100 mg ASA & 10 mg PRA
MRA TOF
15.02.2021
31 days (1 month)
severe smoking, headaches
100 mg ASA & 10 mg PRA

## Slide 61
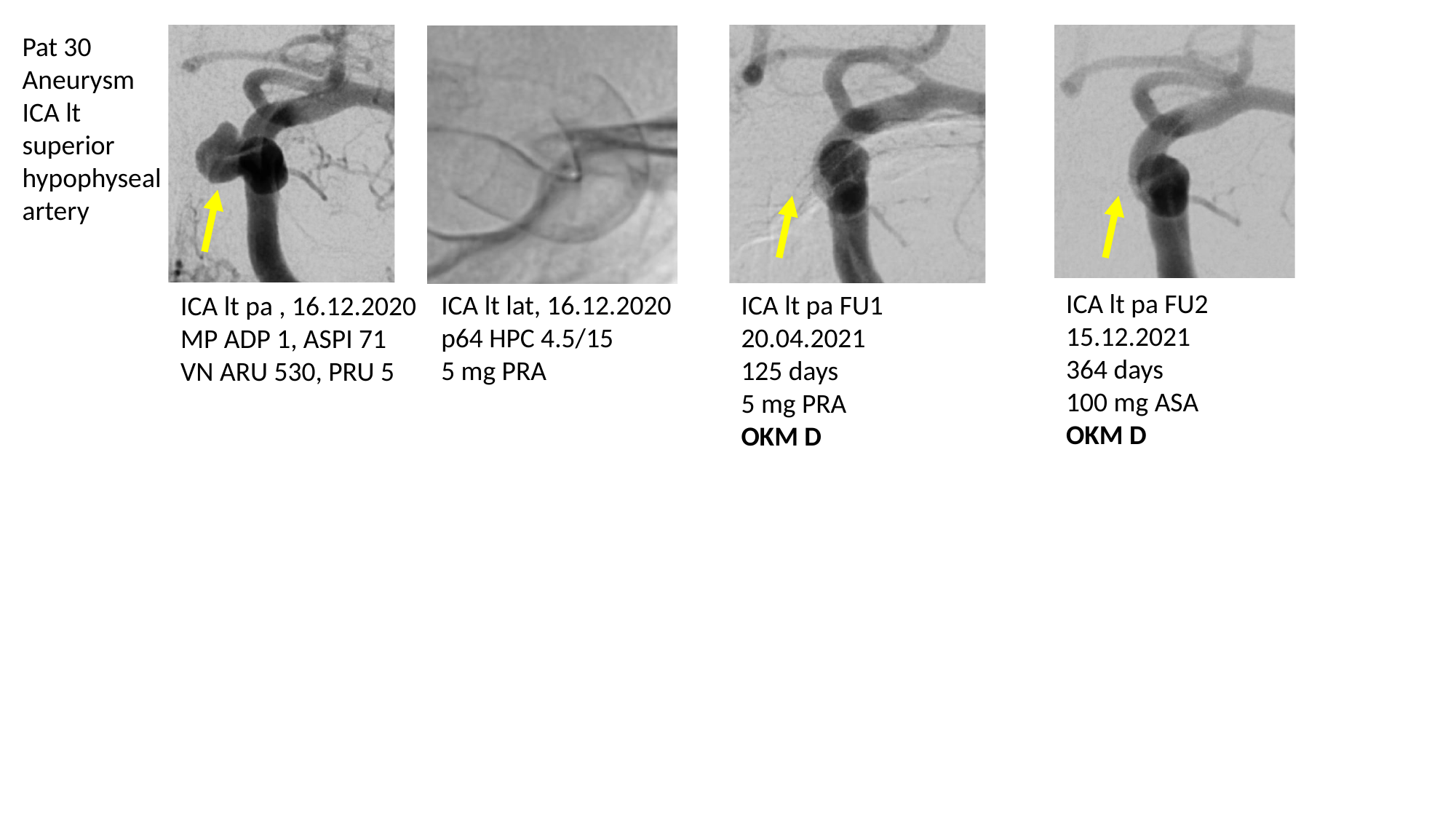

Pat 30
Aneurysm
ICA lt
superior
hypophyseal
artery
ICA lt pa FU2
15.12.2021
364 days
100 mg ASA
OKM D
ICA lt lat, 16.12.2020
p64 HPC 4.5/15
5 mg PRA
ICA lt pa FU1
20.04.2021
125 days
5 mg PRA
OKM D
ICA lt pa , 16.12.2020
MP ADP 1, ASPI 71
VN ARU 530, PRU 5

## Slide 62
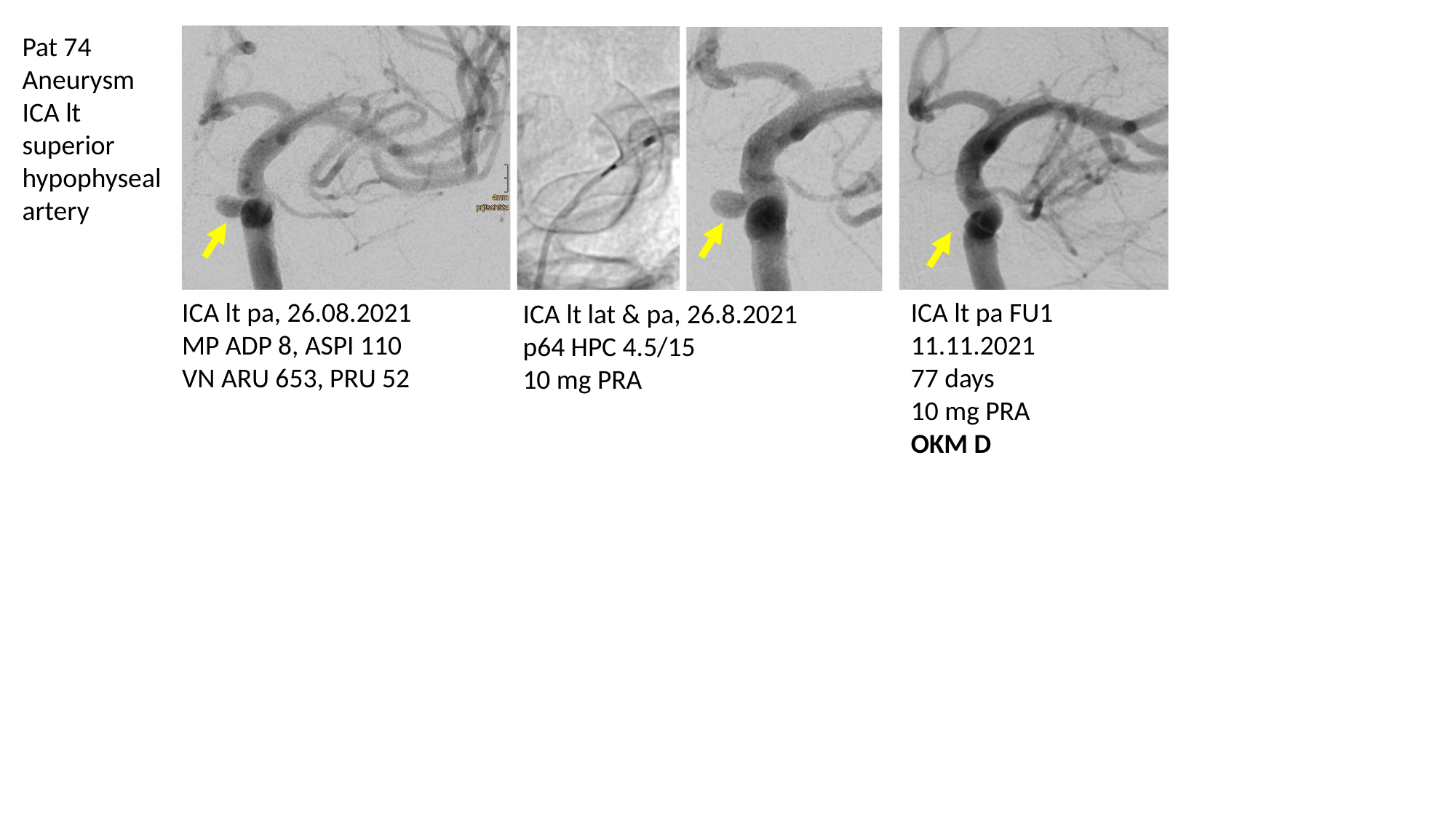

Pat 74
Aneurysm
ICA lt
superior
hypophyseal
artery
ICA lt pa FU1
11.11.2021
77 days
10 mg PRA
OKM D
ICA lt pa, 26.08.2021
MP ADP 8, ASPI 110
VN ARU 653, PRU 52
ICA lt lat & pa, 26.8.2021
p64 HPC 4.5/15
10 mg PRA

## Slide 63
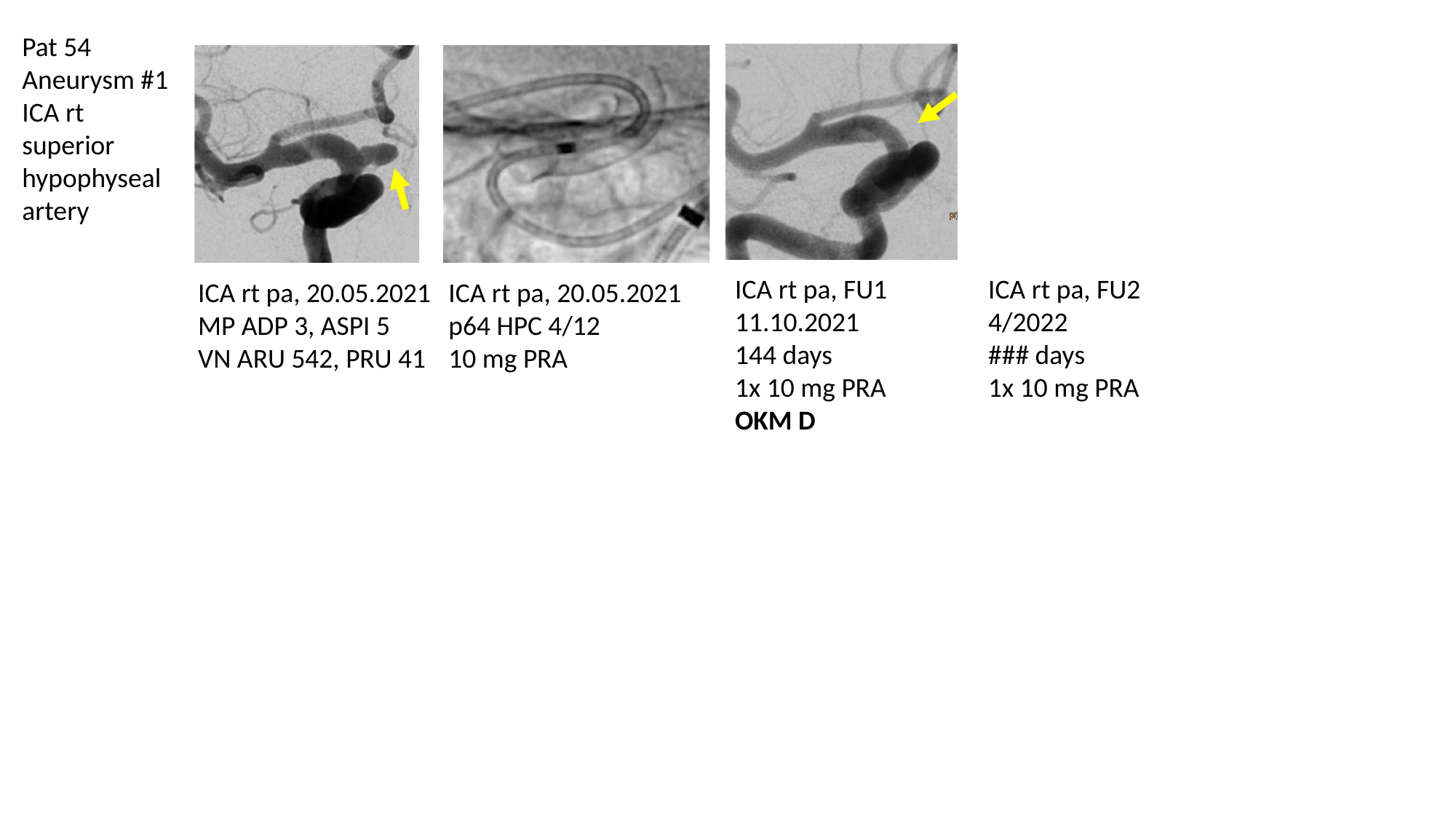

Pat 54
Aneurysm #1
ICA rt
superior
hypophyseal
artery
ICA rt pa, FU2
4/2022
### days
1x 10 mg PRA
ICA rt pa, FU1
11.10.2021
144 days
1x 10 mg PRA
OKM D
ICA rt pa, 20.05.2021
MP ADP 3, ASPI 5
VN ARU 542, PRU 41
ICA rt pa, 20.05.2021
p64 HPC 4/12
10 mg PRA

## Slide 64
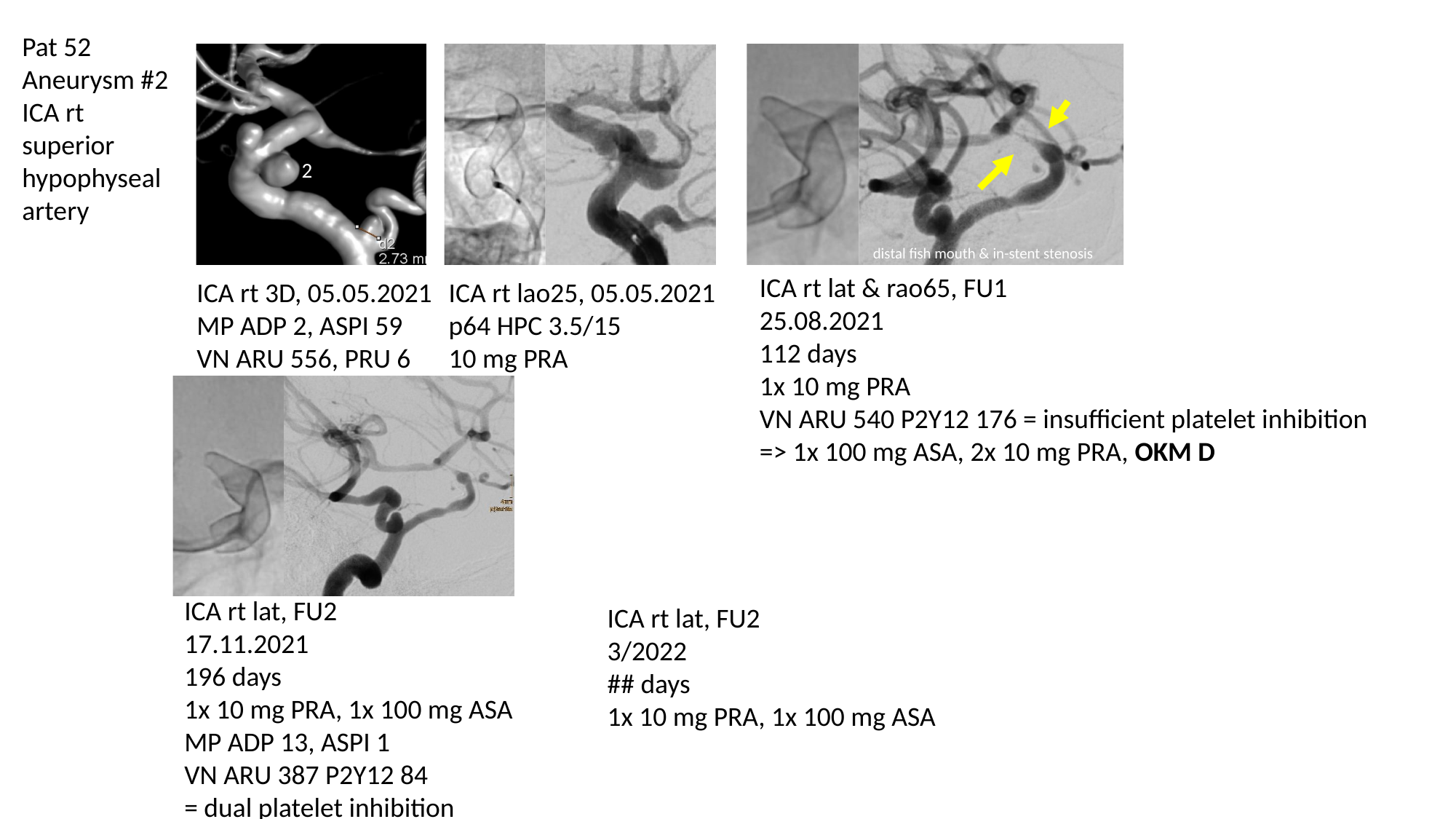

Pat 52
Aneurysm #2
ICA rt
superior
hypophyseal
artery
2
distal fish mouth & in-stent stenosis
ICA rt lat & rao65, FU1
25.08.2021
112 days
1x 10 mg PRA
VN ARU 540 P2Y12 176 = insufficient platelet inhibition
=> 1x 100 mg ASA, 2x 10 mg PRA, OKM D
ICA rt 3D, 05.05.2021
MP ADP 2, ASPI 59
VN ARU 556, PRU 6
ICA rt lao25, 05.05.2021
p64 HPC 3.5/15
10 mg PRA
ICA rt lat, FU2
17.11.2021
196 days
1x 10 mg PRA, 1x 100 mg ASA
MP ADP 13, ASPI 1
VN ARU 387 P2Y12 84
= dual platelet inhibition
ICA rt lat, FU2
3/2022
## days
1x 10 mg PRA, 1x 100 mg ASA

## Slide 65
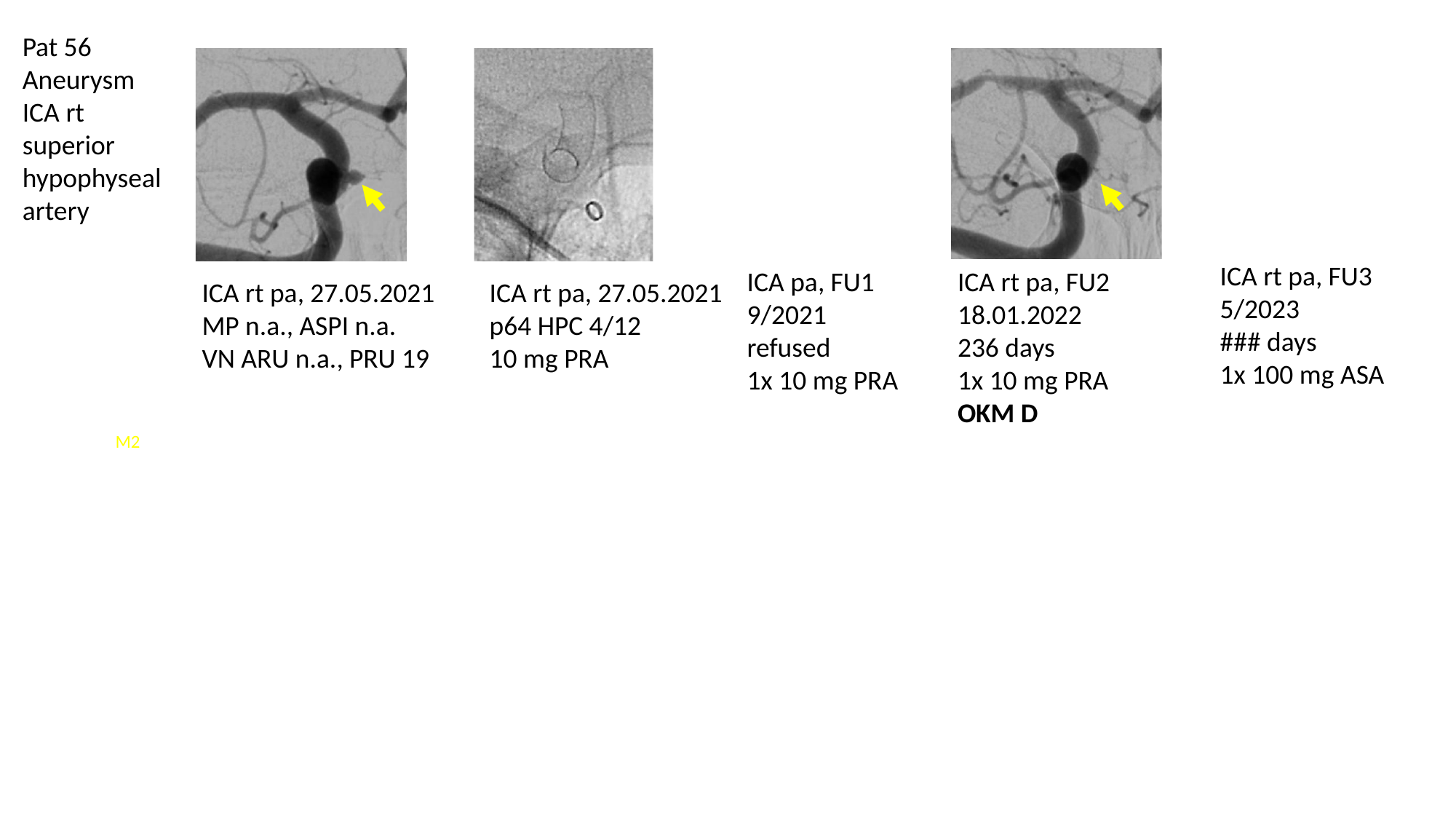

Pat 56
Aneurysm
ICA rt
superior
hypophyseal
artery
ICA rt pa, FU3
5/2023
### days
1x 100 mg ASA
ICA pa, FU1
9/2021
refused
1x 10 mg PRA
ICA rt pa, FU2
18.01.2022
236 days
1x 10 mg PRA
OKM D
ICA rt pa, 27.05.2021
MP n.a., ASPI n.a.
VN ARU n.a., PRU 19
ICA rt pa, 27.05.2021
p64 HPC 4/12
10 mg PRA
M2

## Slide 66
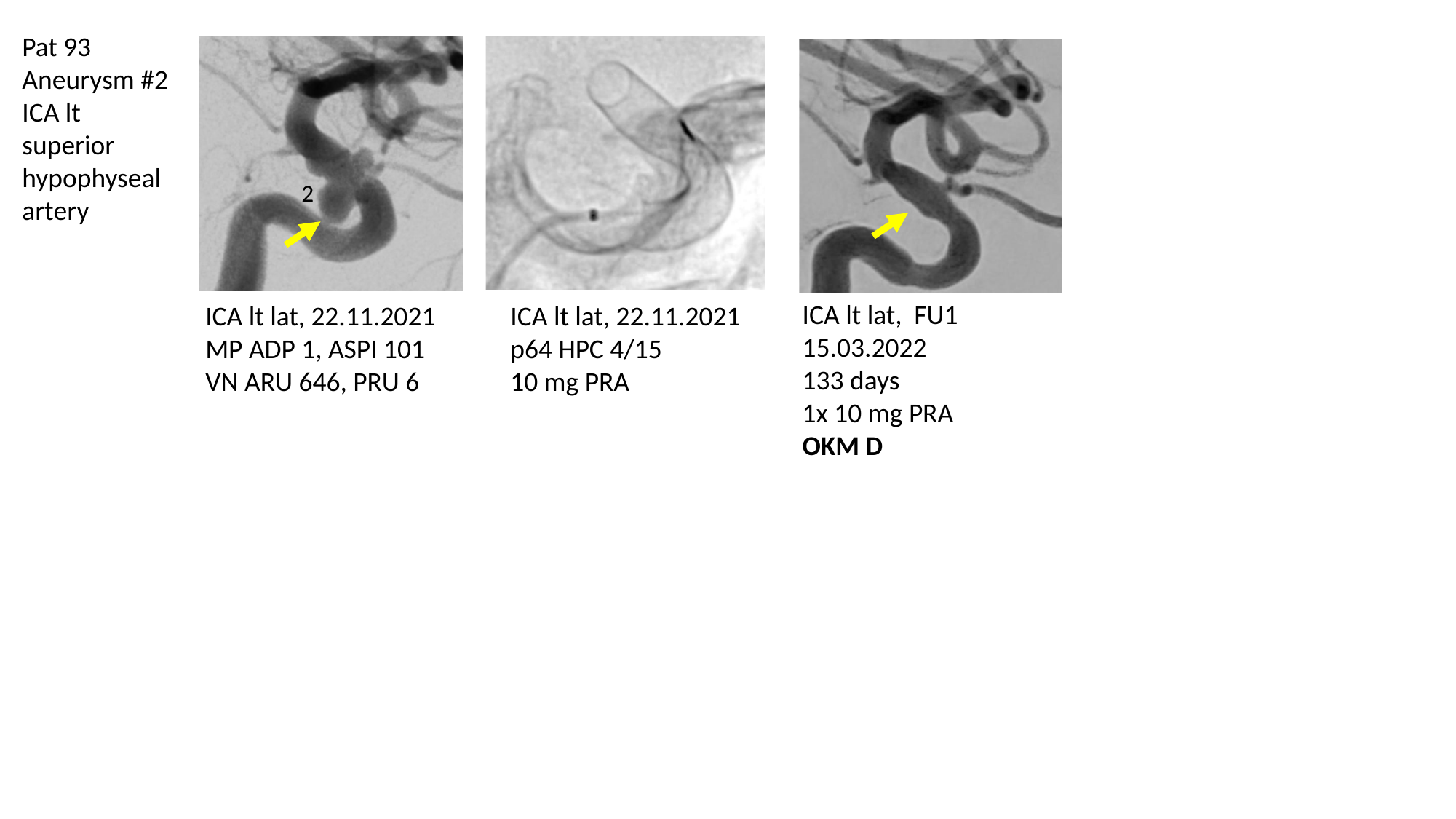

Pat 93
Aneurysm #2
ICA lt
superior
hypophyseal
artery
2
ICA lt lat, FU1
15.03.2022
133 days
1x 10 mg PRA
OKM D
ICA lt lat, 22.11.2021
p64 HPC 4/15
10 mg PRA
ICA lt lat, 22.11.2021
MP ADP 1, ASPI 101
VN ARU 646, PRU 6

## Slide 67
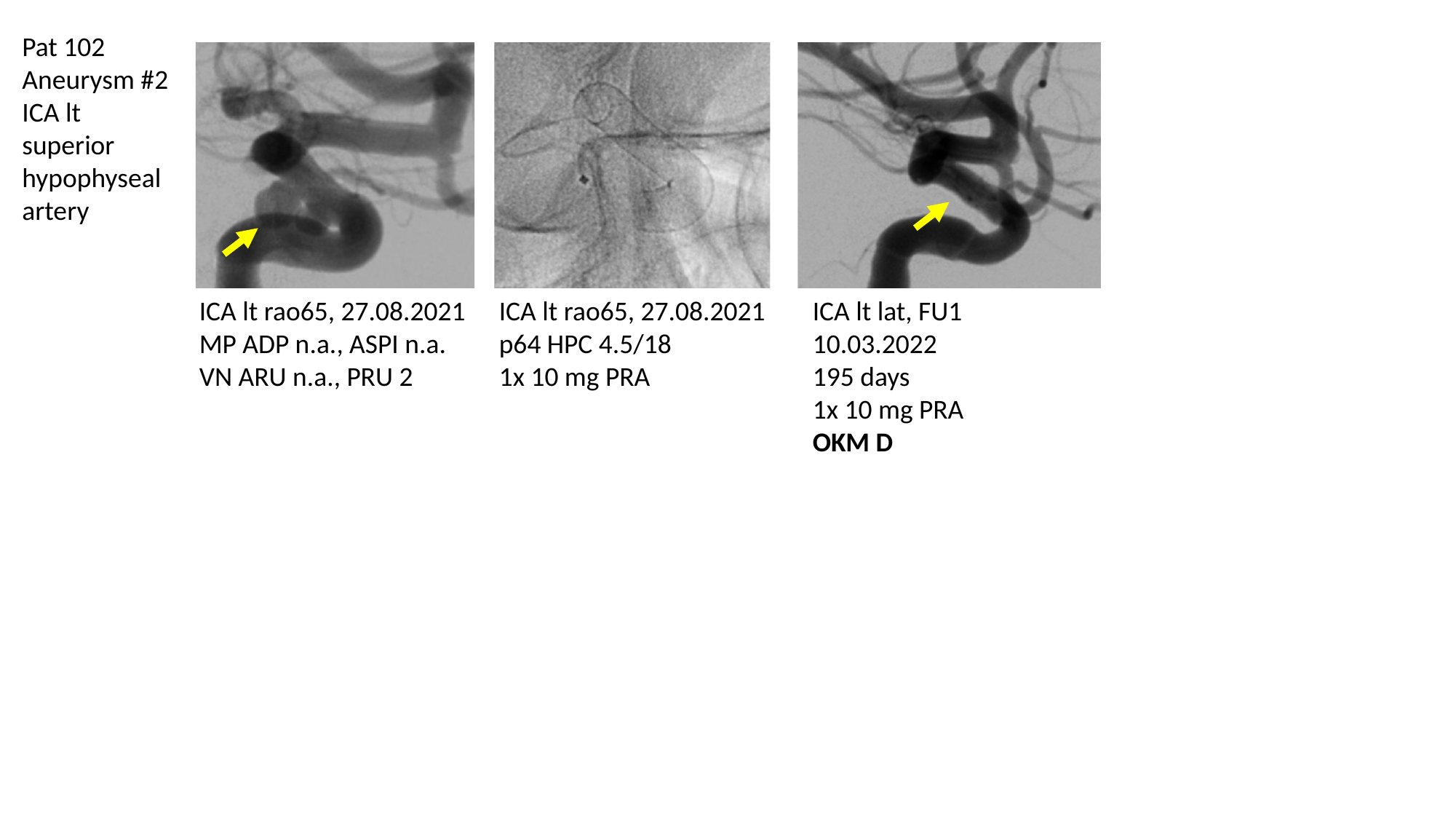

Pat 102
Aneurysm #2
ICA lt
superior
hypophyseal
artery
ICA lt rao65, 27.08.2021
MP ADP n.a., ASPI n.a.
VN ARU n.a., PRU 2
ICA lt rao65, 27.08.2021
p64 HPC 4.5/18
1x 10 mg PRA
ICA lt lat, FU1
10.03.2022
195 days
1x 10 mg PRA
OKM D

## Slide 68
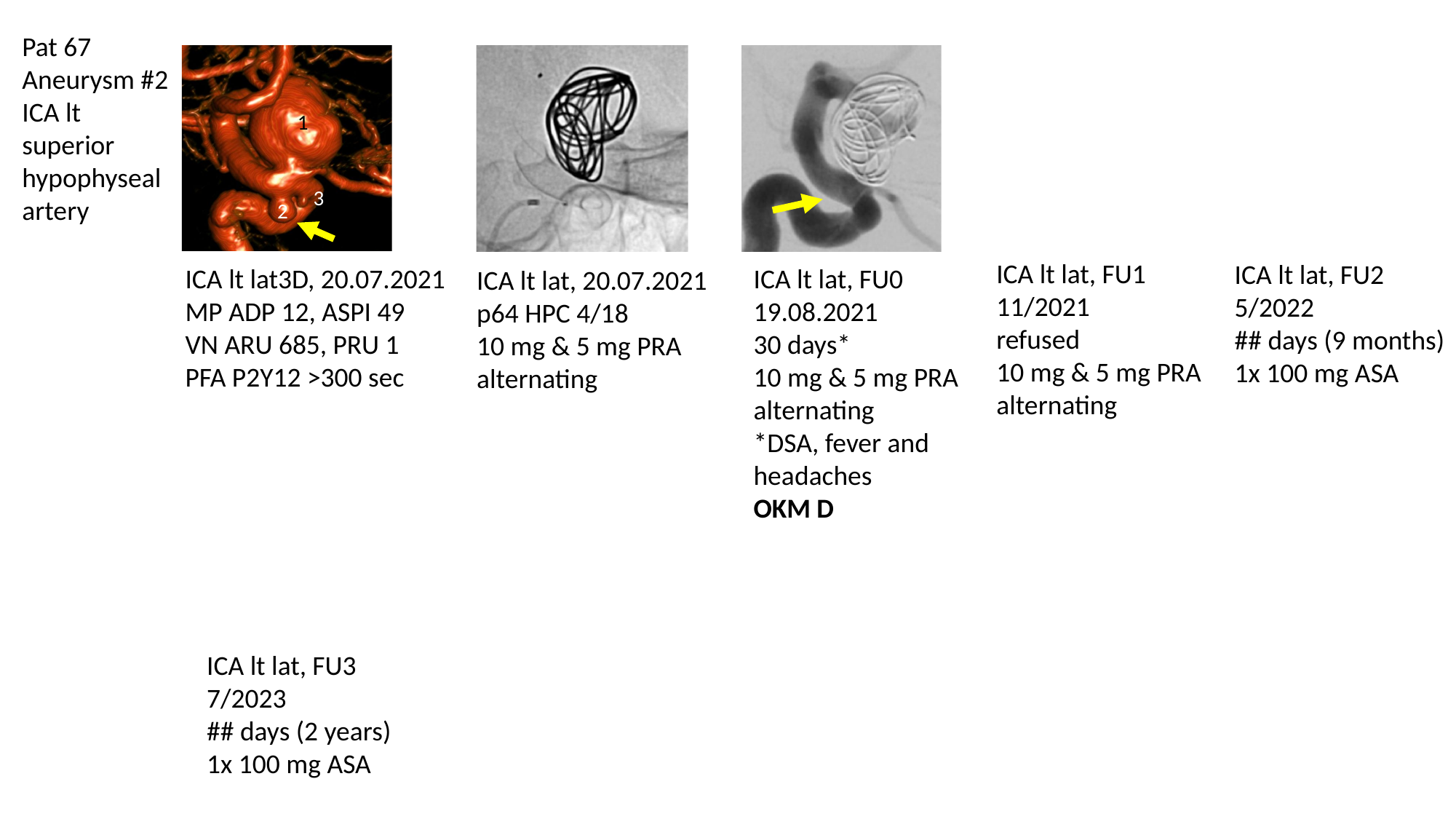

Pat 67
Aneurysm #2
ICA lt
superior
hypophyseal
artery
1
3
2
ICA lt lat, FU1
11/2021
refused
10 mg & 5 mg PRA
alternating
ICA lt lat, FU2
5/2022
## days (9 months)
1x 100 mg ASA
ICA lt lat3D, 20.07.2021
MP ADP 12, ASPI 49
VN ARU 685, PRU 1
PFA P2Y12 >300 sec
ICA lt lat, FU0
19.08.2021
30 days*
10 mg & 5 mg PRA
alternating
*DSA, fever and
headaches
OKM D
ICA lt lat, 20.07.2021
p64 HPC 4/18
10 mg & 5 mg PRA
alternating
ICA lt lat, FU3
7/2023
## days (2 years)
1x 100 mg ASA

## Slide 69
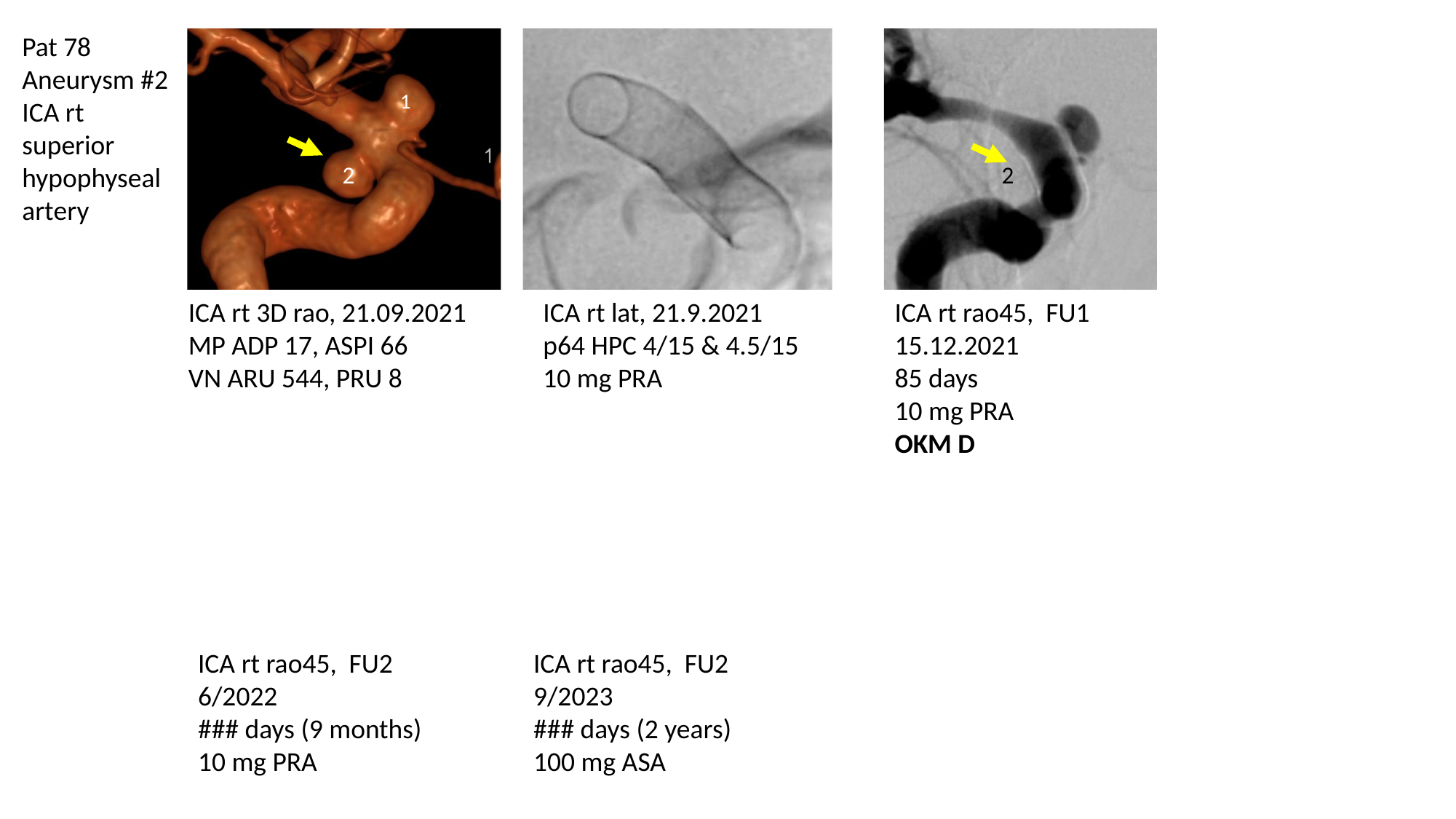

Pat 78
Aneurysm #2
ICA rt
superior
hypophyseal
artery
1
2
2
2
1
ICA rt rao45, FU1
15.12.2021
85 days
10 mg PRA
OKM D
ICA rt 3D rao, 21.09.2021
MP ADP 17, ASPI 66
VN ARU 544, PRU 8
ICA rt lat, 21.9.2021
p64 HPC 4/15 & 4.5/15
10 mg PRA
ICA rt rao45, FU2
9/2023
### days (2 years)
100 mg ASA
ICA rt rao45, FU2
6/2022
### days (9 months)
10 mg PRA

## Slide 70
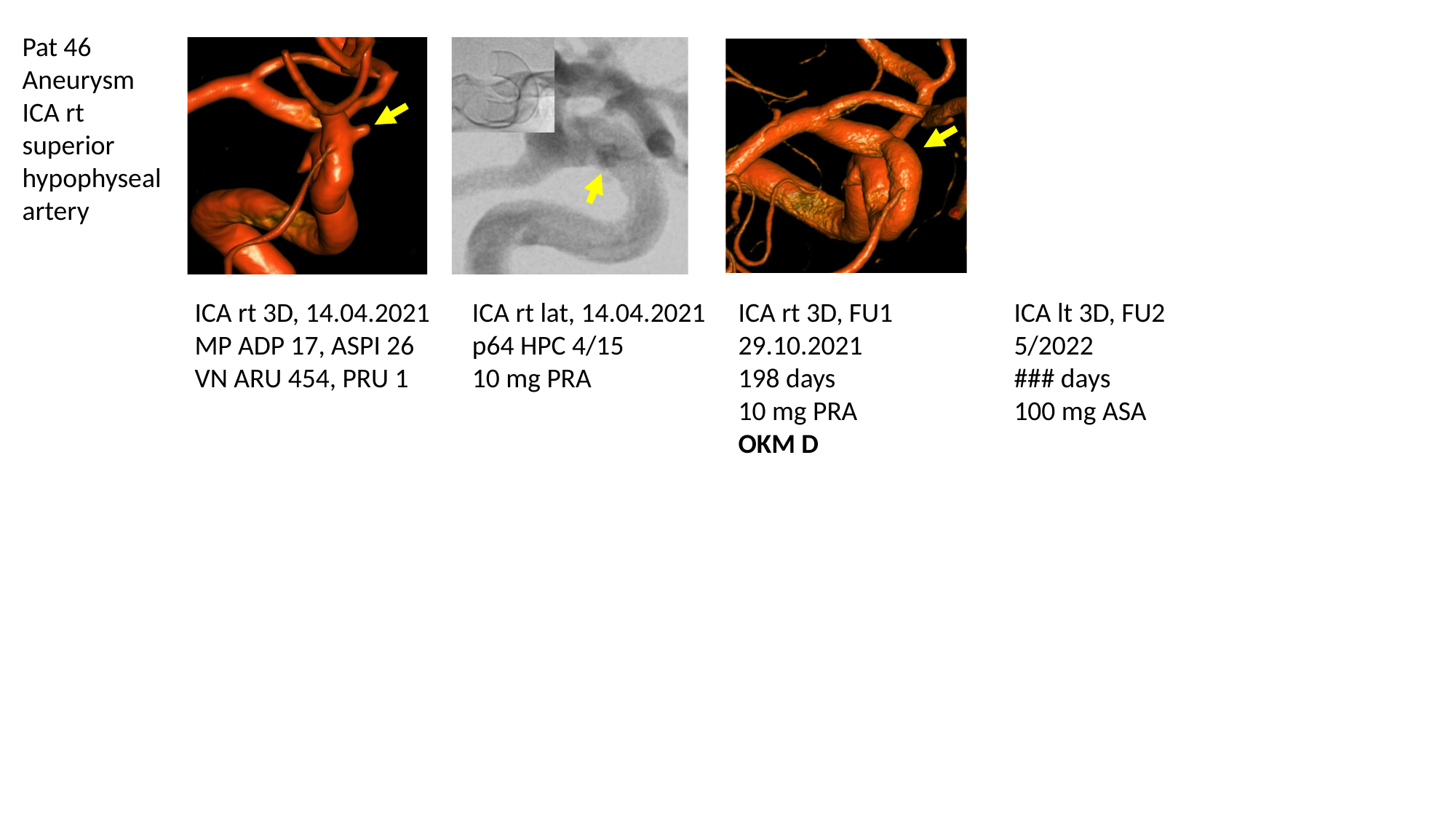

Pat 46
Aneurysm
ICA rt
superior
hypophyseal
artery
ICA rt 3D, 14.04.2021
MP ADP 17, ASPI 26
VN ARU 454, PRU 1
ICA rt lat, 14.04.2021
p64 HPC 4/15
10 mg PRA
ICA rt 3D, FU1
29.10.2021
198 days
10 mg PRA
OKM D
ICA lt 3D, FU2
5/2022
### days
100 mg ASA

## Slide 71
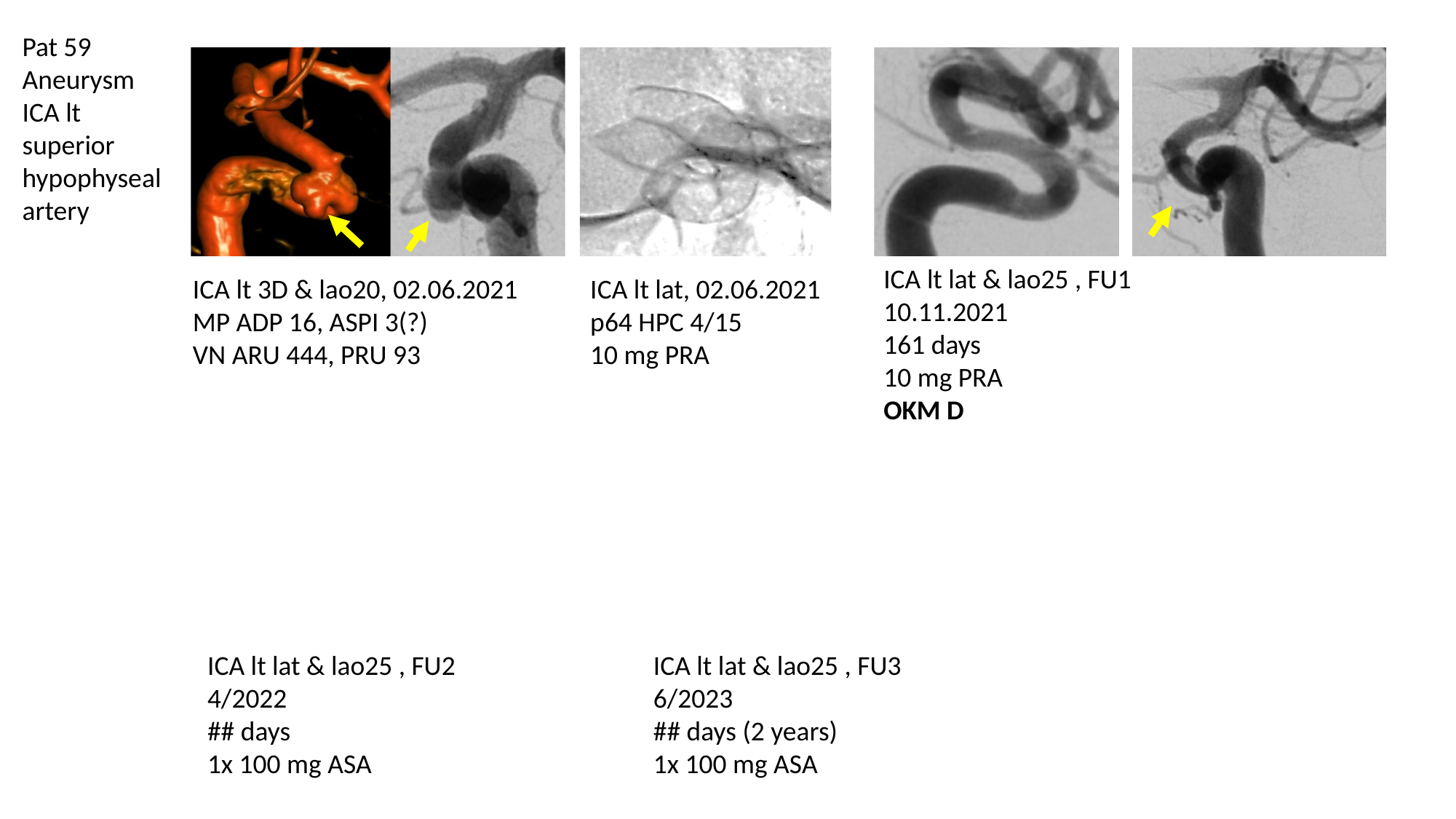

Pat 59
Aneurysm
ICA lt
superior
hypophyseal
artery
ICA lt lat & lao25 , FU1
10.11.2021
161 days
10 mg PRA
OKM D
ICA lt 3D & lao20, 02.06.2021
MP ADP 16, ASPI 3(?)
VN ARU 444, PRU 93
ICA lt lat, 02.06.2021
p64 HPC 4/15
10 mg PRA
ICA lt lat & lao25 , FU3
6/2023
## days (2 years)
1x 100 mg ASA
ICA lt lat & lao25 , FU2
4/2022
## days
1x 100 mg ASA

## Slide 72
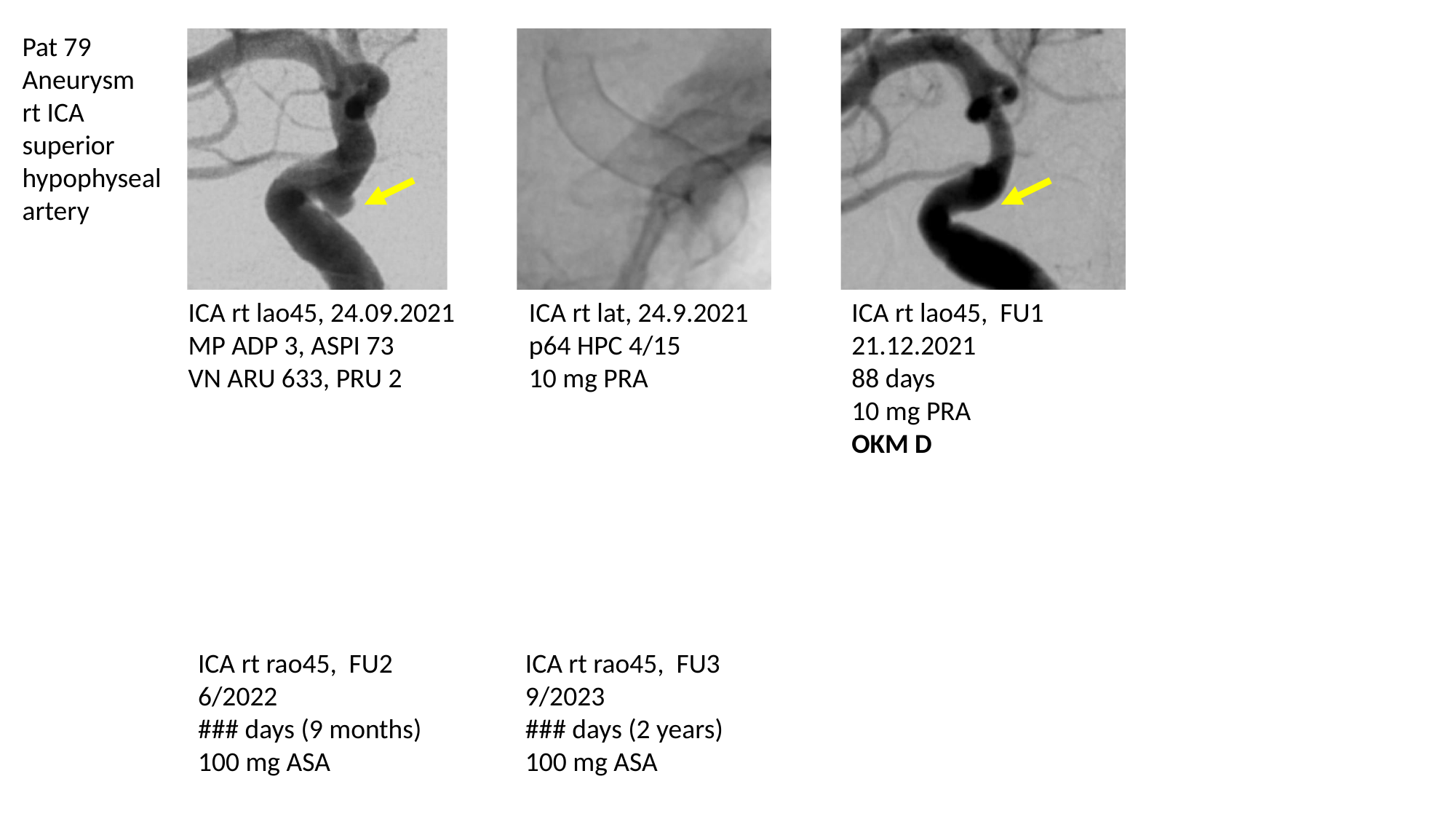

Pat 79
Aneurysm
rt ICA
superior
hypophyseal
artery
1
1
ICA rt lat, 24.9.2021
p64 HPC 4/15
10 mg PRA
ICA rt lao45, FU1
21.12.2021
88 days
10 mg PRA
OKM D
ICA rt lao45, 24.09.2021
MP ADP 3, ASPI 73
VN ARU 633, PRU 2
ICA rt rao45, FU3
9/2023
### days (2 years)
100 mg ASA
ICA rt rao45, FU2
6/2022
### days (9 months)
100 mg ASA

## Slide 73
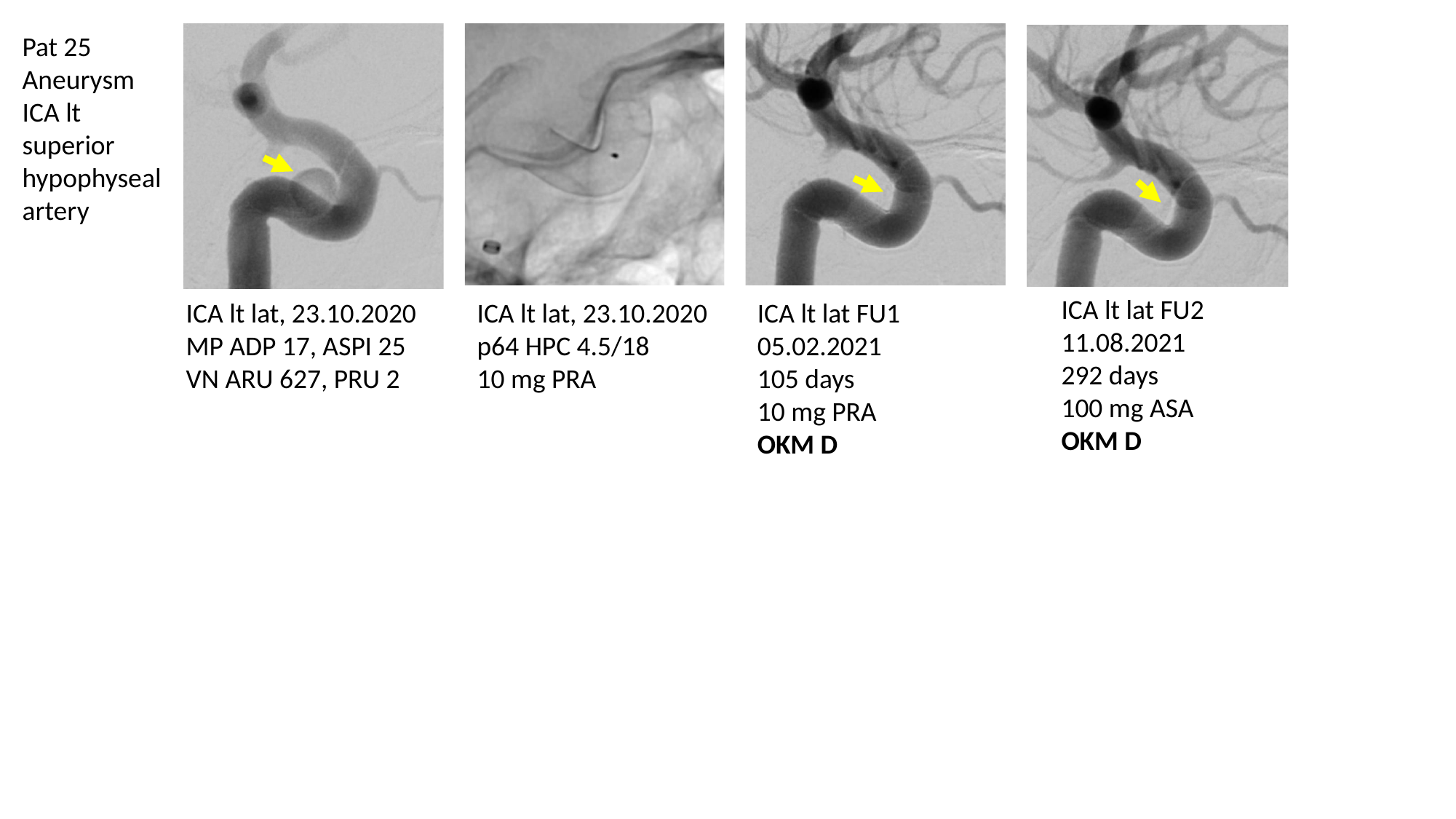

Pat 25
Aneurysm
ICA lt
superior
hypophyseal
artery
ICA lt lat FU2
11.08.2021
292 days
100 mg ASA
OKM D
ICA lt lat, 23.10.2020
MP ADP 17, ASPI 25
VN ARU 627, PRU 2
ICA lt lat, 23.10.2020
p64 HPC 4.5/18
10 mg PRA
ICA lt lat FU1
05.02.2021
105 days
10 mg PRA
OKM D

## Slide 74
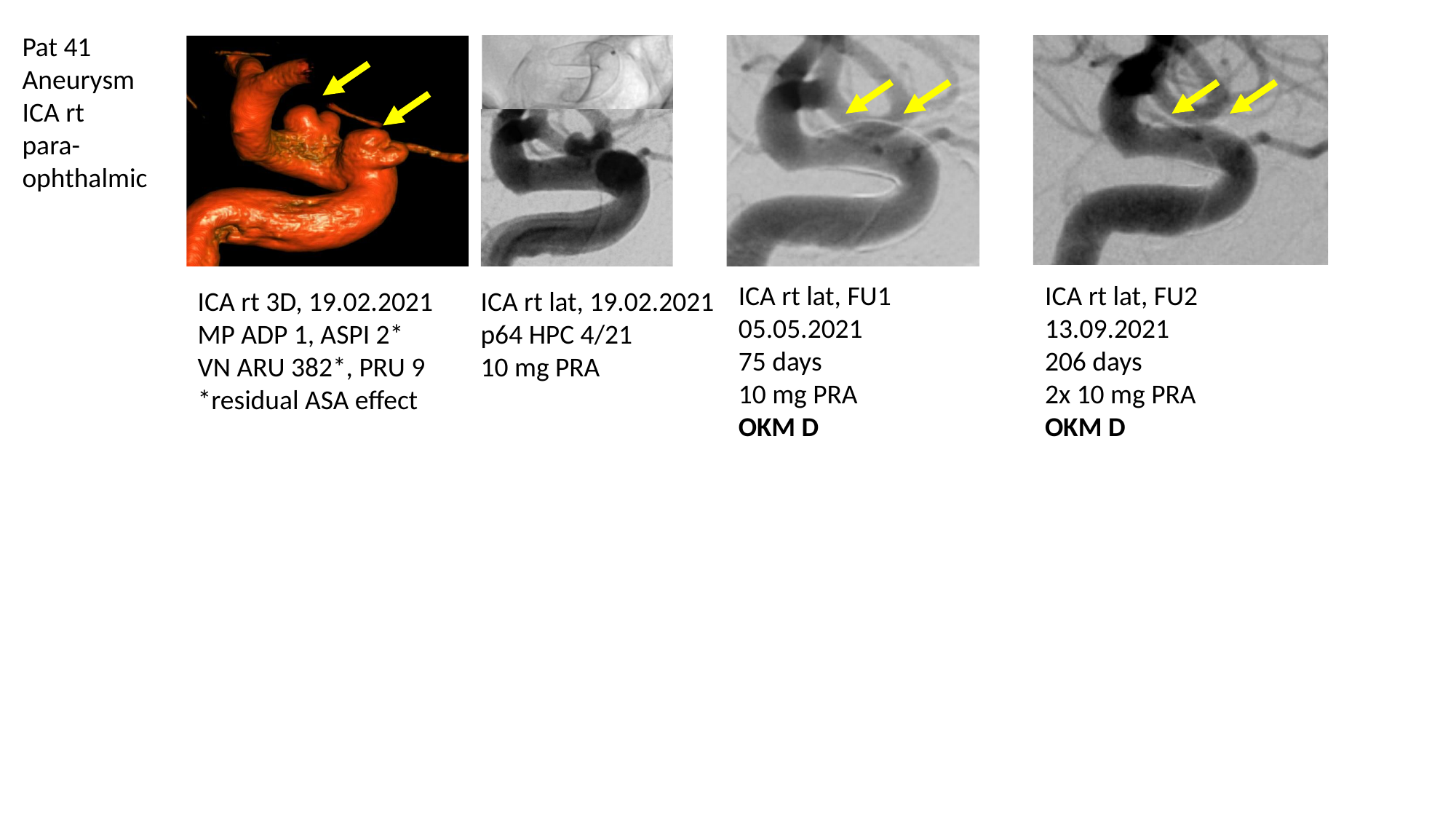

Pat 41
Aneurysm
ICA rt
para-
ophthalmic
ICA rt lat, FU1
05.05.2021
75 days
10 mg PRA
OKM D
ICA rt lat, FU2
13.09.2021
206 days
2x 10 mg PRA
OKM D
ICA rt 3D, 19.02.2021
MP ADP 1, ASPI 2*
VN ARU 382*, PRU 9
*residual ASA effect
ICA rt lat, 19.02.2021
p64 HPC 4/21
10 mg PRA

## Slide 75
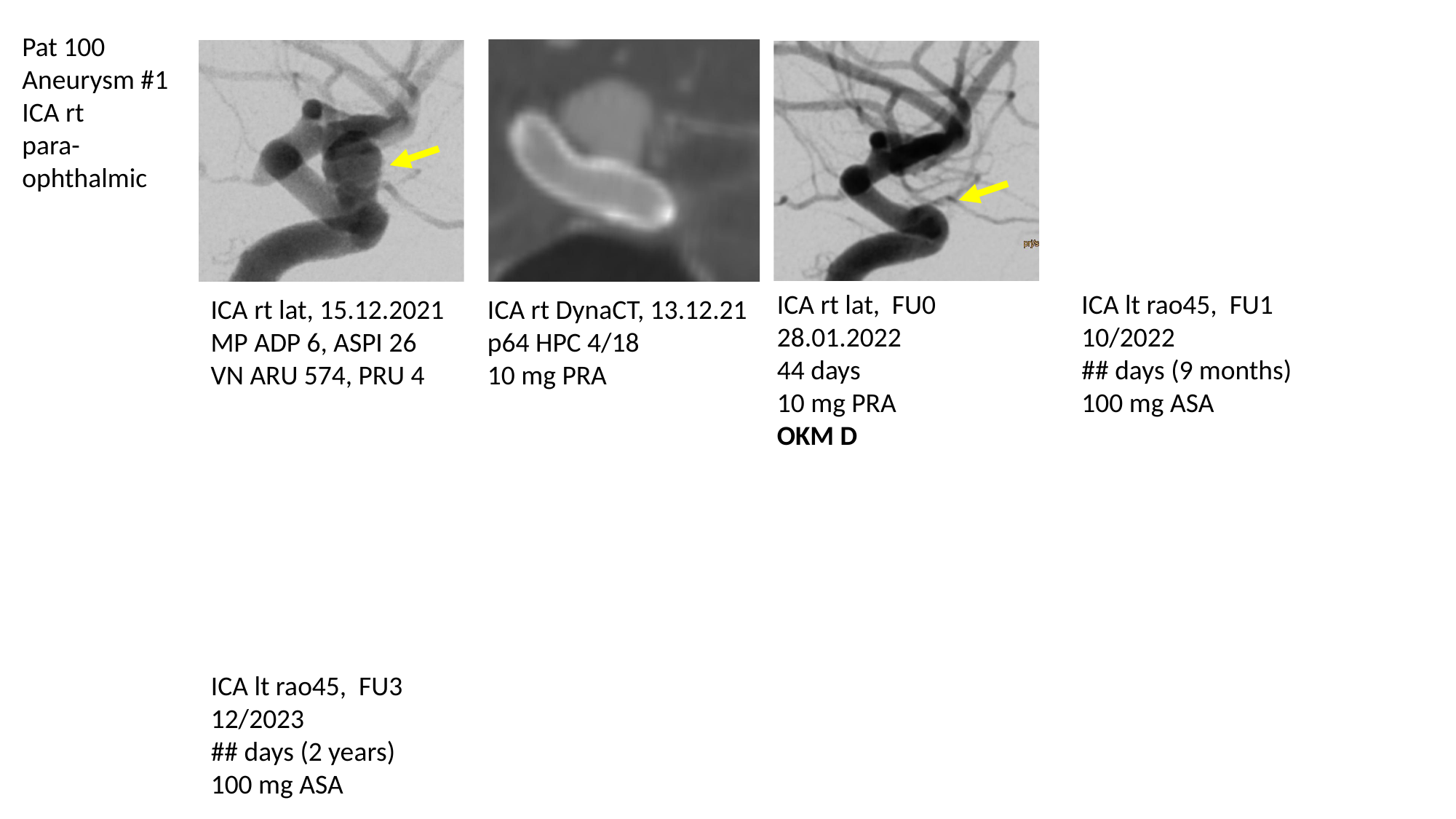

Pat 100
Aneurysm #1
ICA rt
para-
ophthalmic
ICA lt rao45, FU1
10/2022
## days (9 months)
100 mg ASA
ICA rt lat, FU0
28.01.2022
44 days
10 mg PRA
OKM D
ICA rt lat, 15.12.2021
MP ADP 6, ASPI 26
VN ARU 574, PRU 4
ICA rt DynaCT, 13.12.21
p64 HPC 4/18
10 mg PRA
ICA lt rao45, FU3
12/2023
## days (2 years)
100 mg ASA

## Slide 76
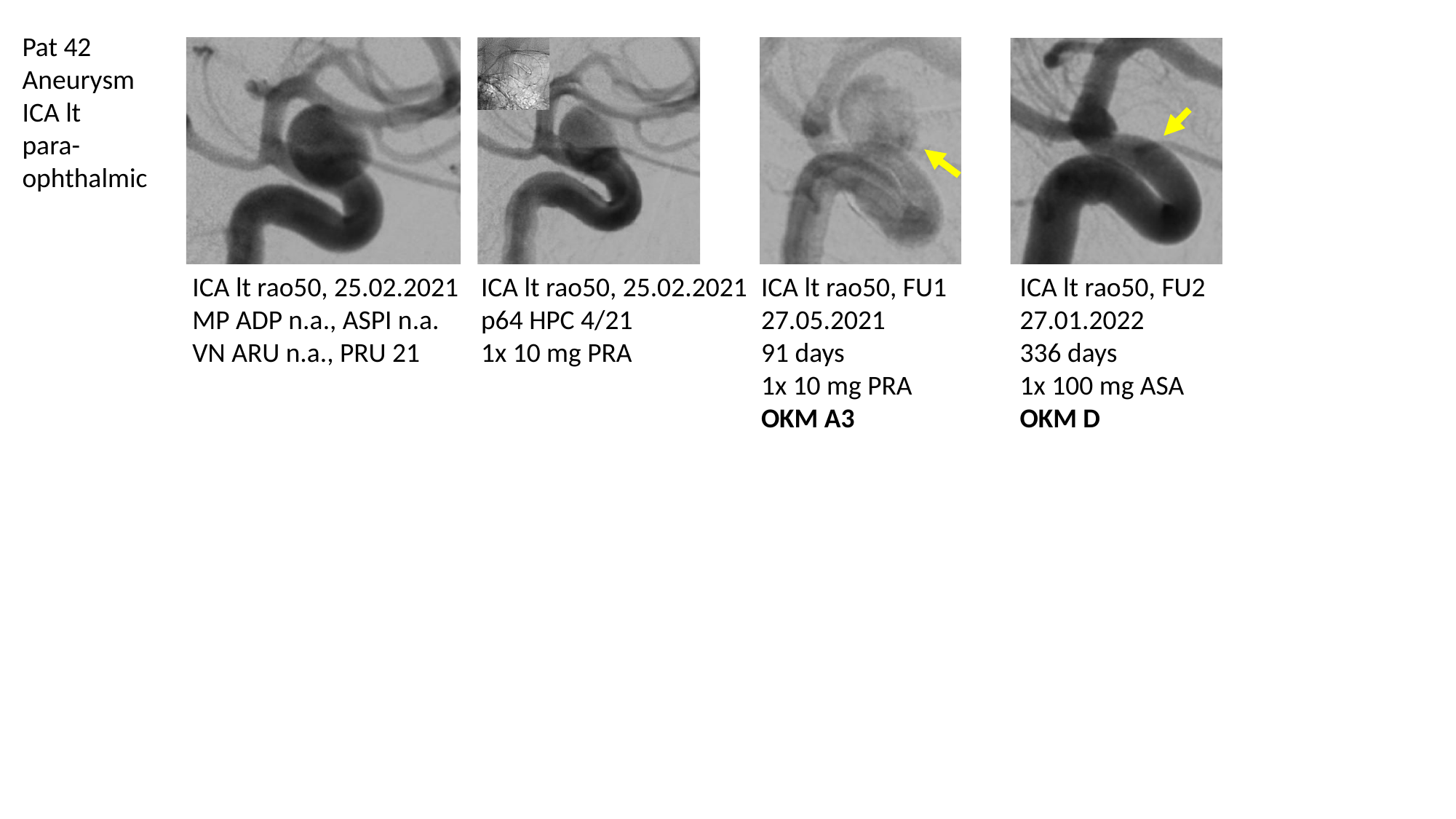

Pat 42
Aneurysm
ICA lt
para-
ophthalmic
ICA lt rao50, FU2
27.01.2022
336 days
1x 100 mg ASA
OKM D
ICA lt rao50, 25.02.2021
MP ADP n.a., ASPI n.a.
VN ARU n.a., PRU 21
ICA lt rao50, 25.02.2021
p64 HPC 4/21
1x 10 mg PRA
ICA lt rao50, FU1
27.05.2021
91 days
1x 10 mg PRA
OKM A3

## Slide 77
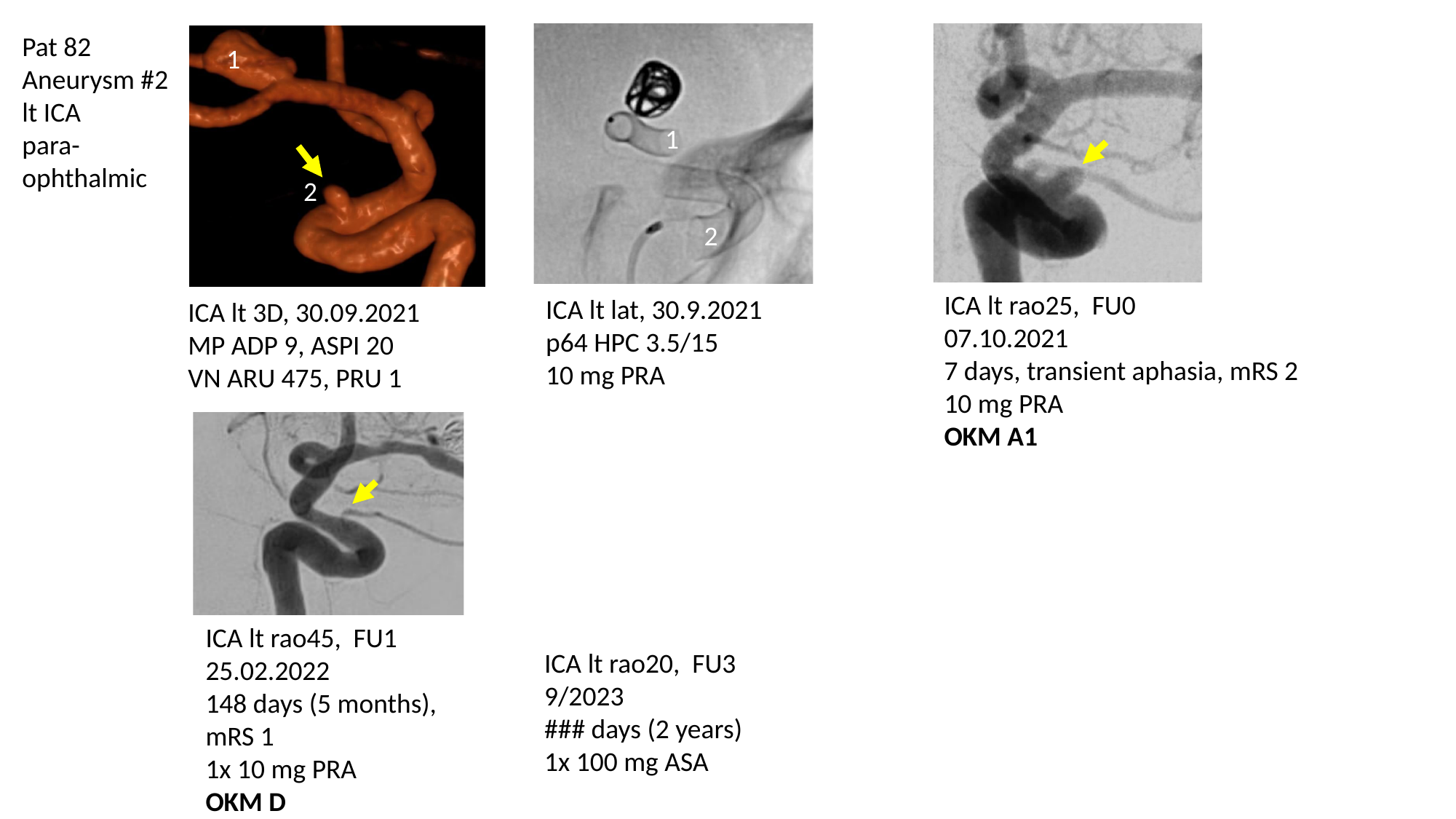

Pat 82
Aneurysm #2
lt ICA
para-
ophthalmic
1
M2
1
2
M1
2
ICA lt rao25, FU0
07.10.2021
7 days, transient aphasia, mRS 2
10 mg PRA
OKM A1
ICA lt lat, 30.9.2021
p64 HPC 3.5/15
10 mg PRA
ICA lt 3D, 30.09.2021
MP ADP 9, ASPI 20
VN ARU 475, PRU 1
ICA lt rao45, FU1
25.02.2022
148 days (5 months), mRS 1
1x 10 mg PRA
OKM D
ICA lt rao20, FU3
9/2023
### days (2 years)
1x 100 mg ASA

## Slide 78
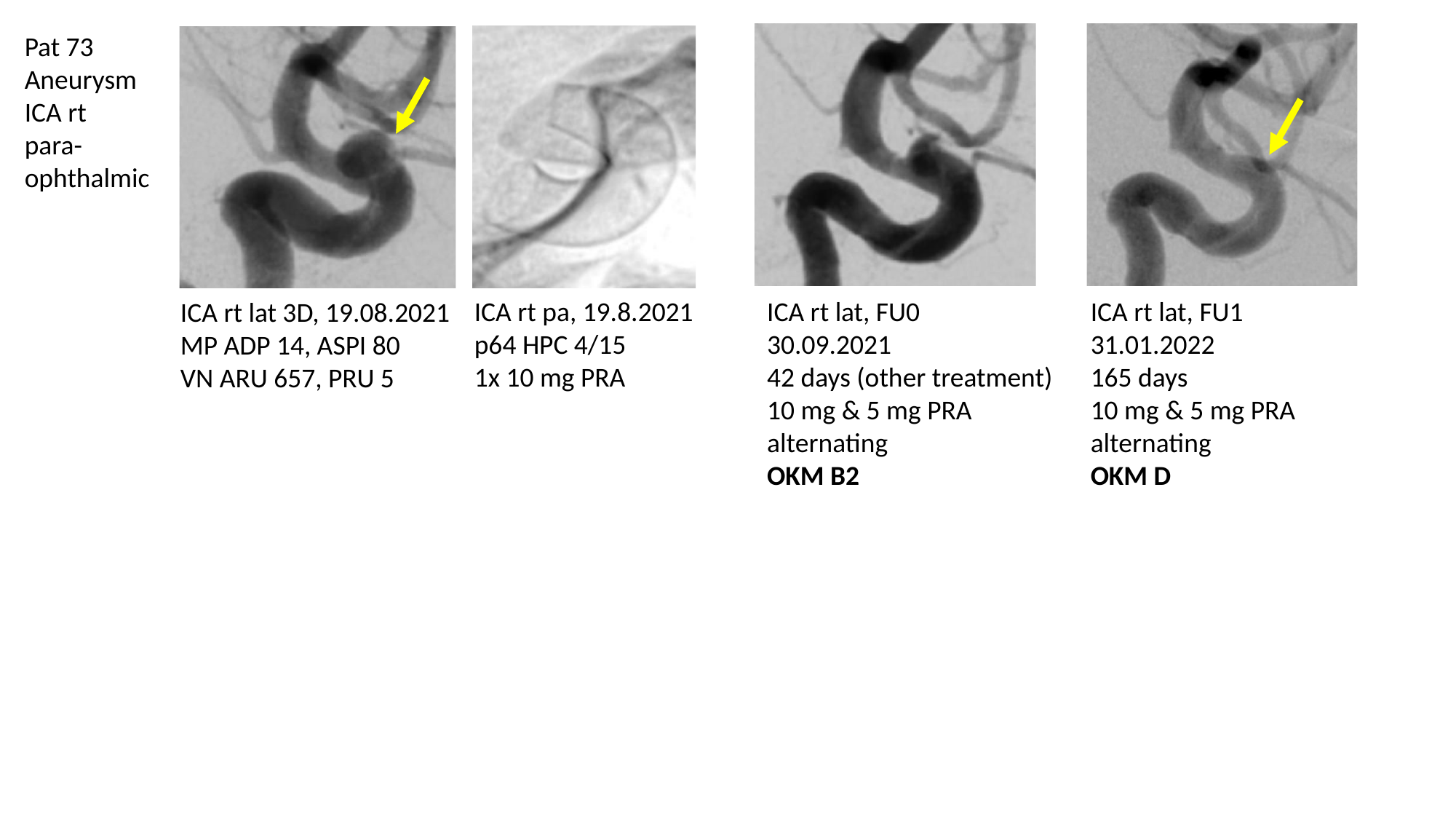

Pat 73
Aneurysm
ICA rt
para-
ophthalmic
ICA rt lat, FU0
30.09.2021
42 days (other treatment)
10 mg & 5 mg PRA
alternating
OKM B2
ICA rt pa, 19.8.2021
p64 HPC 4/15
1x 10 mg PRA
ICA rt lat, FU1
31.01.2022
165 days
10 mg & 5 mg PRA
alternating
OKM D
ICA rt lat 3D, 19.08.2021
MP ADP 14, ASPI 80
VN ARU 657, PRU 5

## Slide 79
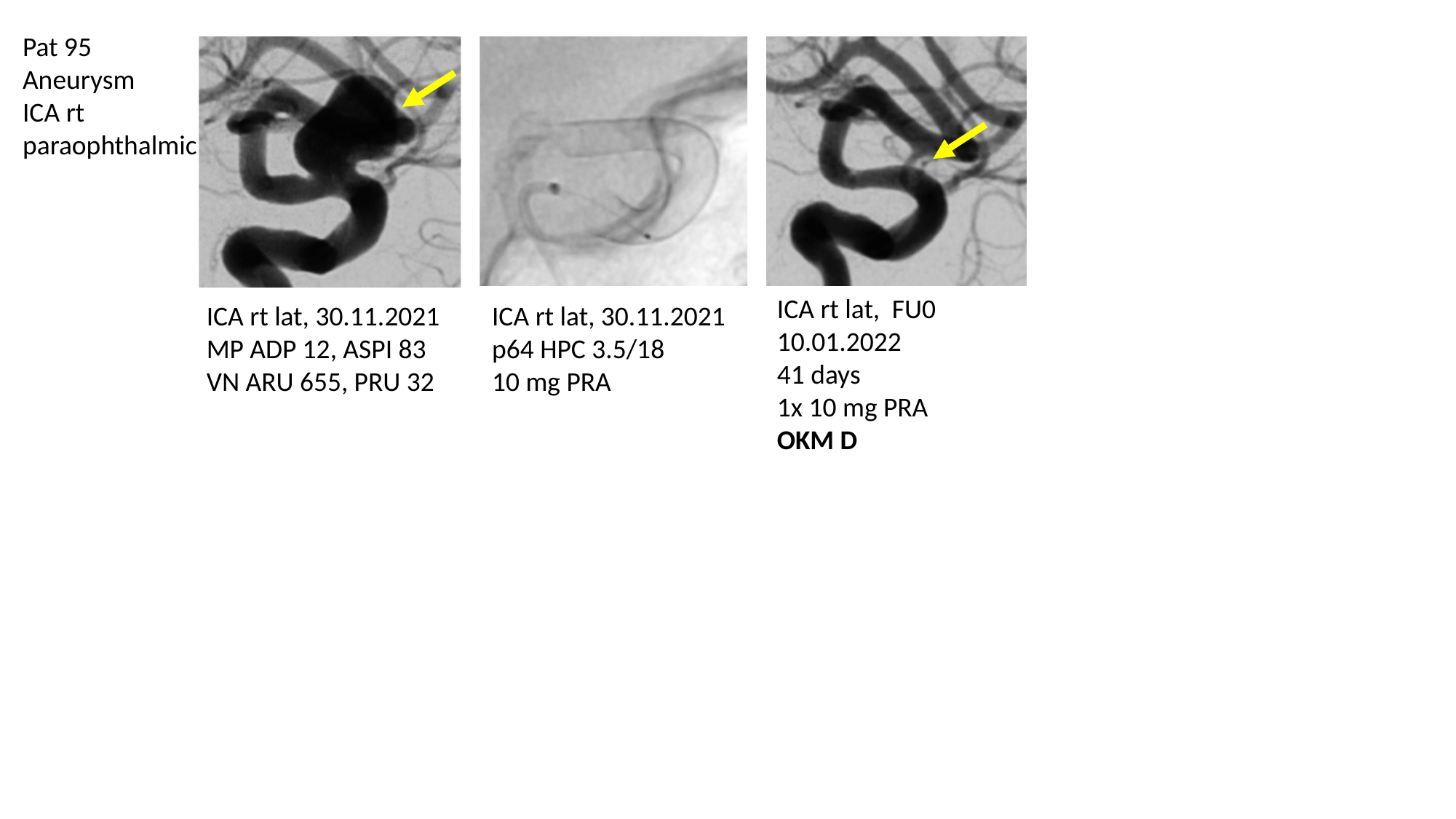

Pat 95
Aneurysm
ICA rt
paraophthalmic
ICA rt lat, FU0
10.01.2022
41 days
1x 10 mg PRA
OKM D
ICA rt lat, 30.11.2021
MP ADP 12, ASPI 83
VN ARU 655, PRU 32
ICA rt lat, 30.11.2021
p64 HPC 3.5/18
10 mg PRA

## Slide 80
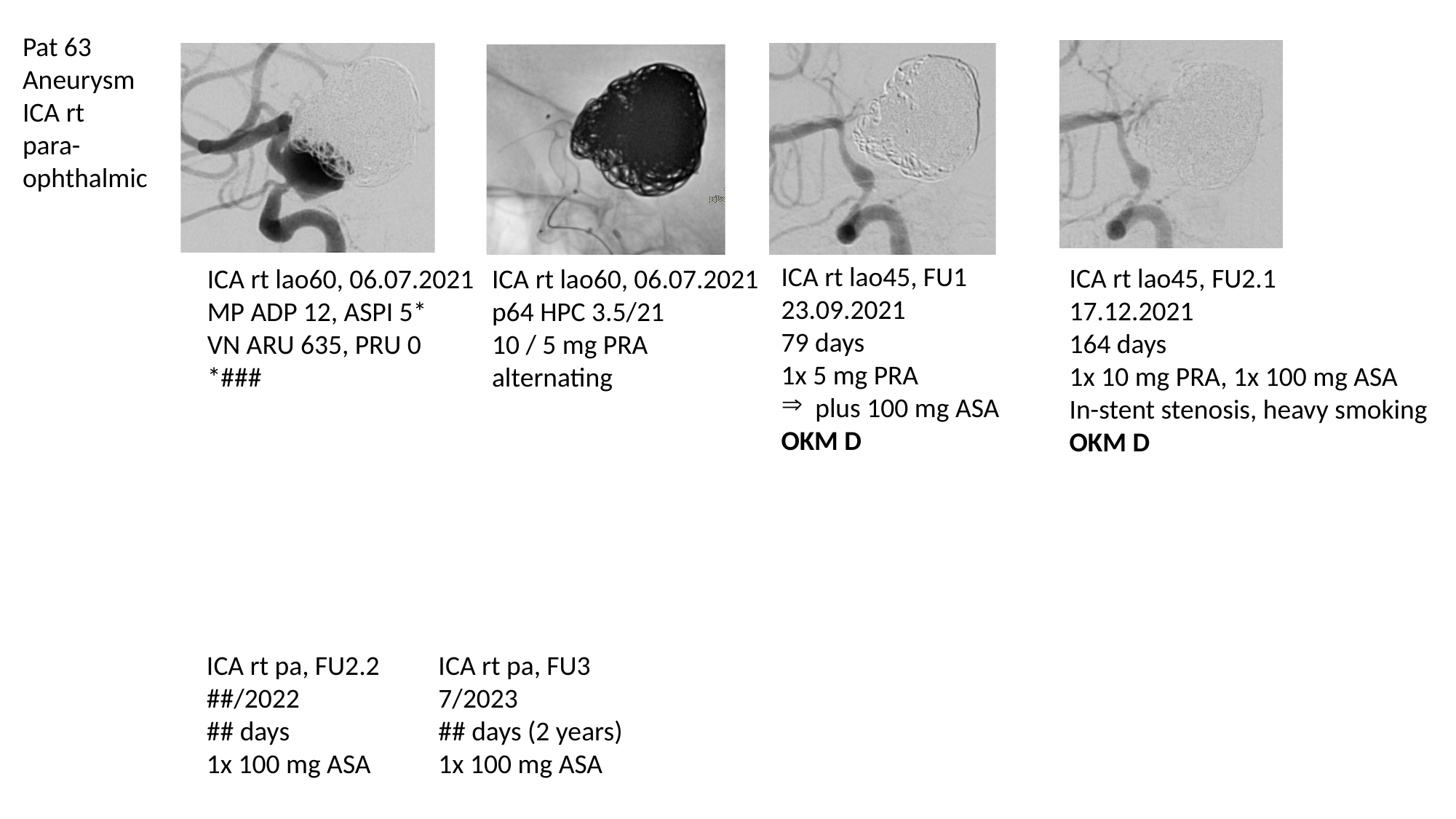

Pat 63
Aneurysm
ICA rt
para-
ophthalmic
ICA rt lao45, FU1
23.09.2021
79 days
1x 5 mg PRA
plus 100 mg ASA
OKM D
ICA rt lao45, FU2.1
17.12.2021
164 days
1x 10 mg PRA, 1x 100 mg ASA
In-stent stenosis, heavy smoking
OKM D
ICA rt lao60, 06.07.2021
MP ADP 12, ASPI 5*
VN ARU 635, PRU 0
*###
ICA rt lao60, 06.07.2021
p64 HPC 3.5/21
10 / 5 mg PRA
alternating
ICA rt pa, FU3
7/2023
## days (2 years)
1x 100 mg ASA
ICA rt pa, FU2.2
##/2022
## days
1x 100 mg ASA

## Slide 81
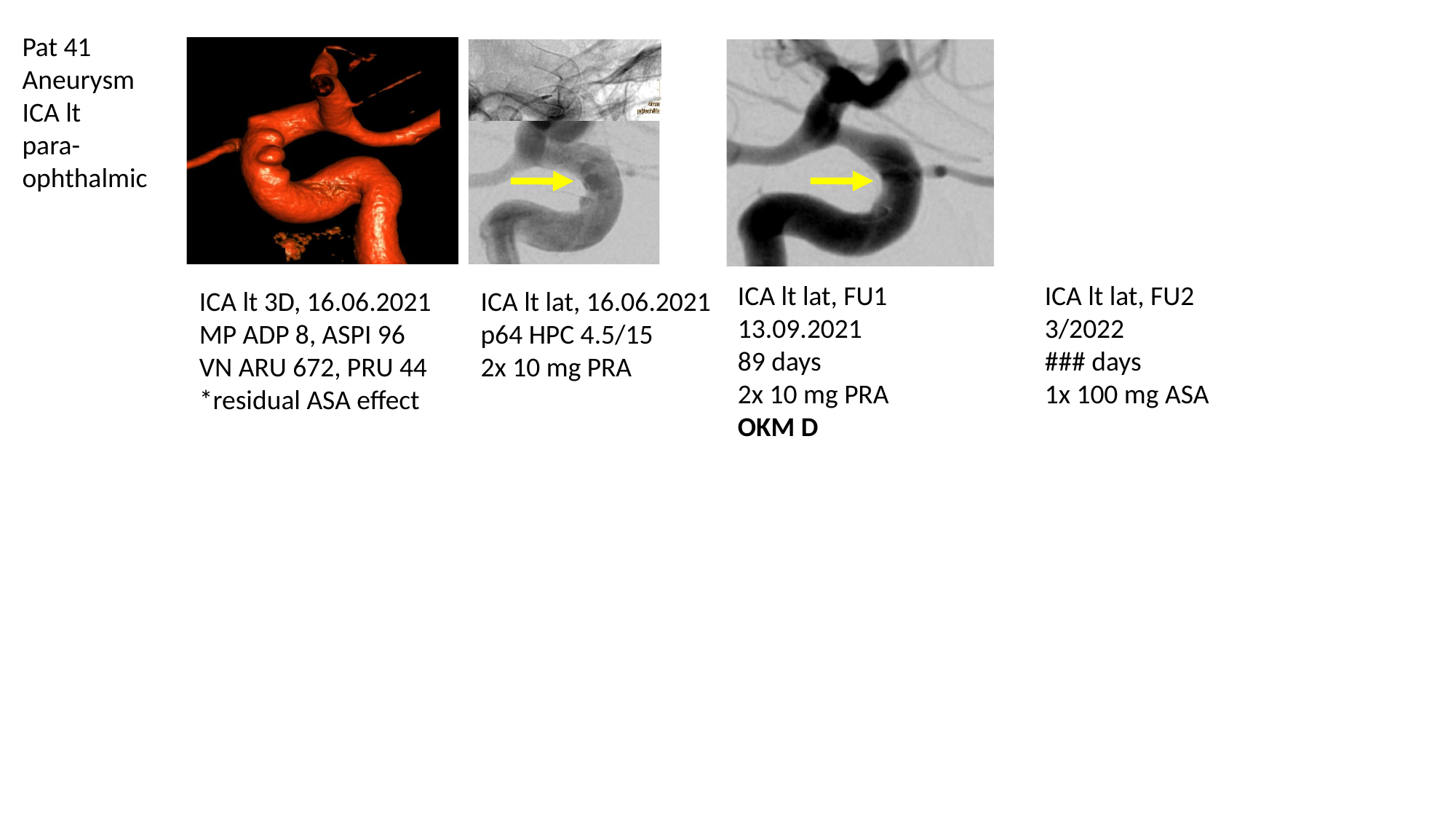

Pat 41
Aneurysm
ICA lt
para-
ophthalmic
ICA lt lat, FU1
13.09.2021
89 days
2x 10 mg PRA
OKM D
ICA lt lat, FU2
3/2022
### days
1x 100 mg ASA
ICA lt 3D, 16.06.2021
MP ADP 8, ASPI 96
VN ARU 672, PRU 44
*residual ASA effect
ICA lt lat, 16.06.2021
p64 HPC 4.5/15
2x 10 mg PRA

## Slide 82
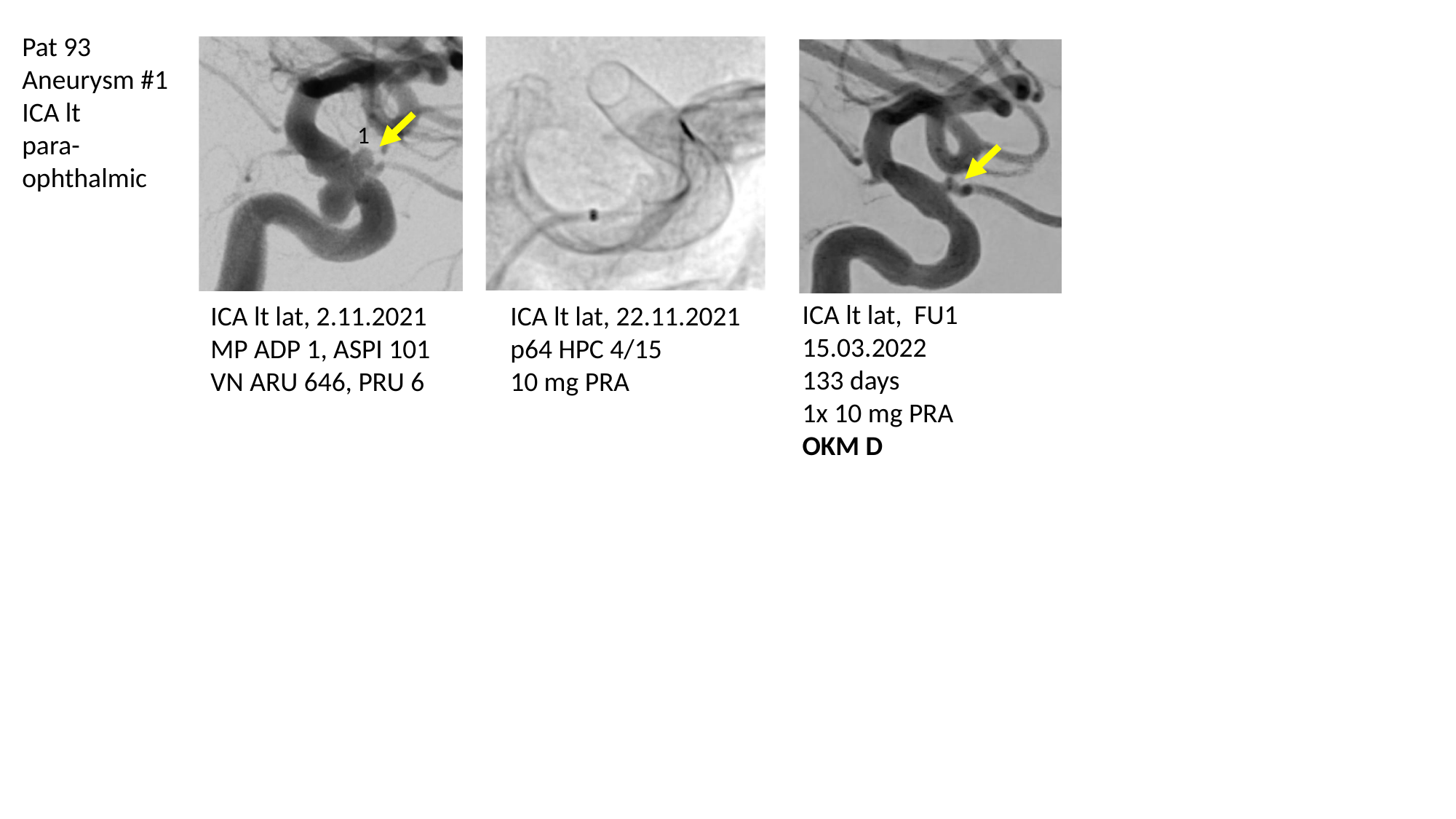

Pat 93
Aneurysm #1
ICA lt
para-
ophthalmic
1
ICA lt lat, FU1
15.03.2022
133 days
1x 10 mg PRA
OKM D
ICA lt lat, 22.11.2021
p64 HPC 4/15
10 mg PRA
ICA lt lat, 2.11.2021
MP ADP 1, ASPI 101
VN ARU 646, PRU 6

## Slide 83
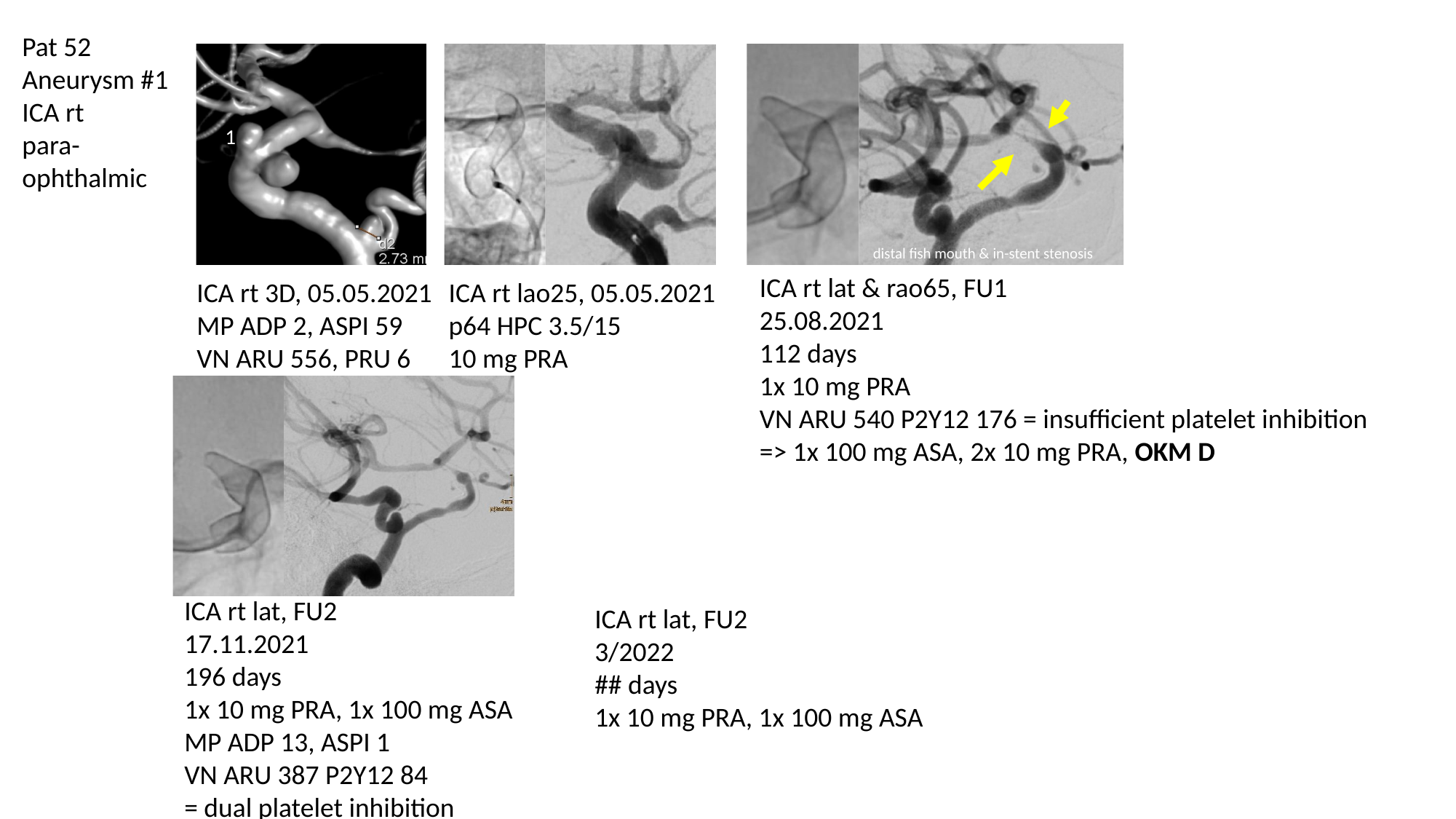

Pat 52
Aneurysm #1
ICA rt
para-
ophthalmic
1
distal fish mouth & in-stent stenosis
ICA rt lat & rao65, FU1
25.08.2021
112 days
1x 10 mg PRA
VN ARU 540 P2Y12 176 = insufficient platelet inhibition
=> 1x 100 mg ASA, 2x 10 mg PRA, OKM D
ICA rt 3D, 05.05.2021
MP ADP 2, ASPI 59
VN ARU 556, PRU 6
ICA rt lao25, 05.05.2021
p64 HPC 3.5/15
10 mg PRA
ICA rt lat, FU2
17.11.2021
196 days
1x 10 mg PRA, 1x 100 mg ASA
MP ADP 13, ASPI 1
VN ARU 387 P2Y12 84
= dual platelet inhibition
ICA rt lat, FU2
3/2022
## days
1x 10 mg PRA, 1x 100 mg ASA

## Slide 84
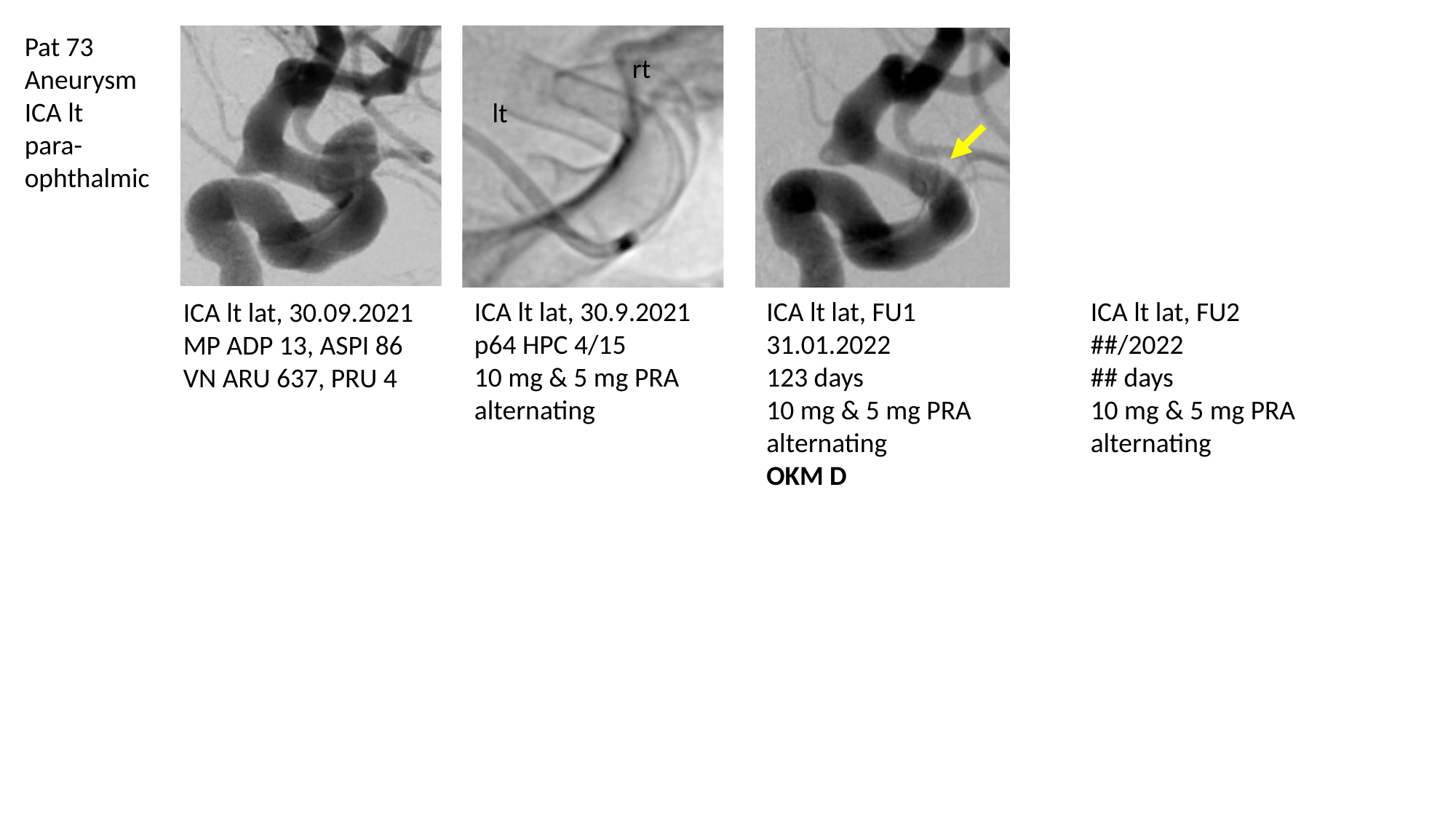

Pat 73
Aneurysm
ICA lt
para-
ophthalmic
rt
lt
ICA lt lat, FU1
31.01.2022
123 days
10 mg & 5 mg PRA
alternating
OKM D
ICA lt lat, 30.9.2021
p64 HPC 4/15
10 mg & 5 mg PRA
alternating
ICA lt lat, FU2
##/2022
## days
10 mg & 5 mg PRA
alternating
ICA lt lat, 30.09.2021
MP ADP 13, ASPI 86
VN ARU 637, PRU 4

## Slide 85
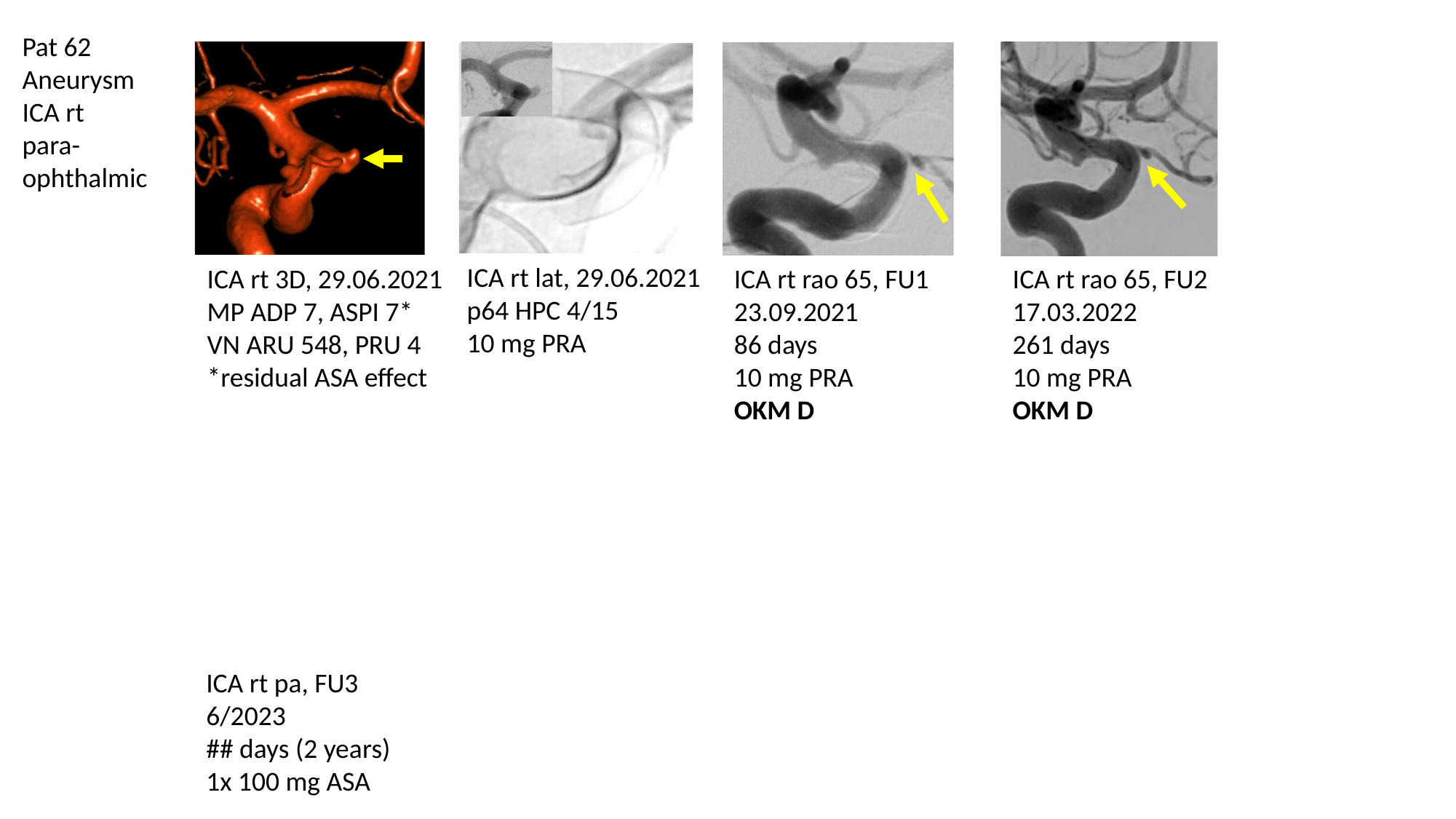

Pat 62
Aneurysm
ICA rt
para-
ophthalmic
ICA rt lat, 29.06.2021
p64 HPC 4/15
10 mg PRA
ICA rt 3D, 29.06.2021
MP ADP 7, ASPI 7*
VN ARU 548, PRU 4
*residual ASA effect
ICA rt rao 65, FU1
23.09.2021
86 days
10 mg PRA
OKM D
ICA rt rao 65, FU2
17.03.2022
261 days
10 mg PRA
OKM D
ICA rt pa, FU3
6/2023
## days (2 years)
1x 100 mg ASA

## Slide 86
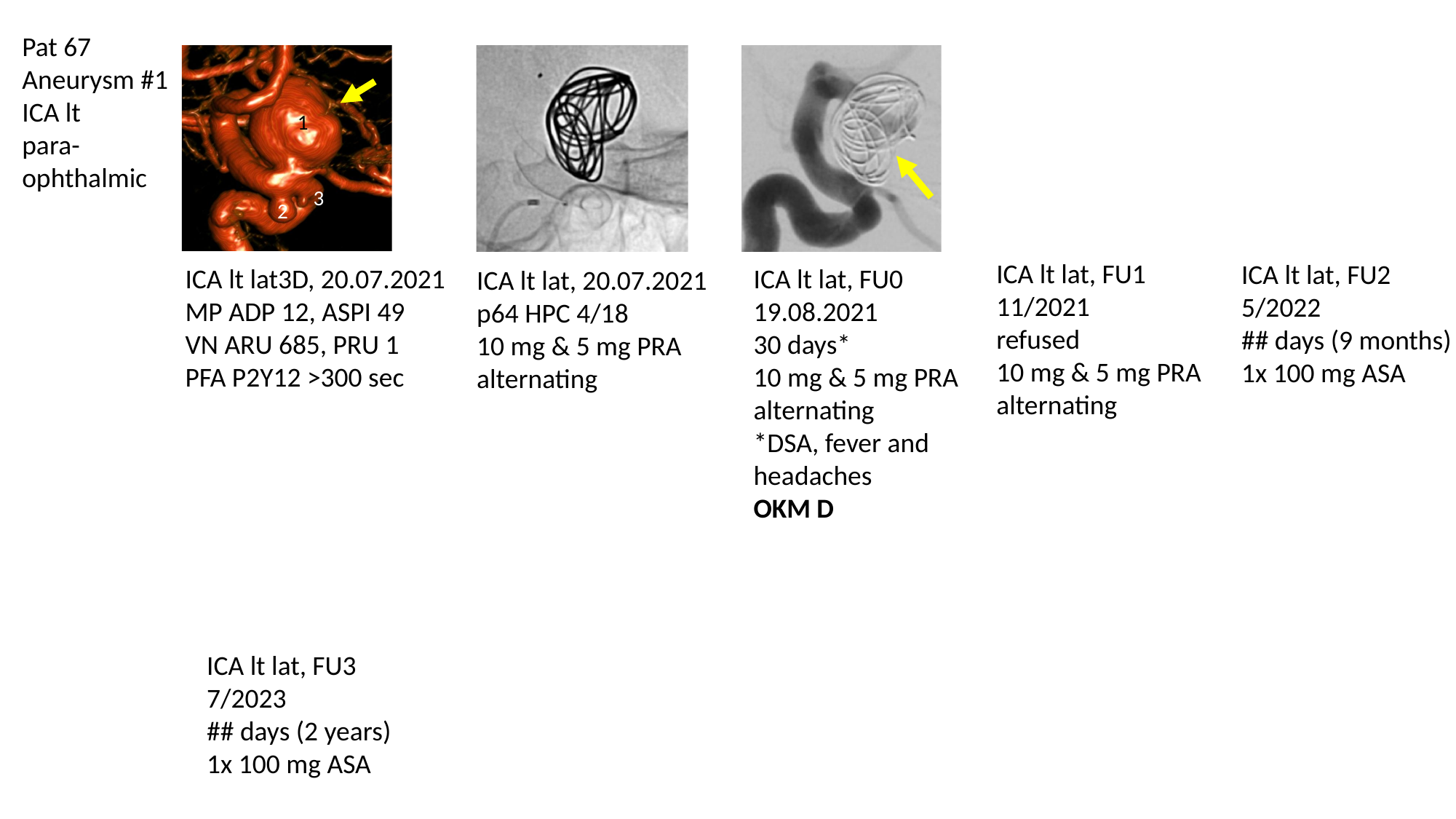

Pat 67
Aneurysm #1
ICA lt
para-
ophthalmic
1
3
2
ICA lt lat, FU1
11/2021
refused
10 mg & 5 mg PRA
alternating
ICA lt lat, FU2
5/2022
## days (9 months)
1x 100 mg ASA
ICA lt lat3D, 20.07.2021
MP ADP 12, ASPI 49
VN ARU 685, PRU 1
PFA P2Y12 >300 sec
ICA lt lat, FU0
19.08.2021
30 days*
10 mg & 5 mg PRA
alternating
*DSA, fever and
headaches
OKM D
ICA lt lat, 20.07.2021
p64 HPC 4/18
10 mg & 5 mg PRA
alternating
ICA lt lat, FU3
7/2023
## days (2 years)
1x 100 mg ASA

## Slide 87
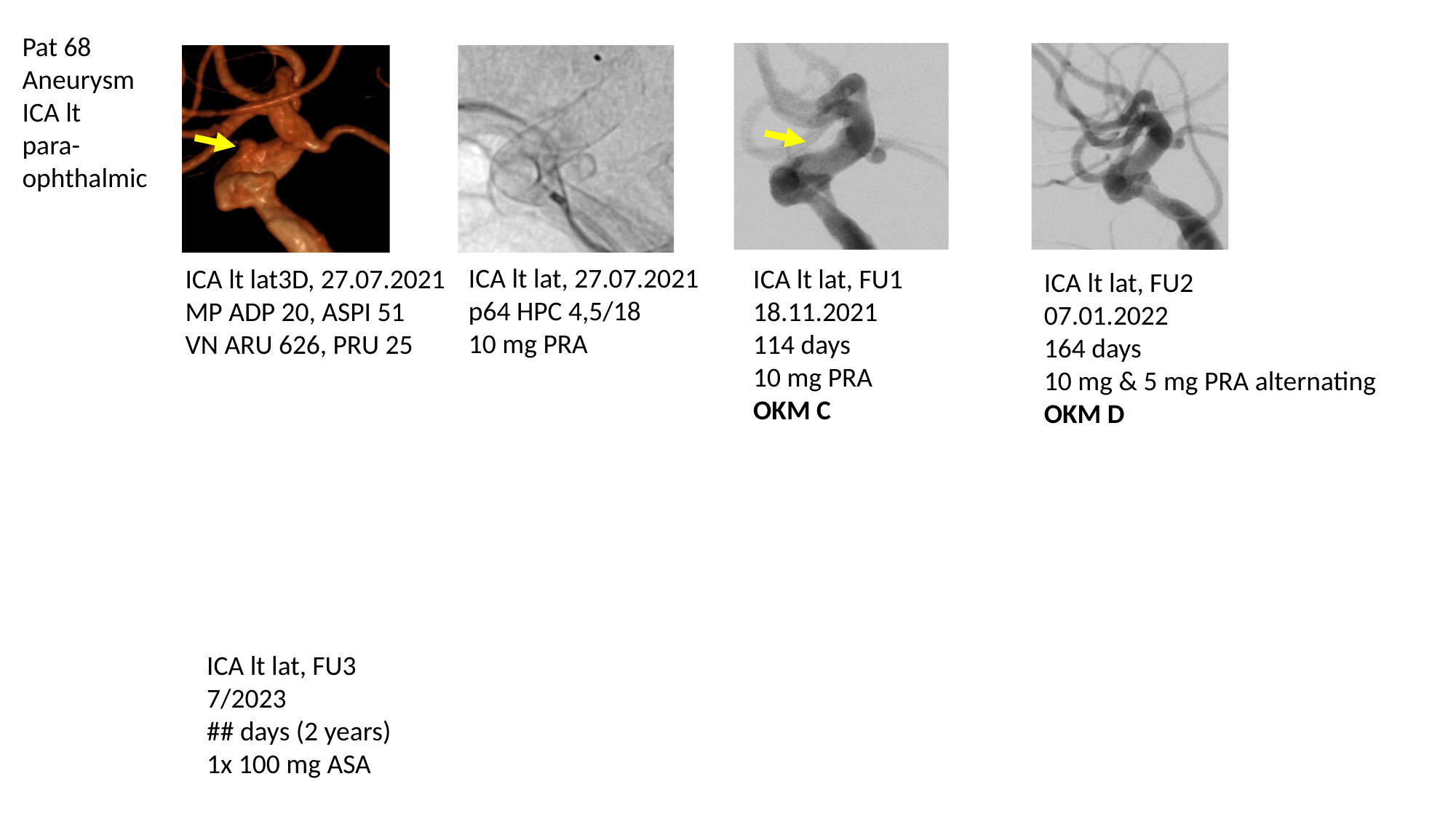

Pat 68
Aneurysm
ICA lt
para-
ophthalmic
ICA lt lat, 27.07.2021
p64 HPC 4,5/18
10 mg PRA
ICA lt lat3D, 27.07.2021
MP ADP 20, ASPI 51
VN ARU 626, PRU 25
ICA lt lat, FU1
18.11.2021
114 days
10 mg PRA
OKM C
ICA lt lat, FU2
07.01.2022
164 days
10 mg & 5 mg PRA alternating
OKM D
ICA lt lat, FU3
7/2023
## days (2 years)
1x 100 mg ASA

## Slide 88
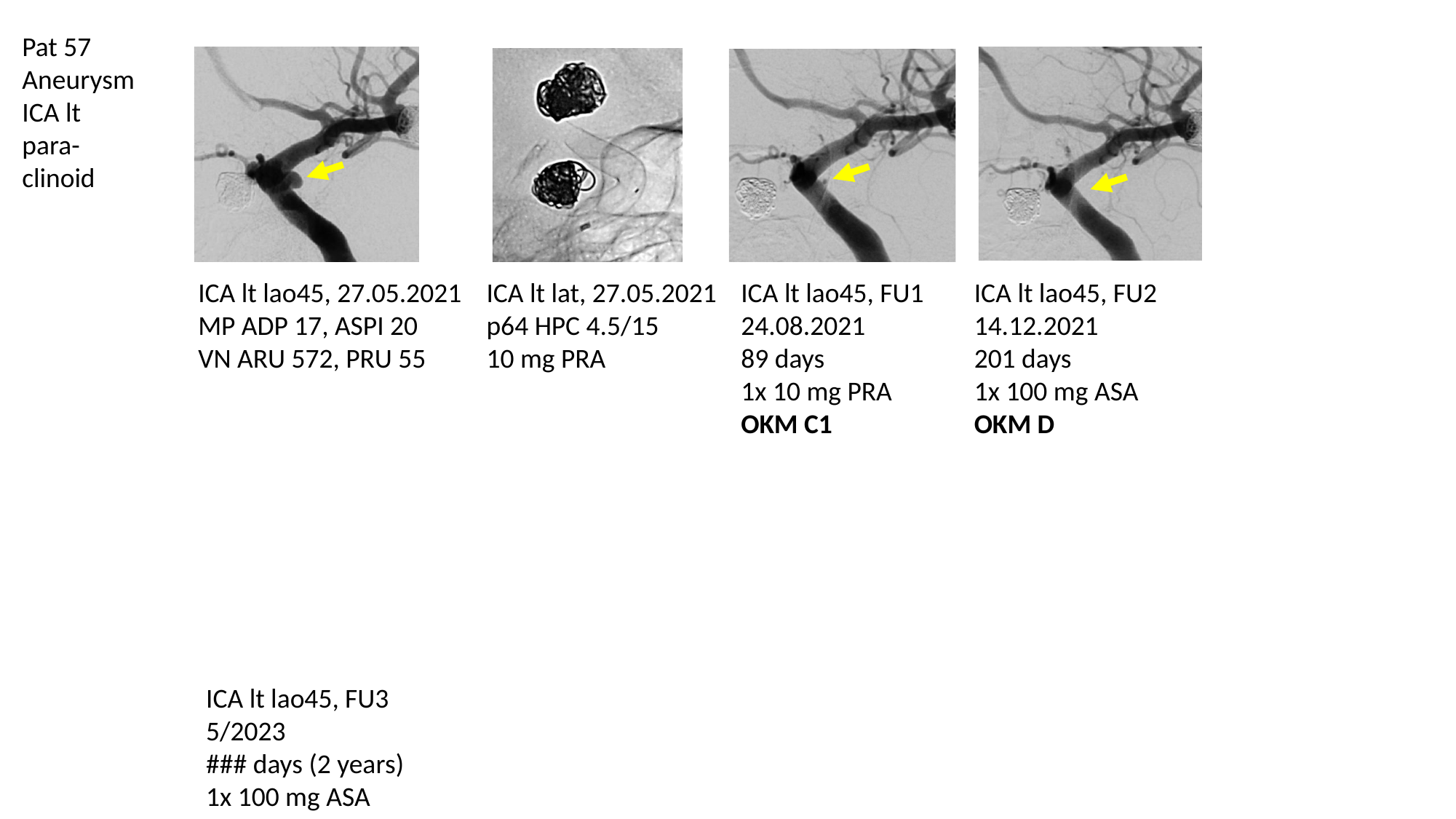

Pat 57
Aneurysm
ICA lt
para-
clinoid
ICA lt lao45, 27.05.2021
MP ADP 17, ASPI 20
VN ARU 572, PRU 55
ICA lt lat, 27.05.2021
p64 HPC 4.5/15
10 mg PRA
ICA lt lao45, FU1
24.08.2021
89 days
1x 10 mg PRA
OKM C1
ICA lt lao45, FU2
14.12.2021
201 days
1x 100 mg ASA
OKM D
ICA lt lao45, FU3
5/2023
### days (2 years)
1x 100 mg ASA

## Slide 89
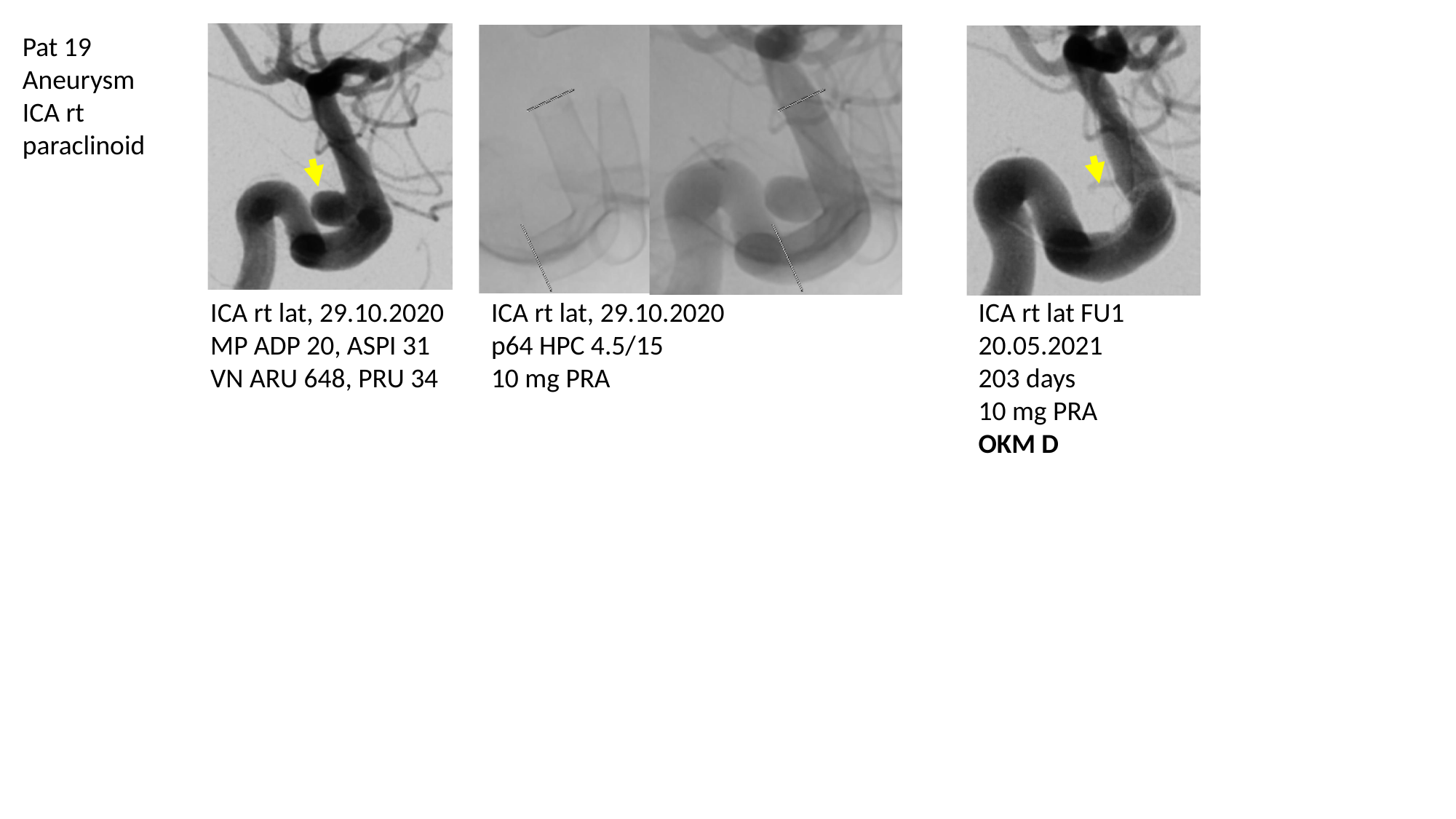

Pat 19
Aneurysm
ICA rt
paraclinoid
ICA rt lat, 29.10.2020
MP ADP 20, ASPI 31
VN ARU 648, PRU 34
ICA rt lat, 29.10.2020
p64 HPC 4.5/15
10 mg PRA
ICA rt lat FU1
20.05.2021
203 days
10 mg PRA
OKM D

## Slide 90
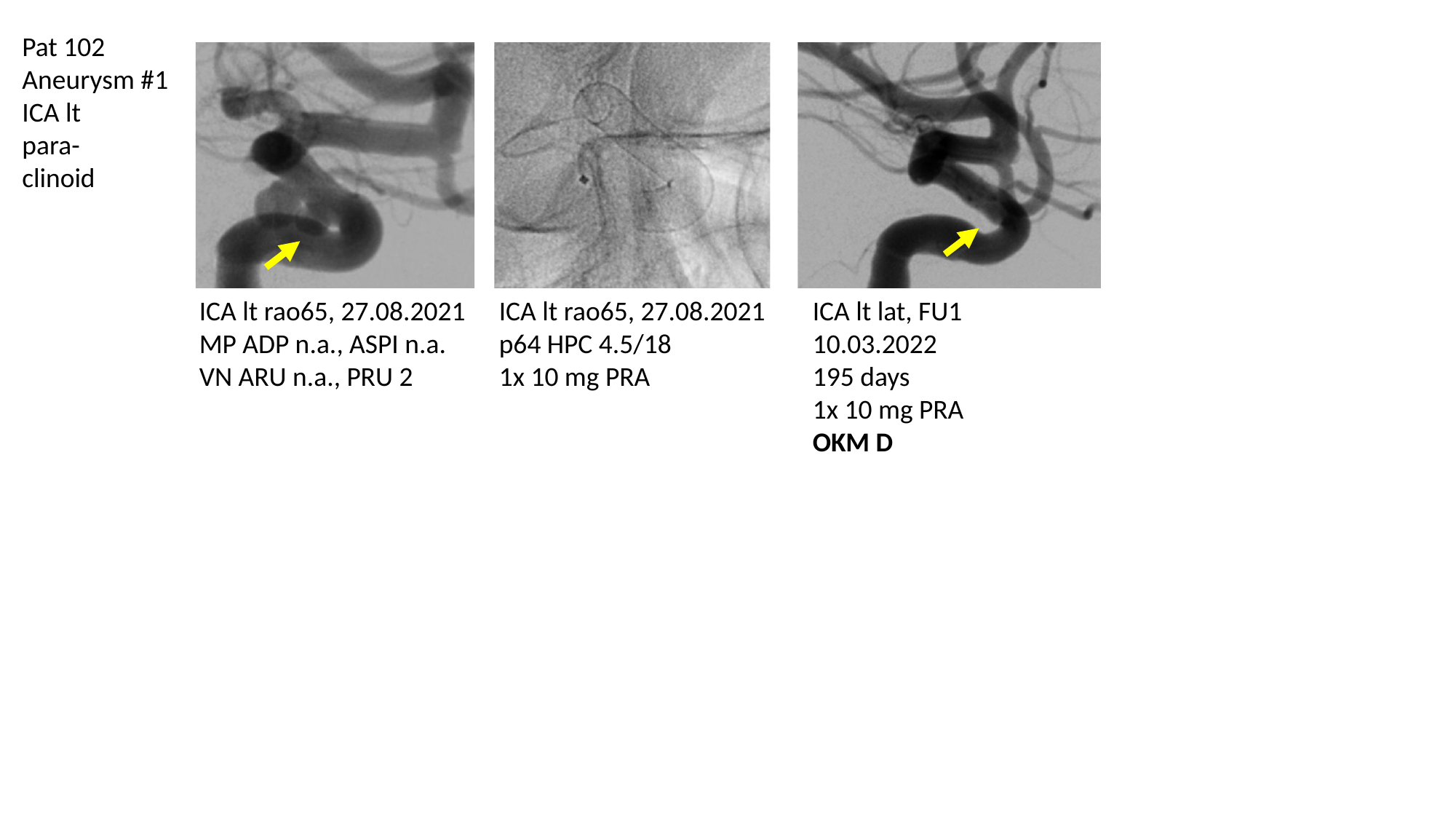

Pat 102
Aneurysm #1
ICA lt
para-
clinoid
ICA lt rao65, 27.08.2021
MP ADP n.a., ASPI n.a.
VN ARU n.a., PRU 2
ICA lt rao65, 27.08.2021
p64 HPC 4.5/18
1x 10 mg PRA
ICA lt lat, FU1
10.03.2022
195 days
1x 10 mg PRA
OKM D

## Slide 91
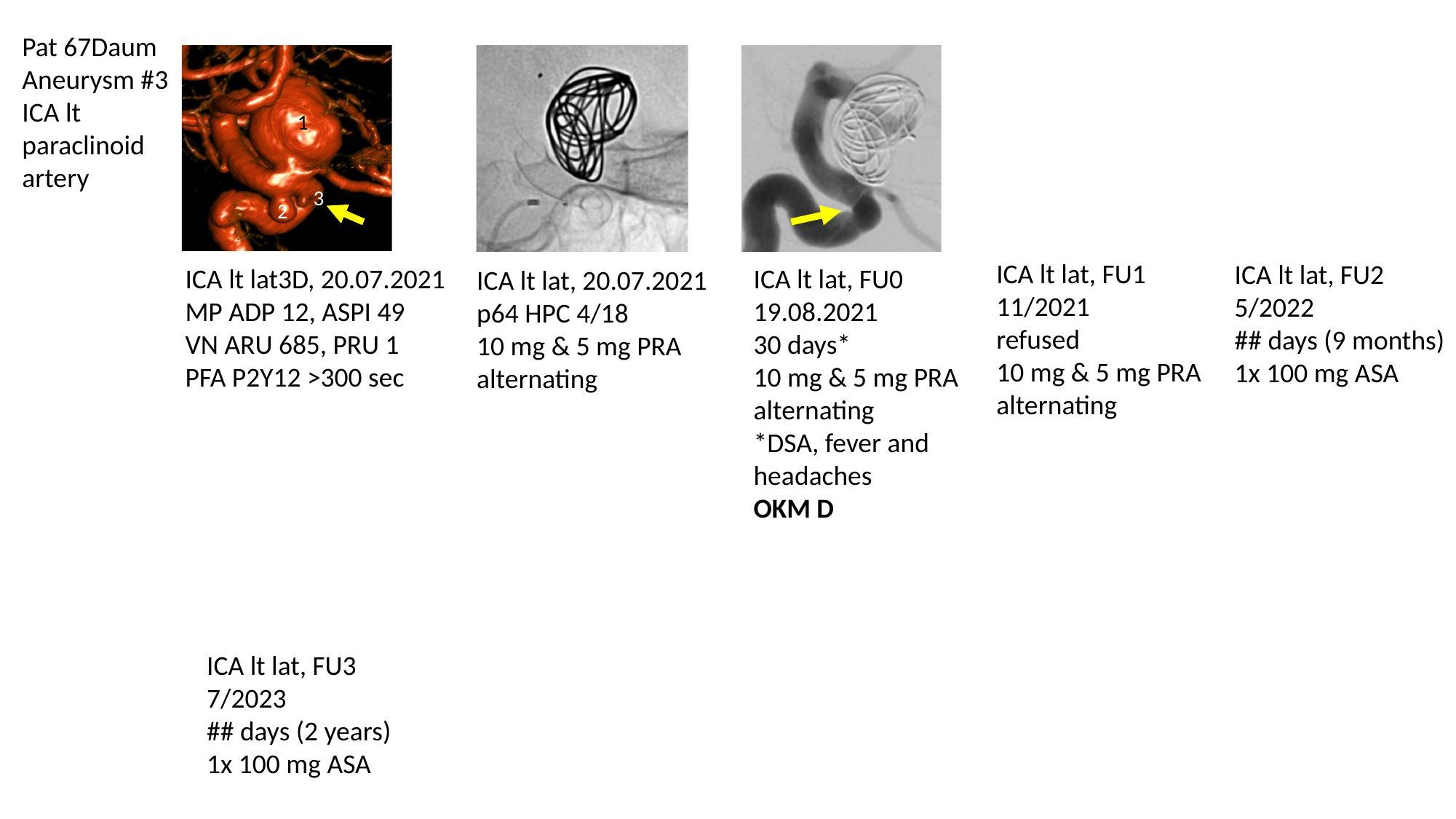

Pat 67Daum
Aneurysm #3
ICA lt
paraclinoid
artery
1
3
2
ICA lt lat, FU1
11/2021
refused
10 mg & 5 mg PRA
alternating
ICA lt lat, FU2
5/2022
## days (9 months)
1x 100 mg ASA
ICA lt lat3D, 20.07.2021
MP ADP 12, ASPI 49
VN ARU 685, PRU 1
PFA P2Y12 >300 sec
ICA lt lat, FU0
19.08.2021
30 days*
10 mg & 5 mg PRA
alternating
*DSA, fever and
headaches
OKM D
ICA lt lat, 20.07.2021
p64 HPC 4/18
10 mg & 5 mg PRA
alternating
ICA lt lat, FU3
7/2023
## days (2 years)
1x 100 mg ASA

## Slide 92
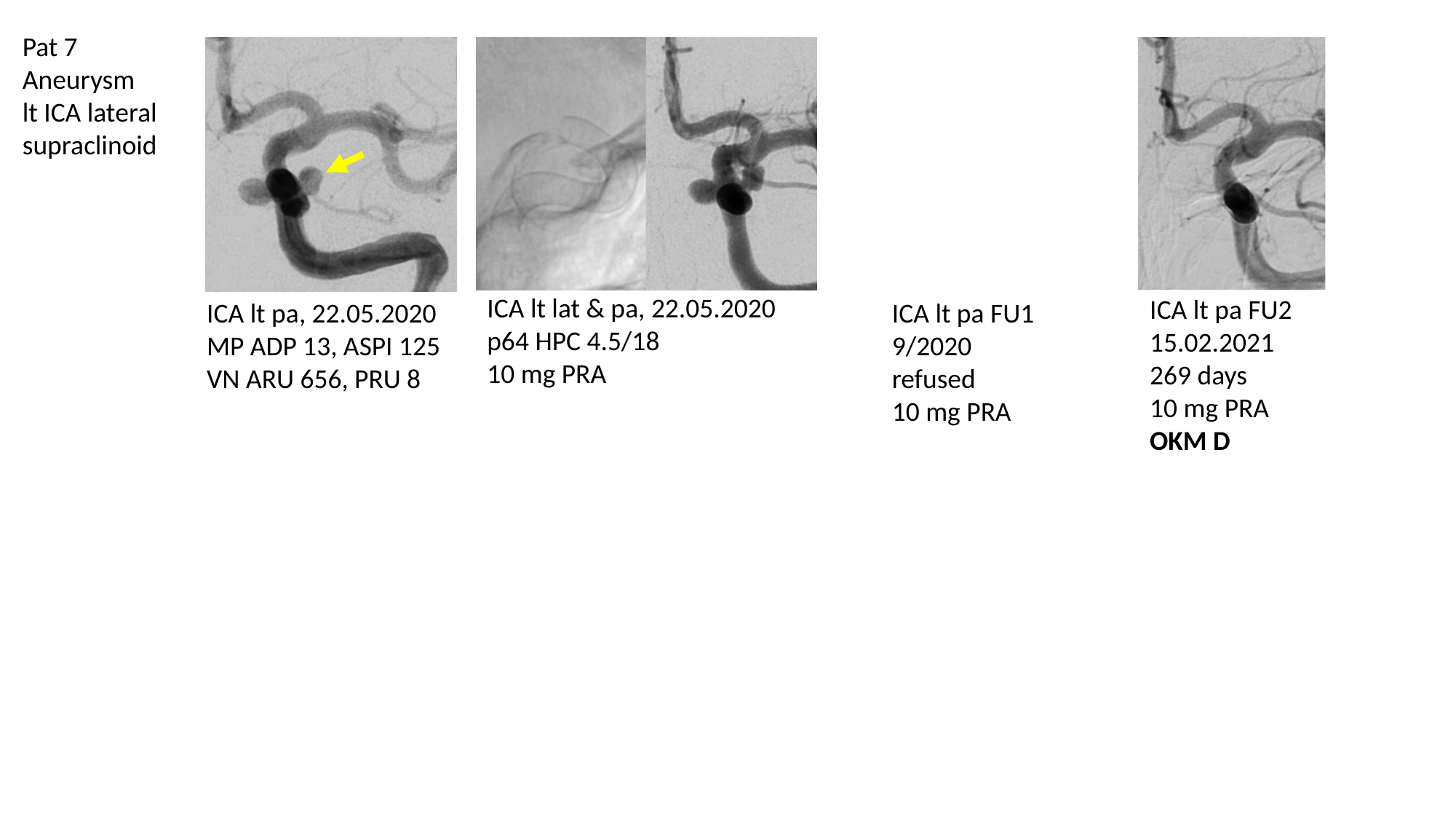

Pat 7
Aneurysm
lt ICA lateral
supraclinoid
ICA lt lat & pa, 22.05.2020
p64 HPC 4.5/18
10 mg PRA
ICA lt pa FU2
15.02.2021
269 days
10 mg PRA
OKM D
ICA lt pa, 22.05.2020
MP ADP 13, ASPI 125
VN ARU 656, PRU 8
ICA lt pa FU1
9/2020
refused
10 mg PRA

## Slide 93
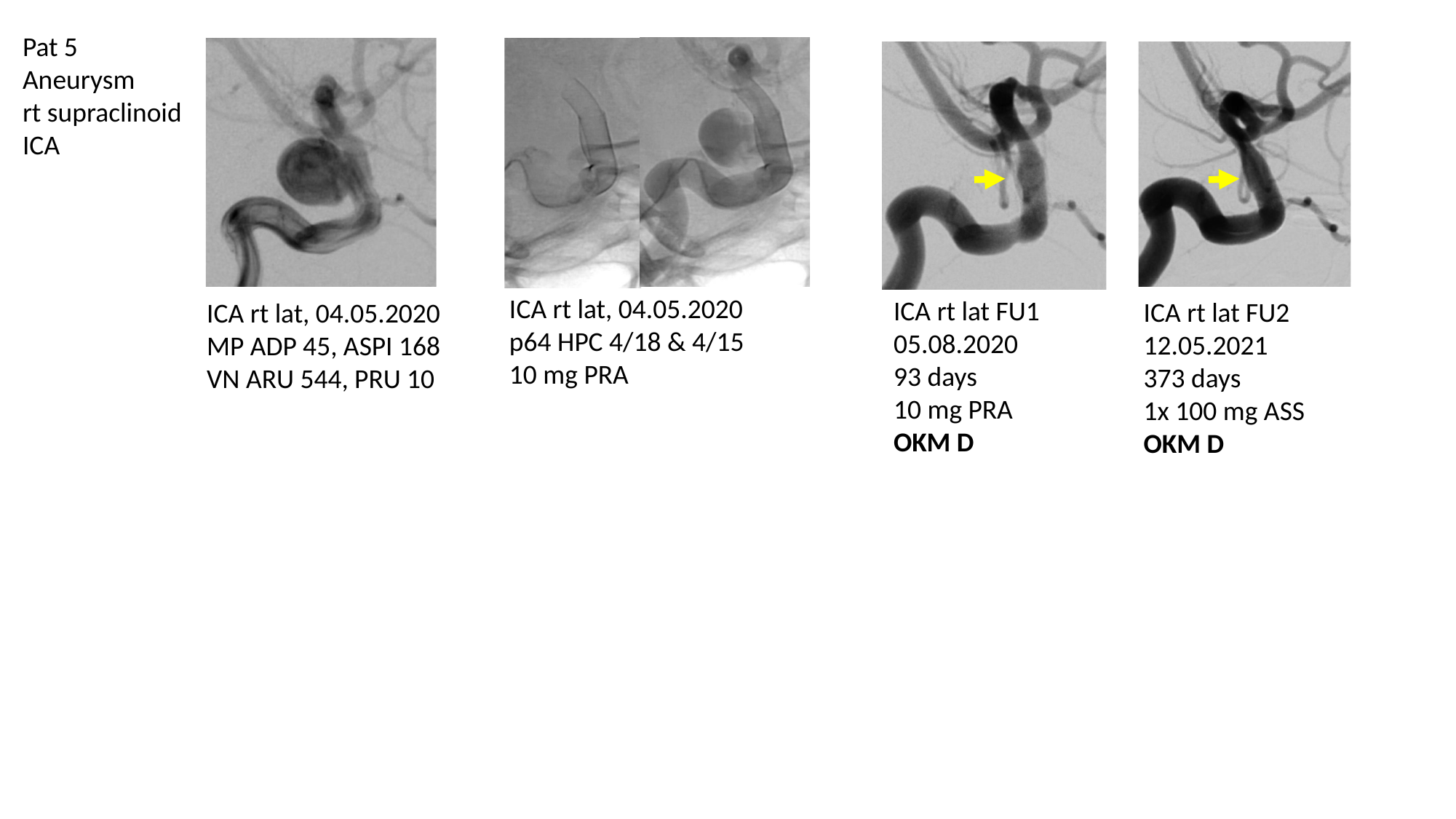

Pat 5
Aneurysm
rt supraclinoid
ICA
ICA rt lat, 04.05.2020
p64 HPC 4/18 & 4/15
10 mg PRA
ICA rt lat FU1
05.08.2020
93 days
10 mg PRA
OKM D
ICA rt lat FU2
12.05.2021
373 days
1x 100 mg ASS
OKM D
ICA rt lat, 04.05.2020
MP ADP 45, ASPI 168
VN ARU 544, PRU 10

## Slide 94
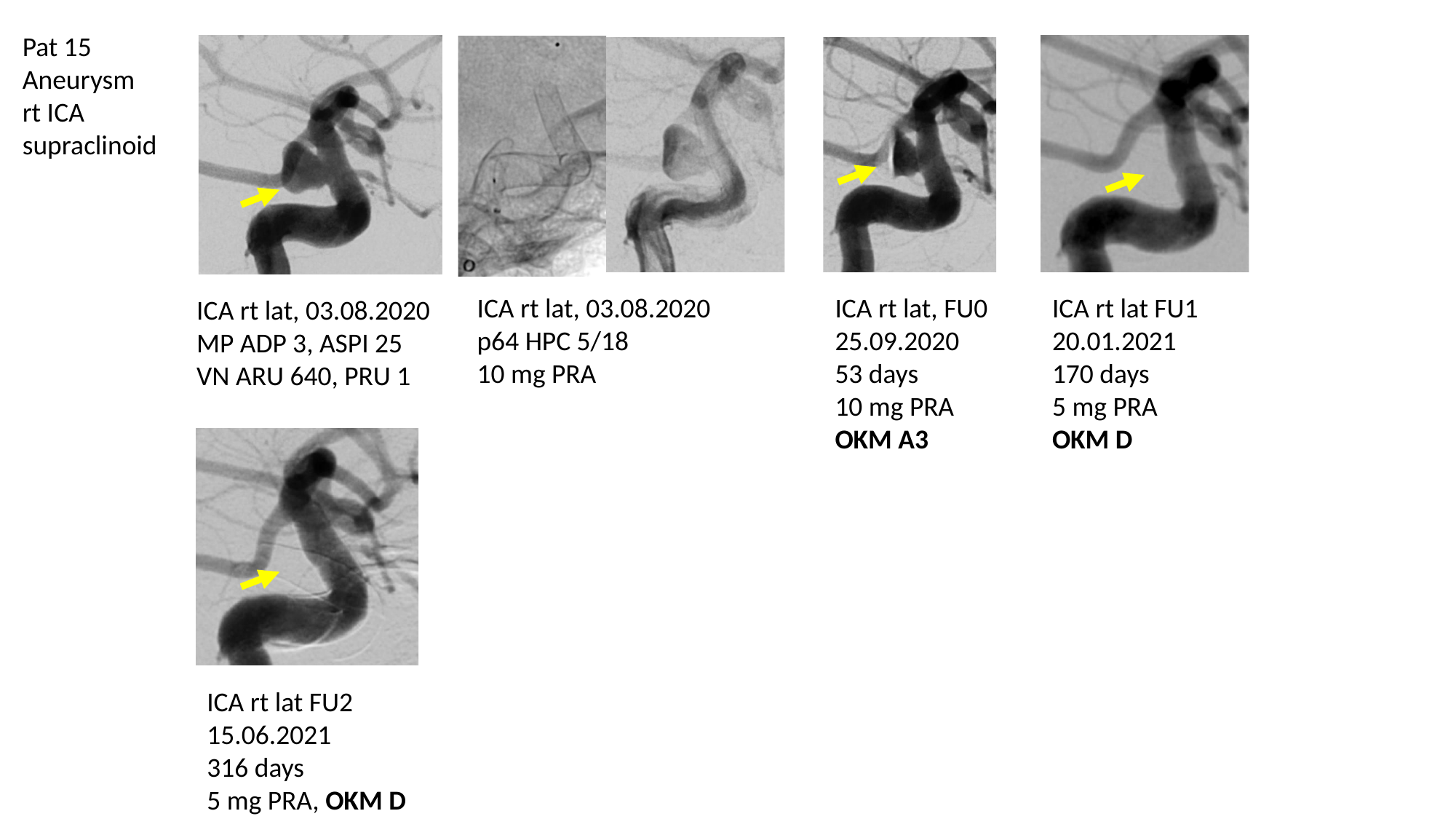

Pat 15
Aneurysm
rt ICA
supraclinoid
ICA rt lat FU1
20.01.2021
170 days
5 mg PRA
OKM D
ICA rt lat, 03.08.2020
p64 HPC 5/18
10 mg PRA
ICA rt lat, FU0
25.09.2020
53 days
10 mg PRA
OKM A3
ICA rt lat, 03.08.2020
MP ADP 3, ASPI 25
VN ARU 640, PRU 1
ICA rt lat FU2
15.06.2021
316 days
5 mg PRA, OKM D

## Slide 95
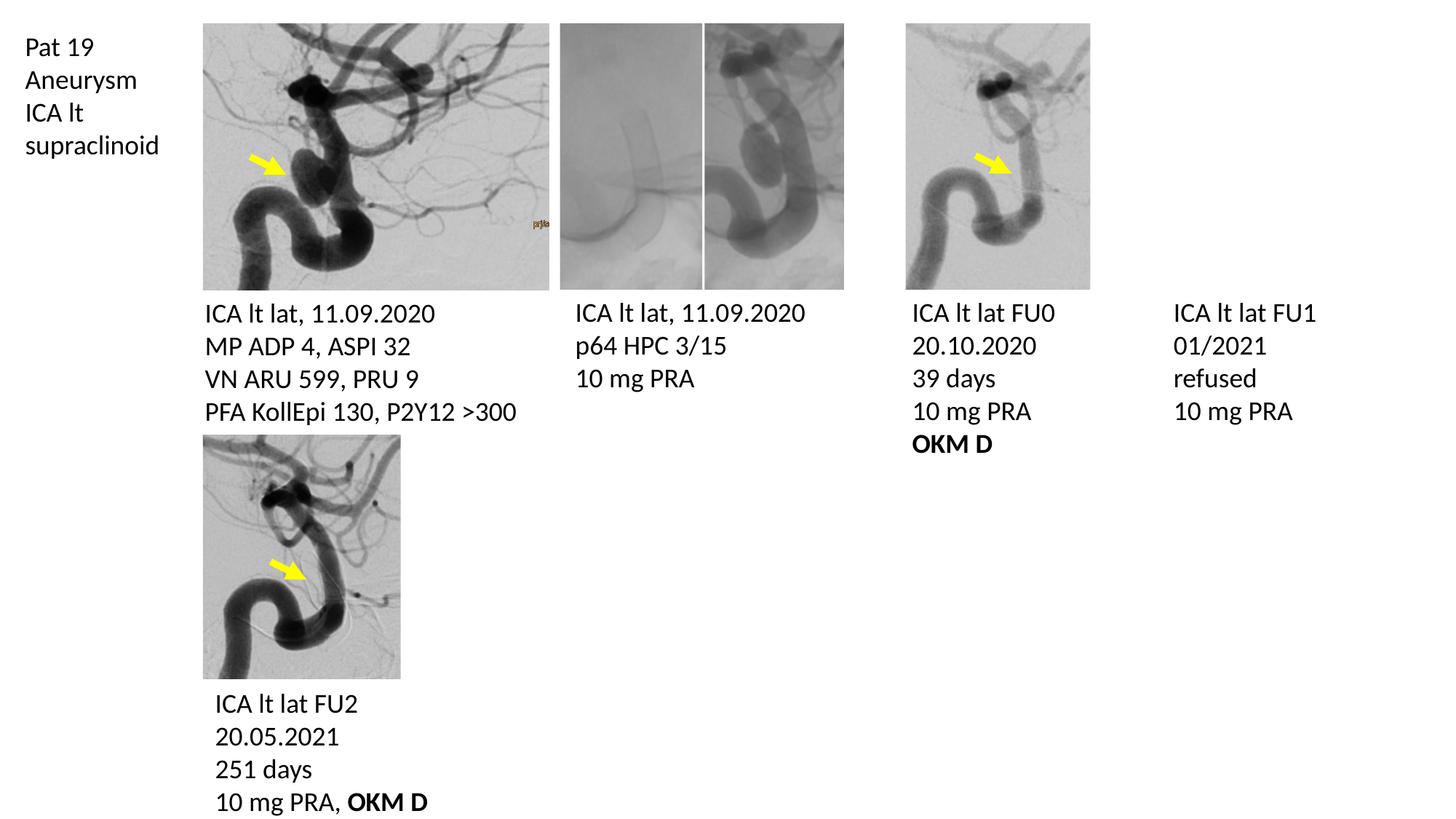

Pat 19
Aneurysm
ICA lt
supraclinoid
ICA lt lat FU0
20.10.2020
39 days
10 mg PRA
OKM D
ICA lt lat FU1
01/2021
refused
10 mg PRA
ICA lt lat, 11.09.2020
p64 HPC 3/15
10 mg PRA
ICA lt lat, 11.09.2020
MP ADP 4, ASPI 32
VN ARU 599, PRU 9
PFA KollEpi 130, P2Y12 >300
ICA lt lat FU2
20.05.2021
251 days
10 mg PRA, OKM D

## Slide 96
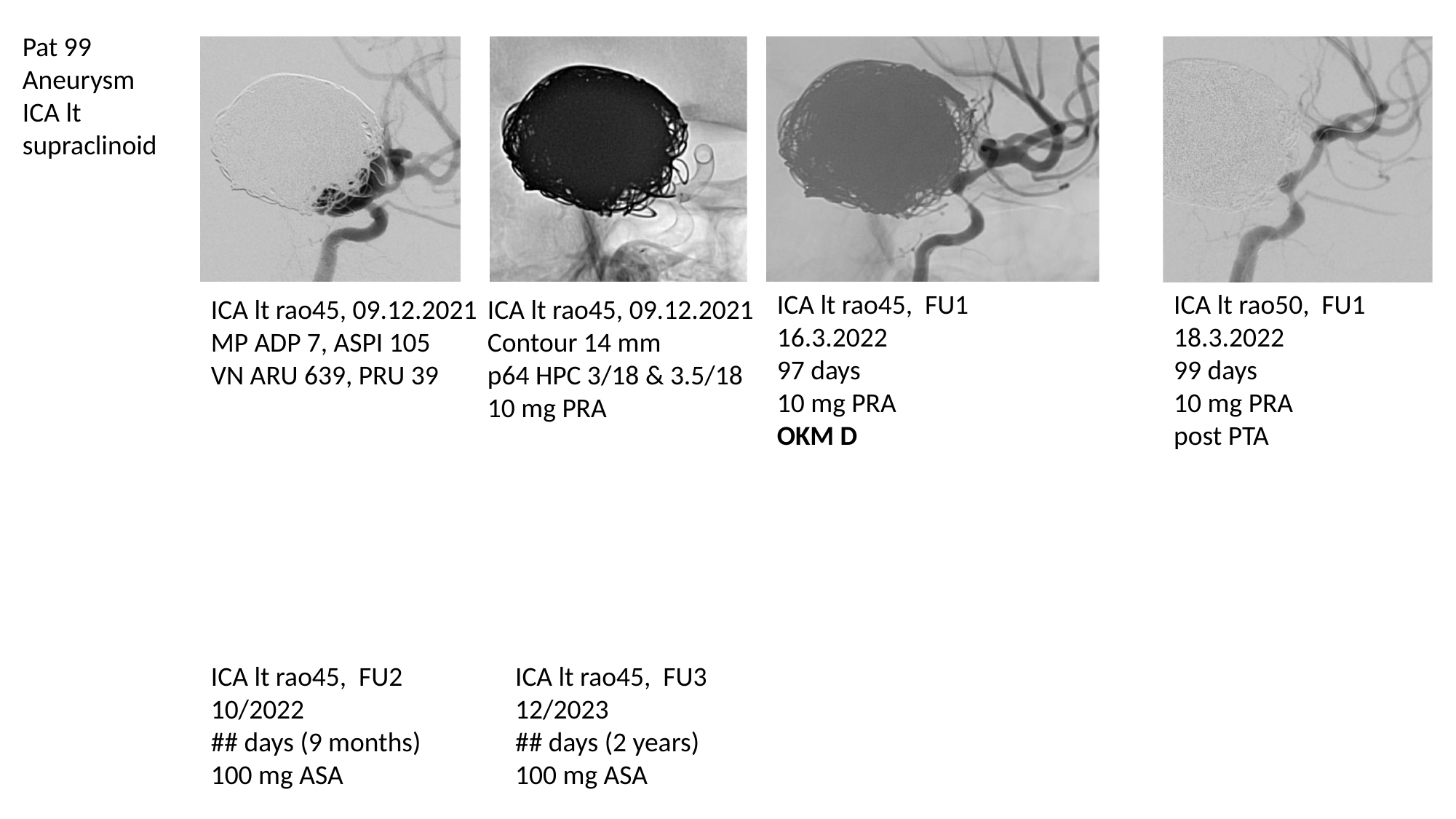

Pat 99
Aneurysm
ICA lt
supraclinoid
ICA lt rao50, FU1
18.3.2022
99 days
10 mg PRA
post PTA
ICA lt rao45, FU1
16.3.2022
97 days
10 mg PRA
OKM D
ICA lt rao45, 09.12.2021
MP ADP 7, ASPI 105
VN ARU 639, PRU 39
ICA lt rao45, 09.12.2021
Contour 14 mm
p64 HPC 3/18 & 3.5/18
10 mg PRA
ICA lt rao45, FU3
12/2023
## days (2 years)
100 mg ASA
ICA lt rao45, FU2
10/2022
## days (9 months)
100 mg ASA

## Slide 97
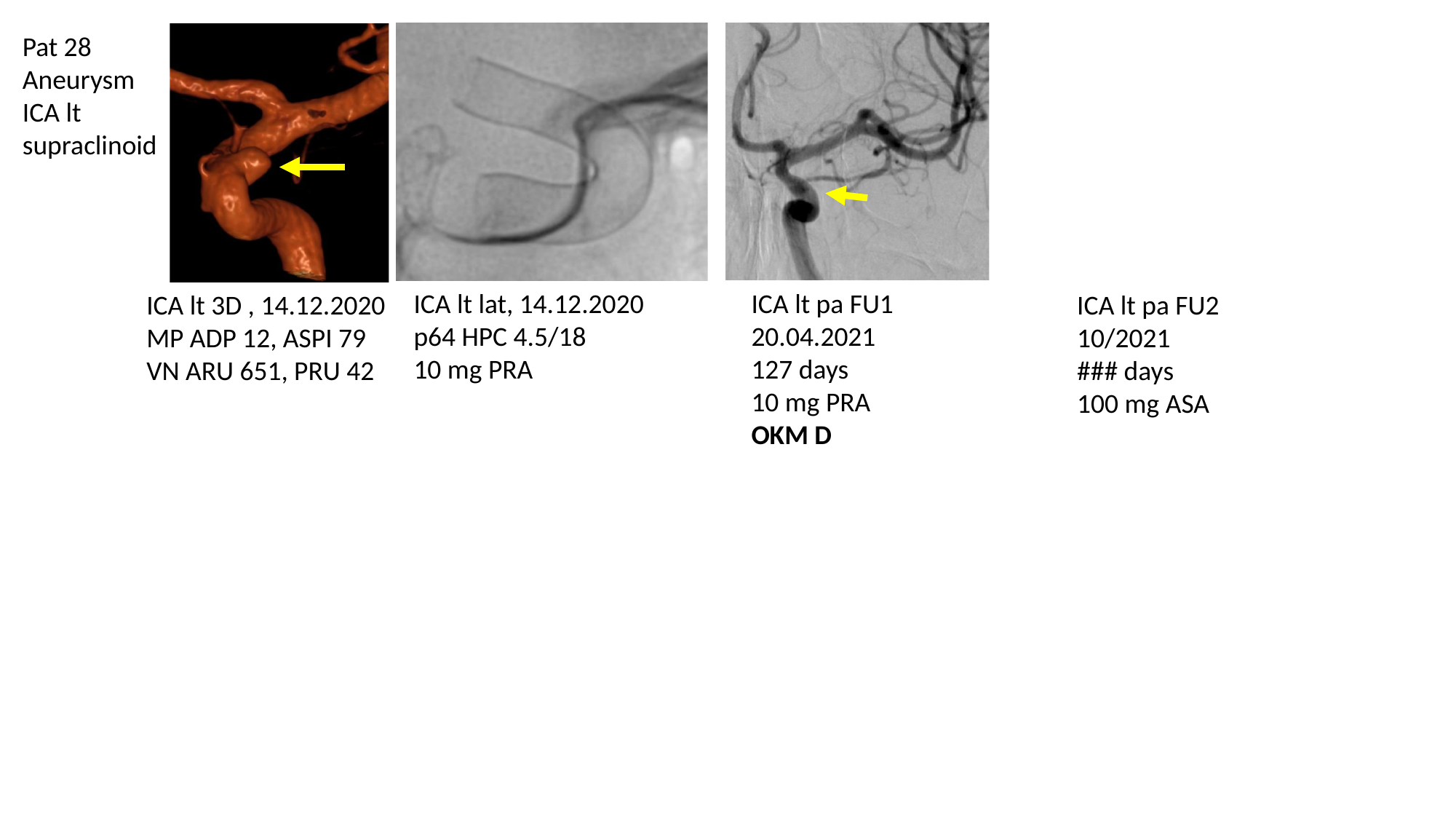

Pat 28
Aneurysm
ICA lt
supraclinoid
ICA lt pa FU1
20.04.2021
127 days
10 mg PRA
OKM D
ICA lt lat, 14.12.2020
p64 HPC 4.5/18
10 mg PRA
ICA lt 3D , 14.12.2020
MP ADP 12, ASPI 79
VN ARU 651, PRU 42
ICA lt pa FU2
10/2021
### days
100 mg ASA

## Slide 98
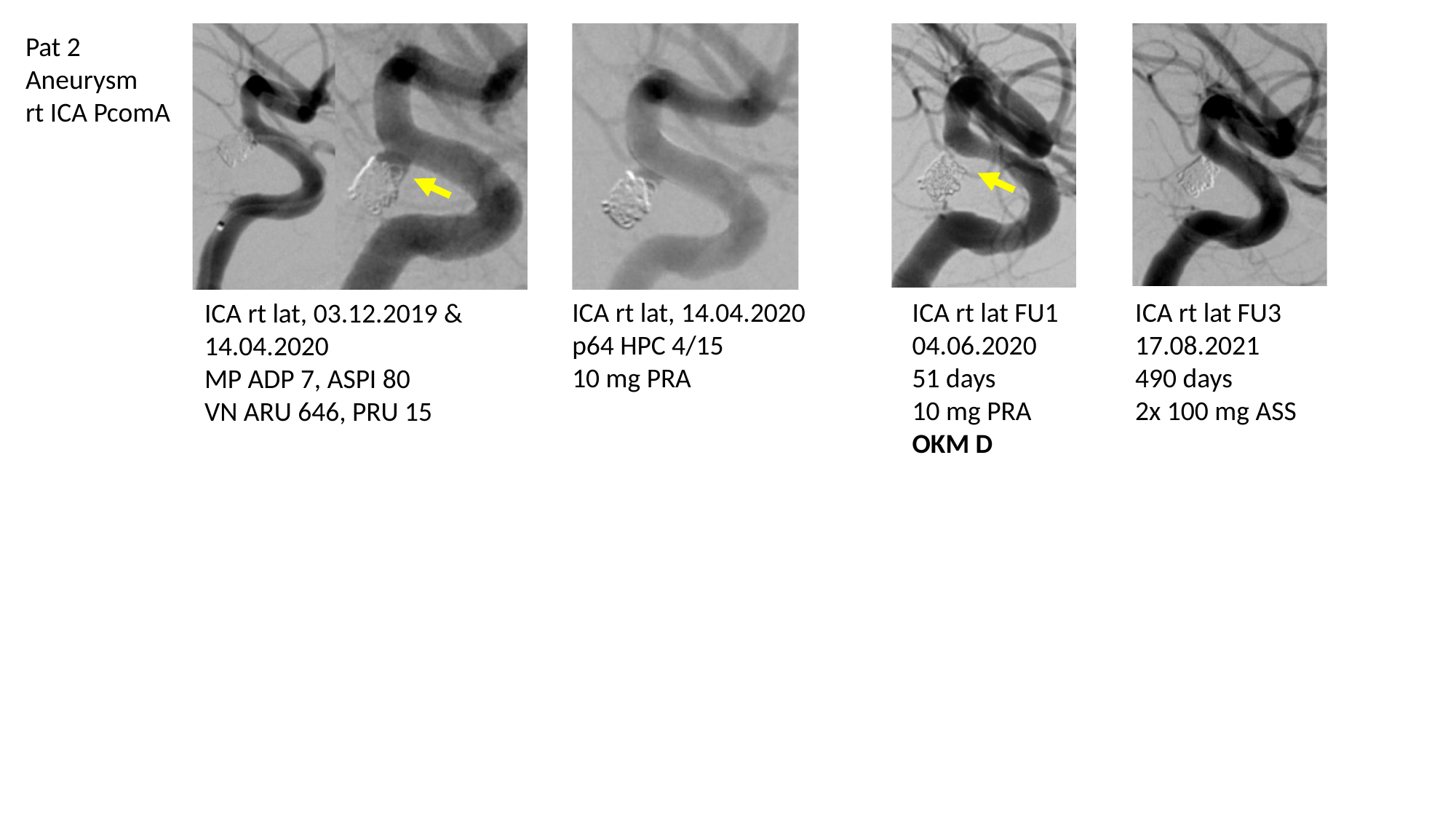

Pat 2
Aneurysm
rt ICA PcomA
ICA rt lat FU1
04.06.2020
51 days
10 mg PRA
OKM D
ICA rt lat FU3
17.08.2021
490 days
2x 100 mg ASS
ICA rt lat, 14.04.2020
p64 HPC 4/15
10 mg PRA
ICA rt lat, 03.12.2019 &
14.04.2020
MP ADP 7, ASPI 80
VN ARU 646, PRU 15

## Slide 99
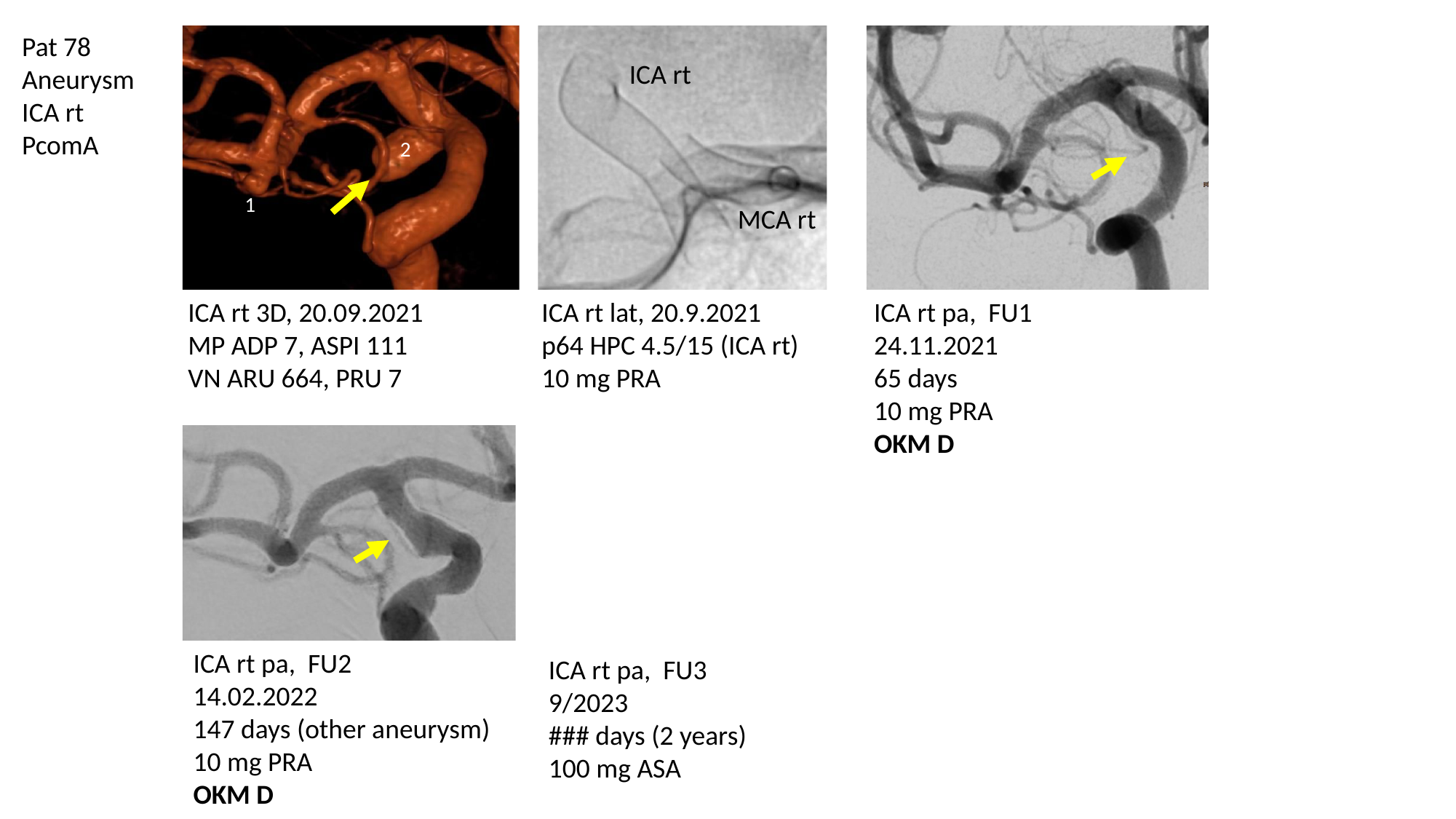

Pat 78
Aneurysm
ICA rt
PcomA
ICA rt
2
1
MCA rt
ICA rt lat, 20.9.2021
p64 HPC 4.5/15 (ICA rt)
10 mg PRA
ICA rt pa, FU1
24.11.2021
65 days
10 mg PRA
OKM D
ICA rt 3D, 20.09.2021
MP ADP 7, ASPI 111
VN ARU 664, PRU 7
ICA rt pa, FU2
14.02.2022
147 days (other aneurysm)
10 mg PRA
OKM D
ICA rt pa, FU3
9/2023
### days (2 years)
100 mg ASA

## Slide 100
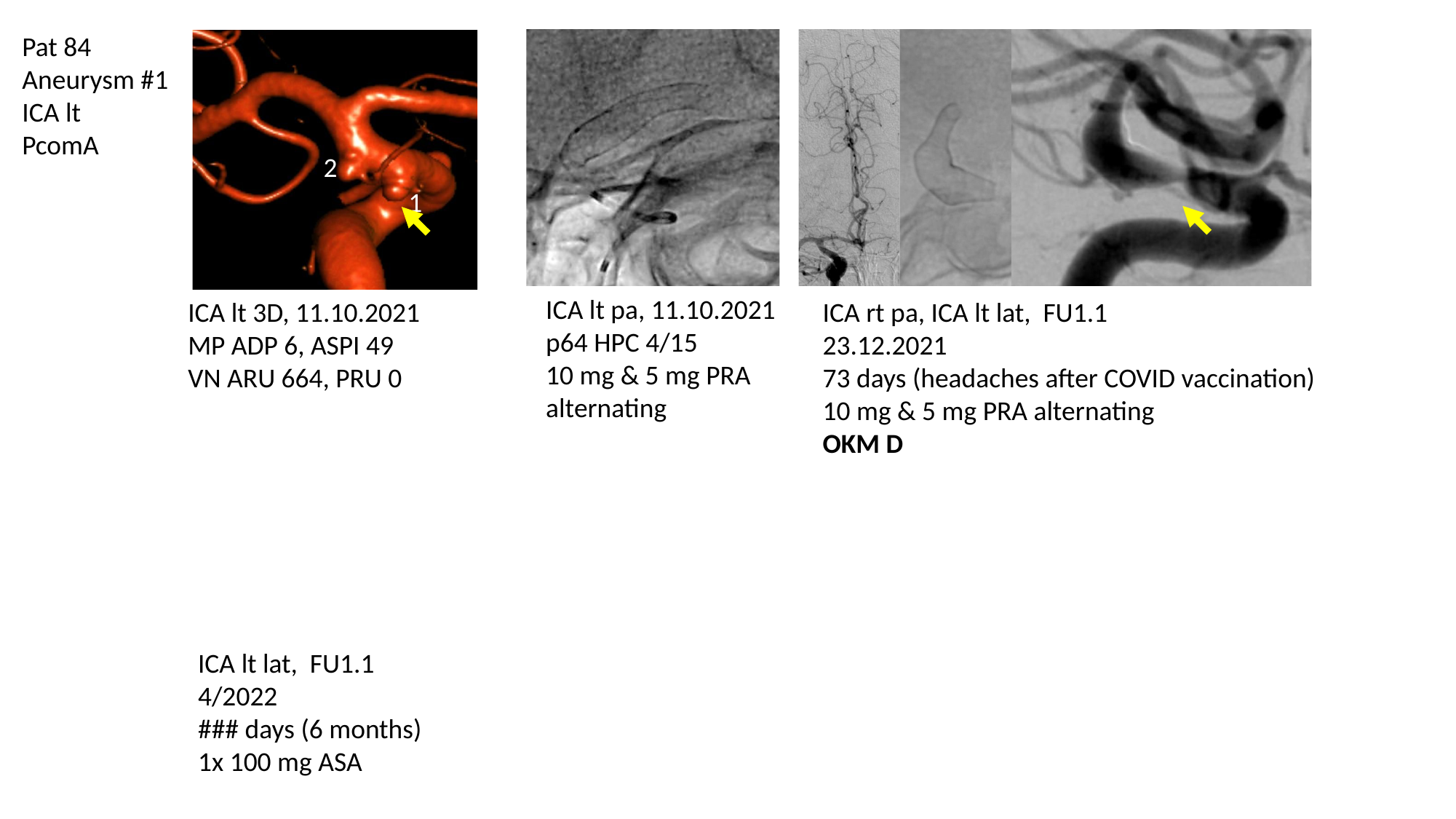

Pat 84
Aneurysm #1
ICA lt
PcomA
M2
2
1
M1
ICA lt pa, 11.10.2021
p64 HPC 4/15
10 mg & 5 mg PRA
alternating
ICA lt 3D, 11.10.2021
MP ADP 6, ASPI 49
VN ARU 664, PRU 0
ICA rt pa, ICA lt lat, FU1.1
23.12.2021
73 days (headaches after COVID vaccination)
10 mg & 5 mg PRA alternating
OKM D
ICA lt lat, FU1.1
4/2022
### days (6 months)
1x 100 mg ASA

## Slide 101
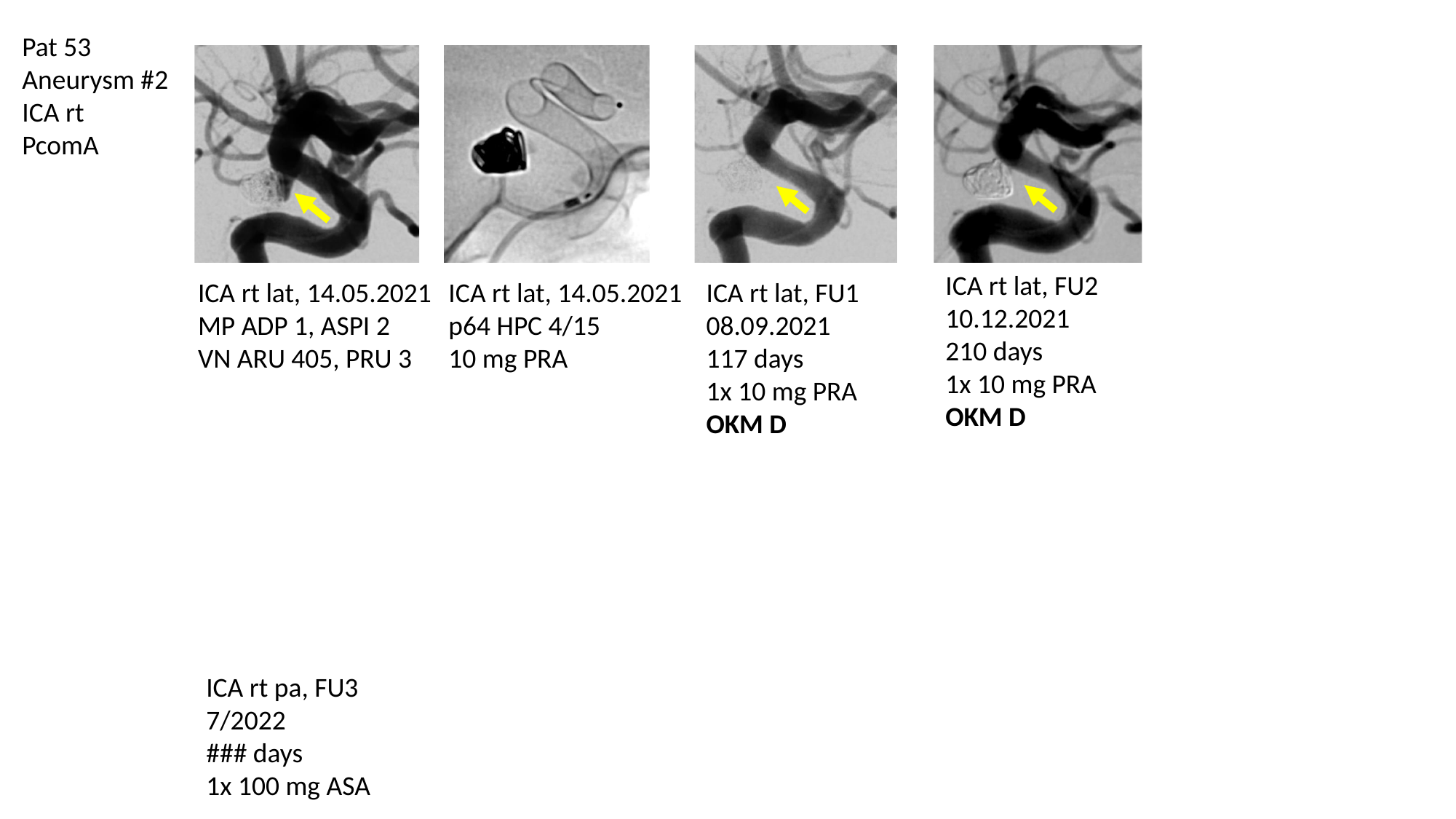

Pat 53
Aneurysm #2
ICA rt
PcomA
ICA rt lat, FU2
10.12.2021
210 days
1x 10 mg PRA
OKM D
ICA rt lat, 14.05.2021
MP ADP 1, ASPI 2
VN ARU 405, PRU 3
ICA rt lat, 14.05.2021
p64 HPC 4/15
10 mg PRA
ICA rt lat, FU1
08.09.2021
117 days
1x 10 mg PRA
OKM D
ICA rt pa, FU3
7/2022
### days
1x 100 mg ASA

## Slide 102
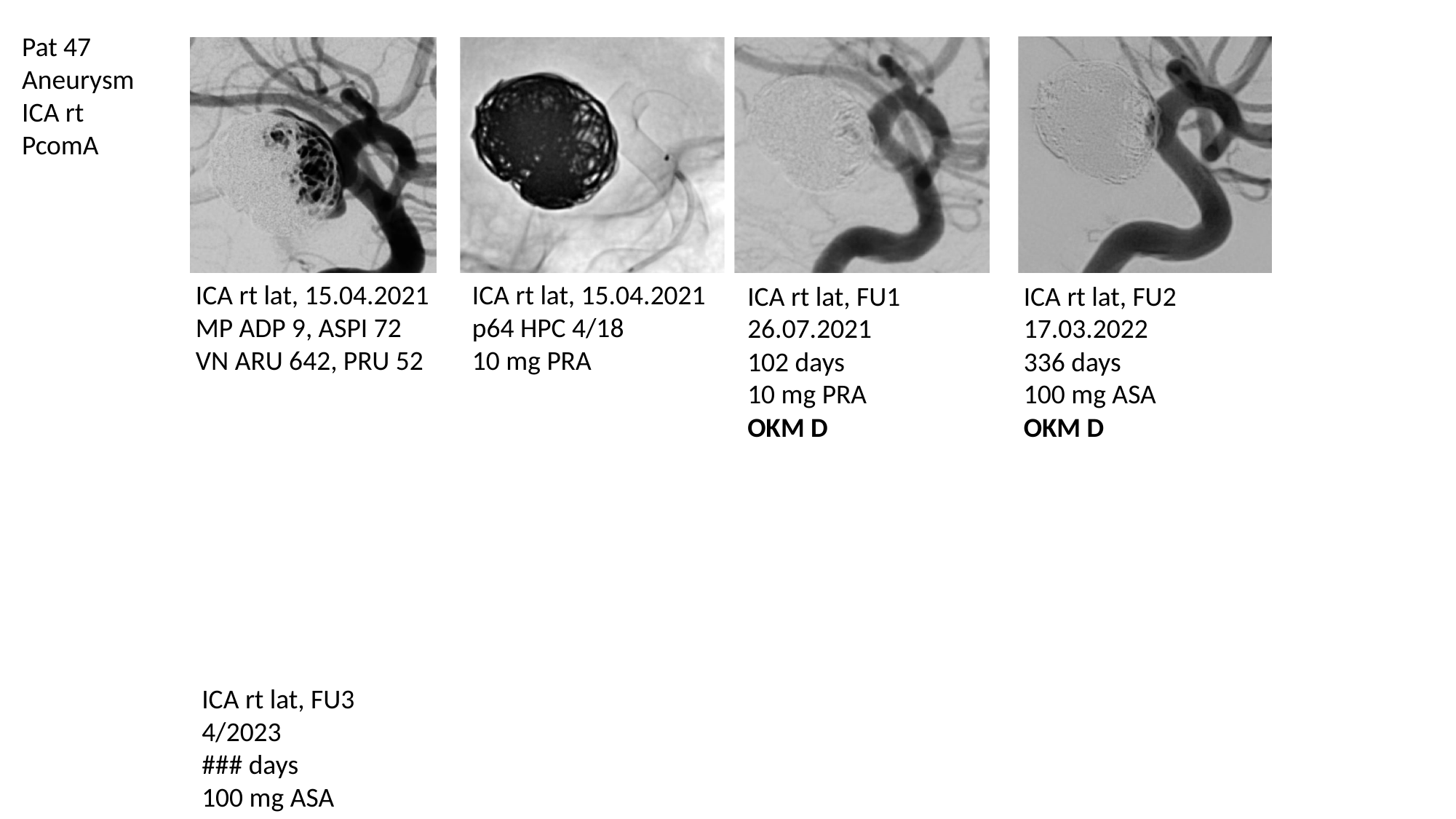

Pat 47
Aneurysm
ICA rt
PcomA
ICA rt lat, 15.04.2021
MP ADP 9, ASPI 72
VN ARU 642, PRU 52
ICA rt lat, 15.04.2021
p64 HPC 4/18
10 mg PRA
ICA rt lat, FU2
17.03.2022
336 days
100 mg ASA
OKM D
ICA rt lat, FU1
26.07.2021
102 days
10 mg PRA
OKM D
ICA rt lat, FU3
4/2023
### days
100 mg ASA

## Slide 103
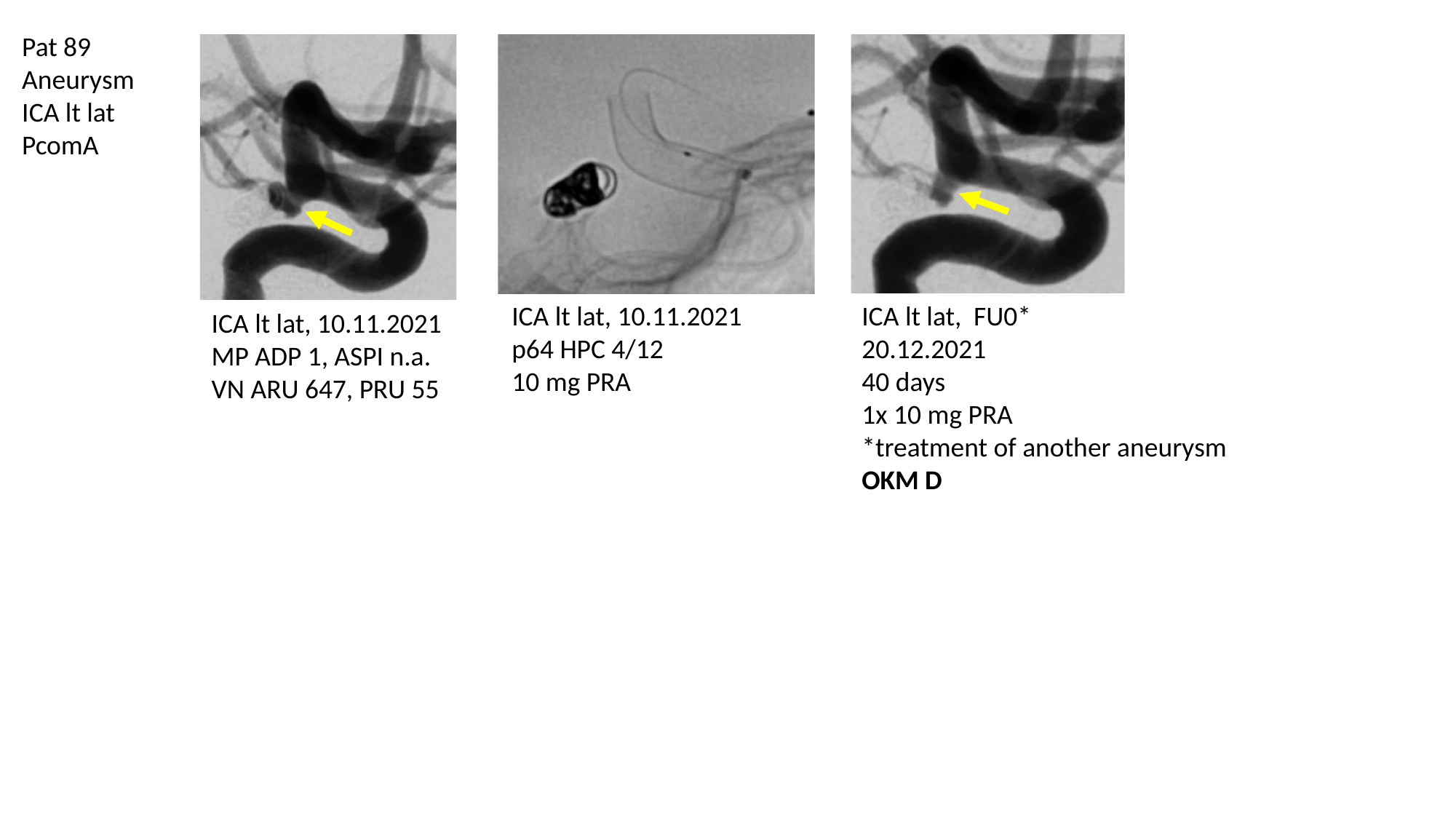

Pat 89
Aneurysm
ICA lt lat
PcomA
ICA lt lat, 10.11.2021
p64 HPC 4/12
10 mg PRA
ICA lt lat, FU0*
20.12.2021
40 days
1x 10 mg PRA
*treatment of another aneurysm
OKM D
ICA lt lat, 10.11.2021
MP ADP 1, ASPI n.a.
VN ARU 647, PRU 55

## Slide 104
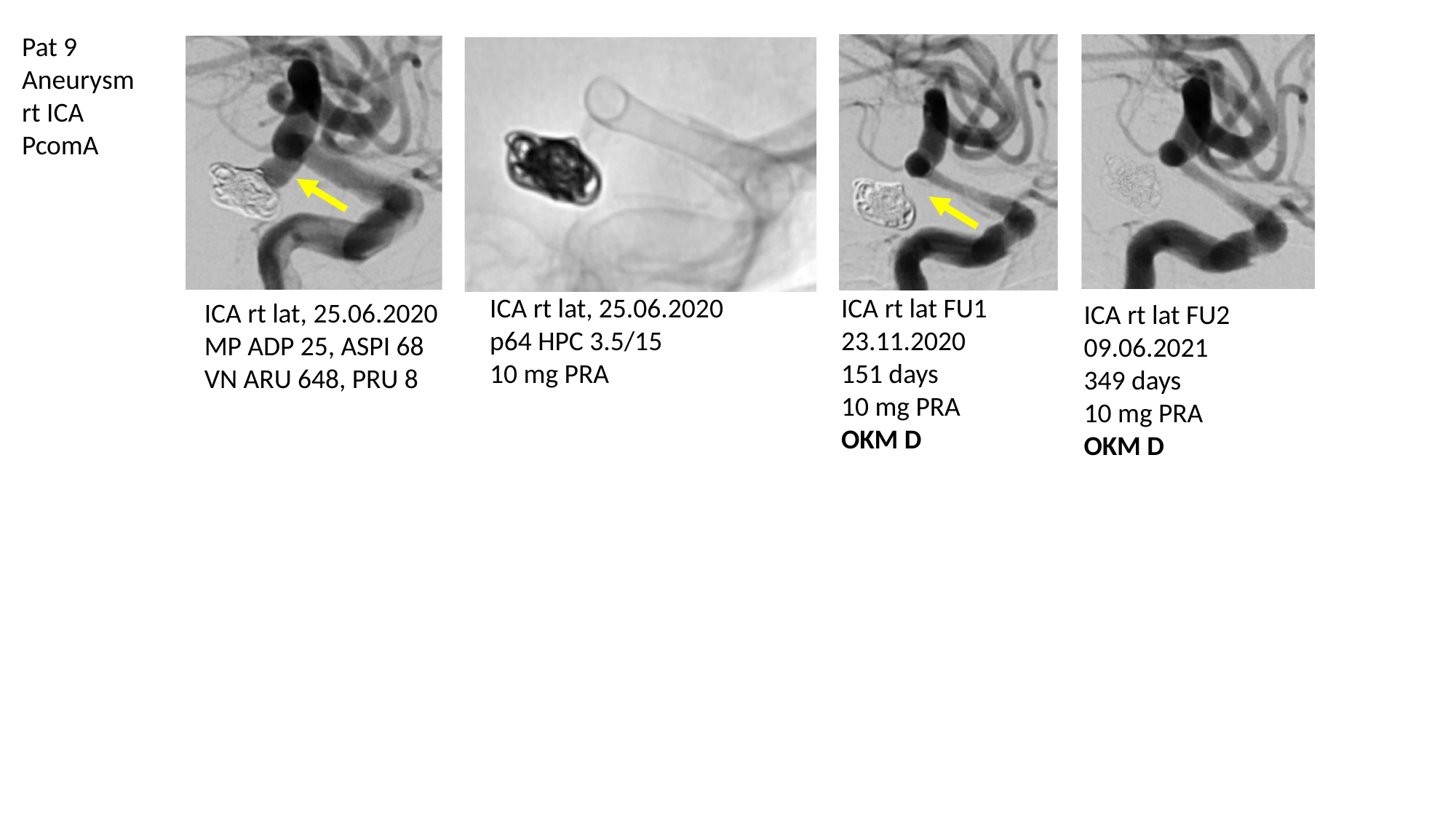

Pat 9
Aneurysm
rt ICA
PcomA
ICA rt lat, 25.06.2020
p64 HPC 3.5/15
10 mg PRA
ICA rt lat FU1
23.11.2020
151 days
10 mg PRA
OKM D
ICA rt lat, 25.06.2020
MP ADP 25, ASPI 68
VN ARU 648, PRU 8
ICA rt lat FU2
09.06.2021
349 days
10 mg PRA
OKM D

## Slide 105
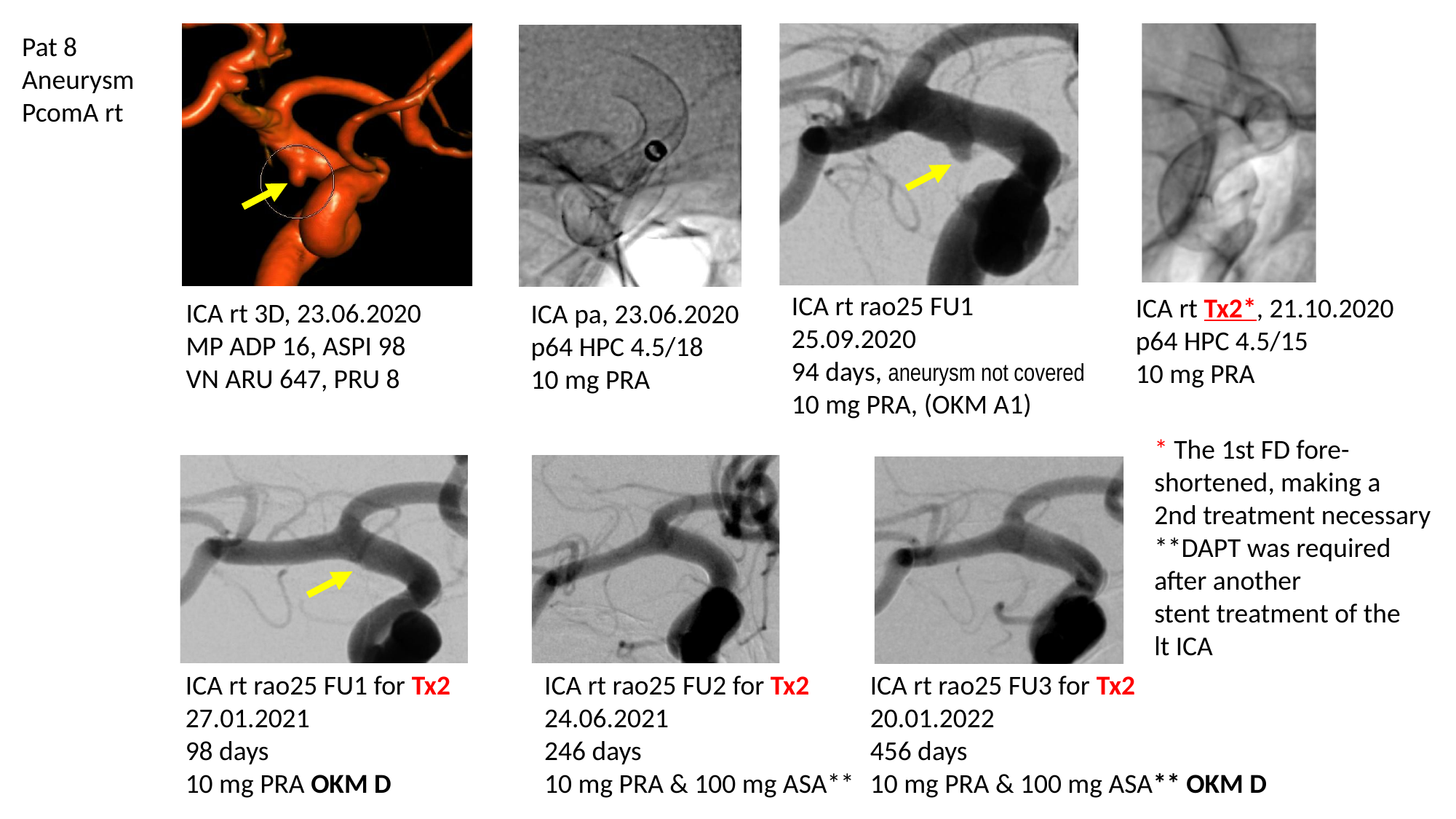

Pat 8
Aneurysm
PcomA rt
ICA rt rao25 FU1
25.09.2020
94 days, aneurysm not covered
10 mg PRA, (OKM A1)
ICA rt Tx2*, 21.10.2020
p64 HPC 4.5/15
10 mg PRA
ICA rt 3D, 23.06.2020
MP ADP 16, ASPI 98
VN ARU 647, PRU 8
ICA pa, 23.06.2020
p64 HPC 4.5/18
10 mg PRA
* The 1st FD fore-
shortened, making a
2nd treatment necessary
**DAPT was required
after another
stent treatment of the
lt ICA
ICA rt rao25 FU2 for Tx2
24.06.2021
246 days
10 mg PRA & 100 mg ASA**
ICA rt rao25 FU3 for Tx2
20.01.2022
456 days
10 mg PRA & 100 mg ASA** OKM D
ICA rt rao25 FU1 for Tx2
27.01.2021
98 days
10 mg PRA OKM D

## Slide 106
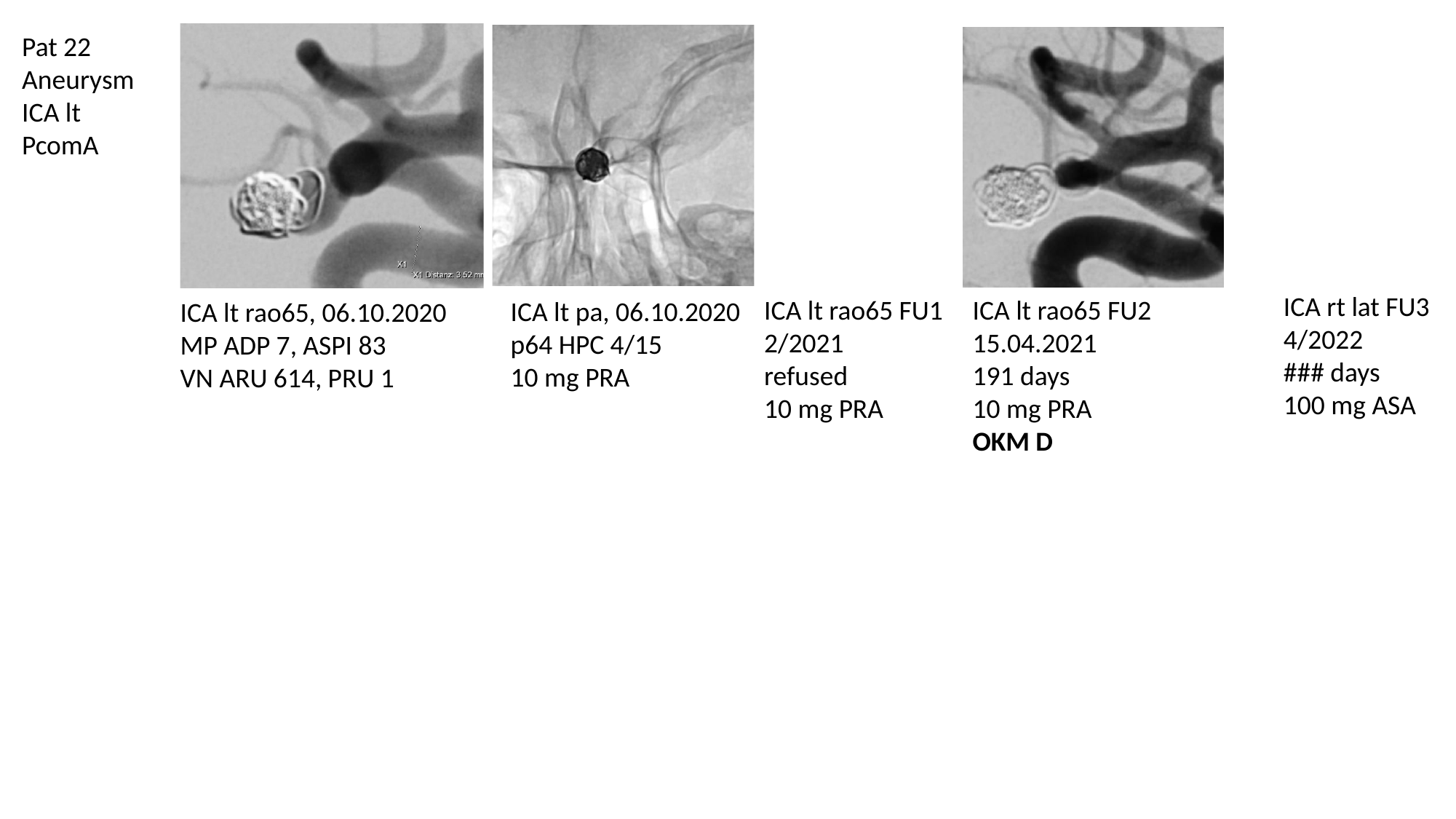

Pat 22
Aneurysm
ICA lt
PcomA
ICA rt lat FU3
4/2022
### days
100 mg ASA
ICA lt rao65 FU1
2/2021
refused
10 mg PRA
ICA lt rao65 FU2
15.04.2021
191 days
10 mg PRA
OKM D
ICA lt pa, 06.10.2020
p64 HPC 4/15
10 mg PRA
ICA lt rao65, 06.10.2020
MP ADP 7, ASPI 83
VN ARU 614, PRU 1

## Slide 107
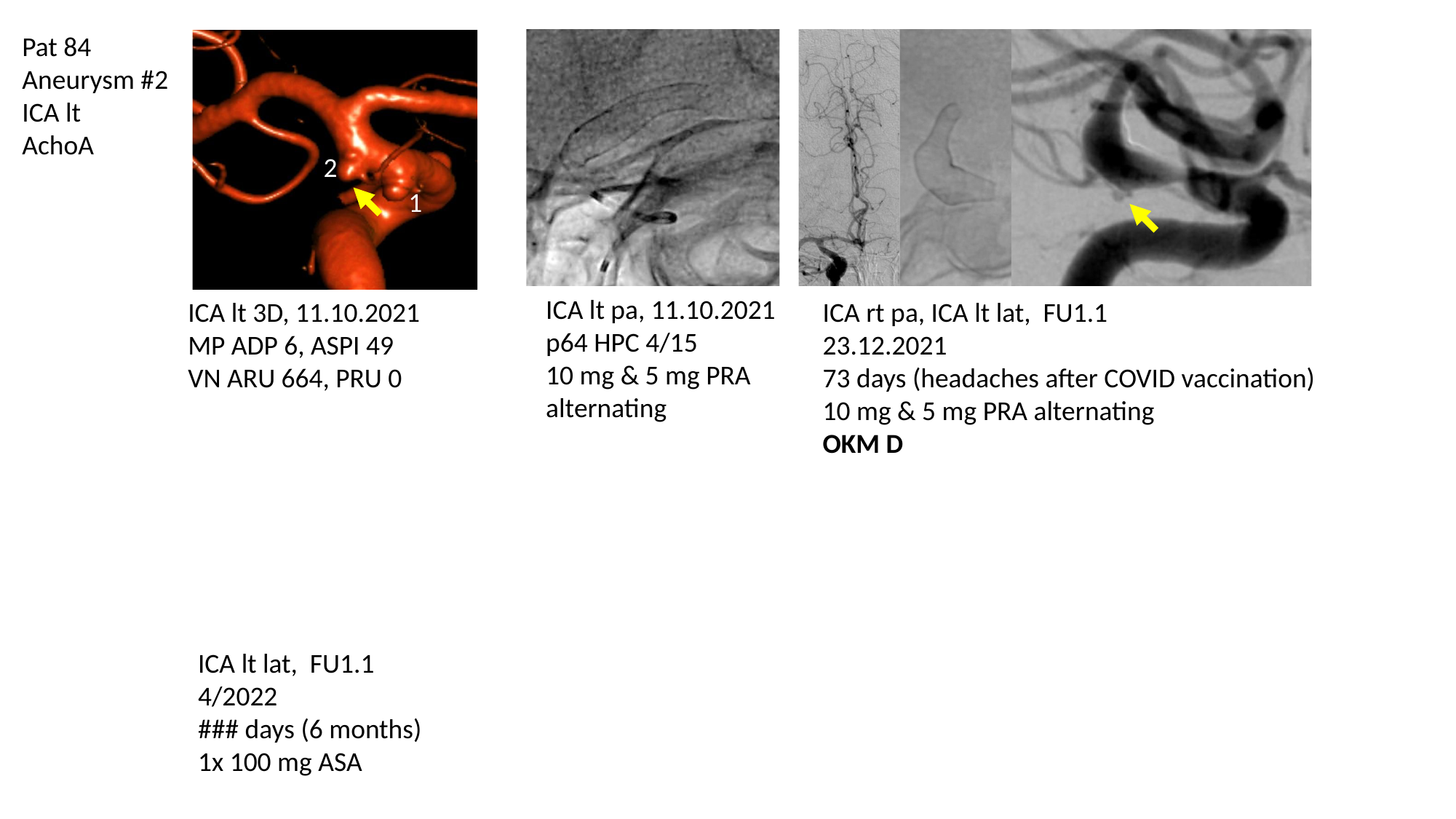

Pat 84
Aneurysm #2
ICA lt
AchoA
M2
2
1
M1
ICA lt pa, 11.10.2021
p64 HPC 4/15
10 mg & 5 mg PRA
alternating
ICA lt 3D, 11.10.2021
MP ADP 6, ASPI 49
VN ARU 664, PRU 0
ICA rt pa, ICA lt lat, FU1.1
23.12.2021
73 days (headaches after COVID vaccination)
10 mg & 5 mg PRA alternating
OKM D
ICA lt lat, FU1.1
4/2022
### days (6 months)
1x 100 mg ASA

## Slide 108
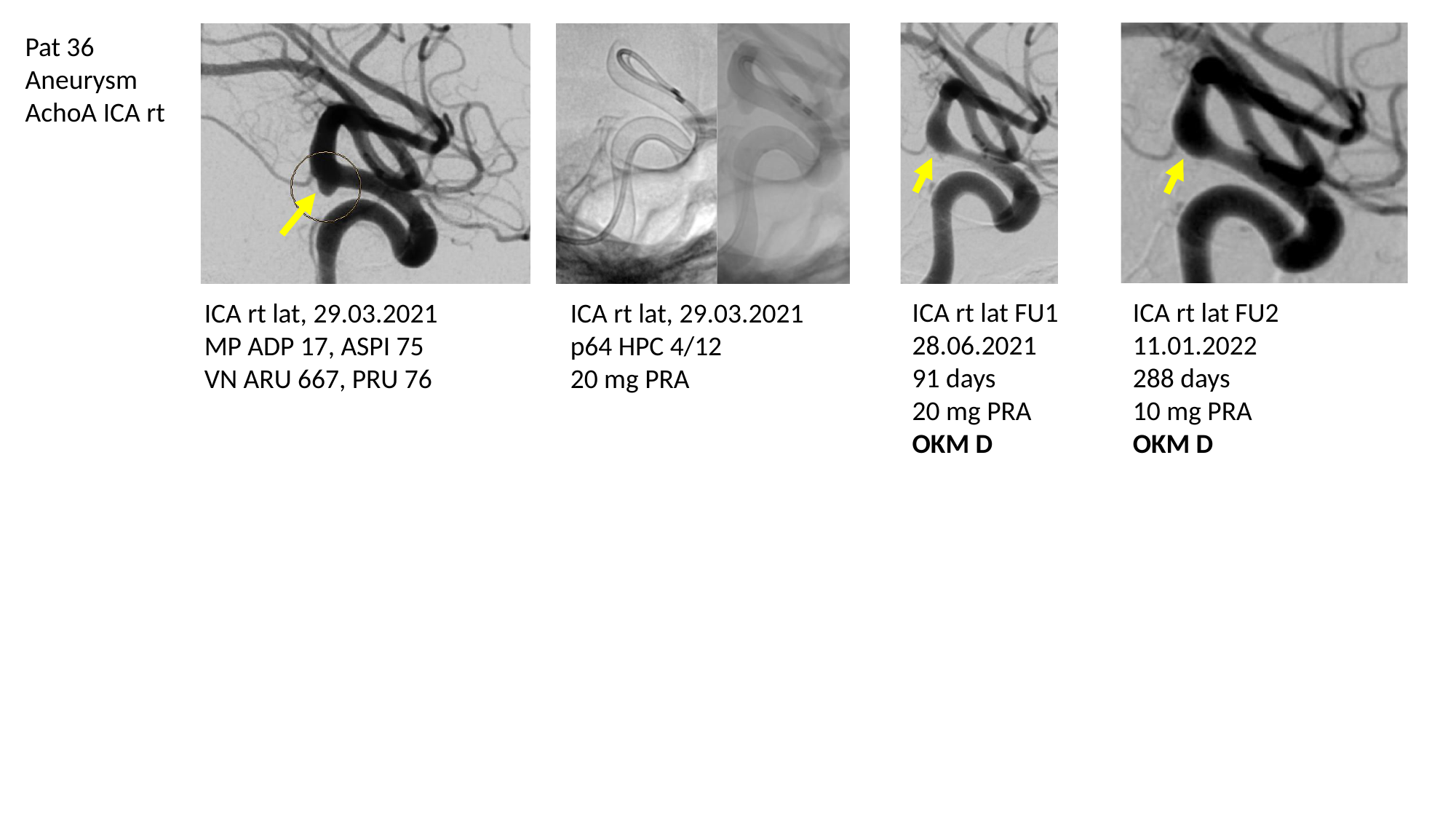

Pat 36
Aneurysm
AchoA ICA rt
ICA rt lat FU1
28.06.2021
91 days
20 mg PRA
OKM D
ICA rt lat FU2
11.01.2022
288 days
10 mg PRA
OKM D
ICA rt lat, 29.03.2021
MP ADP 17, ASPI 75
VN ARU 667, PRU 76
ICA rt lat, 29.03.2021
p64 HPC 4/12
20 mg PRA

## Slide 109
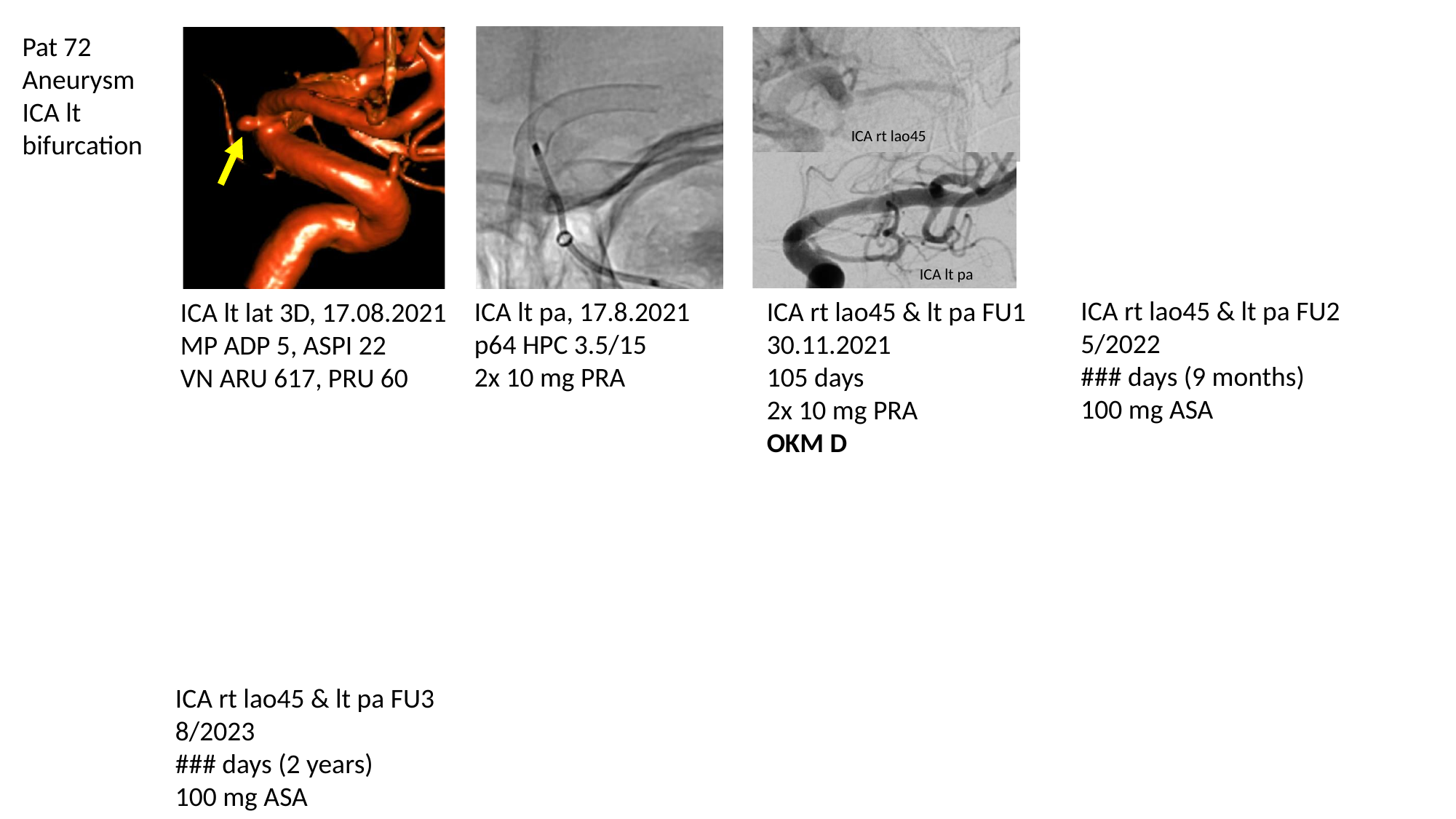

Pat 72
Aneurysm
ICA lt
bifurcation
ICA rt lao45
ICA lt pa
ICA rt lao45 & lt pa FU2
5/2022
### days (9 months)
100 mg ASA
ICA rt lao45 & lt pa FU1
30.11.2021
105 days
2x 10 mg PRA
OKM D
ICA lt pa, 17.8.2021
p64 HPC 3.5/15
2x 10 mg PRA
ICA lt lat 3D, 17.08.2021
MP ADP 5, ASPI 22
VN ARU 617, PRU 60
ICA rt lao45 & lt pa FU3
8/2023
### days (2 years)
100 mg ASA

## Slide 110
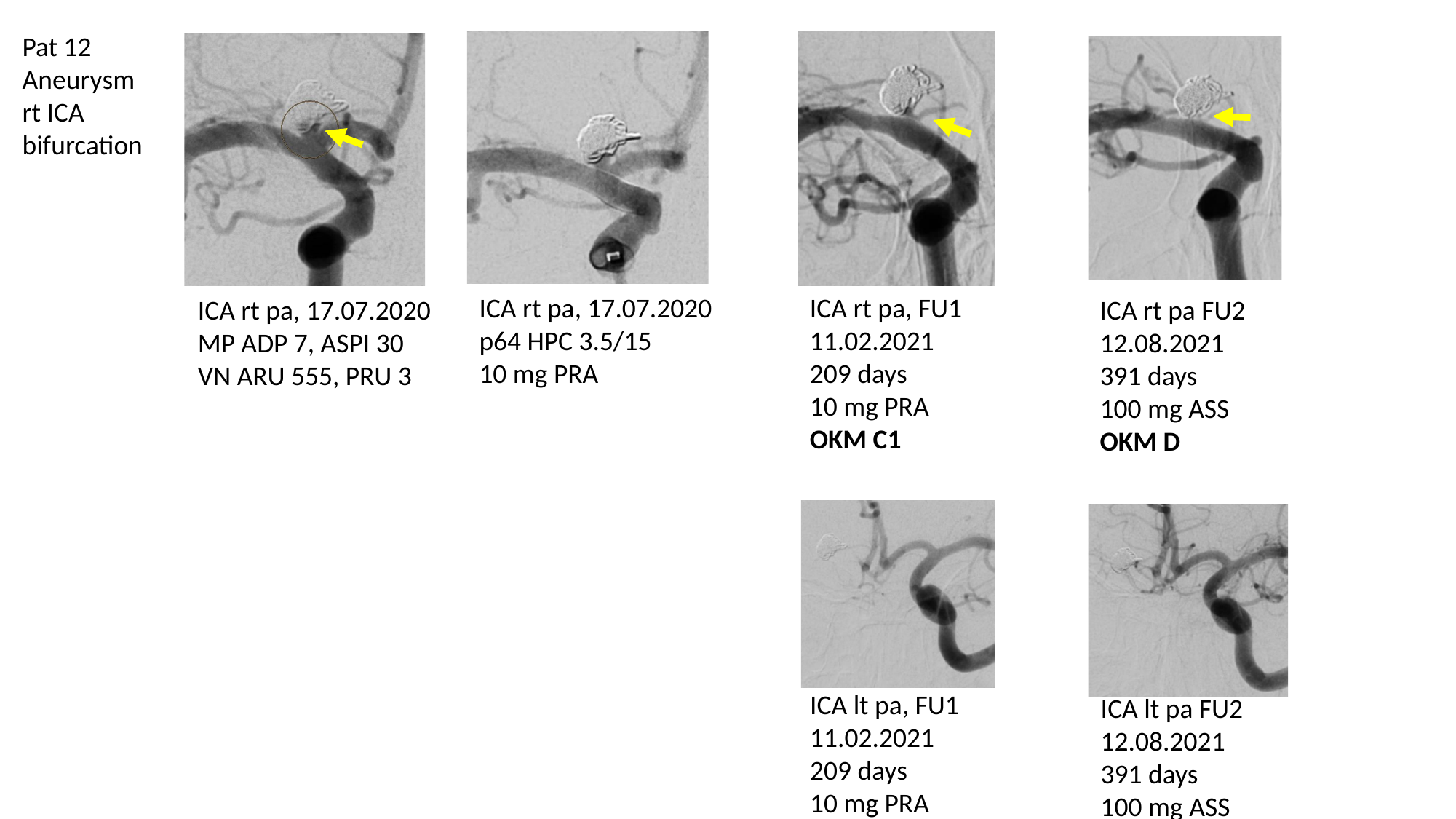

Pat 12
Aneurysm
rt ICA
bifurcation
ICA rt pa, 17.07.2020
p64 HPC 3.5/15
10 mg PRA
ICA rt pa, FU1
11.02.2021
209 days
10 mg PRA
OKM C1
ICA rt pa, 17.07.2020
MP ADP 7, ASPI 30
VN ARU 555, PRU 3
ICA rt pa FU2
12.08.2021
391 days
100 mg ASS
OKM D
ICA lt pa, FU1
11.02.2021
209 days
10 mg PRA
ICA lt pa FU2
12.08.2021
391 days
100 mg ASS

## Slide 111
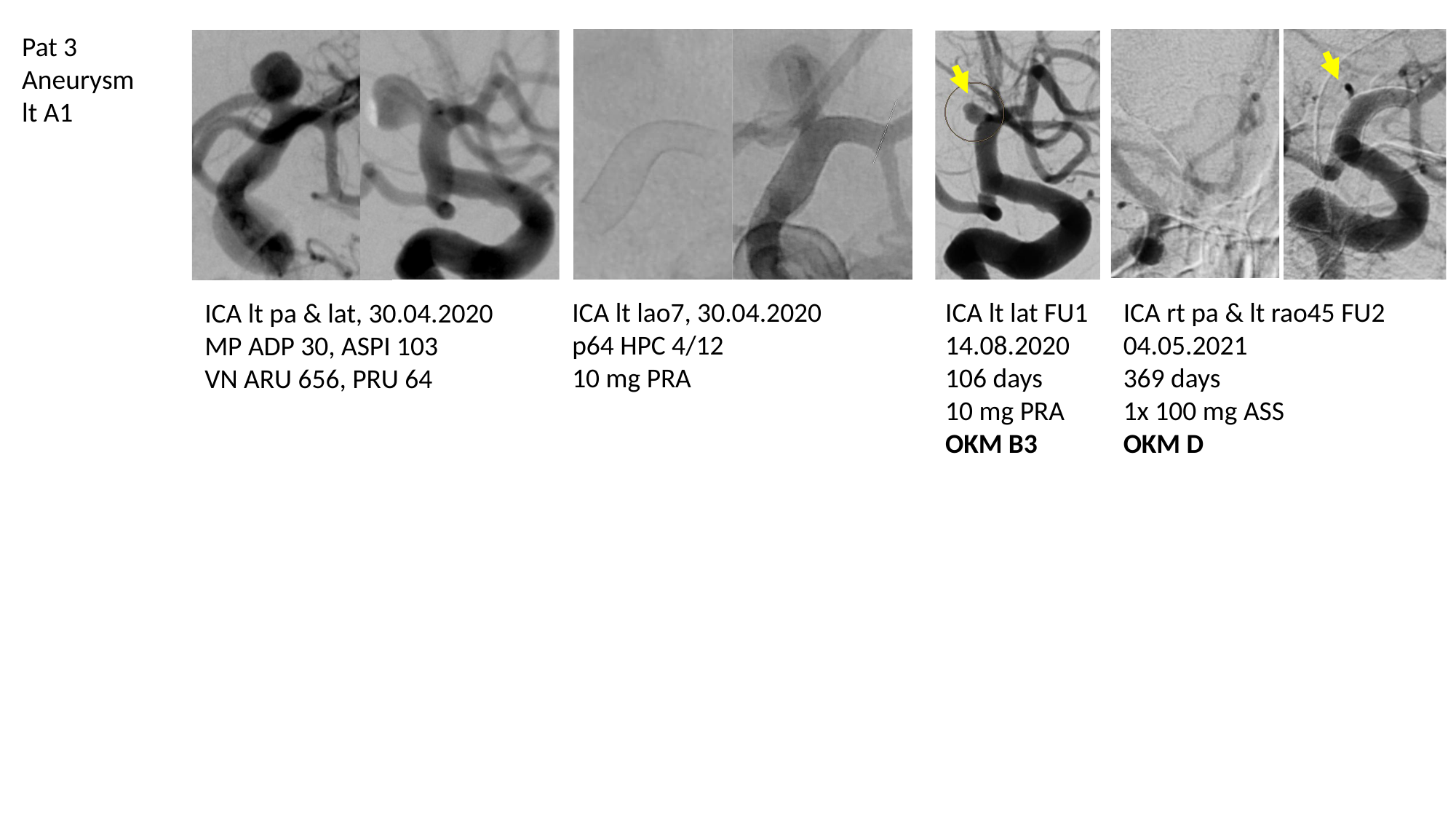

Pat 3
Aneurysm
lt A1
ICA lt lat FU1
14.08.2020
106 days
10 mg PRA
OKM B3
ICA rt pa & lt rao45 FU2
04.05.2021
369 days
1x 100 mg ASS
OKM D
ICA lt lao7, 30.04.2020
p64 HPC 4/12
10 mg PRA
ICA lt pa & lat, 30.04.2020
MP ADP 30, ASPI 103
VN ARU 656, PRU 64

## Slide 112
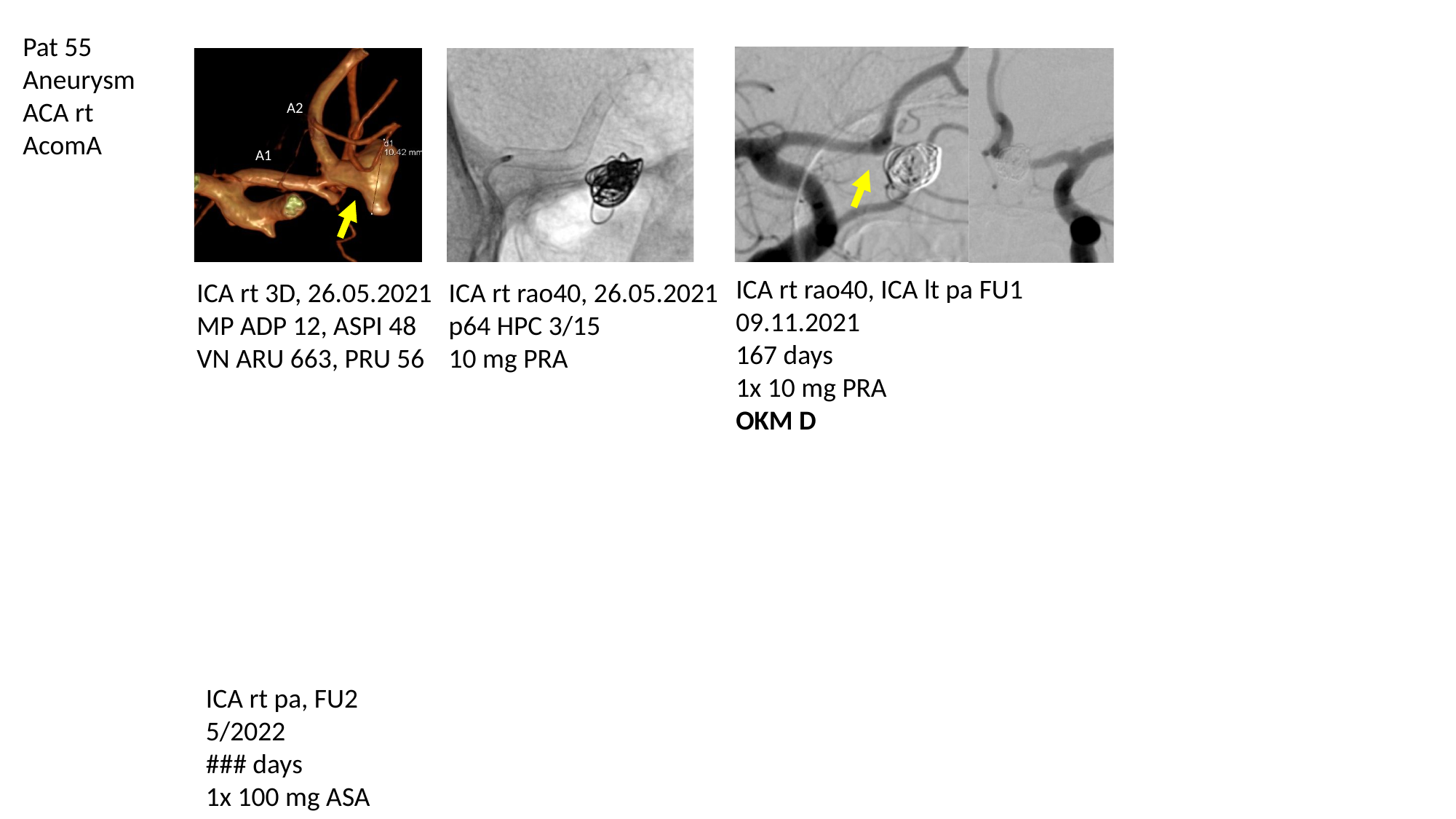

Pat 55
Aneurysm
ACA rt
AcomA
A2
A1
ICA rt rao40, ICA lt pa FU1
09.11.2021
167 days
1x 10 mg PRA
OKM D
ICA rt 3D, 26.05.2021
MP ADP 12, ASPI 48
VN ARU 663, PRU 56
ICA rt rao40, 26.05.2021
p64 HPC 3/15
10 mg PRA
ICA rt pa, FU2
5/2022
### days
1x 100 mg ASA

## Slide 113
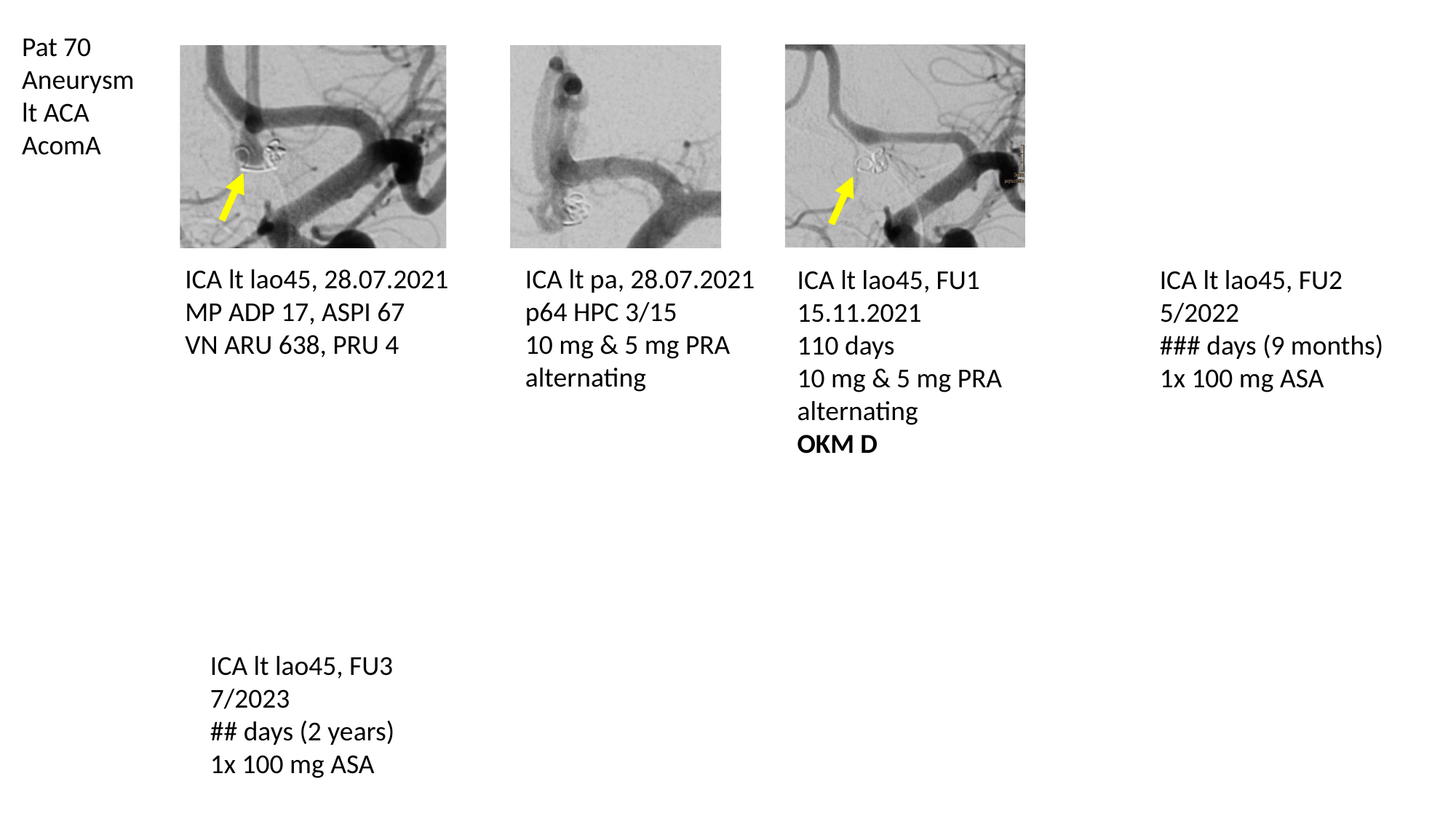

Pat 70
Aneurysm
lt ACA
AcomA
ICA lt pa, 28.07.2021
p64 HPC 3/15
10 mg & 5 mg PRA
alternating
ICA lt lao45, 28.07.2021
MP ADP 17, ASPI 67
VN ARU 638, PRU 4
ICA lt lao45, FU1
15.11.2021
110 days
10 mg & 5 mg PRA
alternating
OKM D
ICA lt lao45, FU2
5/2022
### days (9 months)
1x 100 mg ASA
ICA lt lao45, FU3
7/2023
## days (2 years)
1x 100 mg ASA

## Slide 114
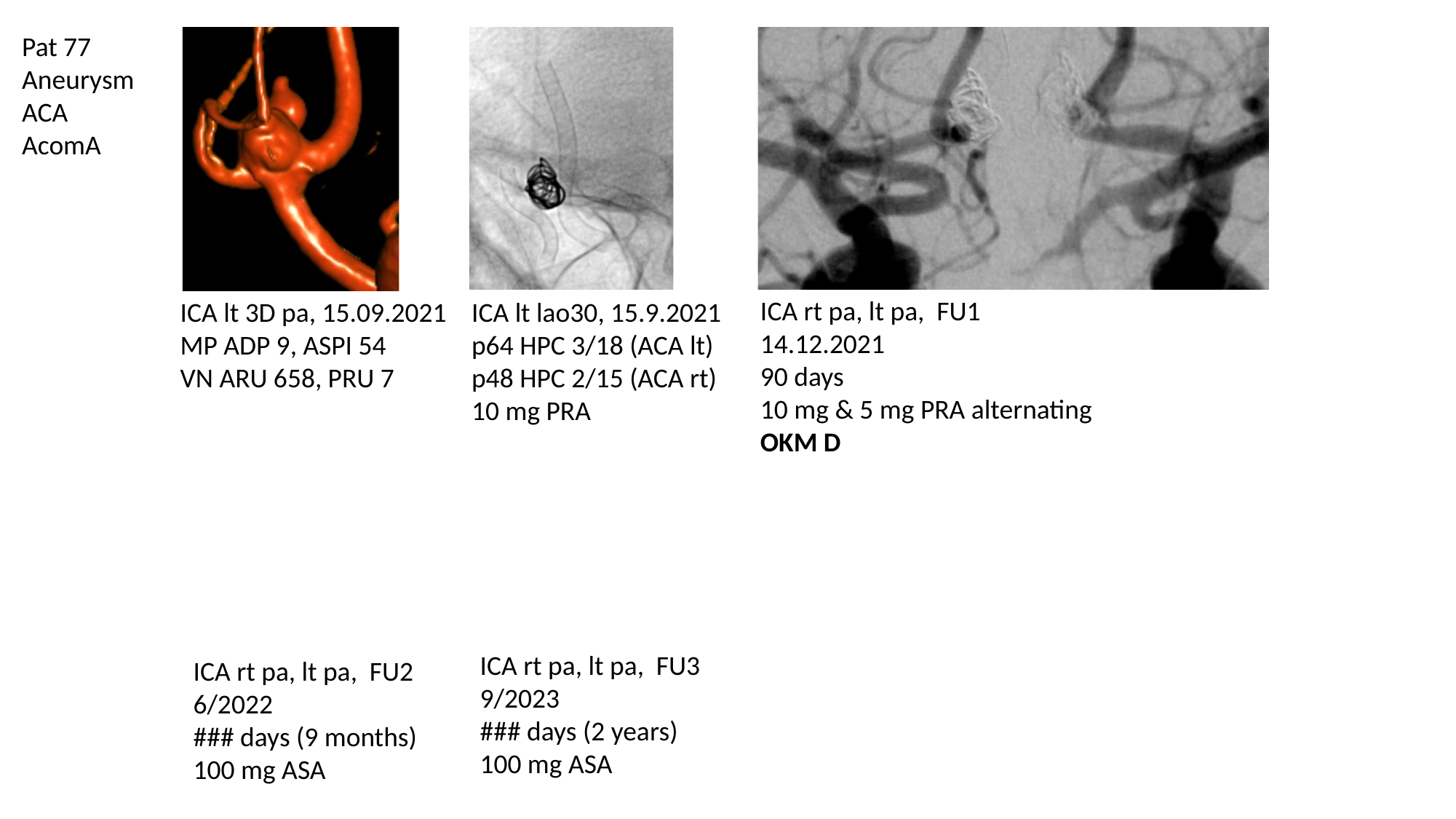

Pat 77
Aneurysm
ACA
AcomA
ICA rt pa, lt pa, FU1
14.12.2021
90 days
10 mg & 5 mg PRA alternating
OKM D
ICA lt 3D pa, 15.09.2021
MP ADP 9, ASPI 54
VN ARU 658, PRU 7
ICA lt lao30, 15.9.2021
p64 HPC 3/18 (ACA lt)
p48 HPC 2/15 (ACA rt)
10 mg PRA
ICA rt pa, lt pa, FU3
9/2023
### days (2 years)
100 mg ASA
ICA rt pa, lt pa, FU2
6/2022
### days (9 months)
100 mg ASA

## Slide 115
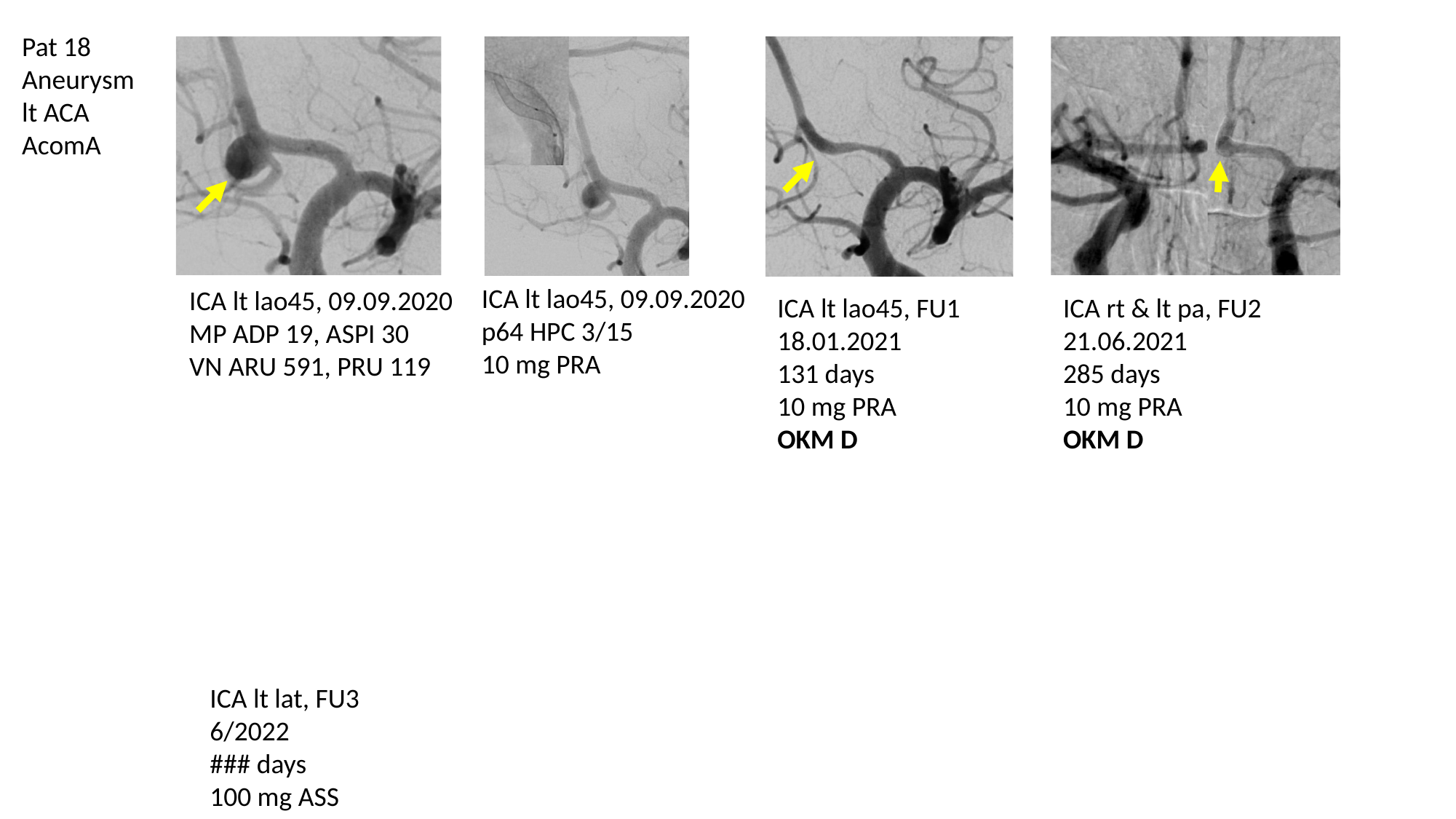

Pat 18
Aneurysm
lt ACA
AcomA
ICA lt lao45, 09.09.2020
p64 HPC 3/15
10 mg PRA
ICA lt lao45, 09.09.2020
MP ADP 19, ASPI 30
VN ARU 591, PRU 119
ICA lt lao45, FU1
18.01.2021
131 days
10 mg PRA
OKM D
ICA rt & lt pa, FU2
21.06.2021
285 days
10 mg PRA
OKM D
ICA lt lat, FU3
6/2022
### days
100 mg ASS

## Slide 116
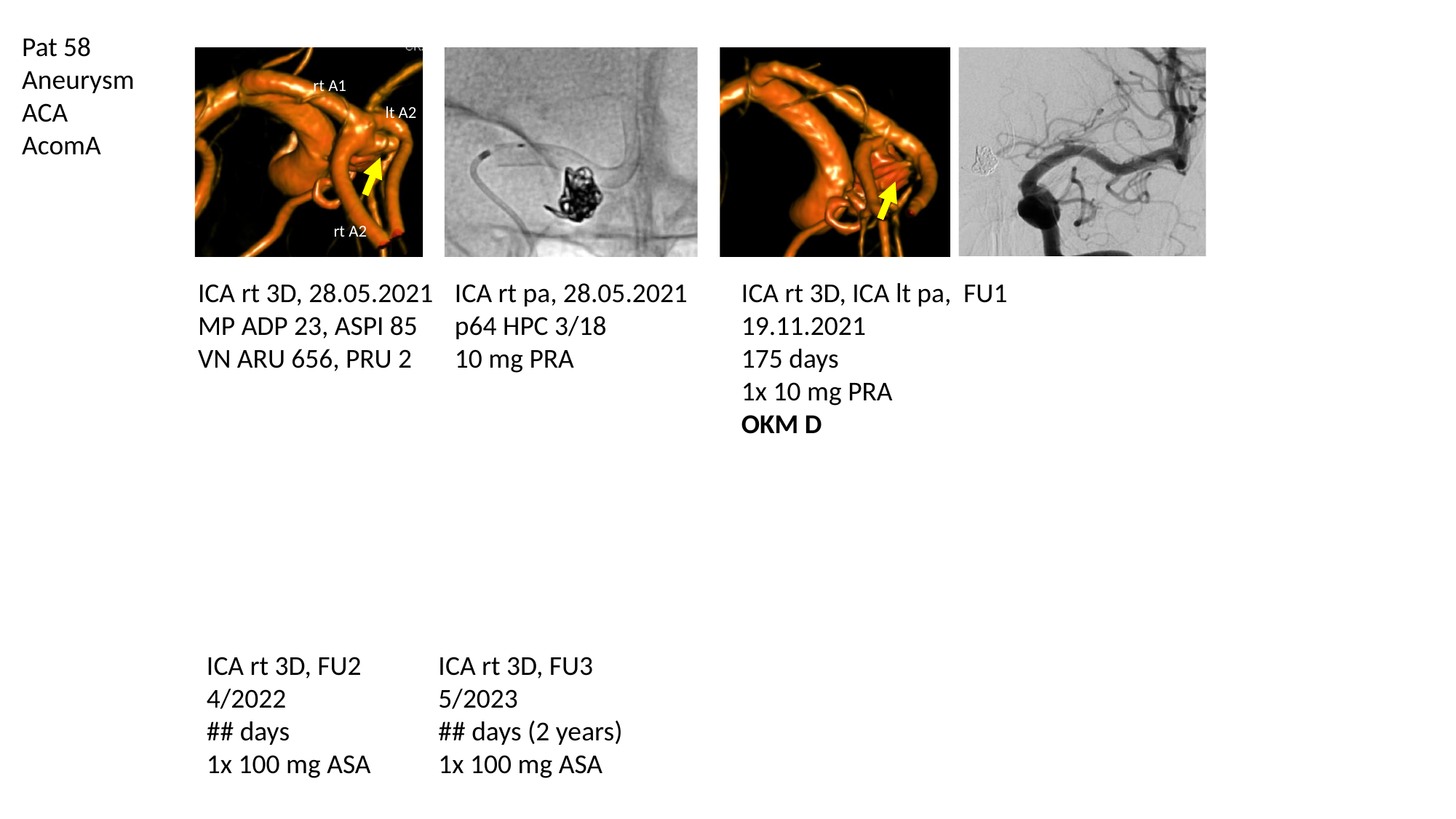

Pat 58
Aneurysm
ACA
AcomA
rt A1
lt A2
rt A2
ICA rt 3D, 28.05.2021
MP ADP 23, ASPI 85
VN ARU 656, PRU 2
ICA rt pa, 28.05.2021
p64 HPC 3/18
10 mg PRA
ICA rt 3D, ICA lt pa, FU1
19.11.2021
175 days
1x 10 mg PRA
OKM D
ICA rt 3D, FU3
5/2023
## days (2 years)
1x 100 mg ASA
ICA rt 3D, FU2
4/2022
## days
1x 100 mg ASA

## Slide 117
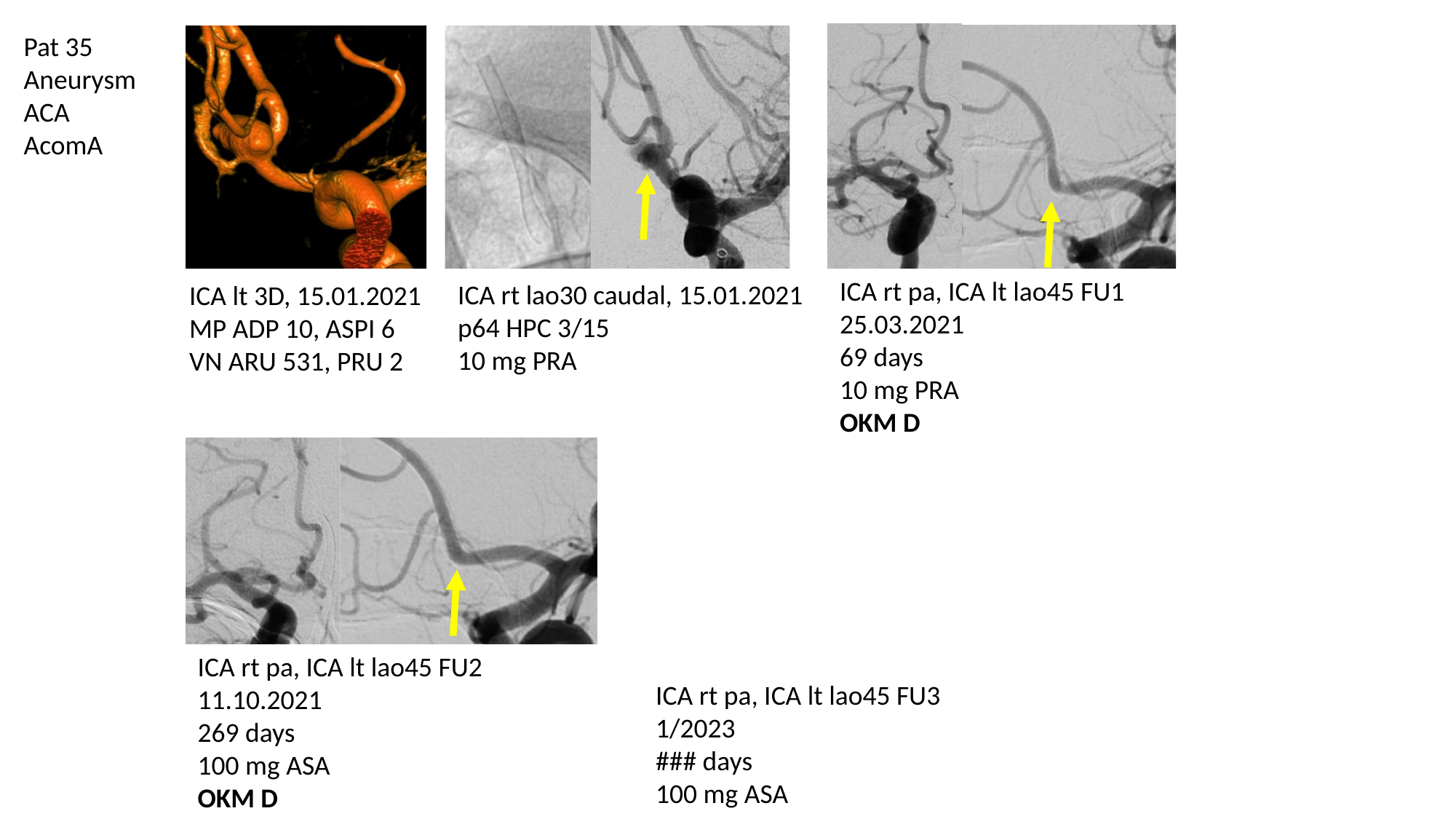

Pat 35
Aneurysm
ACA
AcomA
ICA rt pa, ICA lt lao45 FU1
25.03.2021
69 days
10 mg PRA
OKM D
ICA rt lao30 caudal, 15.01.2021
p64 HPC 3/15
10 mg PRA
ICA lt 3D, 15.01.2021
MP ADP 10, ASPI 6
VN ARU 531, PRU 2
ICA rt pa, ICA lt lao45 FU2
11.10.2021
269 days
100 mg ASA
OKM D
ICA rt pa, ICA lt lao45 FU3
1/2023
### days
100 mg ASA

## Slide 118
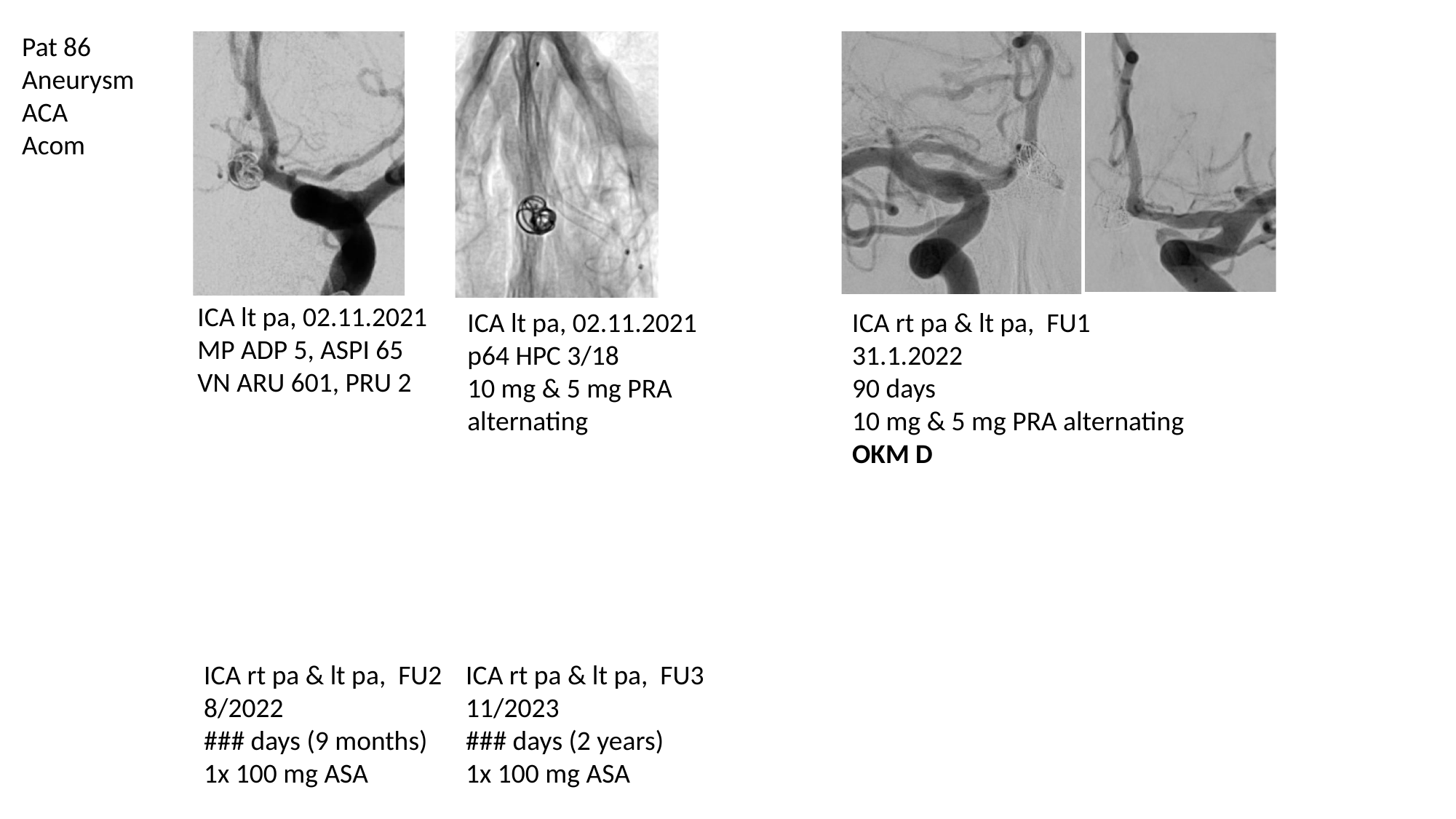

Pat 86
Aneurysm
ACA
Acom
M2
M1
ICA lt pa, 02.11.2021
MP ADP 5, ASPI 65
VN ARU 601, PRU 2
ICA lt pa, 02.11.2021
p64 HPC 3/18
10 mg & 5 mg PRA
alternating
ICA rt pa & lt pa, FU1
31.1.2022
90 days
10 mg & 5 mg PRA alternating
OKM D
ICA rt pa & lt pa, FU2
8/2022
### days (9 months)
1x 100 mg ASA
ICA rt pa & lt pa, FU3
11/2023
### days (2 years)
1x 100 mg ASA

## Slide 119
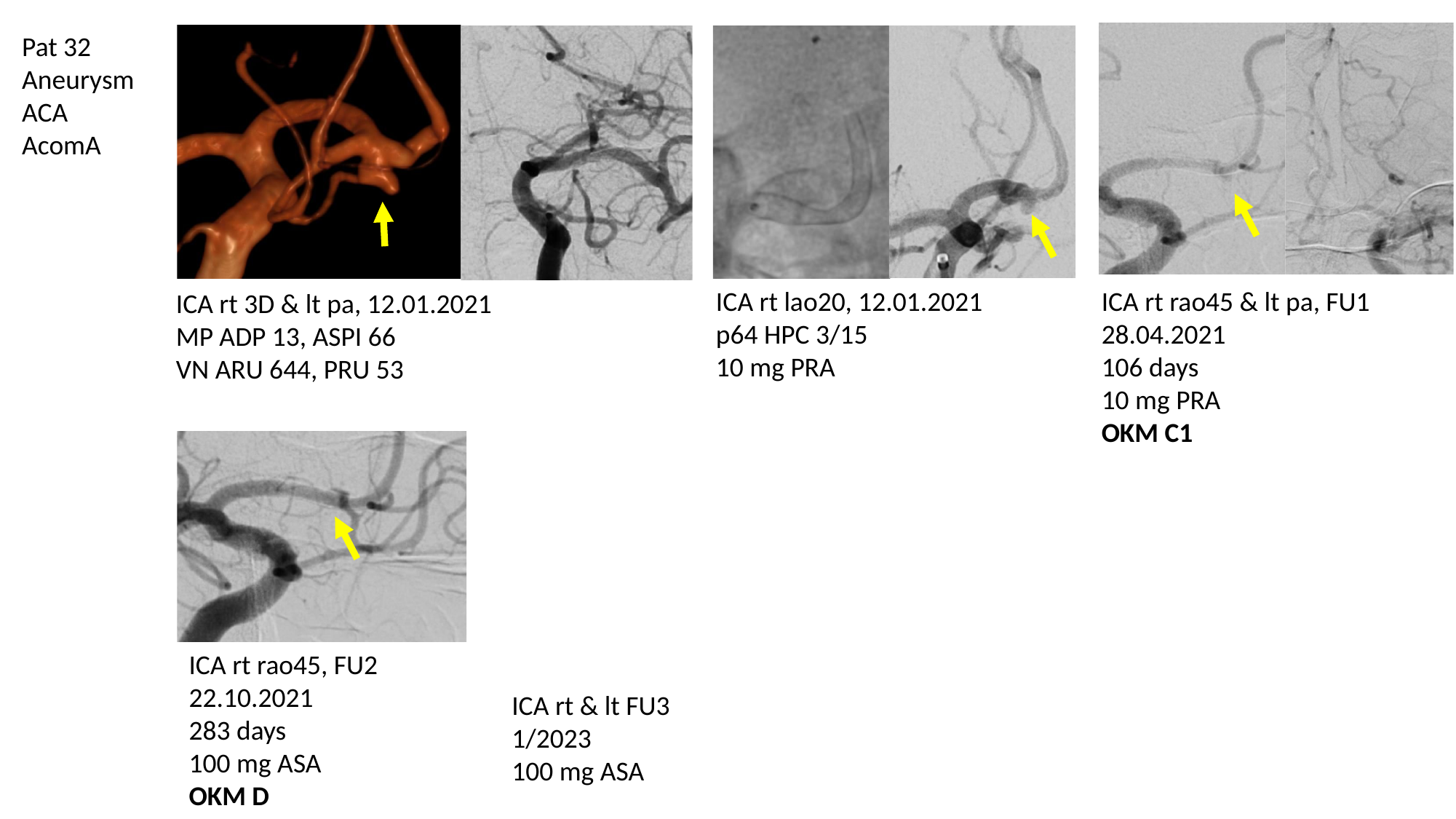

Pat 32
Aneurysm
ACA
AcomA
ICA rt lao20, 12.01.2021
p64 HPC 3/15
10 mg PRA
ICA rt rao45 & lt pa, FU1
28.04.2021
106 days
10 mg PRA
OKM C1
ICA rt 3D & lt pa, 12.01.2021
MP ADP 13, ASPI 66
VN ARU 644, PRU 53
ICA rt rao45, FU2
22.10.2021
283 days
100 mg ASA
OKM D
ICA rt & lt FU3
1/2023
100 mg ASA

## Slide 120
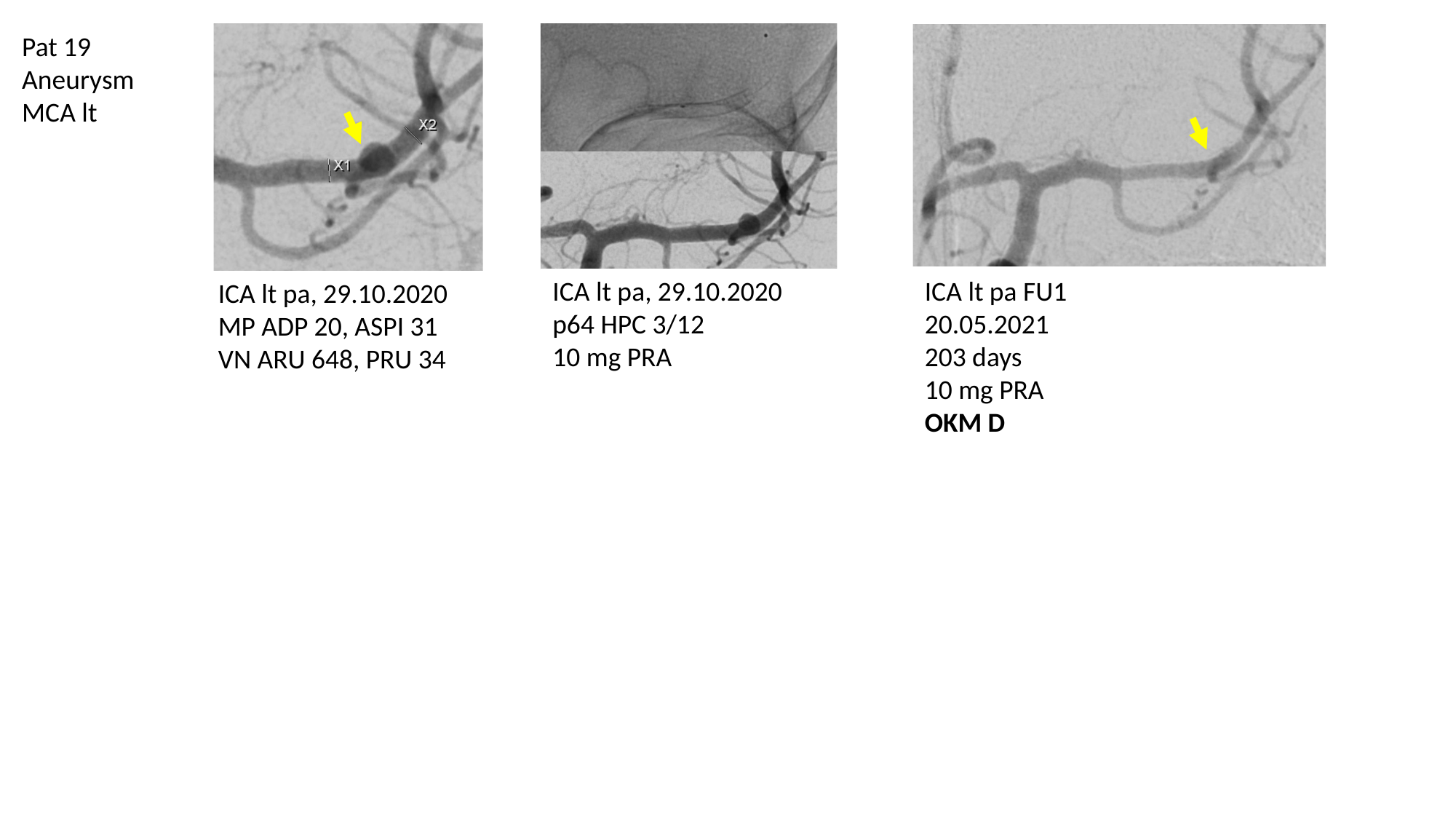

Pat 19
Aneurysm
MCA lt
ICA lt pa, 29.10.2020
p64 HPC 3/12
10 mg PRA
ICA lt pa FU1
20.05.2021
203 days
10 mg PRA
OKM D
ICA lt pa, 29.10.2020
MP ADP 20, ASPI 31
VN ARU 648, PRU 34

## Slide 121
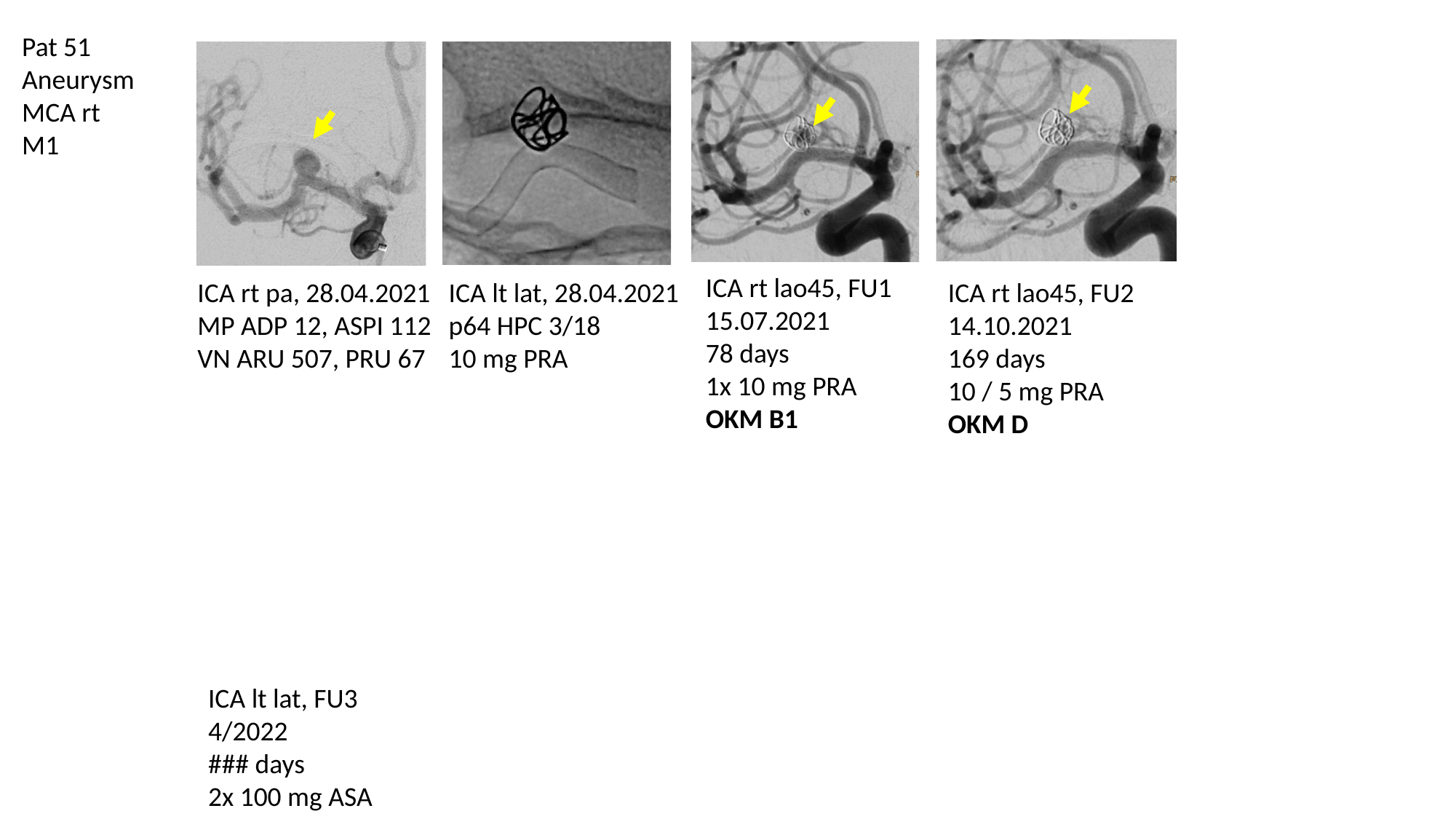

Pat 51
Aneurysm
MCA rt
M1
ICA rt lao45, FU1
15.07.2021
78 days
1x 10 mg PRA
OKM B1
ICA rt pa, 28.04.2021
MP ADP 12, ASPI 112
VN ARU 507, PRU 67
ICA lt lat, 28.04.2021
p64 HPC 3/18
10 mg PRA
ICA rt lao45, FU2
14.10.2021
169 days
10 / 5 mg PRA
OKM D
ICA lt lat, FU3
4/2022
### days
2x 100 mg ASA

## Slide 122
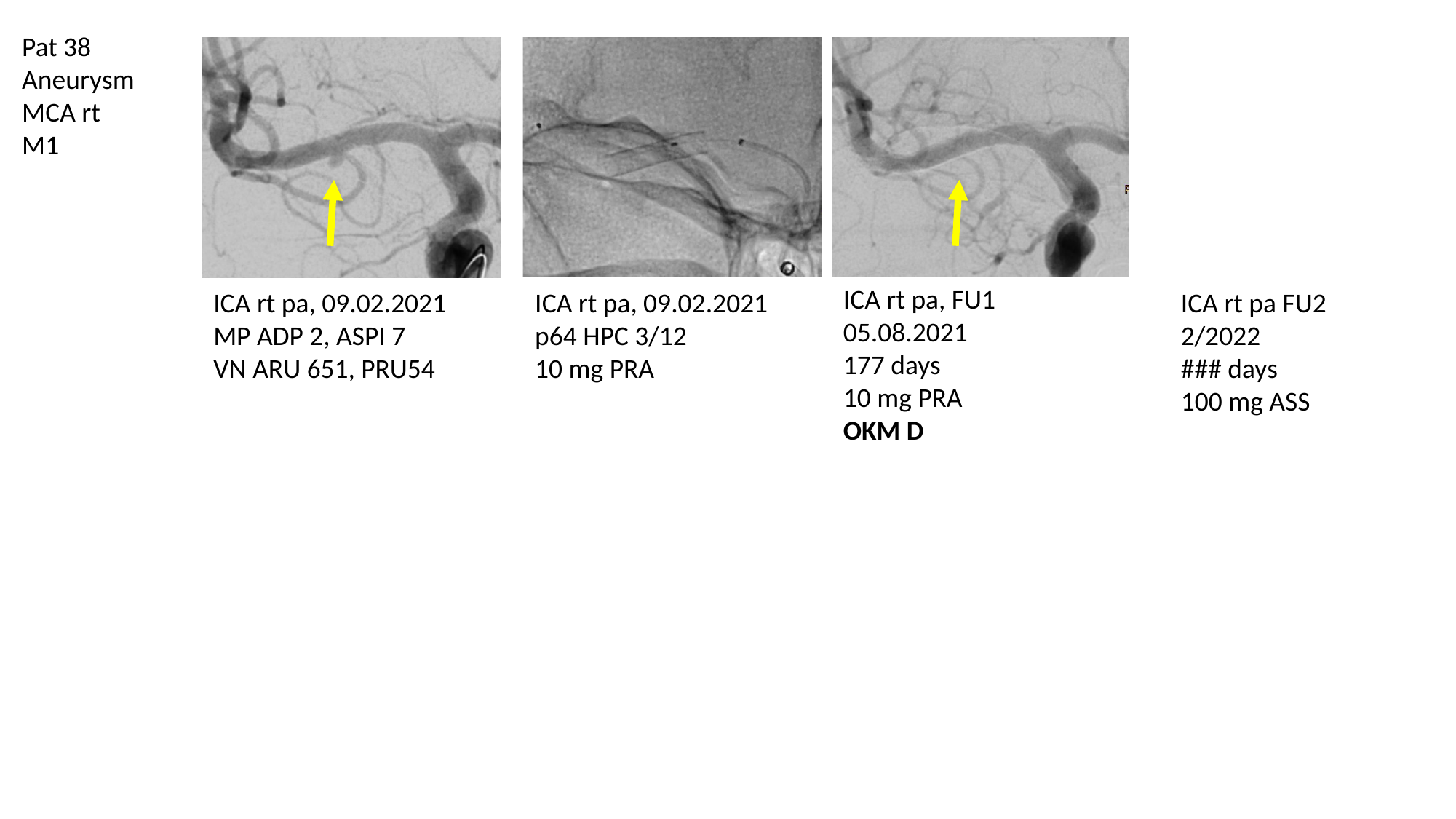

Pat 38
Aneurysm
MCA rt
M1
ICA rt pa, FU1
05.08.2021
177 days
10 mg PRA
OKM D
ICA rt pa, 09.02.2021
MP ADP 2, ASPI 7
VN ARU 651, PRU54
ICA rt pa, 09.02.2021
p64 HPC 3/12
10 mg PRA
ICA rt pa FU2
2/2022
### days
100 mg ASS

## Slide 123
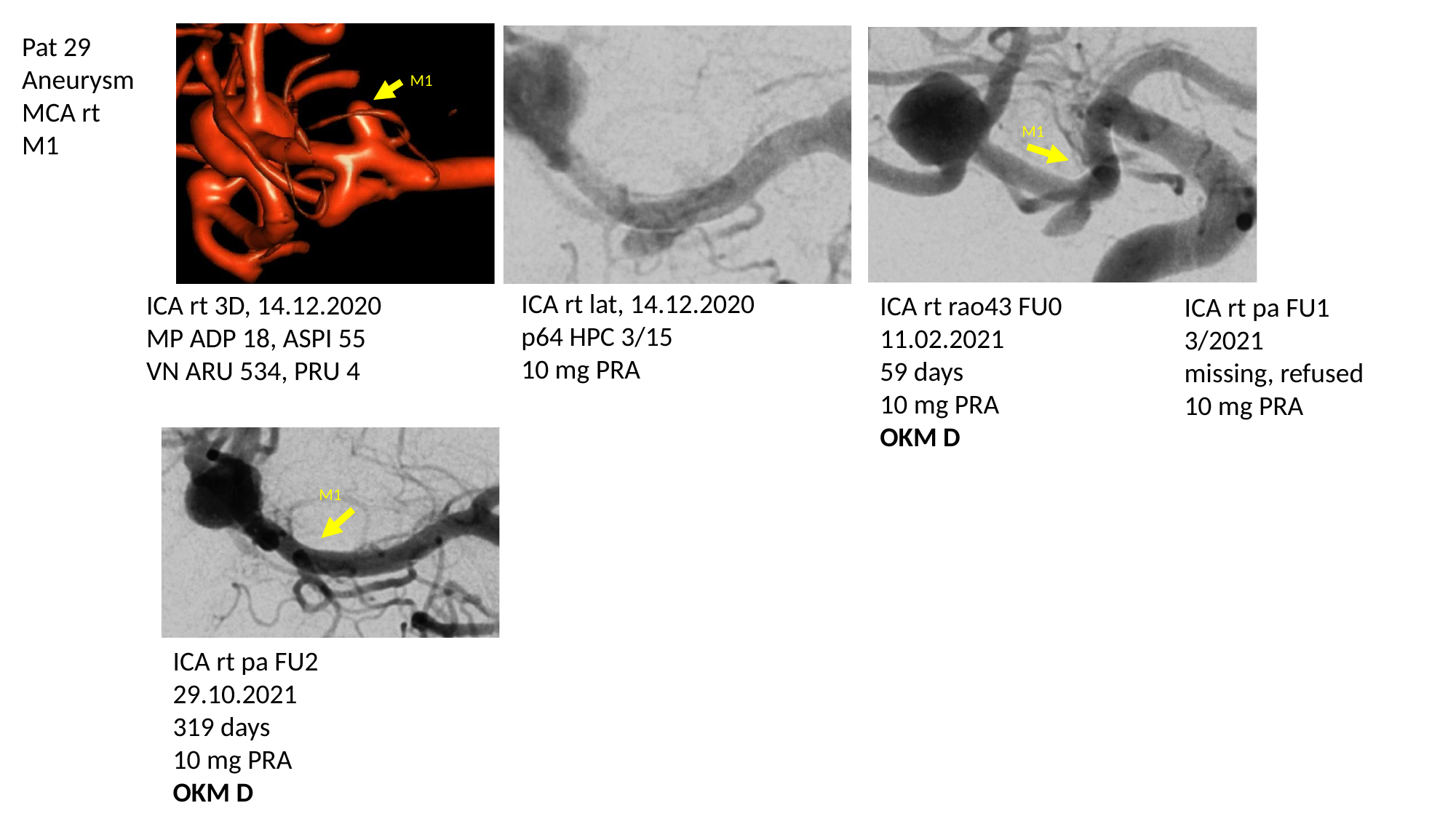

Pat 29
Aneurysm
MCA rt
M1
M1
M1
ICA rt lat, 14.12.2020
p64 HPC 3/15
10 mg PRA
ICA rt 3D, 14.12.2020
MP ADP 18, ASPI 55
VN ARU 534, PRU 4
ICA rt rao43 FU0
11.02.2021
59 days
10 mg PRA
OKM D
ICA rt pa FU1
3/2021
missing, refused
10 mg PRA
M1
ICA rt pa FU2
29.10.2021
319 days
10 mg PRA
OKM D

## Slide 124
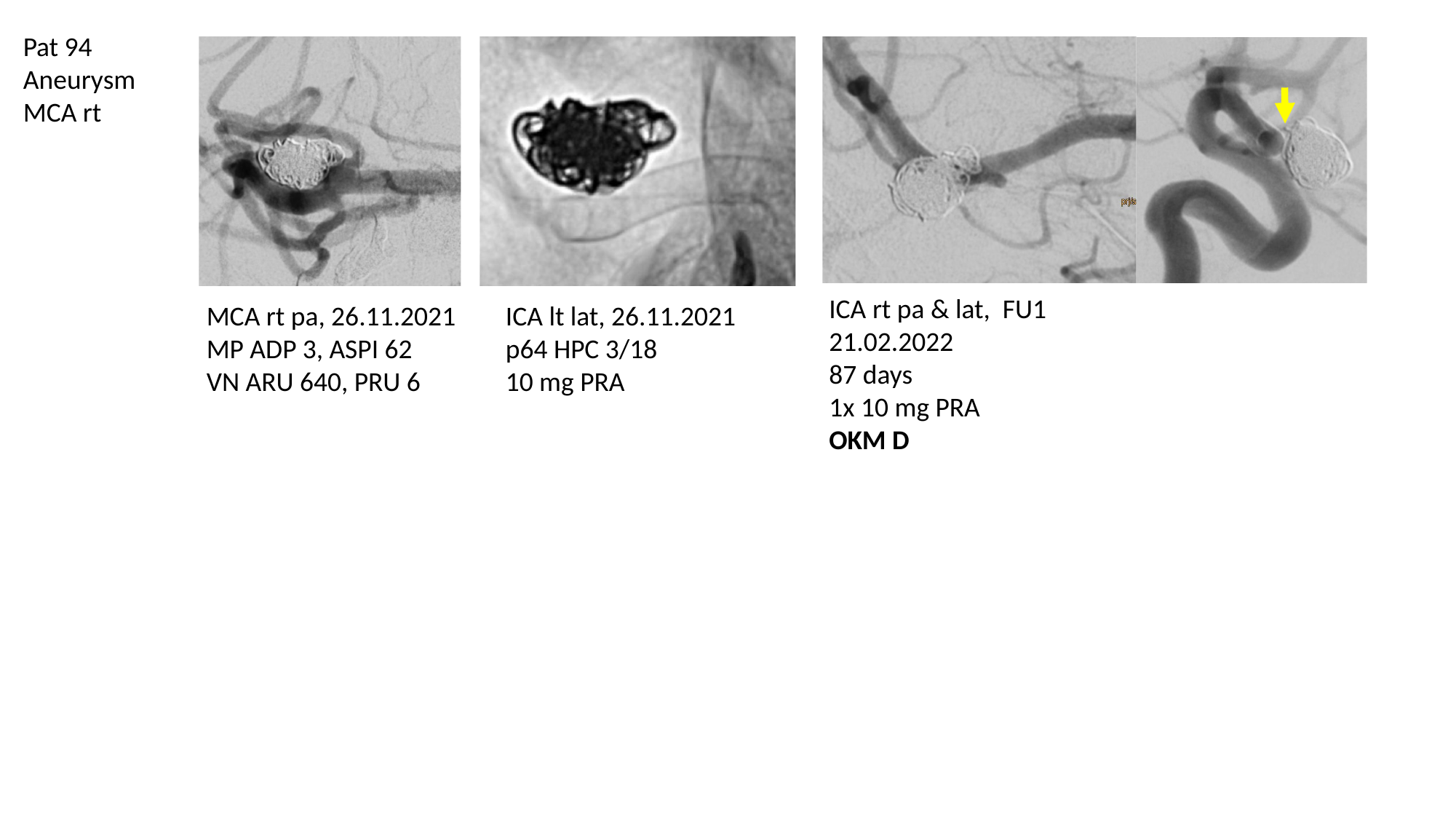

Pat 94
Aneurysm
MCA rt
ICA rt pa & lat, FU1
21.02.2022
87 days
1x 10 mg PRA
OKM D
ICA lt lat, 26.11.2021
p64 HPC 3/18
10 mg PRA
MCA rt pa, 26.11.2021
MP ADP 3, ASPI 62
VN ARU 640, PRU 6

## Slide 125
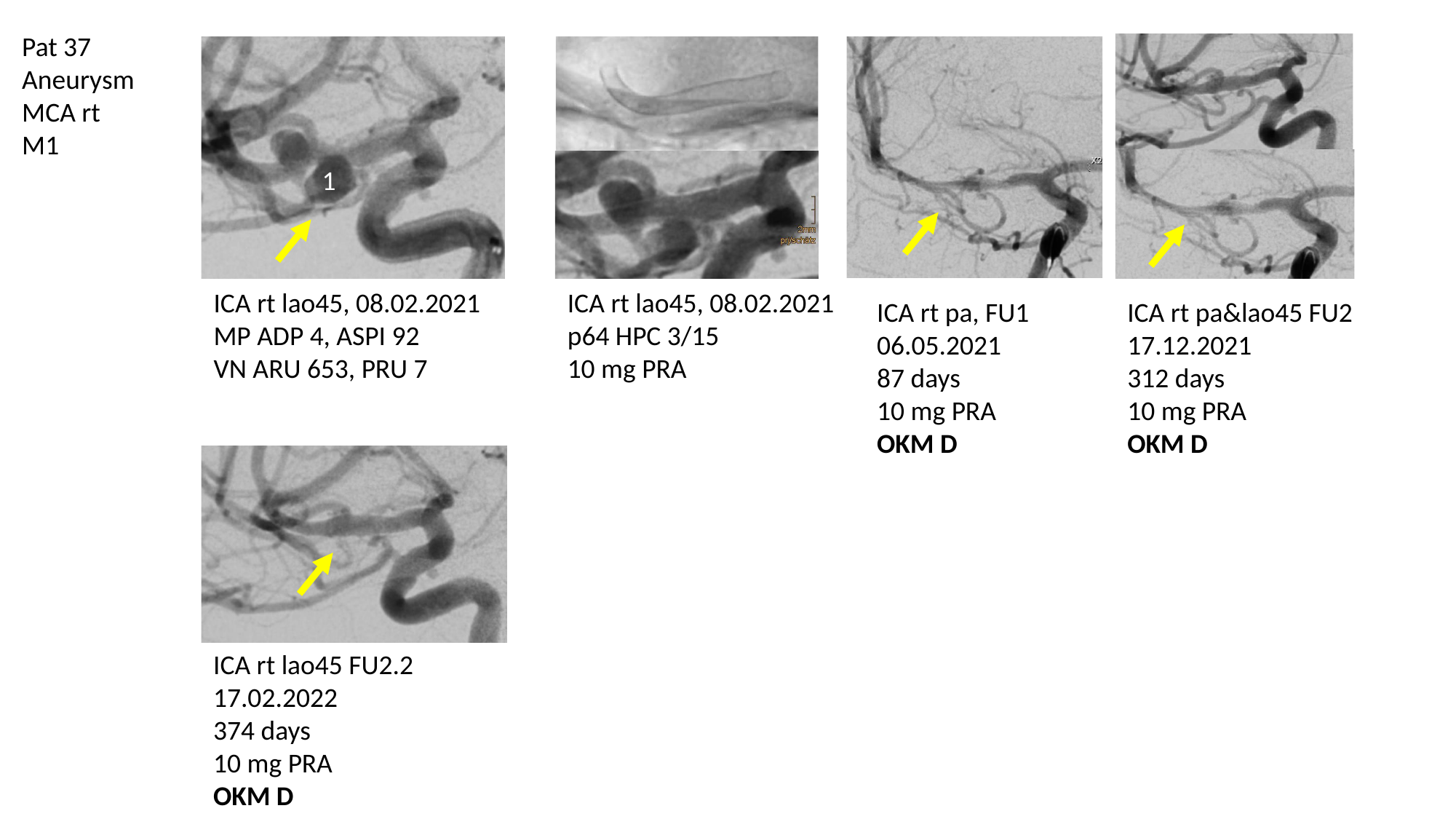

Pat 37
Aneurysm
MCA rt
M1
1
ICA rt lao45, 08.02.2021
MP ADP 4, ASPI 92
VN ARU 653, PRU 7
ICA rt lao45, 08.02.2021
p64 HPC 3/15
10 mg PRA
ICA rt pa, FU1
06.05.2021
87 days
10 mg PRA
OKM D
ICA rt pa&lao45 FU2
17.12.2021
312 days
10 mg PRA
OKM D
ICA rt lao45 FU2.2
17.02.2022
374 days
10 mg PRA
OKM D

## Slide 126
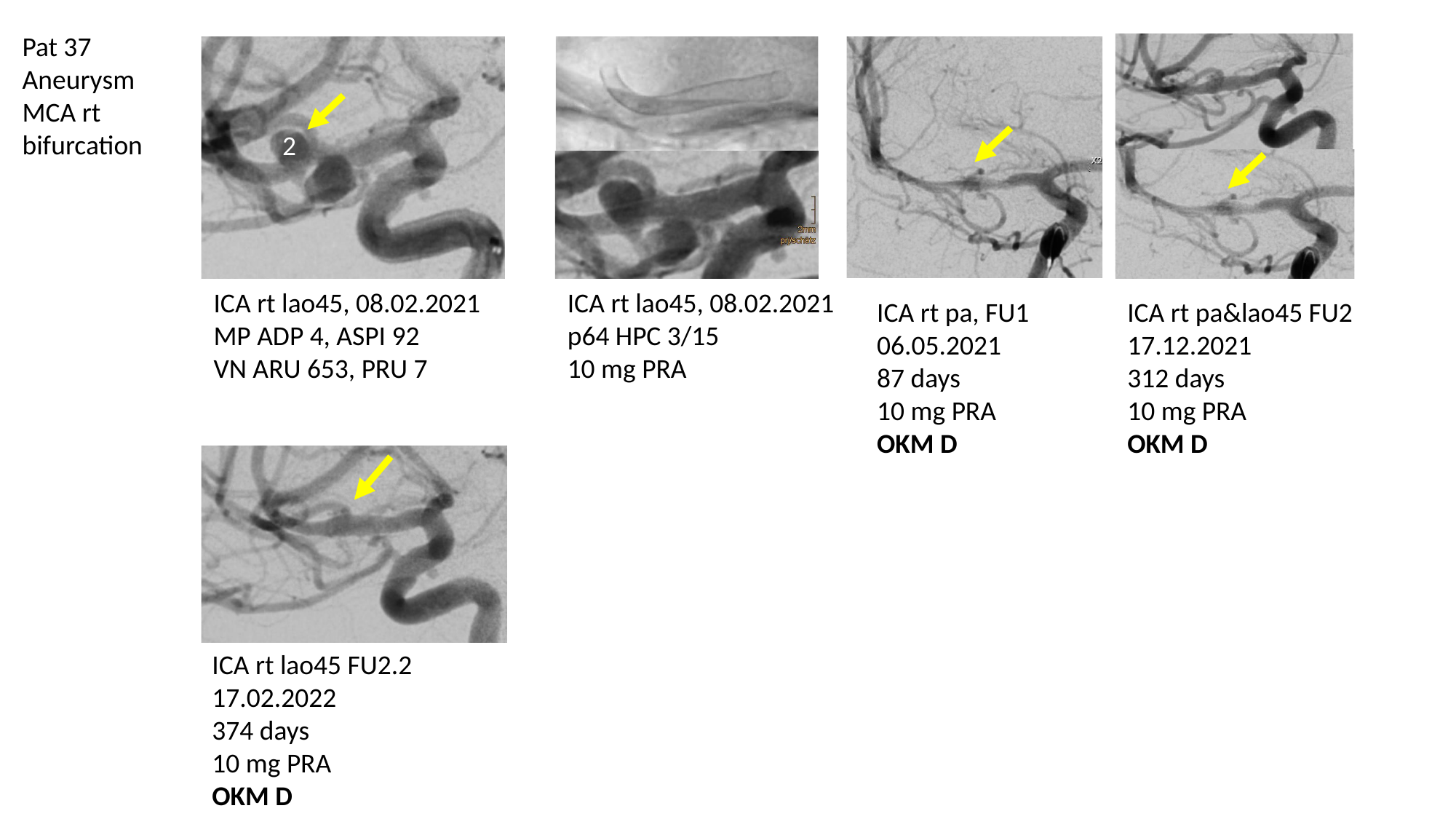

Pat 37
Aneurysm
MCA rt
bifurcation
2
ICA rt lao45, 08.02.2021
MP ADP 4, ASPI 92
VN ARU 653, PRU 7
ICA rt lao45, 08.02.2021
p64 HPC 3/15
10 mg PRA
ICA rt pa, FU1
06.05.2021
87 days
10 mg PRA
OKM D
ICA rt pa&lao45 FU2
17.12.2021
312 days
10 mg PRA
OKM D
ICA rt lao45 FU2.2
17.02.2022
374 days
10 mg PRA
OKM D

## Slide 127
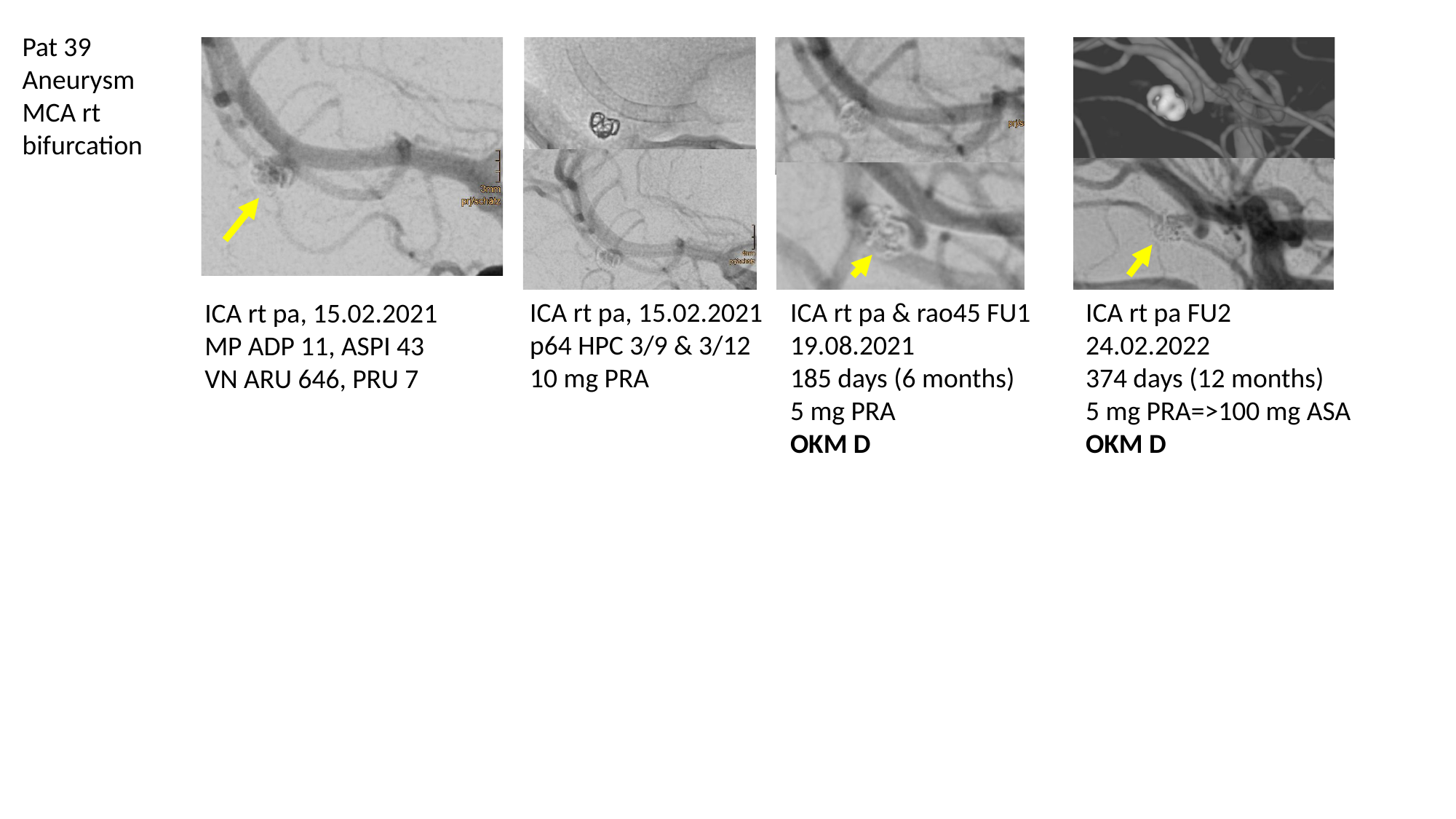

Pat 39
Aneurysm
MCA rt
bifurcation
ICA rt pa FU2
24.02.2022
374 days (12 months)
5 mg PRA=>100 mg ASA
OKM D
ICA rt pa, 15.02.2021
p64 HPC 3/9 & 3/12
10 mg PRA
ICA rt pa & rao45 FU1
19.08.2021
185 days (6 months)
5 mg PRA
OKM D
ICA rt pa, 15.02.2021
MP ADP 11, ASPI 43
VN ARU 646, PRU 7

## Slide 128
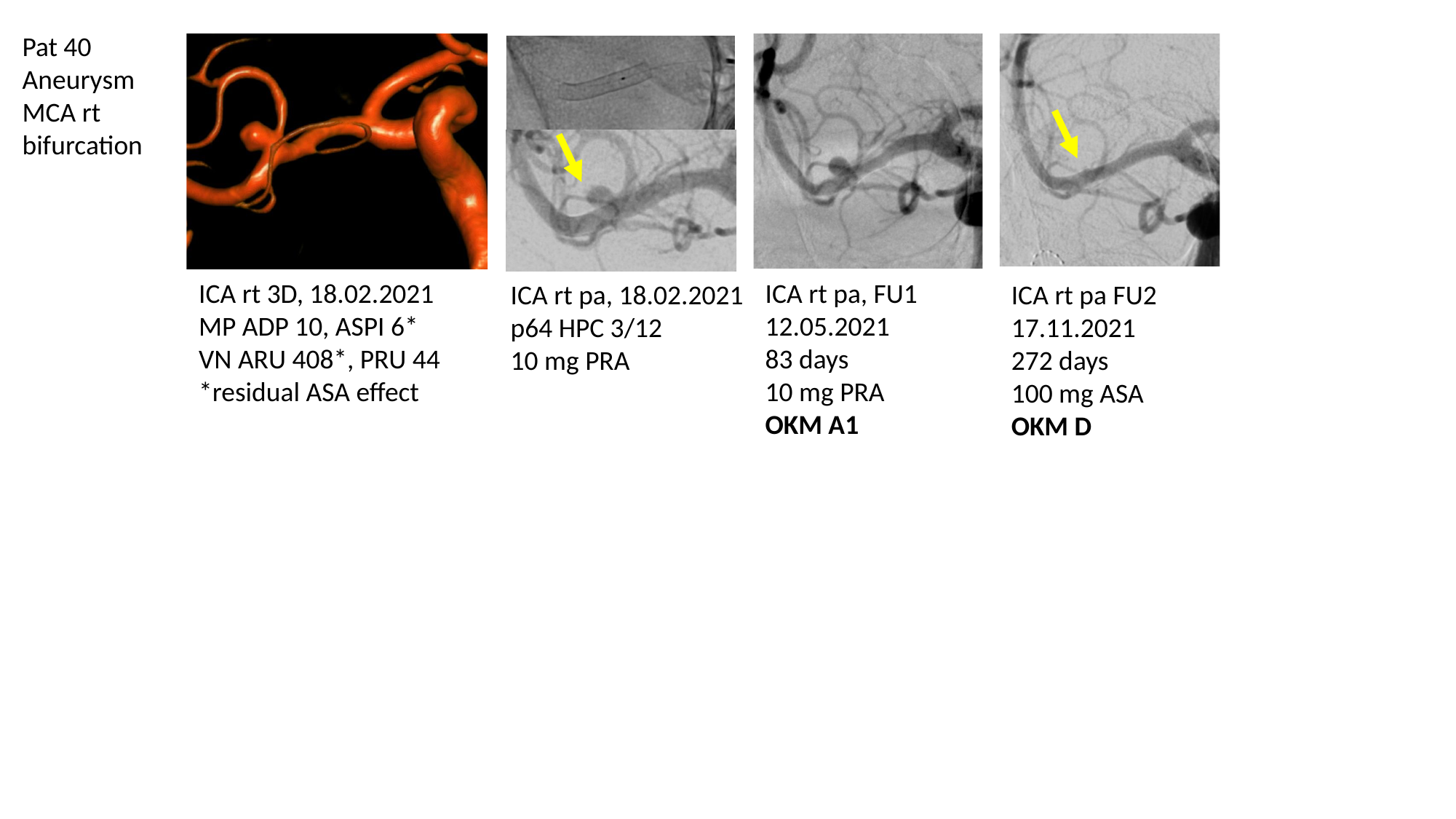

Pat 40
Aneurysm
MCA rt
bifurcation
ICA rt 3D, 18.02.2021
MP ADP 10, ASPI 6*
VN ARU 408*, PRU 44
*residual ASA effect
ICA rt pa, FU1
12.05.2021
83 days
10 mg PRA
OKM A1
ICA rt pa, 18.02.2021
p64 HPC 3/12
10 mg PRA
ICA rt pa FU2
17.11.2021
272 days
100 mg ASA
OKM D

## Slide 129
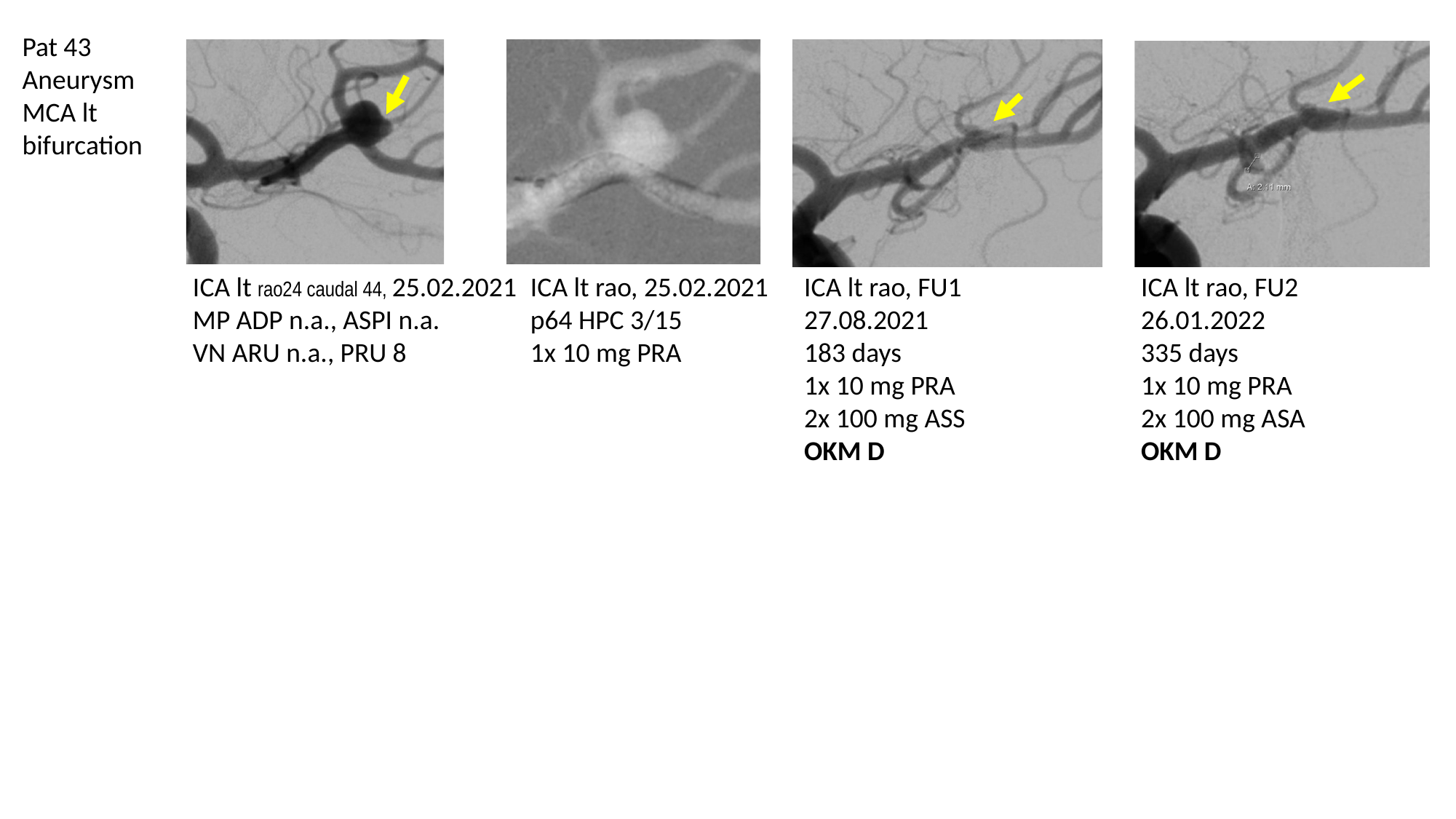

Pat 43
Aneurysm
MCA lt
bifurcation
ICA lt rao, 25.02.2021
p64 HPC 3/15
1x 10 mg PRA
ICA lt rao, FU1
27.08.2021
183 days
1x 10 mg PRA
2x 100 mg ASS
OKM D
ICA lt rao, FU2
26.01.2022
335 days
1x 10 mg PRA
2x 100 mg ASA
OKM D
ICA lt rao24 caudal 44, 25.02.2021
MP ADP n.a., ASPI n.a.
VN ARU n.a., PRU 8

## Slide 130
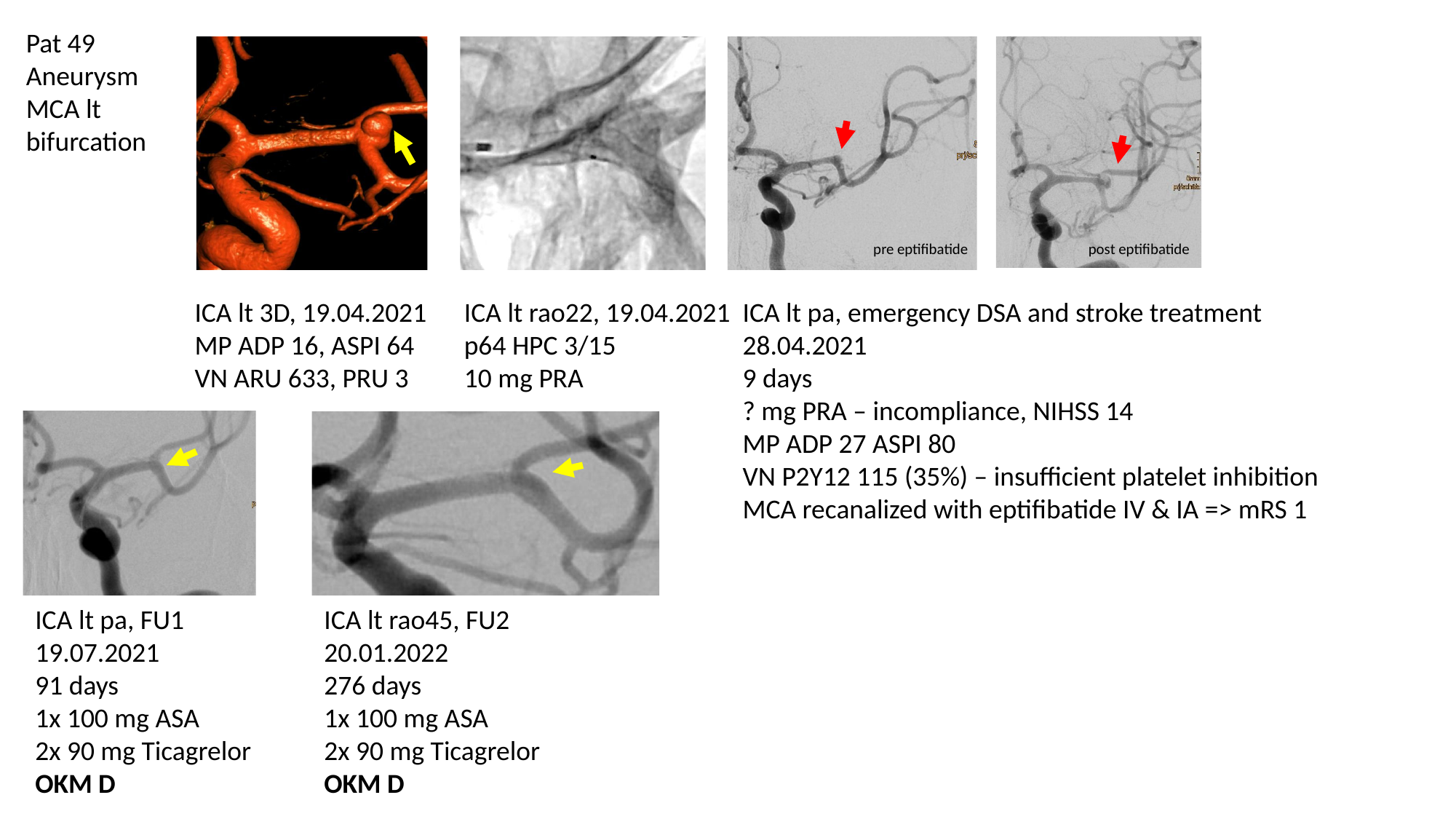

Pat 49
Aneurysm
MCA lt
bifurcation
pre eptifibatide
post eptifibatide
ICA lt rao22, 19.04.2021
p64 HPC 3/15
10 mg PRA
ICA lt pa, emergency DSA and stroke treatment
28.04.2021
9 days
? mg PRA – incompliance, NIHSS 14
MP ADP 27 ASPI 80
VN P2Y12 115 (35%) – insufficient platelet inhibition
MCA recanalized with eptifibatide IV & IA => mRS 1
ICA lt 3D, 19.04.2021
MP ADP 16, ASPI 64
VN ARU 633, PRU 3
ICA lt pa, FU1
19.07.2021
91 days
1x 100 mg ASA
2x 90 mg Ticagrelor
OKM D
ICA lt rao45, FU2
20.01.2022
276 days
1x 100 mg ASA
2x 90 mg Ticagrelor
OKM D

## Slide 131
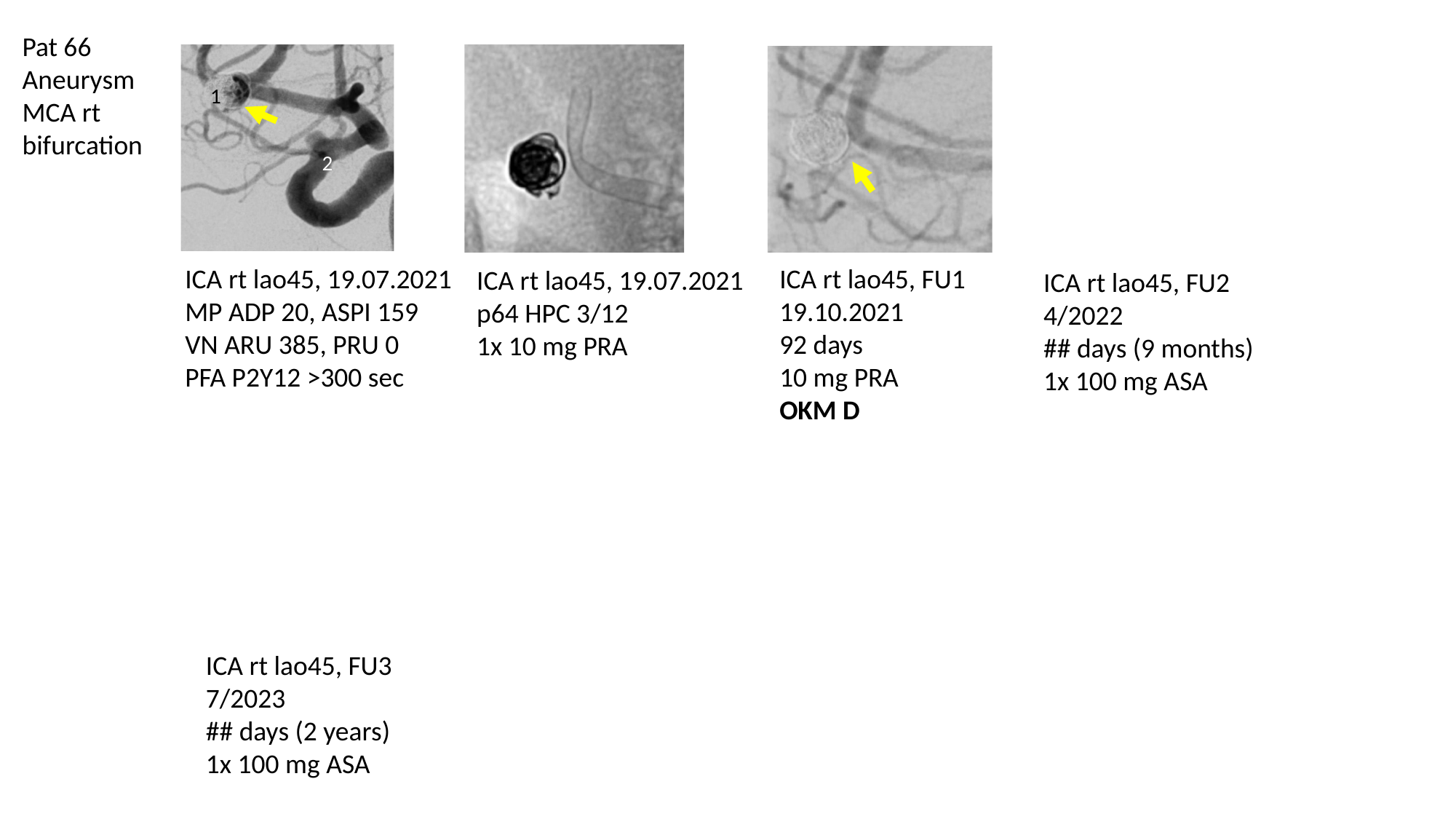

Pat 66
Aneurysm
MCA rt
bifurcation
1
2
ICA rt lao45, 19.07.2021
MP ADP 20, ASPI 159
VN ARU 385, PRU 0
PFA P2Y12 >300 sec
ICA rt lao45, FU1
19.10.2021
92 days
10 mg PRA
OKM D
ICA rt lao45, 19.07.2021
p64 HPC 3/12
1x 10 mg PRA
ICA rt lao45, FU2
4/2022
## days (9 months)
1x 100 mg ASA
ICA rt lao45, FU3
7/2023
## days (2 years)
1x 100 mg ASA

## Slide 132
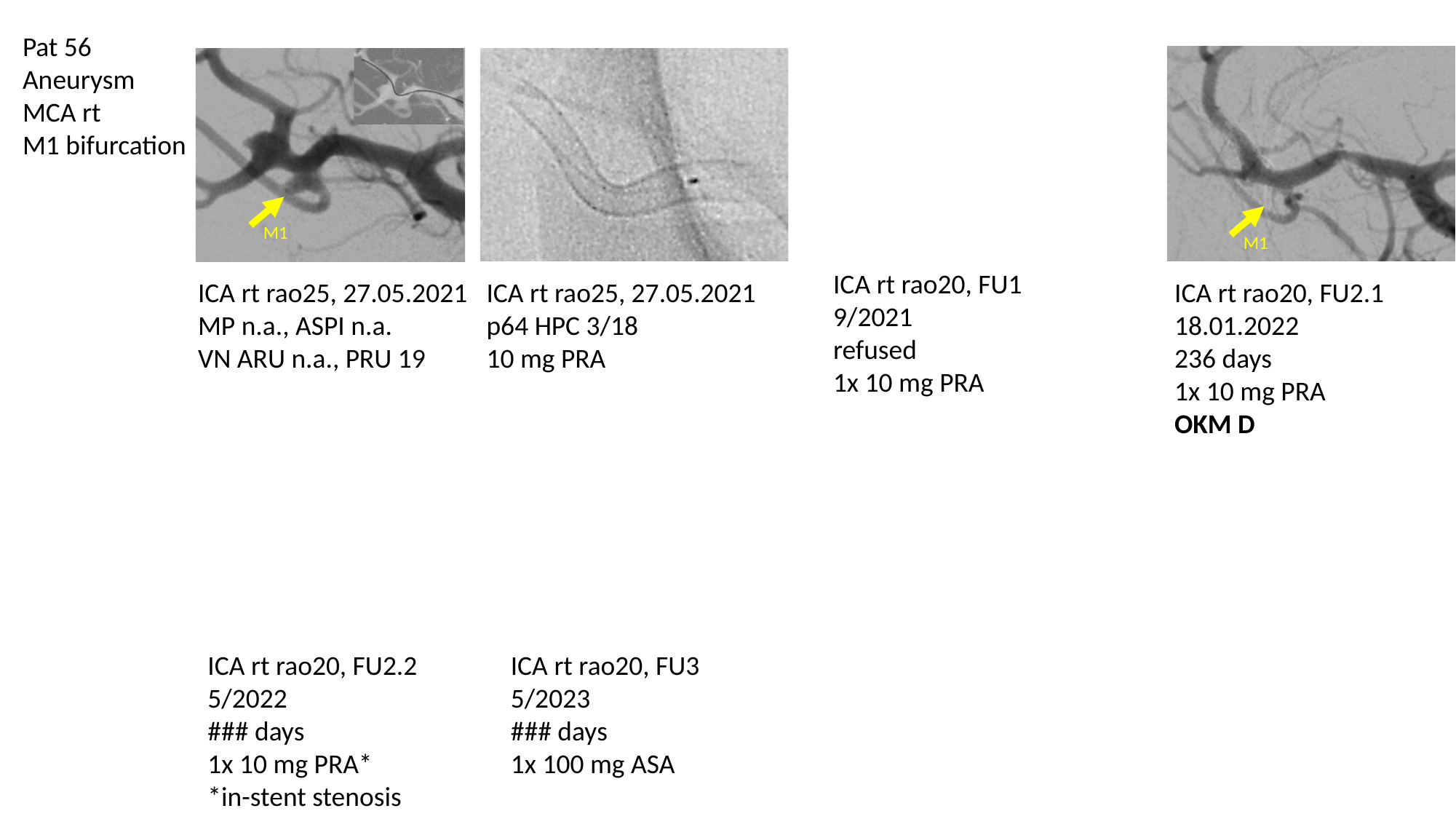

Pat 56
Aneurysm
MCA rt
M1 bifurcation
M1
M1
ICA rt rao20, FU1
9/2021
refused
1x 10 mg PRA
ICA rt rao25, 27.05.2021
MP n.a., ASPI n.a.
VN ARU n.a., PRU 19
ICA rt rao25, 27.05.2021
p64 HPC 3/18
10 mg PRA
ICA rt rao20, FU2.1
18.01.2022
236 days
1x 10 mg PRA
OKM D
ICA rt rao20, FU2.2
5/2022
### days
1x 10 mg PRA*
*in-stent stenosis
ICA rt rao20, FU3
5/2023
### days
1x 100 mg ASA

## Slide 133
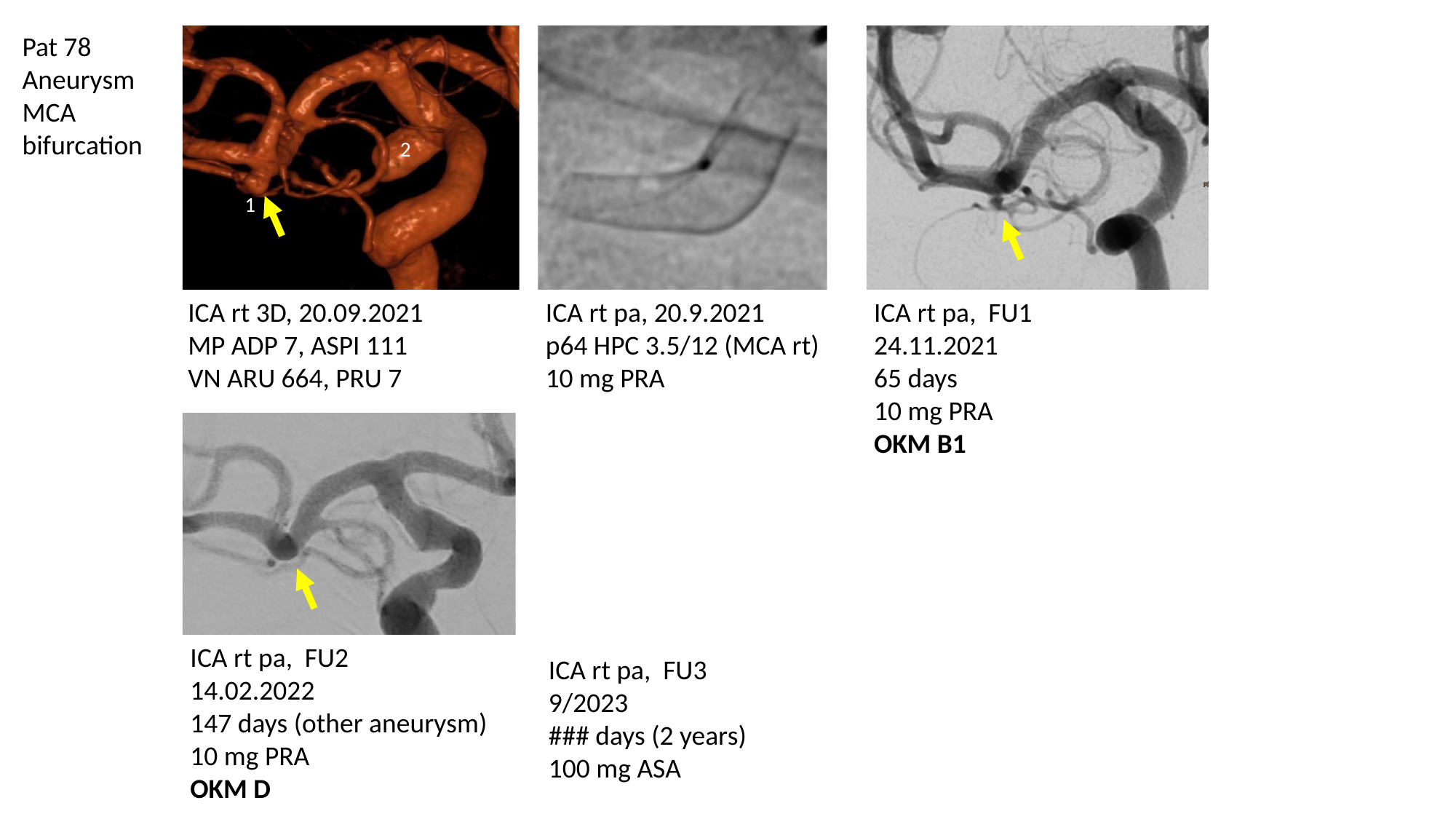

Pat 78
Aneurysm
MCA
bifurcation
2
1
ICA rt pa, FU1
24.11.2021
65 days
10 mg PRA
OKM B1
ICA rt 3D, 20.09.2021
MP ADP 7, ASPI 111
VN ARU 664, PRU 7
ICA rt pa, 20.9.2021
p64 HPC 3.5/12 (MCA rt)
10 mg PRA
ICA rt pa, FU2
14.02.2022
147 days (other aneurysm)
10 mg PRA
OKM D
ICA rt pa, FU3
9/2023
### days (2 years)
100 mg ASA

## Slide 134
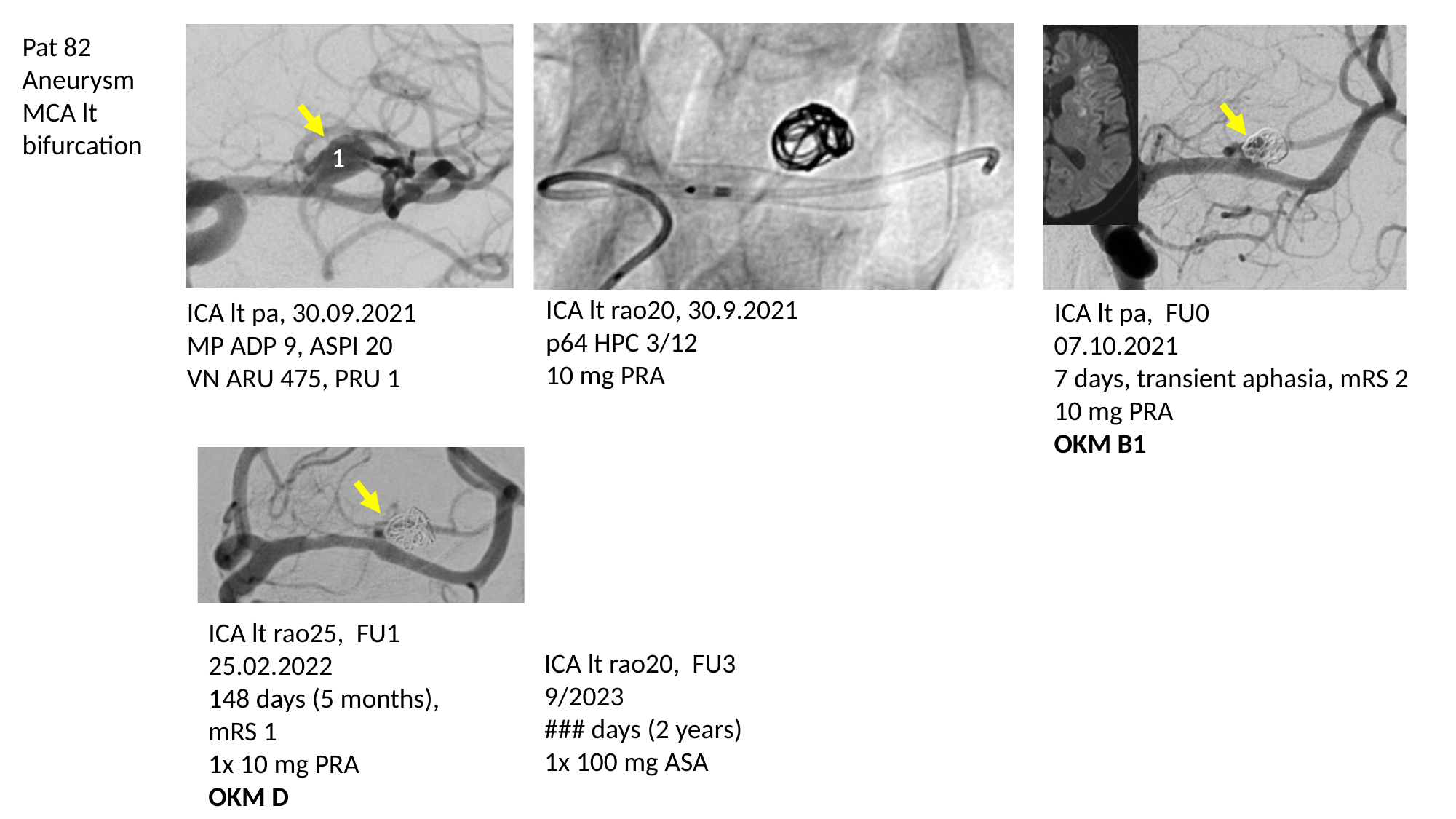

Pat 82
Aneurysm
MCA lt
bifurcation
M2
1
M1
ICA lt rao20, 30.9.2021
p64 HPC 3/12
10 mg PRA
ICA lt pa, FU0
07.10.2021
7 days, transient aphasia, mRS 2
10 mg PRA
OKM B1
ICA lt pa, 30.09.2021
MP ADP 9, ASPI 20
VN ARU 475, PRU 1
ICA lt rao25, FU1
25.02.2022
148 days (5 months), mRS 1
1x 10 mg PRA
OKM D
ICA lt rao20, FU3
9/2023
### days (2 years)
1x 100 mg ASA

## Slide 135
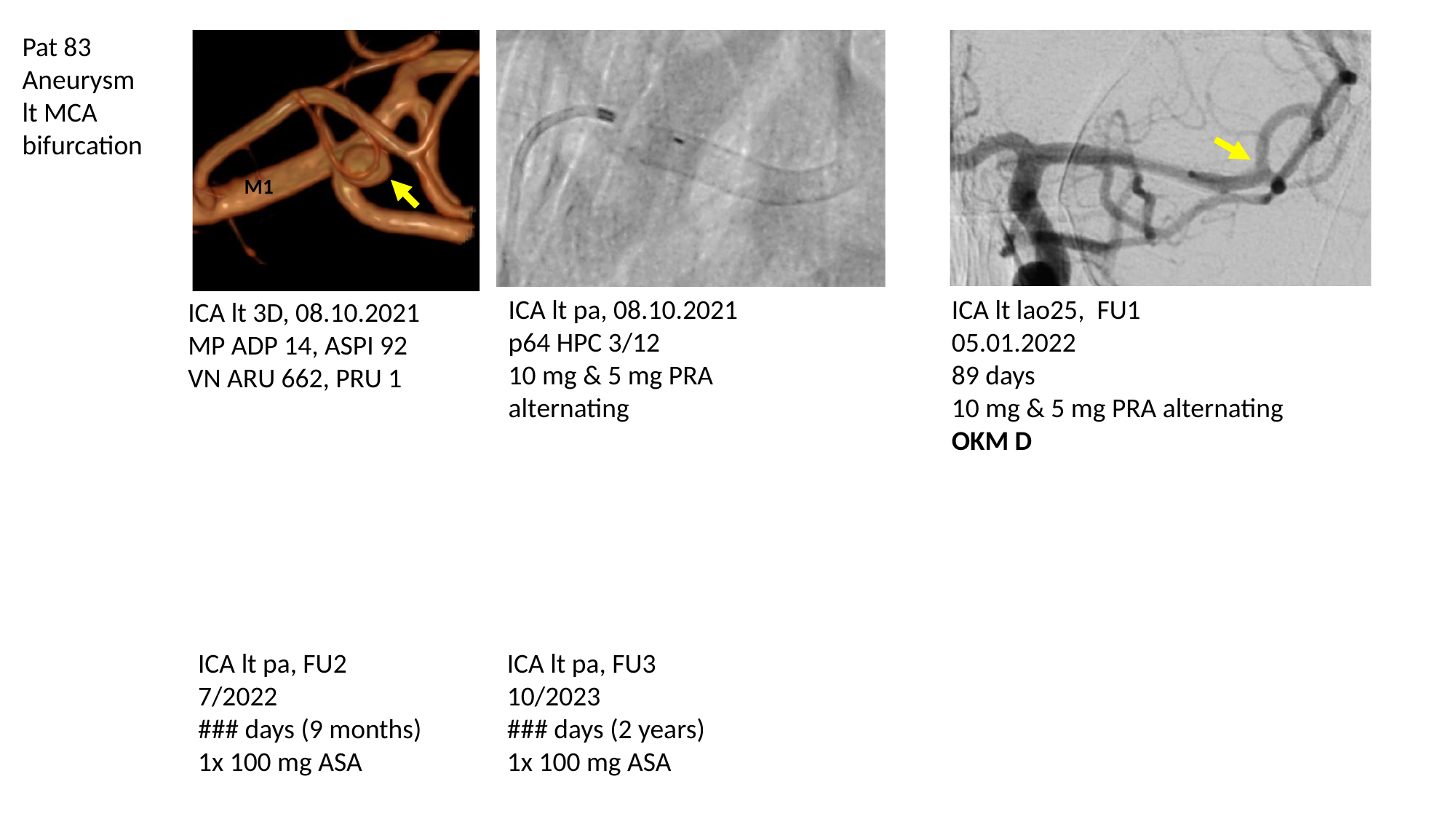

Pat 83
Aneurysm
lt MCA
bifurcation
M2
M1
M1
ICA lt pa, 08.10.2021
p64 HPC 3/12
10 mg & 5 mg PRA
alternating
ICA lt lao25, FU1
05.01.2022
89 days
10 mg & 5 mg PRA alternating
OKM D
ICA lt 3D, 08.10.2021
MP ADP 14, ASPI 92
VN ARU 662, PRU 1
ICA lt pa, FU2
7/2022
### days (9 months)
1x 100 mg ASA
ICA lt pa, FU3
10/2023
### days (2 years)
1x 100 mg ASA

## Slide 136
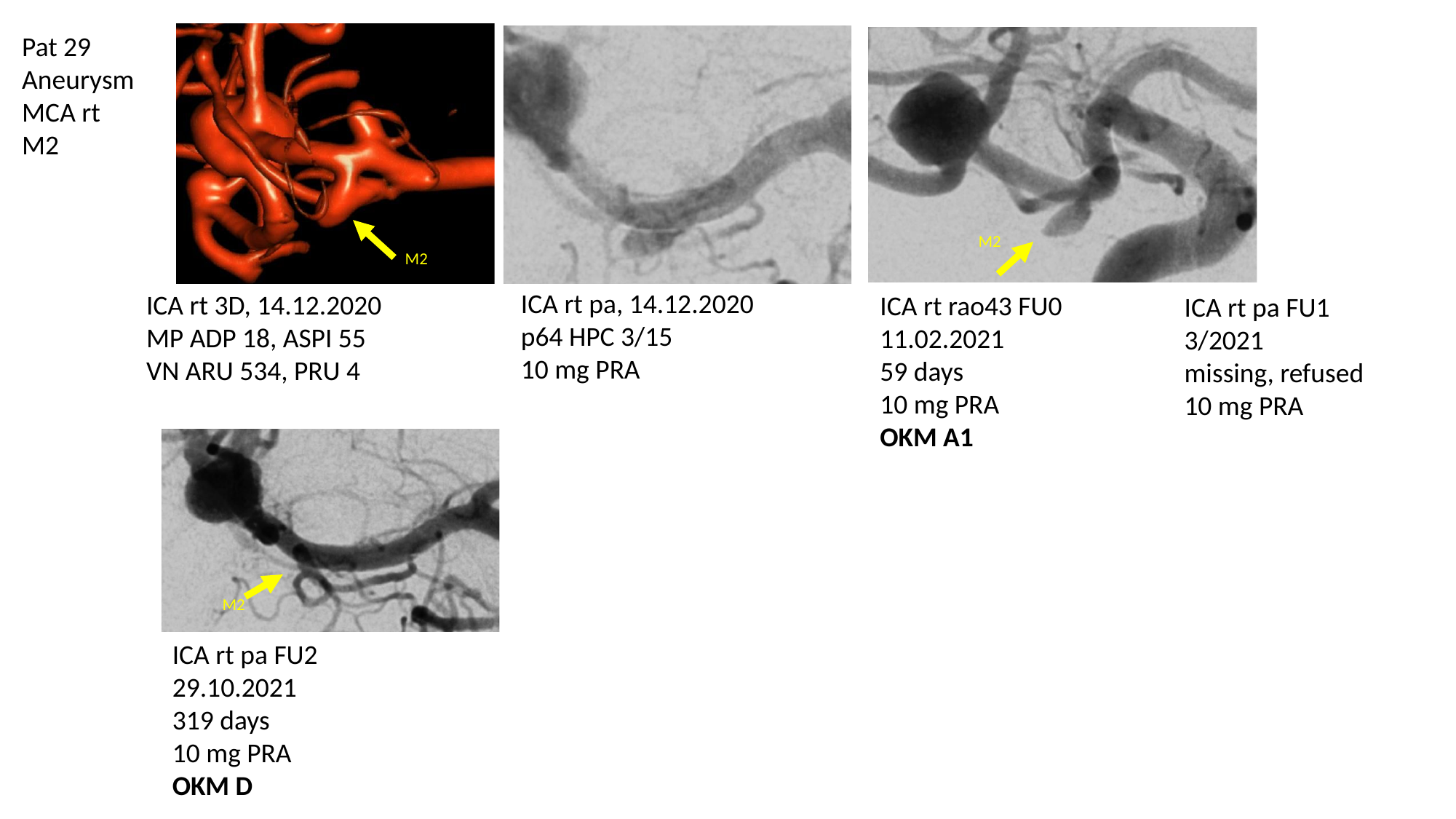

Pat 29
Aneurysm
MCA rt
M2
M2
M2
ICA rt pa, 14.12.2020
p64 HPC 3/15
10 mg PRA
ICA rt 3D, 14.12.2020
MP ADP 18, ASPI 55
VN ARU 534, PRU 4
ICA rt rao43 FU0
11.02.2021
59 days
10 mg PRA
OKM A1
ICA rt pa FU1
3/2021
missing, refused
10 mg PRA
M2
ICA rt pa FU2
29.10.2021
319 days
10 mg PRA
OKM D

## Slide 137
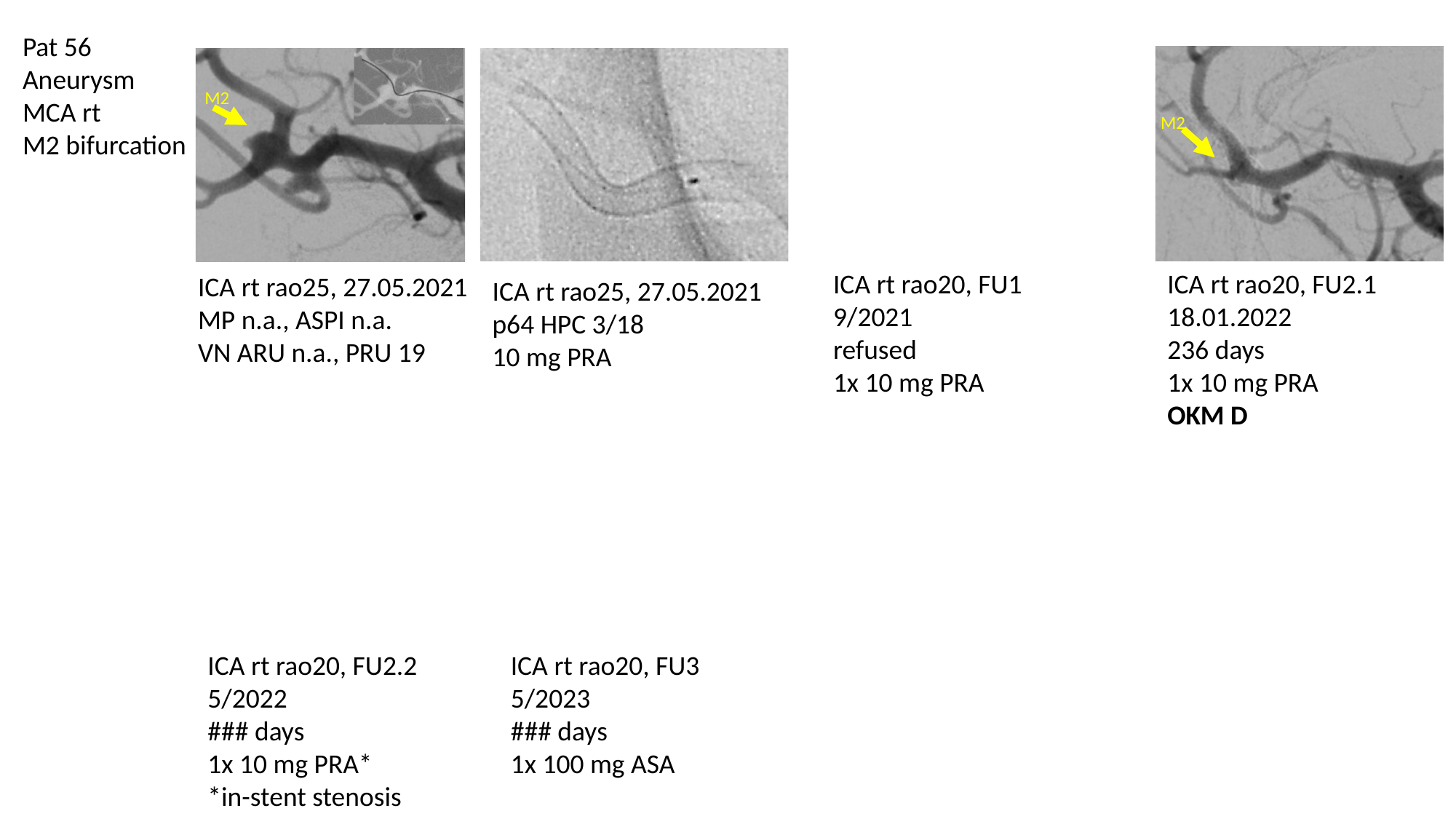

Pat 56
Aneurysm
MCA rt
M2 bifurcation
M2
M2
ICA rt rao20, FU1
9/2021
refused
1x 10 mg PRA
ICA rt rao20, FU2.1
18.01.2022
236 days
1x 10 mg PRA
OKM D
ICA rt rao25, 27.05.2021
MP n.a., ASPI n.a.
VN ARU n.a., PRU 19
ICA rt rao25, 27.05.2021
p64 HPC 3/18
10 mg PRA
ICA rt rao20, FU2.2
5/2022
### days
1x 10 mg PRA*
*in-stent stenosis
ICA rt rao20, FU3
5/2023
### days
1x 100 mg ASA

## Slide 138
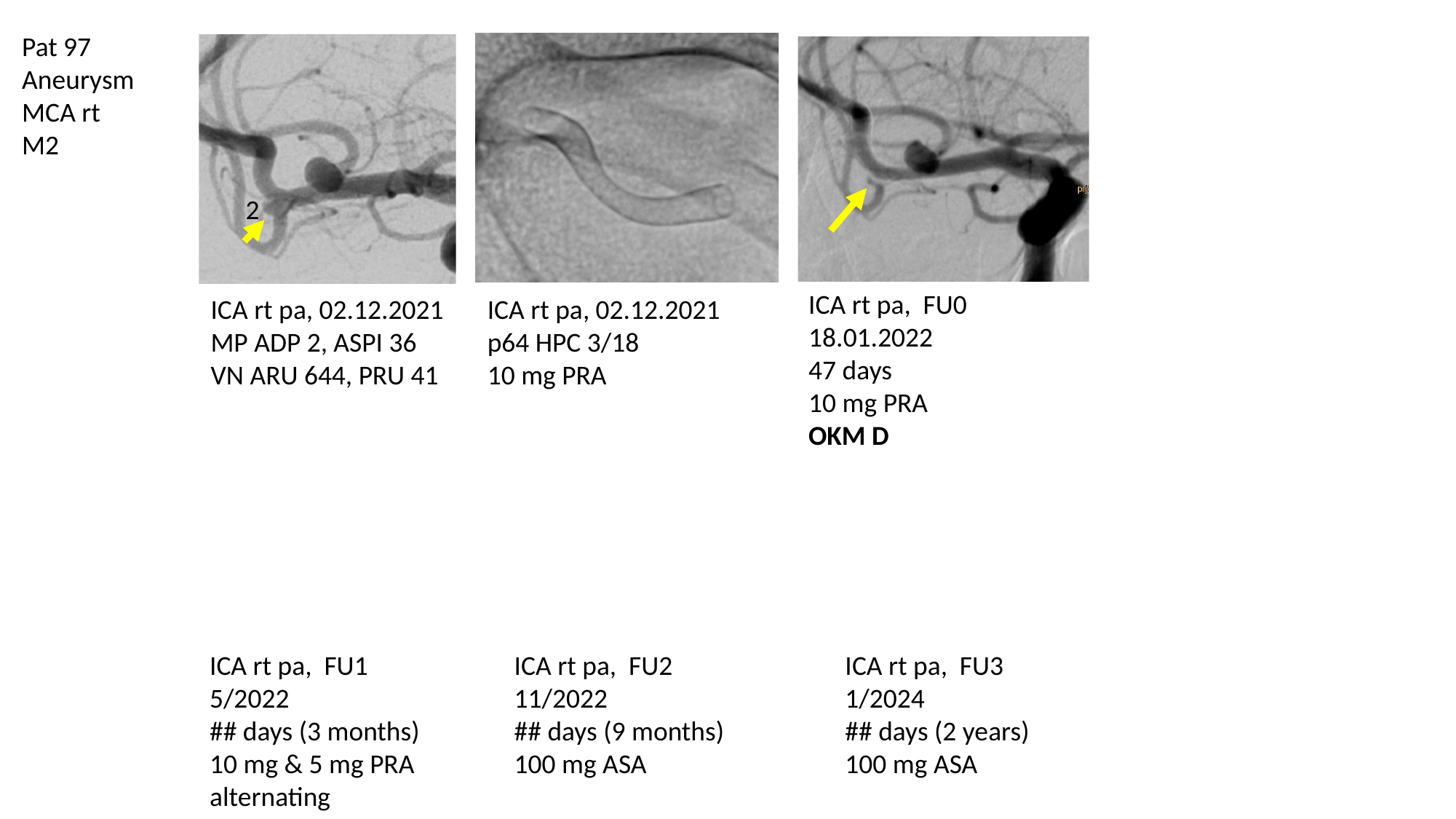

Pat 97
Aneurysm
MCA rt
M2
2
ICA rt pa, FU0
18.01.2022
47 days
10 mg PRA
OKM D
ICA rt pa, 02.12.2021
MP ADP 2, ASPI 36
VN ARU 644, PRU 41
ICA rt pa, 02.12.2021
p64 HPC 3/18
10 mg PRA
ICA rt pa, FU3
1/2024
## days (2 years)
100 mg ASA
ICA rt pa, FU1
5/2022
## days (3 months)
10 mg & 5 mg PRA
alternating
ICA rt pa, FU2
11/2022
## days (9 months)
100 mg ASA
